# Causal effects of genetically predicted type 2 diabetes mellitus on blood lipid profiles and concentration of particle-size-determined lipoprotein subclasses: A two-sample Mendelian randomization study

**DOI:** 10.3389/fcvm.2022.965995

**Published:** 2022-10-13

**Authors:** Ken Chen, Jilin Zheng, Chunli Shao, Qing Zhou, Jie Yang, Tao Huang, Yi-Da Tang

**Affiliations:** ^1^Key Laboratory of Molecular Cardiovascular Sciences, Department of Cardiology, Institute of Vascular Medicine, Ministry of Education, Peking University Third Hospital, Beijing, China; ^2^Department of Cardiology, Xinhua Hospital, Shanghai Jiaotong University School of Medicine, Shanghai, China; ^3^Department of Cardiology, State Key Laboratory of Cardiovascular Disease, Fuwai Hospital, National Center for Cardiovascular Diseases, Chinese Academy of Medical Sciences and Peking Union Medical College, Beijing, China; ^4^Graduate School of Peking Union Medical College, Chinese Academy of Medical Sciences and Peking Union Medical College, Beijing, China; ^5^Department of Epidemiology and Biostatistics, School of Public Health, Peking University, Beijing, China; ^6^Department of Global Health, School of Public Health, Peking University, Beijing, China; ^7^Center for Intelligent Public Health, Institute for Artificial Intelligence, Peking University, Beijing, China

**Keywords:** type-2 diabetes, dyslipidemia, cardiovascular disease, Mendelian randomization, triglyceride, high-density lipoprotein (HDL)

## Abstract

**Background:**

Observational studies have shown inconsistent results of the associations between type 2 diabetes mellitus (T2DM) and blood lipid profiles, while there is also a lack of evidence from randomized controlled trials (RCTs) for the causal effects of T2DM on blood lipid profiles and lipoprotein subclasses.

**Objectives:**

Our study aimed at investigating the causal effects of T2DM on blood lipid profiles and concentration of particle-size-determined lipoprotein subclasses by using the two-sample Mendelian randomization (MR) method.

**Methods:**

We obtained genetic variants for T2DM and blood lipid profiles including high-density lipoprotein-cholesterol (HDL-C), low-density lipoprotein-cholesterol (LDL-C), triglycerides (TG), and total cholesterol (TC) from international genome-wide association studies (GWASs). Two-sample MR method was applied to explore the potential causal effects of genetically predicted T2DM on blood lipid profiles based on different databases, respectively, and results from each MR analysis were further meta-analyzed to obtain the summary results. The causal effects of genetically predicted T2DM on the concentration of different subclasses of lipoproteins that are determined by particle size were also involved in MR analysis.

**Results:**

Genetically predicted 1-unit higher log odds of T2DM had a significant causal effect on a higher level of TG (estimated *β* coefficient: 0.03, 95% confidence interval [CI]: 0.00 to 0.06) and lower level of HDL-C (estimated *β* coefficient: −0.09, 95% CI: −0.11 to −0.06). The causality of T2DM on the level of TC or LDL-C was not found (estimated *β* coefficient: −0.01, 95% CI: −0.02 to 0.01 for TC and estimated *β* coefficient: 0.01, 95% CI: −0.01 to 0.02 for LDL-C). For different sizes of lipoprotein particles, 1-unit higher log odds of T2DM was causally associated with higher level of small LDL particles, and lower level of medium HDL particles, large HDL particles, and very large HDL particles.

**Conclusion:**

Evidence from our present study showed causal effects of T2DM on the level of TG, HDL-C, and concentration of different particle sizes of lipoprotein subclasses comprehensively, which might be particularly helpful in illustrating dyslipidemia experienced by patients with T2DM, and further indicate new treatment targets for these patients to prevent subsequent excessive cardiovascular events from a genetic point of view.

## Introduction

Type 2 diabetes mellitus (T2DM), a global serious condition with reduced quality of life and life expectancy, is expected to reach 550 million people by 2030 (1), and the estimated global health expenditure on T2DM will be $490 billion in 2030 (2). The mortality of cardiovascular disease (CVD) in patients with T2DM was 2–4 times higher compared with non-diabetic population (3, 4). These observational studies arouse the recognition of T2DM as an essential risk factor for CVD.

Dyslipidemia, one of the key cardiometabolic risk factors, was found to be associated with excessive CVD risk in patients with T2DM (5). Dyslipidemia included disorders of high-density lipoprotein-cholesterol (HDL-C), low-density lipoprotein-cholesterol (LDL-C), very low-density lipoprotein-cholesterol (VLDL-C), triglycerides (TG) and total cholesterol (TC). Among these blood lipid profiles, LDL-C was proved to be a major atherogenic lipoprotein which was also a key risk factor for CVD. Reducing the level of LDL-C of patients with T2DM showed significant benefit in CVD risk reduction according to previous randomized controlled trials (RCTs) (6, 7), while evidence supporting the benefits of control of other blood lipid profiles was inadequate. Only LDL-C lowering treatment was recommended by current guidelines to reduce the excessive risk of CVD among patients with T2DM (8–10). However, the Framingham Heart Study found no statistically significant difference in the level of LDL-C and TC in patients with T2DM compared to those without T2DM. Significantly higher level of TG and lower level of HDL-C were found among patients with T2DM compared to patients with non-diabetes instead (11), which were further supported by subsequent observational studies (12–15). The above studies indicated that the efficacy of lowering LDL-C to attenuate excessive CVD risk among patients with T2DM still needs to be reconfirmed, while potential effects of other types of lipid profiles on excessive CVD risk should be taken into consideration seriously. In addition, as the technology of lipoprotein profiling advanced in recent years, measuring the concentration of lipoprotein particles and sub-particles that are related to cardiometabolic risks might provide better predictors of CVD risks among patients with T2DM than traditional lipid panels (16–18). However, the previous observational studies only displayed the associations of T2DM with alterations of blood lipid profiles, without uncovering the causal effects of T2DM on various blood lipid profiles and different sizes of lipoproteins sub-particles remained largely unexamined.

Mendelian randomization (MR) is a genetic epidemiological method to explore the causality of an exposure on an outcome using genetic variants as instrumental variables (19). Unlike observational studies which are more prone to be biased by reverse causality, measurement error, and other confounding factors, the genetic variants used in MR were determined at conception which had lifelong influence and thus could largely avoid reverse causality and other confounding factors. MR could provide efficient and robust results that closely resemble those of RCTs as nature's randomized trials as there is currently a lack of well-conducted, high-quality RCTs through which to investigate the causal relationships of T2DM and blood lipid profiles.

Therefore, to provide more efficient and precise treatment strategies to reduce the excessive CVD risk in patients with T2DM, we conducted a two-sample MR study by analyzing genome-wide association studies (GWASs) summary statistics from international genetic consortia to further explore the potential causal effects of genetically predicted T2DM on genetically predicted various blood lipid profiles as well as genetically determined different particle sizes of lipoproteins.

## Methods

### Overall study design

**Figure 1:**
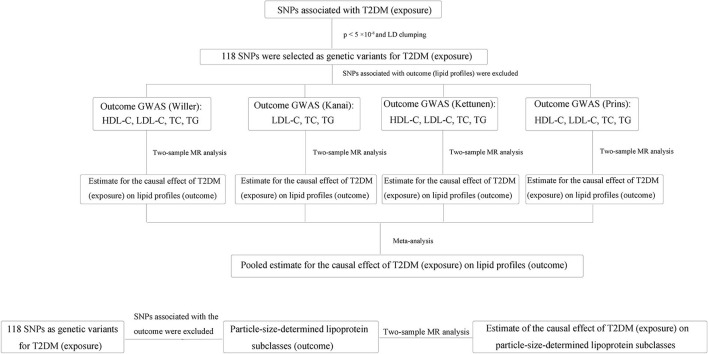
Flow diagram of the overview of our study design. First, we conducted a two-sample MR study to investigate the potential causal effects of genetically predicted T2DM on genetically predicted blood lipid profiles according to different large GWAS data resources, respectively. Then, we performed the overall meta-analysis to obtain the summary results by meta-analyzing each MR result to determine those causal effects more comprehensively across different data sources. In addition, the estimates of the causal effect of T2DM on each particle-size-determined lipoprotein subclass were also made via the two-sample MR method. SNP, single-nucleotide polymorphism; T2DM, type 2 diabetes mellitus; GWAS, genome-wide association study; HDL, high-density lipoprotein; LDL, low-density lipoprotein; TC, total cholesterol; TG, triglycerides; MR, Mendelian randomization.

First, we conducted a two-sample MR study by using genetic instrumental variables as proxies for T2DM and blood lipid profiles to investigate the potential causal effects of genetically predicted T2DM on genetically predicted blood lipid profiles according to different large GWAS data resources, respectively. Then, we performed an overall meta-analysis to obtain the summary results by meta-analyzing each MR result to determine those causal effects more comprehensively across different data sources. An overview of our study design is shown in Figure 1.

## Data sources

### Genetic instrumental variable for T2DM

We searched GWAS summarized data and extracted 143 single-nucleotide polymorphisms (SNPs) from the latest analysis including 62,892 patients with T2DM and 596,424 controls of European ancestry (20). Among these SNPs, 118 were finally used for genetic variants for T2DM which met the standard of genome-wide significance (*p*-value <5 × 10^−8^) and not in linkage disequilibrium (r^2^ < 0.001). The analysis we selected was larger in scale than previous studies and explained 10% of the heritability of T2DM (20) which was almost two times than previous GWAS (21–23). Genetic variants used as proxies for T2DM in our study are shown in Supplementary Table 1.

### GWAS summary statistics for blood lipid profiles

Genome-wide association studies' summary statistics for HDL-C, LDL-C, TC, and TG were obtained from various sources including: (1) a GWAS including 1,88,578 European ancestry individuals and 7,898 non-European ancestry individuals (24); (2) 24,925 European ancestry participants from a GWAS (25); (3) 9,961 European ancestry individuals from the UK Household Longitudinal Study (26); and (4) a GWAS including 1,62,255 Asian ancestry individuals (27). The GWAS including 24,925 individuals further categorized LDL and HDL according to the particle size of lipoproteins, and we took each category (e.g., small LDL, medium LDL, and large LDL) into subsequent subgroup analysis. Summary information of the GWAS sources for outcomes in our study are shown in Table 1.

**Table 1:** Summary information of GWAS for blood lipid profiles.

	**Author**		**Sample size**	**Ancestry**
1	Willer et al. (24)		18,8,578	Mostly European
2	Kettunen et al. (25)		24,925	European
3	Prins et al. (26)		9,961	European
4	Kanai et al. (27)		1,62,255	European

### Statistical analysis

All the analyses in our study were performed by using R version 4.0.3 (The R Foundation for Statistical Computing) through TwoSampleMR and Meta package.

### Mendelian randomization

The MR method used in our study was based on three assumptions: (1) the genetic variants used as instrumental variables are associated with the exposure; (2) the genetic variants are not associated with other confounders; and (3) the genetic variants are only associated with the outcome through the exposure (28). In addition, estimated SNPs were used to calculate the causal effects of exposure (genetically predicted T2DM) on the outcome (genetically predicted level of blood lipid profiles and concentration of particle-size-determined lipoprotein subclasses) using an inverse-variance weighted (IVW) which combined the estimates from each SNP based on summarized GWAS database. For binary exposure (T2DM), MR estimates are odds ratios (ORs) per genetically predicted unit difference in log odds of having the relevant exposure (T2DM). Thus, causal estimates are presented as the association of one unit genetically predicted higher log odds of T2DM with genetically predicted levels of various blood lipid profiles and concentration of particle-size-determined lipoprotein subclasses. We scaled genetically predicted effect size of the genetic variant of exposure (T2DM) on the continuous outcome (levels of various blood lipid profiles and concentrations of particle-size-determined lipoprotein subclasses) as one copy addition of the effect allele of exposure on standard deviation units difference in outcome according to the database resources we referred to. IVW method was applied as the main analysis because of its efficiency, but it must satisfy all three assumptions for MR (29). Results of IVW were expressed as estimated *β* coefficient, standard error, 95% confidence interval (CI) keeping two decimals and *p*-value. *P*-value <0.05 indicates statistical significance. It is noteworthy that even one invalid genetic instrument may cause bias for IVW results (29). In addition, if the strength of genetic variants is not strong enough, this “weak instrument bias” may also cause bias. Besides, horizontal pleiotropy may also greatly influence the results of IVW. Therefore, we applied multiple sensitivity analyses to evaluate those possible biases.

### MR sensitivity analysis

We performed various MR sensitivity analyses to testify whether our results were influenced by the violation of the three assumptions of MR. F-statistic for each SNP was calculated to examine whether weak instrument bias might influence IVW results. F-statistic larger than 10 indicates less prone to weak instrument bias (30). Simple median estimator and weighted median estimator analysis were also performed and the estimated results would be consistent with IVW if at least more than half of the genetic variants were valid. Briefly speaking, a simple median can be regarded as a certain situation that weighted median estimators have equal weights (31). We applied the weighted median method to explore whether the results of IVW were influenced by an invalid instrument. The *p*-value of both IVW and weighted median was <0.05, indicating that the result of IVW was not likely to be influenced by invalid instrument bias. MR-Egger regression and the intercept of MR-Egger estimates the horizontal pleiotropy which is the main source of bias for MR studies (32). The *p*-value of MR-Egger regression larger than 0.05 indicated horizontal pleiotropy, and the *p*-value of MR-Egger intercept suggested whether such pleiotropy was statistically significant. Mendelian randomization pleiotropy residual sum and outlier (MR-PRESSO) was designed to detect and correct for pervasive horizontal pleiotropy (33). A unique test in MR-PRESSO called “the distortion test” allows it to evaluate whether the difference between the causal estimate before and after the removal of outliers is significant. Whether our results were influenced by horizontal pleiotropy was carefully examined according to the results of MR-Egger regression, MR-Egger intercept, and MR-PRESSO. Besides, we still applied single-SNP analysis to identify the association between each genetic variant and the outcome and leave-one-out analysis to explore whether the causality of the exposure on the outcome is mainly credited to a certain genetic variant. All the MR analyses were performed in the “TwoSampleMR” package in R software.

### Meta-analysis of the estimates from various outcome sources

We performed MR analyses to identify the causality of T2DM on each blood lipid profile according to each outcome source, respectively, and these results were further meta-analyzed to obtain the pooled estimates for the causality of T2DM on each blood lipid profile. *I*^2^ statistics were calculated to quantify the heterogeneity between estimates from different outcome sources. All meta-analyses were performed using the “Meta” package in R software.

## Results

### Pooled estimate of the causal effect of T2DM on blood lipid profiles

**Figure 2:**
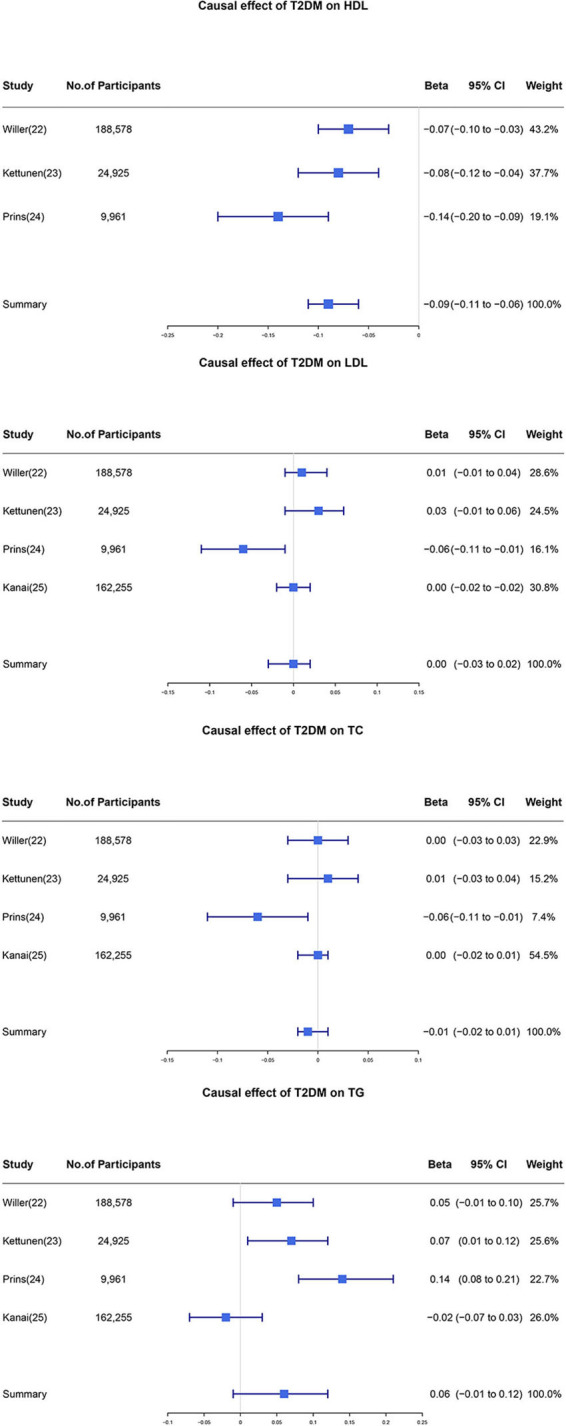
Individual and pooled estimate of the causal effect of T2DM on each blood lipid profile. Firstly we applied the two-sample MR method to estimate the causal effect of T2DM on each blood lipid profile. Then these individual estimates were further meta-analyzed to obtain the pooled results. T2DM, type 2 diabetes mellitus; HDL, high-density lipoprotein; LDL, low-density lipoprotein; TC, total cholesterol; TG, triglycerides; CI, confidence interval.

Estimate of the causal effect of T2DM on each data source was made individually and details are shown in Supplementary Tables 2–16, and genetic association for the causal effect of T2DM on blood lipid profiles using the IVW method in each data source is shown in Table 2. Pooled *β* for HDL-C, LDL-C, TC, and TG per unit increase in log odds of T2DM was −0.09 (95% CI: −0.11 to −0.06, *I*^2^ = 62%, *p-*value = 0.07), 0.00 (95% CI: −0.03 to 0.02, *I*^2^ = 67%, *p*-value = 0.03), −0.01 (95% CI: −0.02 to 0.01, *I*^2^ = 35%, *p*-value = 0.20), and 0.06 (95% CI: −0.01 to 0.12, *I*^2^ = 80%, *p*-value < 0.01), respectively (Figure 2). Random-effect model meta-analyses were used due to the heterogeneity across databases in terms of LDL-C and TG when pooling the primary analyses results using the IVW method. Fix-effect model meta-analyses were used because the heterogeneity in terms of HDL-C and TC was minor across databases when the primary analyses were pooled using the IVW method.

**Table 2:** Genetic association for the causal effect of T2DM on blood lipid profiles using IVW method in each data source.

**Author**		***β***	**SE**	***P-*** **value**
Willer	HDL-C	−0.07	0.02	<0.001
	LDL-C	0.01	0.01	0.418
	TC	6.04 × 10^−04^	0.02	0.969
	TG	0.05	0.03	0.09
Kettunen	HDL-C	−0.08	0.02	<0.001
	LDL-C	0.02	0.02	0.146
	TC	5.25 × 10^−03^	0.02	0.782
	TG	0.06	0.03	0.02
Prins	HDL-C	−0.15	0.03	<0.001
	LDL-C	−0.07	0.03	0.008
	TC	−0.06	0.03	0.025
	TG	0.14	0.03	<0.001
Kanai	LDL-C	3.00 × 10^−04^	0.01	0.974
	TC	−4.89 × 10^−03^	0.01	0.625
	TG	−0.02	0.03	0.41

T2DM, Type 2 diabetes mellitus; IVW, inverse variance weighted; HDL, high-density lipoprotein; LDL, low-density lipoprotein; TC, total cholesterol; TG, triglycerides; SE, standard error.

### Sensitivity analysis for heterogeneity

**Figure 3:**
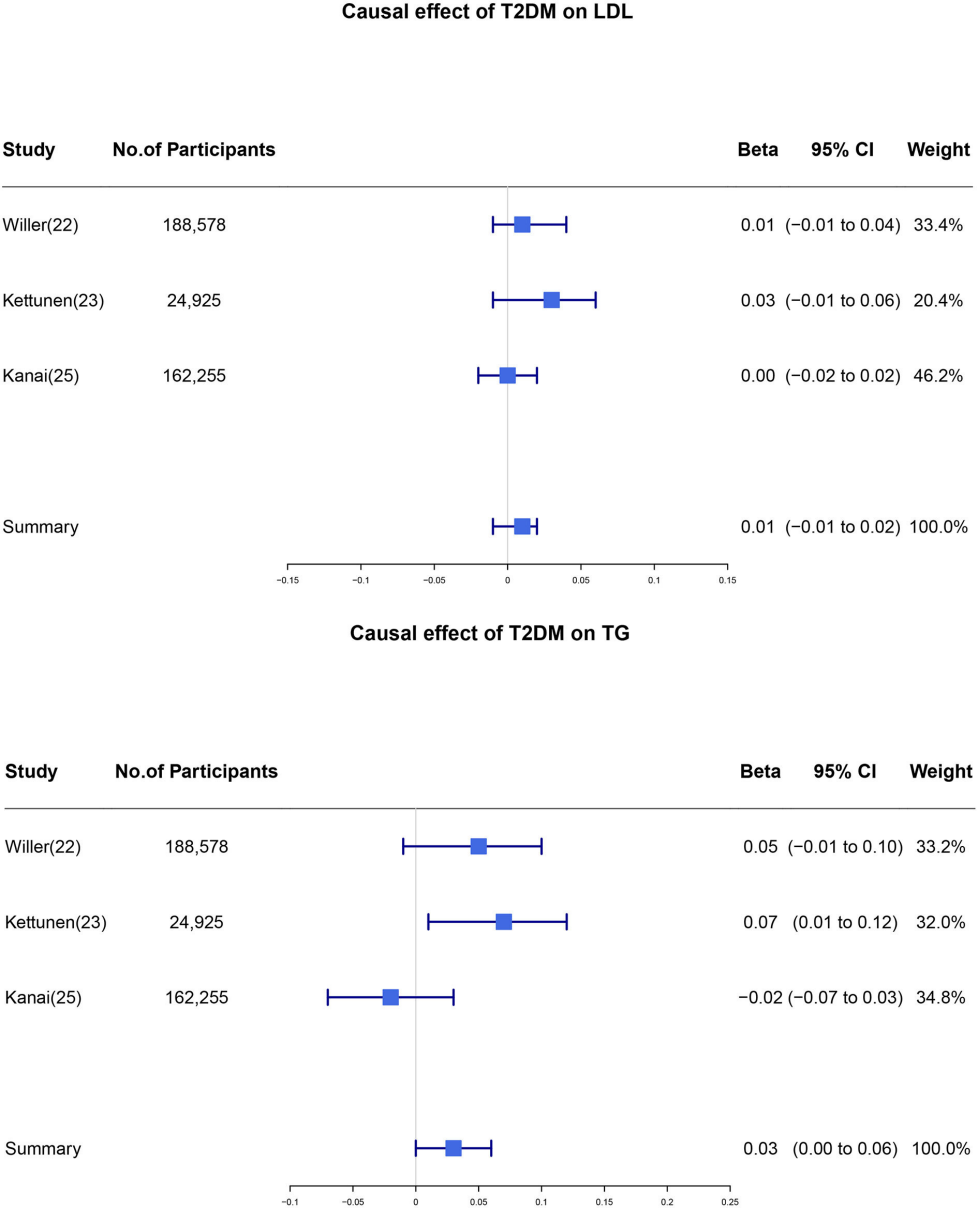
Individual and pooled estimate of the causal effect of T2DM on the level of LDL and TG after sensitivity analysis. We removed the study which generated heterogeneity in our meta-analysis and the individual and pooled estimates of the causal effect of T2DM on the level of LDL and TG were re-analyzed. T2DM, type 2 diabetes mellitus; LDL, low-density lipoprotein; TG, triglycerides; CI, confidence interval.

We noticed that the *I*^2^ statistics for pooled *β* in regard to LDL-C and TG were larger than 50% with a *p*-value <0.05. After performing the senstivity analysis, we identified that the source of heterogeneity was mainly from the UK Household Longitudinal Study. After removing the data from the UK Household Longitudinal Study, pooled *β* for LDL-C and TG were 0.01 (95% CI: −0.01 to 0.02, *I*^2^ = 0%, *p*-value = 0.44) and 0.03 (95% CI: 0.00 to 0.06, *I*^2^ = 66%, *p*-value = 0.06), respectively (Figure 3). Overall, our results showed that one unit higher log odds of T2DM was associated with lower level of HDL-C and higher level of TG. Pooled *β* did not support the causality of T2DM on TC and LDL-C levels.

### Estimate of the causal effect of T2DM on different sizes of HDL and LDL particles

**Figure 4:**
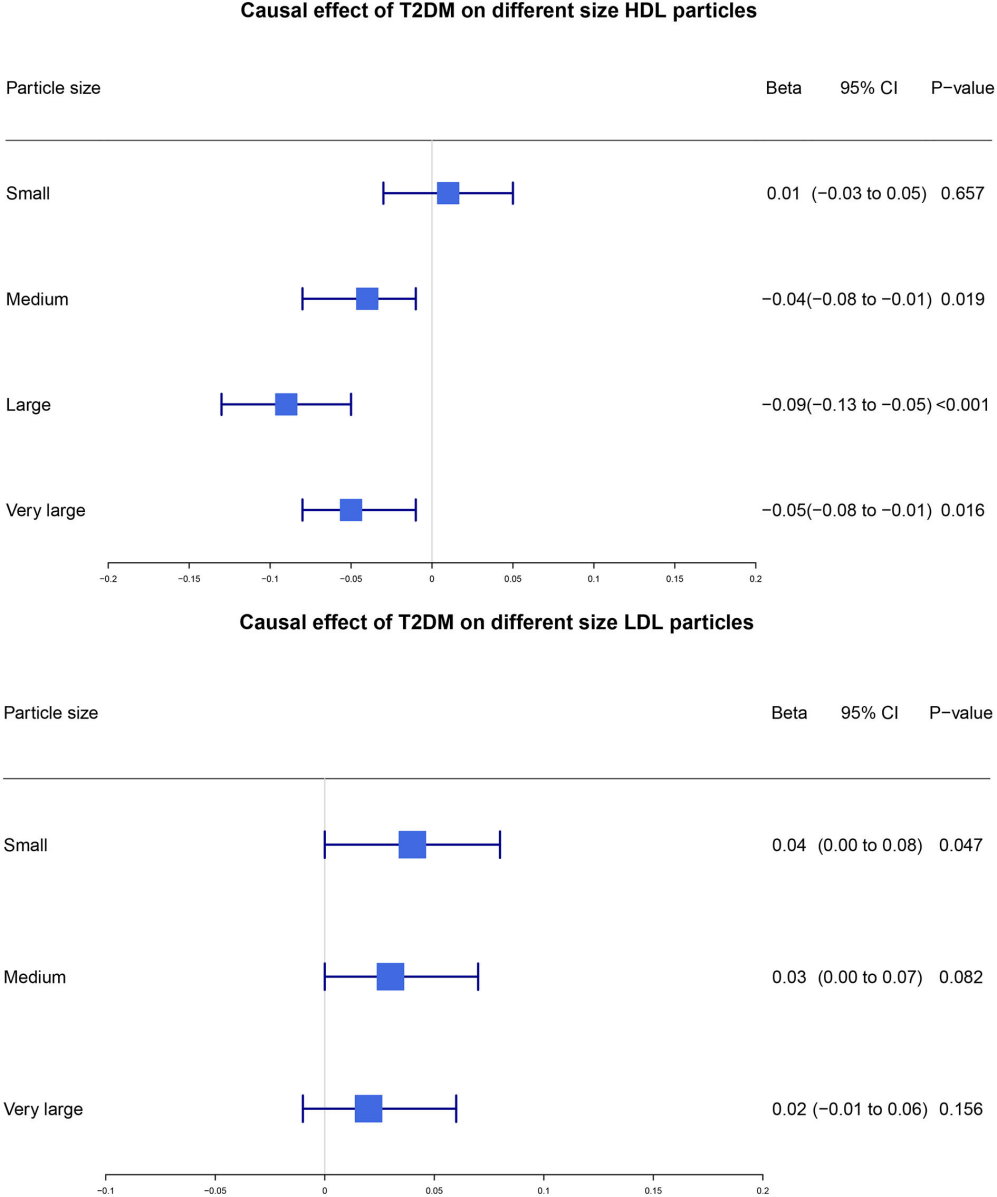
Estimate of the causal effect of T2DM on different sizes of HDL and LDL particles. We used the two-sample MR method to estimate the potential causal effect of T2DM on different sizes of HDL and LDL particles. T2DM, type 2 diabetes mellitus; HDL, high-density lipoprotein; LDL, low-density lipoprotein; CI, confidence interval.

Our study further categorized HDL particles and LDL particles into subgroups to investigate whether T2DM had different causal effects on the levels of different particle sizes. Results showed that T2DM had causal effects on the level of medium HDL particles (*β*: −0.04, 95% CI: −0.08 to −0.01, *p*-value = 0.019), large HDL particles (*β*: −0.09, 95% CI: −0.13 to −0.05, *p*-value < 0.001), and very large HDL particles (*β*: −0.05, 95% CI: −0.08 to −0.01, *p*-value = 0.016). In addition, the causality of T2DM on the level of small LDL particles was also found (*β*: 0.04, 95% CI: 0.00 to 0.08, *p*-value = 0.048). However, the causal effects of T2DM on the level of small HDL particles or large LDL or medium LDL-C particles were not found (*p*-value > 0.05) (Figure 4).

### MR sensitivity analyses

In MR sensitivity analyses, F-statistics of genetic variants for T2DM in our study was larger than 10 which indicated that genetic variants included in our study were strong enough to avoid weak instrument bias.

For the estimate of the causality of T2DM on the level of HDL-C (data source: Kettunen et.al), the MR-Egger intercept was non-zero with a *p*-value larger than 0.05, and the *p*-value for the distortion test of MR-PRESSO was larger than 0.05, which both indicated that the influence of horizontal pleiotropy was not statistically significant, and the difference between before and after removing outliers which caused horizontal pleiotropy was also not statistically significant either. In addition, the *p*-value for weighted median was <0.05 which was also consistent with the IVW method. Thus, we reckon that the estimate of the causal effect of T2DM on the level of HDL-C (data source: Kettunen) based on IVW results was robust.

Likewise, for the estimate of the causality of T2DM on the level of LDL-C (data source: Kanai, Kettunen, Willer), TC (data source: Kanai, Kettunen, Willer), TG (data source:Kanai, Willer), results of MR sensitivity analyses showed no horizontal pleiotropy, while for the estimate of the causality of T2DM on the level of LDL-C (data source: Prins), TC (data source: Prins), TG (data source: Kettunen, Prins), and MR sensitivity analyses indicated that the influence of horizontal pleiotropy on our results was not statistically significant either.

Results of single-SNP analyses and leave-one-out analyses were also consistent with our main analyses. The only exception was the estimate of the causality of T2DM on the level of HDL-C (data source: Prins), for the *p*-value of MR-Egger regression and distortion test in MR-PRESSO was both <0.05, thus we reckon that IVW results might be influenced by horizontal pleiotropy which we further discussed in the limitation part. Details of MR sensitivity analyses are shown in Supplementary Tables 24–69.

## Discussion

In the present study, we explored the causal effect of T2DM on blood lipid profiles and found that one additional unit of log odds of T2DM was causally associated with a 4% higher level of TG, 8% lower level of HDL-C, and also had significant causality on the concentration of different sizes of HDL and LDL lipoprotein particles. The above blood lipid profiles causally influenced by T2DM might be more specifically responsible for the excessive CVD risk among patients with T2DM, and new treatments may be developed to target these blood lipid alterations to further reduce secondary CVD risk among patients with T2DM.

As MR studies that explored the potential causal effects of T2DM on blood lipid profiles were lacking and RCTs were also scarce due to the difficulties in design and ethics, thus previous research which illustrated the relationship between T2DM and blood lipid profiles was mainly observational. Results of our study showed that T2DM had a positive causal effect on TG, and a negative causal effect on HDL-C, which were consistent with previous studies (11, 12, 15, 34–36). The causal relationship between T2DM and TC level was not found in our study which was also proved by the Framingham study (11), but there were also opposite results from comparative studies with 8-year follow-up claiming that TC levels of patients with T2DM were higher than non-diabetic participants (men: 5.84 ± 0.18 mmol/L, women: 5.84 ± 0.18 mmol/L vs. men: 5.55 ± 0.05 mmol/L, women: 5.20 ± 0.05 mmol/L, *p*-value = 0.002) (12). However, this difference may be due to the limited number of participants (17 males and 26 females). Most previous studies showed normal LDL-C levels among patients with T2DM (14, 36–38). A more recent study “PROCAM” also found little difference in the occurrence of high LDL levels between patients with T2DM and non-diabetic participants (22.7 vs. 21.7%) (15).

It is particularly noteworthy that the increasing number of studies in recent years illustrated that there were little differences in LDL-C levels between diabetic and non-diabetic population; however, the level of small dense LDL particles was found to increase instead (39–44) which were in accordance with our MR results. As the increasing number of small dense LDL is the consequence of lipolysis of increasing TG-rich VLDL particles (44), it is rational to observe both the increase of small LDL particles and TG levels in our study. Recent studies have shown that increasing levels of small LDL particles were closely associated with increased risk of CVD (45, 46) and a statement also suggested that cardiovascular disease risk may be more closely related to the number of atherogenic lipoprotein particles than to LDL cholesterol (47). Thus, our MR results which further indicated significant causal effects of genetically predicted T2DM on the subclass of lipoprotein particles could provide more robust evidence for better predictors of future CVD risks than the traditional lipid panel, especially in subjects with cardiometabolic diseases such as T2DM when traditional lipid panel is relatively normal. In addition, previous observational studies also showed that medium and large HDL particles were associated with T2DM (44, 48) which is consistent with the estimated *β* coefficient for genetically predicted medium, large, and very large HDL particles in our study. Due to the close relationship of atherogenic lipoprotein subclass particles with non-alcoholic fatty liver disease (NAFLD) and subsequent CVD events (49–51), our results may explain the excessive CVD risk among patients with T2DM.

Including data from the UK Household Longitudinal Study in meta-analyzing pooled *β* for TG and LDL-C could originate significant heterogeneity in our study. After comparing with other GWAS, we found patients taking statins and other lipid-lowering treatments were excluded except for the UK Household Longitudinal Study. Therefore, it might partly explain the lower estimated *β* coefficient for HDL-C and higher for TG using data from the UK Household Longitudinal Study, and lower estimated *β* coefficient for LDL-C might be due to statin intake.

### Strengths

First, to the best of our knowledge, the present study is the first MR study to identify the potential causality of T2DM on blood lipid profiles. MR studies can largely avoid bias from reverse causation, measurement error, and other unknown confoundings which are responsible for less conclusive results from observational studies. Besides, MR studies are less time-consuming, less expensive, easier to design, and with lifelong exposure compared with RCTs. Therefore, the uniqueness of MR studies makes them an effective method to explore the possible causal effect. Our MR study illustrated that T2DM had causal effects on certain types of blood lipid profiles which might provide help in exploring the complex mechanism of dyslipidemia in patients with T2DM.

Second, using MR-based statistics, we further conducted a meta-analysis to integrate different causal effects of T2DM on blood lipid profiles across distinct data resources to provide a more consolidated and desirable conclusion.

Third, the number of genetic variants used as proxies for each blood lipid profile in the present study is considerable and sensitivity analyses have confirmed the results of MR main analyses are less prone to be biased by weak instruments and less significant influence by horizontal pleiotropy, thus adding to the robustness of MR estimates. In addition, genetic variants for T2DM in the present study were identified from the latest GWAS which was larger in scale and also explained double heritability of T2DM than previous GWAS. Besides, data sources for outcomes were thoroughly searched and carefully checked for consistency, which further contributes to less heterogeneity and more reliability for final pooled MR estimates.

In addition, we further involved MR analysis for genetically predicted HDL and LDL into different subgroups according to the genetically determined size of lipoprotein particles. MR results showed the causal relationship between genetically predicted T2DM with a higher level of small LDL particles, and lower level of medium, large and very large HDL particles, which shed new light on the treatment targets to reduce the CVD risk of patients with T2DM despite the relatively normal level of LDL-C.

Last but not least, the present study design is different from normal MR studies that our individual estimates of the estimated *β* coefficient for each blood lipid profile were subsequently meta-analyzed to obtain a pooled estimated *β* coefficient from various data sources which made our results more persuasive.

### Limitations

There are several limitations of our study. First of all, the number of GWAS included for each blood lipid profile is not large enough and the sample size of some GWAS was also moderate. For example, the largest GWAS included in the present study recruited 1,88,578 participants, while the sample size of the UK Household Longitudinal Study was <10,000 and the sample size of another GWAS included was merely over 20,000. Second, although the number of genetic variants used in the present study was from the latest GWAS and was far more than previous studies, they could only explain a small proportion of T2DM heritability, leaving almost 90% unexplained. Besides, MR studies can avoid most biases affecting observational studies, but bias from horizontal pleiotropy should always be considered. Horizontal pleiotropy was generated when there were other pathways for the exposure to influence the outcome and the assumption of MR studies was violated, thus resulting in bias for MR main analysis. We have examined that most of our results were robust, while the estimate of the causal effect of T2DM on the level of HDL-C (data source: Prins) might be biased. In addition, restricted by the contradiction between the format of our data sources and the R software codes, we did not perform MR analyses that could detect correlated horizontal pleiotropy including Genome-wide mR Analysis under Pervasive PLEiotropy (GRAPPLE) (52) or Causal Analysis Using Summary Effect Estimates (CAUSE) (53). Future investigators are encouraged to apply more sensitivity analyses to detect both correlated and uncorrelated horizontal pleiotropy in order to minimize bias. Lastly, inspite of the large sample sizes of GWAS included in our study, the effect size of LDL-C and TC was rather small; thus, we could not completely rule out the causality of T2DM on LDL-C and TC. More large-scale GWAS and well-conducted observational studies are expected in future to provide more supportive evidence.

### Clinical and public implications

The causal effect of T2DM on LDL-C is still unclear and results from observational studies indicated an increase of small dense LDL-C in patients with T2DM. Results of our study were in accordance with previous studies and also found lower level of very large HDL, large HDL, and medium HDL, which might be indicative of the excessvie CVD risk of patients with T2DM. Besides, statin treatment for lowering LDL-C is widely recommended by various dyslipidemia guidelines for patients with T2DM. The 2016 ESC/EAS Guidelines for the Management of Dyslipidaemias regarded LDL-C as the primary target of lipid-lowering treatment among patients with T2DM (54). However, whether treatment for diabetic patients with higher TG and lower HDL-C is beneficial for CVD risk reduction remains controversial. These might be ascribable to the negative effects on reducing major cardiovascular events by using fenofibrate on patients with T2DM reported by FIELD and ACCORD studies (55, 56). The present study which was conducted in an MR framework indicated that patients with T2DM were more inclined to experience higher level of TG and lower level of HDL-C despite the relatively normal level of LDL-C. Moreover, our MR analysis also uncovered the causal effects of genetically predicted T2DM on genetically predicted atherogenic lipoprotein sub-particles. As the atherogenic lipoprotein sub-particles were closely related to cardiometabolic diseases such as NAFLD and increased subsequent risk of CVD, our results might provide new ideas for future RCTs to evaluate the impact of targeting the concentration of atherogenic lipoproteins sub-particles to attenuate CVD risk among patients with T2DM.

## Conclusion

Evidence from our study supported higher level of TG and lower level of HDL-C among patients with T2DM. Moreover, T2DM was also causally associated with higher level of small LDL particles, and lower level of medium, large, and very large HDL particles. New evidence from a genetic point of view for the causality of T2DM on these blood lipid profiles and lipoprotein subclass particles based on our study might be constructive for developing new therapeutic strategies to reduce the excessive CVD risk experienced by patients with T2DM.

## Data availability statement

The datasets presented in this study can be found in online repositories. The names of the repository/repositories and accession number(s) can be found in the article/Supplementary material.

## Author contributions

KC and JZ contributed equally to this manuscript, performed data analysis, and wrote the manuscript. KC and Y-DT designed this study, also took responsibility for the integrity, accuracy of data analysis in this study, and are the guarantors. CS, QZ, JY, TH, and Y-DT reviewed and revised the manuscript. All authors had access to data in this study and contributed to statistical analysis and reviewing of the manuscript. All listed authors meet authorship criteria. All authors contributed to the article and approved the submitted version.

## Funding

This work was supported by (1) National Key R&D Program of China (2020YFC2004700). (2) Research Unit of Medical Science Research Management/Basic and Clinical Research of Metabolic Cardiovascular Diseases from Chinese Academy of Medical Sciences (2021RU003). (3) National Natural Program (81825003). (4) Science and Technology Project of Xicheng District Finance (XCSTS-SD2021-01). (5) Basic research funds of the Central Public Welfare Research Institutes of the Chinese Academy of Medical Sciences (2018PT32032, 2018RC320014-2, and 3332019044).

## Conflict of interest

The authors declare that the research was conducted in the absence of any commercial or financial relationships that could be construed as a potential conflict of interest.

## Publisher's note

All claims expressed in this article are solely those of the authors and do not necessarily represent those of their affiliated organizations, or those of the publisher, the editors and the reviewers. Any product that may be evaluated in this article, or claim that may be made by its manufacturer, is not guaranteed or endorsed by the publisher.
