# Supplementary material for: Causal effects of genetically predicted type 2 diabetes mellitus on blood lipid profiles and concentration of particle-size-determined lipoprotein subclasses: A two-sample Mendelian randomization study
Source: Front Cardiovasc Med. 2022 Oct 13;9:965995. doi: 10.3389/fcvm.2022.965995 (PMC9606322; doi:10.3389/fcvm.2022.965995)
Supplement: Supplementary file 1 [file Table_1.DOCX]

目录

[Table 1. Characteristics of SNPs used as instrumental variables for T2DM 4](#_Toc102230233)

[Table 2. Genetic association estimates for the effect of T2DM on HDL (Prins). ea=effect allele, gx=T2DM, gy=HDL, se=standard error 9](#_Toc102230234)

[Table 3. Genetic association estimates for the effect of T2DM on HDL (Willer). ea=effect allele, gx=T2DM, gy=HDL, se=standard error 13](#_Toc102230235)

[Table 4. Genetic association estimates for the effect of T2DM on HDL (Kettunen). ea=effect allele, gx=T2DM, gy=HDL, se=standard error 17](#_Toc102230236)

[Table 5. Genetic association estimates for the effect of T2DM on LDL (Willer). ea=effect allele, gx=T2DM, gy=LDL, se=standard error 22](#_Toc102230237)

[Table 6. Genetic association estimates for the effect of T2DM on LDL (Prins). ea=effect allele, gx=T2DM, gy=LDL, se=standard error 27](#_Toc102230238)

[Table 7. Genetic association estimates for the effect of T2DM on LDL (Kanai). ea=effect allele, gx=T2DM, gy=LDL, se=standard error 31](#_Toc102230239)

[Table 8. Genetic association estimates for the effect of T2DM on LDL (Kettunen). ea=effect allele, gx=T2DM, gy=LDL, se=standard error 35](#_Toc102230240)

[Table 9. Genetic association estimates for the effect of T2DM on TC (Willer). ea=effect allele, gx=T2DM, gy=TC, se=standard error 40](#_Toc102230241)

[Table 10. Genetic association estimates for the effect of T2DM on TC (Kettunen). ea=effect allele, gx=T2DM, gy=TC, se=standard error 44](#_Toc102230242)

[Table 11. Genetic association estimates for the effect of T2DM on TC (Prins). ea=effect allele, gx=T2DM, gy=TC, se=standard error 49](#_Toc102230243)

[Table 12. Genetic association estimates for the effect of T2DM on TC (Kanai). ea=effect allele, gx=T2DM, gy=TC, se=standard error 53](#_Toc102230244)

[Table 13. Genetic association estimates for the effect of T2DM on TG (Willer). ea=effect allele, gx=T2DM, gy=TG, se=standard error 57](#_Toc102230245)

[Table 14. Genetic association estimates for the effect of T2DM on TG (Prins). ea=effect allele, gx=T2DM, gy=TG, se=standard error 62](#_Toc102230246)

[Table 15. Genetic association estimates for the effect of T2DM on TG (Kettunen). ea=effect allele, gx=T2DM, gy=TG, se=standard error 66](#_Toc102230247)

[Table 16. Genetic association estimates for the effect of T2DM on TG (Kanai). ea=effect allele, gx=T2DM, gy=TG, se=standard error 71](#_Toc102230248)

[Table 17. Genetic association estimates for the effect of T2DM on small HDL particle. ea=effect allele, gx=T2DM, gy= small HDL particle, se=standard error 75](#_Toc102230249)

[Table 18. Genetic association estimates for the effect of T2DM on medium HDL particle. ea=effect allele, gx=T2DM, gy= medium HDL particle, se=standard error 80](#_Toc102230250)

[Table 19. Genetic association estimates for the effect of T2DM on large HDL particle. ea=effect allele, gx=T2DM, gy= large HDL particle, se=standard error 85](#_Toc102230251)

[Table 20. Genetic association estimates for the effect of T2DM on very large HDL particle. ea=effect allele, gx=T2DM, gy= very large HDL particle, se=standard error 90](#_Toc102230252)

[Table 21. Genetic association estimates for the effect of T2DM on small LDL particle. ea=effect allele, gx=T2DM, gy= small LDL particle, se=standard error 94](#_Toc102230253)

[Table 22. Genetic association estimates for the effect of T2DM on medium LDL particle. ea=effect allele, gx=T2DM, gy= medium LDL particle, se=standard error 99](#_Toc102230254)

[Table 23. Genetic association estimates for the effect of T2DM on large LDL particle. ea=effect allele, gx=T2DM, gy= large LDL particle, se=standard error 104](#_Toc102230255)

[Table 24: MR-PRESSO for estimate of T2DM-HDL(Prins). 109](#_Toc102230256)

[Table 25: MR-PRESSO for estimate of T2DM-HDL(Willer). 109](#_Toc102230257)

[Table 26: MR-PRESSO for estimate of T2DM-HDL(Kettunen). 109](#_Toc102230258)

[Table 27: MR-PRESSO for estimate of T2DM-LDL(Willer). 110](#_Toc102230259)

[Table 28: MR-PRESSO for estimate of T2DM-LDL(Prins). 110](#_Toc102230260)

[Table 29: MR-PRESSO for estimate of T2DM-LDL(Kanai). 111](#_Toc102230261)

[Table 30: MR-PRESSO for estimate of T2DM-LDL(Kettunen). 111](#_Toc102230262)

[Table 31: MR-PRESSO for estimate of T2DM-TC(Willer). 111](#_Toc102230263)

[Table 32: MR-PRESSO for estimate of T2DM-TC(Kettunen). 112](#_Toc102230264)

[Table 33: MR-PRESSO for estimate of T2DM-TC(Prins). 112](#_Toc102230265)

[Table 34: MR-PRESSO for estimate of T2DM-TC(Kanai). 113](#_Toc102230266)

[Table 35: MR-PRESSO for estimate of T2DM-TG(Willer). 113](#_Toc102230267)

[Table 36: MR-PRESSO for estimate of T2DM-TG(Prins). 113](#_Toc102230268)

[Table 37: MR-PRESSO for estimate of T2DM-TG(Kettunen). 114](#_Toc102230269)

[Table 38: MR-PRESSO for estimate of T2DM-TG(Kanai). 114](#_Toc102230270)

[Table 39: Single SNP analysis for estimate of T2DM-HDL(Prins). 115](#_Toc102230271)

[Table 40: Leave-one-out analysis for estimate of T2DM-HDL(Prins). 119](#_Toc102230272)

[Table 41: Single SNP analysis for estimate of T2DM-HDL(Willer). 123](#_Toc102230273)

[Table 42: Leave-one-out analysis for estimate of T2DM-HDL(Willer). 128](#_Toc102230274)

[Table 43: Single SNP analysis for estimate of T2DM-HDL(Kettunen). 132](#_Toc102230275)

[Table 44: Leave-one-out analysis for estimate of T2DM-HDL(Kettunen). 137](#_Toc102230276)

[Table 45: Single SNP analysis for estimate of T2DM-LDL(Willer). 142](#_Toc102230277)

[Table 46: Leave-one-out analysis for estimate of T2DM-LDL(Willer). 146](#_Toc102230278)

[Table 47: Single SNP analysis for estimate of T2DM-LDL(Prins). 151](#_Toc102230279)

[Table 48: Leave-one-out analysis for estimate of T2DM-LDL(Prins). 155](#_Toc102230280)

[Table 49: Single SNP analysis for estimate of T2DM-LDL(Kanai). 159](#_Toc102230281)

[Table 50: Leave-one-out analysis for estimate of T2DM-LDL(Kanai). 164](#_Toc102230282)

[Table 51: Single SNP analysis for estimate of T2DM-LDL(Kettunen). 168](#_Toc102230283)

[Table 52: Leave-one-out analysis for estimate of T2DM-LDL(Kettunen). 173](#_Toc102230284)

[Table 53: Single SNP analysis for estimate of T2DM-TC(Willer). 178](#_Toc102230285)

[Table 54: Leave-one-out analysis for estimate of T2DM-TC(Willer). 182](#_Toc102230286)

[Table 55: Single SNP analysis for estimate of T2DM- TC(Kettunen). 187](#_Toc102230287)

[Table 56: Leave-one-out analysis for estimate of T2DM- TC(Kettunen). 192](#_Toc102230288)

[Table 57: Single SNP analysis for estimate of T2DM- TC(Prins). 196](#_Toc102230289)

[Table 58: Leave-one-out analysis for estimate of T2DM- TC(Prins). 200](#_Toc102230290)

[Table 59: Single SNP analysis for estimate of T2DM-TC(Kanai). 204](#_Toc102230291)

[Table 60: Leave-one-out analysis for estimate of T2DM-TC(Kanai). 209](#_Toc102230292)

[Table 61: Single SNP analysis for estimate of T2DM-TG(Willer). 213](#_Toc102230293)

[Table 62: Leave-one-out analysis for estimate of T2DM-TG(Willer). 218](#_Toc102230294)

[Table 63: Single SNP analysis for estimate of T2DM-TG(Prins). 222](#_Toc102230295)

[Table 64: Leave-one-out analysis for estimate of T2DM-TG(Prins). 227](#_Toc102230296)

[Table 65: Single SNP analysis for estimate of T2DM-TG(Kettunen). 231](#_Toc102230297)

[Table 66: Leave-one-out analysis for estimate of T2DM-TG(Kettunen). 236](#_Toc102230298)

[Table 67: Single SNP analysis for estimate of T2DM-TG(Kanai). 241](#_Toc102230299)

[Table 68: Leave-one-out analysis for estimate of T2DM-TG(Kanai). 245](#_Toc102230300)

[Table 69. Weighted Median, MR-Egger regression results for estimate of causal effect of T2DM on blood lipid profiles. 249](#_Toc102230301)

## Table 1. Characteristics of SNPs used as instrumental variables for T2DM

|  | SNP | effect_allele | other_allele | EAF.exposure | effect | SE | p-value | F-statistic |
| --- | --- | --- | --- | --- | --- | --- | --- | --- |
| 1 | rs1127655 | T | C | 0.53 | -0.04 | 0.01 | 2.47E-08 | 30.74 |
| 2 | rs2296173 | G | A | 0.21 | 0.07 | 0.01 | 7.66E-14 | 55.82 |
| 3 | rs12088739 | G | A | 0.09 | -0.09 | 0.01 | 9.79E-12 | 46.24 |
| 4 | rs340874 | C | T | 0.56 | 0.06 | 0.01 | 8.41E-18 | 73.54 |
| 5 | rs2820426 | G | A | 0.61 | 0.05 | 0.01 | 1.30E-12 | 50.94 |
| 6 | rs2493394 | G | A | 0.11 | 0.07 | 0.01 | 1.15E-10 | 41.73 |
| 7 | rs348330 | A | G | 0.63 | -0.05 | 0.01 | 1.86E-09 | 36.15 |
| 8 | rs2867125 | C | T | 0.83 | 0.06 | 0.01 | 4.33E-10 | 39.19 |
| 9 | rs17334919 | T | C | 0.10 | -0.14 | 0.01 | 6.69E-28 | 119.29 |
| 10 | rs13389219 | T | C | 0.39 | -0.07 | 0.01 | 2.11E-22 | 95.19 |
| 11 | rs2972144 | G | A | 0.65 | 0.09 | 0.01 | 2.55E-34 | 148.19 |
| 12 | rs840967 | A | C | 0.61 | -0.05 | 0.01 | 5.44E-10 | 38.60 |
| 13 | rs243019 | C | T | 0.46 | 0.06 | 0.01 | 2.29E-15 | 63.55 |
| 14 | rs7561798 | G | A | 0.48 | 0.04 | 0.01 | 2.79E-08 | 30.86 |
| 15 | rs780094 | C | T | 0.61 | 0.07 | 0.01 | 5.16E-21 | 87.45 |
| 16 | rs12617659 | T | C | 0.15 | -0.07 | 0.01 | 2.83E-11 | 44.23 |
| 17 | rs7572970 | G | A | 0.72 | 0.06 | 0.01 | 1.39E-11 | 45.99 |
| 18 | rs4686471 | C | T | 0.61 | 0.05 | 0.01 | 4.28E-11 | 43.46 |
| 19 | rs6795735 | T | C | 0.41 | -0.06 | 0.01 | 1.63E-14 | 58.43 |
| 20 | rs9844972 | C | G | 0.07 | 0.10 | 0.01 | 1.03E-10 | 41.72 |
| 21 | rs1899951 | T | C | 0.12 | -0.11 | 0.01 | 1.64E-24 | 105.20 |
| 22 | rs11708067 | G | A | 0.24 | -0.10 | 0.01 | 5.93E-29 | 125.91 |
| 23 | rs11926707 | C | T | 0.63 | 0.05 | 0.01 | 1.69E-08 | 31.88 |
| 24 | rs2292662 | T | C | 0.15 | -0.06 | 0.01 | 1.24E-08 | 32.11 |
| 25 | rs7619041 | A | T | 0.51 | -0.04 | 0.01 | 2.76E-08 | 30.53 |
| 26 | rs7651090 | G | A | 0.31 | 0.12 | 0.01 | 3.85E-57 | 250.97 |
| 27 | rs1496653 | G | A | 0.20 | -0.08 | 0.01 | 2.57E-18 | 76.36 |
| 28 | rs11925227 | A | G | 0.18 | -0.05 | 0.01 | 2.25E-08 | 31.60 |
| 29 | rs1801214 | T | C | 0.60 | 0.09 | 0.01 | 5.52E-34 | 148.91 |
| 30 | rs11098676 | C | T | 0.79 | 0.05 | 0.01 | 2.03E-08 | 31.64 |
| 31 | rs17086692 | T | G | 0.31 | -0.05 | 0.01 | 2.48E-08 | 30.91 |
| 32 | rs7674212 | T | G | 0.41 | -0.05 | 0.01 | 6.18E-10 | 38.44 |
| 33 | rs7685296 | T | C | 0.28 | -0.05 | 0.01 | 2.32E-10 | 39.80 |
| 34 | rs993380 | G | A | 0.67 | -0.05 | 0.01 | 4.59E-10 | 39.18 |
| 35 | rs735949 | C | T | 0.14 | -0.07 | 0.01 | 1.95E-11 | 44.99 |
| 36 | rs1061813 | A | G | 0.54 | -0.04 | 0.01 | 3.37E-09 | 34.54 |
| 37 | rs4865796 | A | G | 0.69 | 0.05 | 0.01 | 1.33E-11 | 46.17 |
| 38 | rs459193 | G | A | 0.75 | 0.07 | 0.01 | 8.81E-18 | 73.38 |
| 39 | rs6878122 | A | G | 0.68 | -0.06 | 0.01 | 1.19E-12 | 50.97 |
| 40 | rs10077431 | A | C | 0.21 | -0.05 | 0.01 | 4.75E-08 | 29.94 |
| 41 | rs7729395 | T | C | 0.05 | 0.14 | 0.02 | 1.10E-17 | 73.64 |
| 42 | rs2246618 | T | C | 0.31 | 0.05 | 0.01 | 1.20E-09 | 37.30 |
| 43 | rs72892910 | T | G | 0.17 | 0.06 | 0.01 | 6.43E-11 | 42.84 |
| 44 | rs3756784 | G | T | 0.19 | 0.05 | 0.01 | 2.59E-08 | 30.80 |
| 45 | rs1050226 | G | A | 0.41 | -0.05 | 0.01 | 3.34E-11 | 44.03 |
| 46 | rs7756992 | G | A | 0.27 | 0.13 | 0.01 | 6.00E-62 | 276.50 |
| 47 | rs9369425 | A | G | 0.71 | -0.05 | 0.01 | 1.13E-10 | 41.26 |
| 48 | rs1063355 | G | T | 0.60 | 0.07 | 0.01 | 3.72E-19 | 80.54 |
| 49 | rs853974 | C | T | 0.74 | -0.06 | 0.01 | 7.86E-12 | 46.64 |
| 50 | rs622217 | C | T | 0.48 | -0.05 | 0.01 | 3.13E-10 | 39.67 |
| 51 | rs2191348 | T | G | 0.55 | 0.07 | 0.01 | 3.44E-19 | 79.77 |
| 52 | rs17168486 | T | C | 0.17 | 0.07 | 0.01 | 2.18E-15 | 62.31 |
| 53 | rs849135 | A | G | 0.50 | -0.10 | 0.01 | 1.04E-43 | 192.52 |
| 54 | rs2908282 | A | G | 0.18 | 0.06 | 0.01 | 4.25E-09 | 34.48 |
| 55 | rs2299383 | T | C | 0.42 | 0.04 | 0.01 | 1.49E-08 | 31.85 |
| 56 | rs13234269 | A | T | 0.49 | -0.06 | 0.01 | 6.98E-14 | 55.87 |
| 57 | rs7786095 | G | A | 0.10 | -0.07 | 0.01 | 9.64E-09 | 33.17 |
| 58 | rs13239186 | T | C | 0.30 | 0.05 | 0.01 | 2.70E-10 | 40.21 |
| 59 | rs10087241 | A | G | 0.59 | -0.05 | 0.01 | 2.80E-09 | 35.25 |
| 60 | rs7845219 | C | T | 0.49 | -0.04 | 0.01 | 4.54E-09 | 34.35 |
| 61 | rs516946 | C | T | 0.76 | 0.08 | 0.01 | 3.16E-22 | 93.98 |
| 62 | rs3802177 | A | G | 0.31 | -0.12 | 0.01 | 2.32E-52 | 231.42 |
| 63 | rs10100265 | C | A | 0.61 | -0.05 | 0.01 | 6.29E-10 | 38.63 |
| 64 | rs17411031 | G | C | 0.26 | -0.05 | 0.01 | 3.04E-08 | 30.86 |
| 65 | rs2294120 | G | A | 0.46 | -0.04 | 0.01 | 1.62E-08 | 31.45 |
| 66 | rs10974438 | C | A | 0.35 | 0.06 | 0.01 | 3.01E-15 | 62.09 |
| 67 | rs10114341 | C | T | 0.44 | -0.04 | 0.01 | 1.15E-08 | 32.27 |
| 68 | rs17791483 | G | A | 0.06 | -0.10 | 0.01 | 3.42E-12 | 48.15 |
| 69 | rs1758632 | G | C | 0.62 | 0.05 | 0.01 | 1.36E-09 | 36.74 |
| 70 | rs10811661 | C | T | 0.17 | -0.16 | 0.01 | 4.13E-58 | 256.33 |
| 71 | rs1333039 | C | G | 0.60 | 0.05 | 0.01 | 5.64E-13 | 52.07 |
| 72 | rs2796441 | A | G | 0.42 | -0.07 | 0.01 | 1.96E-22 | 95.93 |
| 73 | rs10740322 | A | G | 0.69 | 0.05 | 0.01 | 2.11E-08 | 31.49 |
| 74 | rs11257655 | T | C | 0.21 | 0.07 | 0.01 | 1.97E-17 | 71.76 |
| 75 | rs753270 | C | T | 0.58 | 0.05 | 0.01 | 2.70E-11 | 44.67 |
| 76 | rs7923866 | T | C | 0.38 | -0.10 | 0.01 | 9.34E-40 | 172.53 |
| 77 | rs4506565 | T | A | 0.31 | 0.28 | 0.01 | 1.00E-200 | 1389.80 |
| 78 | rs1552224 | C | A | 0.15 | -0.10 | 0.01 | 8.64E-25 | 104.81 |
| 79 | rs67232546 | T | C | 0.21 | 0.06 | 0.01 | 4.66E-10 | 38.54 |
| 80 | rs10830963 | G | C | 0.28 | 0.09 | 0.01 | 5.85E-30 | 129.11 |
| 81 | rs2237892 | T | C | 0.06 | -0.10 | 0.02 | 8.75E-10 | 37.39 |
| 82 | rs5215 | T | C | 0.64 | -0.07 | 0.01 | 2.09E-20 | 86.26 |
| 83 | rs7929543 | C | A | 0.08 | 0.08 | 0.01 | 2.20E-09 | 36.00 |
| 84 | rs2261181 | T | C | 0.10 | 0.10 | 0.01 | 9.18E-17 | 69.68 |
| 85 | rs7138300 | T | C | 0.56 | -0.04 | 0.01 | 5.65E-10 | 37.86 |
| 86 | rs12299509 | G | A | 0.48 | 0.05 | 0.01 | 2.09E-10 | 40.92 |
| 87 | rs11107116 | T | G | 0.22 | 0.05 | 0.01 | 3.75E-08 | 30.19 |
| 88 | rs10842994 | T | C | 0.20 | -0.08 | 0.01 | 1.02E-16 | 68.84 |
| 89 | rs61953351 | T | G | 0.25 | -0.07 | 0.01 | 1.98E-14 | 59.17 |
| 90 | rs825476 | T | C | 0.58 | 0.05 | 0.01 | 6.80E-13 | 51.52 |
| 91 | rs576674 | A | G | 0.83 | -0.07 | 0.01 | 1.79E-11 | 45.46 |
| 92 | rs963740 | T | A | 0.29 | -0.05 | 0.01 | 2.23E-08 | 31.02 |
| 93 | rs1359790 | A | G | 0.29 | -0.08 | 0.01 | 2.80E-23 | 99.00 |
| 94 | rs7144011 | T | G | 0.22 | 0.05 | 0.01 | 1.64E-08 | 32.16 |
| 95 | rs7177055 | A | G | 0.72 | 0.06 | 0.01 | 2.75E-16 | 67.07 |
| 96 | rs6494307 | G | C | 0.43 | -0.04 | 0.01 | 1.67E-08 | 32.26 |
| 97 | rs982077 | G | A | 0.57 | -0.05 | 0.01 | 2.58E-10 | 39.59 |
| 98 | rs12910825 | G | A | 0.36 | 0.05 | 0.01 | 2.16E-12 | 48.81 |
| 99 | rs9940149 | A | G | 0.18 | -0.06 | 0.01 | 9.29E-10 | 37.27 |
| 100 | rs13330951 | G | A | 0.49 | -0.05 | 0.01 | 1.54E-08 | 31.69 |
| 101 | rs77258096 | A | C | 0.10 | -0.12 | 0.01 | 1.78E-18 | 76.37 |
| 102 | rs2925979 | C | T | 0.70 | -0.05 | 0.01 | 9.06E-12 | 46.87 |
| 103 | rs7185735 | G | A | 0.40 | 0.11 | 0.01 | 1.59E-47 | 209.26 |
| 104 | rs17405722 | A | G | 0.07 | 0.09 | 0.01 | 2.28E-09 | 35.51 |
| 105 | rs8068804 | A | G | 0.33 | 0.06 | 0.01 | 4.41E-14 | 56.64 |
| 106 | rs12945601 | C | T | 0.61 | -0.05 | 0.01 | 1.72E-09 | 36.00 |
| 107 | rs9894220 | G | A | 0.43 | -0.06 | 0.01 | 1.52E-13 | 54.83 |
| 108 | rs17631783 | T | C | 0.26 | -0.05 | 0.01 | 3.95E-08 | 29.94 |
| 109 | rs7240767 | C | T | 0.38 | 0.05 | 0.01 | 2.16E-08 | 31.00 |
| 110 | rs12970134 | A | G | 0.27 | 0.06 | 0.01 | 5.31E-12 | 48.13 |
| 111 | rs10401969 | C | T | 0.08 | 0.09 | 0.01 | 4.13E-12 | 47.95 |
| 112 | rs8108269 | G | T | 0.28 | 0.06 | 0.01 | 3.11E-16 | 66.45 |
| 113 | rs6515236 | C | A | 0.25 | -0.05 | 0.01 | 3.34E-08 | 30.67 |
| 114 | rs6066138 | A | G | 0.28 | -0.05 | 0.01 | 1.93E-09 | 35.71 |
| 115 | rs6059662 | G | A | 0.66 | 0.04 | 0.01 | 1.51E-08 | 31.87 |
| 116 | rs4810426 | T | C | 0.10 | 0.07 | 0.01 | 2.15E-08 | 31.19 |
| 117 | rs16988333 | G | A | 0.09 | -0.07 | 0.01 | 9.17E-09 | 32.84 |
| 118 | rs4823182 | G | A | 0.34 | 0.05 | 0.01 | 3.36E-10 | 39.18 |

EAF: Effect allele frequency; T2DM: Type-2 diabetes.

Table 2. Genetic association estimates for the effect of T2DM on HDL (Prins). ea=effect allele, gx=T2DM, gy=HDL, se=standard error

|  | SNP | ea | gx | gx_se | gy | gy_se |
| --- | --- | --- | --- | --- | --- | --- |
| 1 | rs10077431 | A | -0.05 | 0.01 | 0.01 | 0.02 |
| 2 | rs10100265 | C | -0.05 | 0.01 | -0.01 | 0.02 |
| 3 | rs10114341 | C | -0.04 | 0.01 | 0.02 | 0.01 |
| 4 | rs10401969 | C | 0.09 | 0.01 | 0.04 | 0.03 |
| 5 | rs1050226 | G | -0.05 | 0.01 | 0.04 | 0.02 |
| 6 | rs1061813 | A | -0.04 | 0.01 | 0.02 | 0.01 |
| 7 | rs1063355 | G | 0.07 | 0.01 | 0.01 | 0.02 |
| 8 | rs10740322 | A | 0.05 | 0.01 | 0.01 | 0.02 |
| 9 | rs10811661 | C | -0.16 | 0.01 | 0.02 | 0.02 |
| 10 | rs10974438 | C | 0.06 | 0.01 | -0.01 | 0.02 |
| 11 | rs11098676 | C | 0.05 | 0.01 | -0.03 | 0.02 |
| 12 | rs11107116 | T | 0.05 | 0.01 | -0.02 | 0.02 |
| 13 | rs11257655 | T | 0.07 | 0.01 | 0.01 | 0.02 |
| 14 | rs1127655 | T | -0.04 | 0.01 | 0.02 | 0.02 |
| 15 | rs11708067 | G | -0.10 | 0.01 | -0.01 | 0.02 |
| 16 | rs11925227 | A | -0.05 | 0.01 | 0.01 | 0.02 |
| 17 | rs12088739 | G | -0.09 | 0.01 | 0.08 | 0.03 |
| 18 | rs12299509 | G | 0.05 | 0.01 | 0.03 | 0.02 |
| 19 | rs12617659 | T | -0.07 | 0.01 | 0.01 | 0.02 |
| 20 | rs12910825 | G | 0.05 | 0.01 | 0.01 | 0.02 |
| 21 | rs12945601 | C | -0.05 | 0.01 | 0.02 | 0.02 |
| 22 | rs12970134 | A | 0.06 | 0.01 | -0.02 | 0.02 |
| 23 | rs13234269 | A | -0.06 | 0.01 | 0.03 | 0.01 |
| 24 | rs13330951 | G | -0.05 | 0.01 | 0.01 | 0.02 |
| 25 | rs13389219 | T | -0.07 | 0.01 | 0.02 | 0.02 |
| 26 | rs1496653 | G | -0.08 | 0.01 | 0.02 | 0.02 |
| 27 | rs1552224 | C | -0.10 | 0.01 | 0.01 | 0.02 |
| 28 | rs16988333 | G | -0.07 | 0.01 | 0.01 | 0.03 |
| 29 | rs17086692 | T | -0.05 | 0.01 | 0.11 | 0.06 |
| 30 | rs17168486 | T | 0.07 | 0.01 | -0.03 | 0.02 |
| 31 | rs17405722 | A | 0.09 | 0.01 | -0.03 | 0.03 |
| 32 | rs17411031 | G | -0.05 | 0.01 | 0.10 | 0.02 |
| 33 | rs1758632 | G | 0.05 | 0.01 | -0.04 | 0.02 |
| 34 | rs17791513 | G | -0.10 | 0.01 | 0.01 | 0.03 |
| 35 | rs1801214 | T | 0.09 | 0.01 | -0.01 | 0.02 |
| 36 | rs1899951 | T | -0.11 | 0.01 | 0.03 | 0.02 |
| 37 | rs2058913 | T | -0.05 | 0.01 | 0.01 | 0.02 |
| 38 | rs2237892 | T | -0.10 | 0.02 | 0.05 | 0.03 |
| 39 | rs2246618 | T | 0.05 | 0.01 | -0.01 | 0.02 |
| 40 | rs2261181 | T | 0.10 | 0.01 | 0.01 | 0.02 |
| 41 | rs2294120 | G | -0.04 | 0.01 | -0.01 | 0.02 |
| 42 | rs2296173 | G | 0.07 | 0.01 | -0.03 | 0.02 |
| 43 | rs2299383 | T | 0.04 | 0.01 | -0.02 | 0.01 |
| 44 | rs243019 | C | 0.06 | 0.01 | 0.01 | 0.02 |
| 45 | rs2493394 | G | 0.07 | 0.01 | -0.03 | 0.02 |
| 46 | rs2820426 | G | 0.05 | 0.01 | -0.03 | 0.02 |
| 47 | rs2867125 | C | 0.06 | 0.01 | -0.02 | 0.02 |
| 48 | rs2908282 | A | 0.06 | 0.01 | 0.04 | 0.02 |
| 49 | rs2925979 | C | -0.05 | 0.01 | 0.04 | 0.02 |
| 50 | rs2943656 | G | 0.09 | 0.01 | -0.03 | 0.02 |
| 51 | rs3217992 | T | 0.05 | 0.01 | -0.01 | 0.02 |
| 52 | rs3756784 | G | 0.05 | 0.01 | -0.03 | 0.02 |
| 53 | rs3802177 | A | -0.12 | 0.01 | 0.01 | 0.02 |
| 54 | rs459193 | G | 0.07 | 0.01 | -0.02 | 0.02 |
| 55 | rs4622883 | G | -0.04 | 0.01 | 0.02 | 0.01 |
| 56 | rs4686471 | C | 0.05 | 0.01 | -0.01 | 0.02 |
| 57 | rs4812829 | A | 0.05 | 0.01 | 0.01 | 0.02 |
| 58 | rs4823182 | G | 0.05 | 0.01 | -0.03 | 0.02 |
| 59 | rs4865796 | A | 0.05 | 0.01 | -0.01 | 0.02 |
| 60 | rs516946 | C | 0.08 | 0.01 | -0.02 | 0.02 |
| 61 | rs5215 | T | -0.07 | 0.01 | -0.01 | 0.02 |
| 62 | rs55966194 | G | -0.05 | 0.01 | -0.01 | 0.02 |
| 63 | rs576674 | A | -0.07 | 0.01 | -0.02 | 0.02 |
| 64 | rs6059662 | G | 0.04 | 0.01 | 0.01 | 0.02 |
| 65 | rs61953351 | T | -0.07 | 0.01 | -0.01 | 0.02 |
| 66 | rs622217 | C | -0.05 | 0.01 | 0.01 | 0.02 |
| 67 | rs6494307 | G | -0.04 | 0.01 | -0.01 | 0.02 |
| 68 | rs6515236 | C | -0.05 | 0.01 | -0.01 | 0.02 |
| 69 | rs67232546 | T | 0.06 | 0.01 | 0.02 | 0.02 |
| 70 | rs6767484 | G | 0.12 | 0.01 | -0.04 | 0.02 |
| 71 | rs6785040 | C | -0.06 | 0.01 | 0.01 | 0.02 |
| 72 | rs6878122 | A | -0.06 | 0.01 | -0.01 | 0.02 |
| 73 | rs6960043 | C | 0.06 | 0.01 | -0.01 | 0.02 |
| 74 | rs7240767 | C | 0.05 | 0.01 | -0.01 | 0.01 |
| 75 | rs72802358 | C | -0.12 | 0.01 | 0.01 | 0.02 |
| 76 | rs72892910 | T | 0.06 | 0.01 | 0.02 | 0.02 |
| 77 | rs735949 | C | -0.07 | 0.01 | -0.01 | 0.02 |
| 78 | rs753270 | C | 0.05 | 0.01 | -0.01 | 0.02 |
| 79 | rs7561798 | G | 0.04 | 0.01 | 0.02 | 0.01 |
| 80 | rs7572970 | G | 0.06 | 0.01 | -0.01 | 0.02 |
| 81 | rs7607777 | T | -0.14 | 0.01 | -0.03 | 0.02 |
| 82 | rs7674212 | T | -0.05 | 0.01 | 0.01 | 0.02 |
| 83 | rs7685296 | T | -0.05 | 0.01 | 0.03 | 0.02 |
| 84 | rs7729395 | T | 0.14 | 0.02 | -0.06 | 0.03 |
| 85 | rs7756992 | G | 0.13 | 0.01 | -0.01 | 0.02 |
| 86 | rs7786095 | G | -0.07 | 0.01 | 0.01 | 0.02 |
| 87 | rs780094 | C | 0.07 | 0.01 | 0.01 | 0.02 |
| 88 | rs7845219 | C | -0.04 | 0.01 | 0.01 | 0.02 |
| 89 | rs7903146 | T | 0.31 | 0.01 | -0.01 | 0.02 |
| 90 | rs7929543 | C | 0.08 | 0.01 | 0.01 | 0.03 |
| 91 | rs7955901 | T | -0.04 | 0.01 | 0.03 | 0.02 |
| 92 | rs8068804 | A | 0.06 | 0.01 | -0.01 | 0.02 |
| 93 | rs8108269 | G | 0.06 | 0.01 | -0.02 | 0.02 |
| 94 | rs825476 | T | 0.05 | 0.01 | -0.01 | 0.01 |
| 95 | rs853974 | C | -0.06 | 0.01 | 0.01 | 0.02 |
| 96 | rs9369425 | A | -0.05 | 0.01 | 0.02 | 0.02 |
| 97 | rs9844972 | C | 0.10 | 0.01 | -0.03 | 0.03 |
| 98 | rs9894220 | G | -0.06 | 0.01 | 0.01 | 0.02 |
| 99 | rs9928094 | G | 0.10 | 0.01 | -0.02 | 0.02 |
| 100 | rs993380 | G | -0.05 | 0.01 | 0.01 | 0.02 |
| 101 | rs9940149 | A | -0.06 | 0.01 | 0.02 | 0.02 |

T2DM: Type-2 diabetes; HDL: High-density lipoprotein.

Table 3. Genetic association estimates for the effect of T2DM on HDL (Willer). ea=effect allele, gx=T2DM, gy=HDL, se=standard error

|  | SNP | ea | gx | gx_se | gy | gy_se |
| --- | --- | --- | --- | --- | --- | --- |
| 1 | rs10077431 | A | -0.05 | 0.01 | 0.01 | 0.00 |
| 2 | rs10100265 | C | -0.05 | 0.01 | 0.00 | 0.00 |
| 3 | rs10114341 | C | -0.04 | 0.01 | 0.01 | 0.00 |
| 4 | rs10401969 | C | 0.09 | 0.01 | 0.01 | 0.01 |
| 5 | rs1050226 | G | -0.05 | 0.01 | 0.00 | 0.00 |
| 6 | rs1061813 | A | -0.04 | 0.01 | -0.01 | 0.00 |
| 7 | rs1063355 | G | 0.07 | 0.01 | 0.01 | 0.01 |
| 8 | rs10740322 | A | 0.05 | 0.01 | 0.00 | 0.01 |
| 9 | rs10811661 | C | -0.16 | 0.01 | 0.00 | 0.00 |
| 10 | rs10830963 | G | 0.09 | 0.01 | 0.00 | 0.00 |
| 11 | rs10842994 | T | -0.08 | 0.01 | 0.01 | 0.00 |
| 12 | rs10974438 | C | 0.06 | 0.01 | 0.01 | 0.00 |
| 13 | rs11098676 | C | 0.05 | 0.01 | -0.01 | 0.01 |
| 14 | rs11107116 | T | 0.05 | 0.01 | -0.01 | 0.00 |
| 15 | rs1111875 | T | -0.09 | 0.01 | 0.01 | 0.00 |
| 16 | rs11257655 | T | 0.07 | 0.01 | 0.00 | 0.00 |
| 17 | rs1127655 | T | -0.04 | 0.01 | 0.00 | 0.00 |
| 18 | rs11708067 | G | -0.10 | 0.01 | -0.02 | 0.00 |
| 19 | rs11925227 | A | -0.05 | 0.01 | 0.00 | 0.00 |
| 20 | rs11926707 | C | 0.05 | 0.01 | -0.01 | 0.01 |
| 21 | rs12088739 | G | -0.09 | 0.01 | 0.00 | 0.01 |
| 22 | rs12299509 | G | 0.05 | 0.01 | 0.00 | 0.00 |
| 23 | rs12617659 | T | -0.07 | 0.01 | 0.02 | 0.00 |
| 24 | rs12910825 | G | 0.05 | 0.01 | 0.00 | 0.00 |
| 25 | rs12945601 | C | -0.05 | 0.01 | 0.01 | 0.00 |
| 26 | rs12970134 | A | 0.06 | 0.01 | -0.02 | 0.00 |
| 27 | rs13234269 | A | -0.06 | 0.01 | 0.04 | 0.00 |
| 28 | rs13239186 | T | 0.05 | 0.01 | 0.00 | 0.01 |
| 29 | rs13330951 | G | -0.05 | 0.01 | 0.01 | 0.00 |
| 30 | rs13389219 | T | -0.07 | 0.01 | 0.02 | 0.00 |
| 31 | rs1359790 | A | -0.08 | 0.01 | 0.00 | 0.00 |
| 32 | rs1496653 | G | -0.08 | 0.01 | 0.00 | 0.00 |
| 33 | rs1552224 | C | -0.10 | 0.01 | 0.00 | 0.00 |
| 34 | rs16988333 | G | -0.07 | 0.01 | 0.01 | 0.01 |
| 35 | rs17086692 | T | -0.05 | 0.01 | 0.00 | 0.01 |
| 36 | rs17168486 | T | 0.07 | 0.01 | 0.01 | 0.00 |
| 37 | rs17405722 | A | 0.09 | 0.01 | -0.01 | 0.01 |
| 38 | rs17411031 | G | -0.05 | 0.01 | 0.10 | 0.00 |
| 39 | rs1758632 | G | 0.05 | 0.01 | -0.01 | 0.00 |
| 40 | rs17631783 | T | -0.05 | 0.01 | 0.00 | 0.01 |
| 41 | rs17791513 | G | -0.10 | 0.01 | -0.01 | 0.01 |
| 42 | rs1801214 | T | 0.09 | 0.01 | 0.00 | 0.00 |
| 43 | rs1899951 | T | -0.11 | 0.01 | 0.01 | 0.01 |
| 44 | rs2058913 | T | -0.05 | 0.01 | 0.01 | 0.00 |
| 45 | rs2237892 | T | -0.10 | 0.02 | -0.01 | 0.01 |
| 46 | rs2246618 | T | 0.05 | 0.01 | -0.01 | 0.00 |
| 47 | rs2261181 | T | 0.10 | 0.01 | -0.01 | 0.01 |
| 48 | rs2294120 | G | -0.04 | 0.01 | -0.01 | 0.00 |
| 49 | rs2296173 | G | 0.07 | 0.01 | -0.04 | 0.00 |
| 50 | rs2299383 | T | 0.04 | 0.01 | 0.00 | 0.00 |
| 51 | rs243019 | C | 0.06 | 0.01 | 0.00 | 0.00 |
| 52 | rs2493394 | G | 0.07 | 0.01 | 0.00 | 0.01 |
| 53 | rs2796441 | A | -0.07 | 0.01 | 0.00 | 0.00 |
| 54 | rs2820426 | G | 0.05 | 0.01 | -0.01 | 0.00 |
| 55 | rs2867125 | C | 0.06 | 0.01 | -0.01 | 0.00 |
| 56 | rs2908282 | A | 0.06 | 0.01 | 0.00 | 0.00 |
| 57 | rs2925979 | C | -0.05 | 0.01 | 0.04 | 0.00 |
| 58 | rs2943656 | G | 0.09 | 0.01 | -0.03 | 0.00 |
| 59 | rs3217992 | T | 0.05 | 0.01 | 0.00 | 0.00 |
| 60 | rs340874 | C | 0.06 | 0.01 | -0.01 | 0.00 |
| 61 | rs3756784 | G | 0.05 | 0.01 | -0.01 | 0.00 |
| 62 | rs3802177 | A | -0.12 | 0.01 | 0.00 | 0.00 |
| 63 | rs459193 | G | 0.07 | 0.01 | -0.02 | 0.00 |
| 64 | rs4622883 | G | -0.04 | 0.01 | 0.01 | 0.00 |
| 65 | rs4812829 | A | 0.05 | 0.01 | 0.00 | 0.00 |
| 66 | rs4823182 | G | 0.05 | 0.01 | -0.01 | 0.00 |
| 67 | rs4865796 | A | 0.05 | 0.01 | -0.01 | 0.00 |
| 68 | rs516946 | C | 0.08 | 0.01 | 0.01 | 0.00 |
| 69 | rs5215 | T | -0.07 | 0.01 | 0.00 | 0.00 |
| 70 | rs55966194 | G | -0.05 | 0.01 | 0.01 | 0.01 |
| 71 | rs576674 | A | -0.07 | 0.01 | -0.01 | 0.00 |
| 72 | rs6059662 | G | 0.04 | 0.01 | -0.01 | 0.00 |
| 73 | rs622217 | C | -0.05 | 0.01 | 0.00 | 0.00 |
| 74 | rs6494307 | G | -0.04 | 0.01 | 0.00 | 0.00 |
| 75 | rs6515236 | C | -0.05 | 0.01 | 0.00 | 0.01 |
| 76 | rs6767484 | G | 0.12 | 0.01 | -0.01 | 0.00 |
| 77 | rs6785040 | C | -0.06 | 0.01 | 0.00 | 0.01 |
| 78 | rs6795735 | T | -0.06 | 0.01 | 0.01 | 0.00 |
| 79 | rs6878122 | A | -0.06 | 0.01 | 0.01 | 0.00 |
| 80 | rs6960043 | C | 0.06 | 0.01 | 0.00 | 0.00 |
| 81 | rs7144011 | T | 0.05 | 0.01 | 0.00 | 0.00 |
| 82 | rs7177055 | A | 0.06 | 0.01 | 0.00 | 0.00 |
| 83 | rs7240767 | C | 0.05 | 0.01 | 0.00 | 0.01 |
| 84 | rs72802358 | C | -0.12 | 0.01 | 0.01 | 0.01 |
| 85 | rs72892910 | T | 0.06 | 0.01 | 0.00 | 0.01 |
| 86 | rs735949 | C | -0.07 | 0.01 | 0.00 | 0.00 |
| 87 | rs753270 | C | 0.05 | 0.01 | -0.01 | 0.01 |
| 88 | rs7561798 | G | 0.04 | 0.01 | -0.01 | 0.00 |
| 89 | rs7572970 | G | 0.06 | 0.01 | -0.01 | 0.01 |
| 90 | rs7607777 | T | -0.14 | 0.01 | 0.00 | 0.01 |
| 91 | rs7685296 | T | -0.05 | 0.01 | 0.01 | 0.00 |
| 92 | rs7729395 | T | 0.14 | 0.02 | -0.01 | 0.01 |
| 93 | rs7756992 | G | 0.13 | 0.01 | 0.00 | 0.00 |
| 94 | rs7786095 | G | -0.07 | 0.01 | 0.00 | 0.01 |
| 95 | rs780094 | C | 0.07 | 0.01 | 0.01 | 0.00 |
| 96 | rs7845219 | C | -0.04 | 0.01 | 0.00 | 0.00 |
| 97 | rs7903146 | T | 0.31 | 0.01 | 0.00 | 0.00 |
| 98 | rs7929543 | C | 0.08 | 0.01 | -0.01 | 0.01 |
| 99 | rs7955901 | T | -0.04 | 0.01 | 0.01 | 0.00 |
| 100 | rs8068804 | A | 0.06 | 0.01 | -0.01 | 0.00 |
| 101 | rs8108269 | G | 0.06 | 0.01 | -0.02 | 0.00 |
| 102 | rs825476 | T | 0.05 | 0.01 | -0.02 | 0.00 |
| 103 | rs840967 | A | -0.05 | 0.01 | 0.00 | 0.00 |
| 104 | rs849135 | A | -0.10 | 0.01 | 0.00 | 0.00 |
| 105 | rs9369425 | A | -0.05 | 0.01 | -0.02 | 0.01 |
| 106 | rs963740 | T | -0.05 | 0.01 | 0.00 | 0.01 |
| 107 | rs9844972 | C | 0.10 | 0.01 | -0.02 | 0.01 |
| 108 | rs9894220 | G | -0.06 | 0.01 | 0.01 | 0.01 |
| 109 | rs9928094 | G | 0.10 | 0.01 | -0.02 | 0.00 |
| 110 | rs993380 | G | -0.05 | 0.01 | 0.00 | 0.00 |
| 111 | rs9940149 | A | -0.06 | 0.01 | 0.00 | 0.00 |

T2DM: Type-2 diabetes; HDL: High-density lipoprotein.

Table 4. Genetic association estimates for the effect of T2DM on HDL (Kettunen). ea=effect allele, gx=T2DM, gy=HDL, se=standard error

|  | SNP | ea | gx | gx_se | gy | gy_se |
| --- | --- | --- | --- | --- | --- | --- |
| 1 | rs10077431 | A | -0.05 | 0.01 | 0.00 | 0.01 |
| 2 | rs10087241 | A | -0.05 | 0.01 | -0.01 | 0.01 |
| 3 | rs10100265 | C | -0.05 | 0.01 | 0.00 | 0.01 |
| 4 | rs10114341 | C | -0.04 | 0.01 | 0.00 | 0.01 |
| 5 | rs10401969 | C | 0.09 | 0.01 | 0.01 | 0.02 |
| 6 | rs1050226 | G | -0.05 | 0.01 | 0.01 | 0.01 |
| 7 | rs1061813 | A | -0.04 | 0.01 | 0.01 | 0.01 |
| 8 | rs1063355 | G | 0.07 | 0.01 | -0.01 | 0.01 |
| 9 | rs10740322 | A | 0.05 | 0.01 | 0.02 | 0.01 |
| 10 | rs10811661 | C | -0.16 | 0.01 | 0.00 | 0.01 |
| 11 | rs10830963 | G | 0.09 | 0.01 | 0.00 | 0.01 |
| 12 | rs10842994 | T | -0.08 | 0.01 | 0.01 | 0.01 |
| 13 | rs10974438 | C | 0.06 | 0.01 | 0.02 | 0.01 |
| 14 | rs11098676 | C | 0.05 | 0.01 | 0.01 | 0.01 |
| 15 | rs11107116 | T | 0.05 | 0.01 | -0.02 | 0.01 |
| 16 | rs1111875 | T | -0.09 | 0.01 | 0.01 | 0.01 |
| 17 | rs11257655 | T | 0.07 | 0.01 | 0.01 | 0.01 |
| 18 | rs1127655 | T | -0.04 | 0.01 | 0.01 | 0.01 |
| 19 | rs11708067 | G | -0.10 | 0.01 | 0.00 | 0.01 |
| 20 | rs11925227 | A | -0.05 | 0.01 | -0.02 | 0.02 |
| 21 | rs11926707 | C | 0.05 | 0.01 | -0.01 | 0.01 |
| 22 | rs12088739 | G | -0.09 | 0.01 | 0.00 | 0.02 |
| 23 | rs12299509 | G | 0.05 | 0.01 | 0.02 | 0.01 |
| 24 | rs12617659 | T | -0.07 | 0.01 | 0.00 | 0.01 |
| 25 | rs12910825 | G | 0.05 | 0.01 | -0.01 | 0.01 |
| 26 | rs12945601 | C | -0.05 | 0.01 | 0.01 | 0.01 |
| 27 | rs12970134 | A | 0.06 | 0.01 | -0.02 | 0.01 |
| 28 | rs13234269 | A | -0.06 | 0.01 | 0.02 | 0.01 |
| 29 | rs13239186 | T | 0.05 | 0.01 | 0.00 | 0.01 |
| 30 | rs13330951 | G | -0.05 | 0.01 | 0.01 | 0.01 |
| 31 | rs13389219 | T | -0.07 | 0.01 | 0.04 | 0.01 |
| 32 | rs1359790 | A | -0.08 | 0.01 | 0.01 | 0.01 |
| 33 | rs1496653 | G | -0.08 | 0.01 | 0.00 | 0.01 |
| 34 | rs1552224 | C | -0.10 | 0.01 | -0.02 | 0.01 |
| 35 | rs16988333 | G | -0.07 | 0.01 | 0.00 | 0.02 |
| 36 | rs17086692 | T | -0.05 | 0.01 | -0.01 | 0.01 |
| 37 | rs17168486 | T | 0.07 | 0.01 | 0.01 | 0.01 |
| 38 | rs17405722 | A | 0.09 | 0.01 | 0.00 | 0.02 |
| 39 | rs17411031 | G | -0.05 | 0.01 | 0.09 | 0.01 |
| 40 | rs1758632 | G | 0.05 | 0.01 | -0.01 | 0.01 |
| 41 | rs17631783 | T | -0.05 | 0.01 | -0.01 | 0.01 |
| 42 | rs17791513 | G | -0.10 | 0.01 | -0.02 | 0.02 |
| 43 | rs1801214 | T | 0.09 | 0.01 | 0.00 | 0.01 |
| 44 | rs1899951 | T | -0.11 | 0.01 | 0.02 | 0.01 |
| 45 | rs2058913 | T | -0.05 | 0.01 | -0.01 | 0.01 |
| 46 | rs2237892 | T | -0.10 | 0.02 | 0.00 | 0.02 |
| 47 | rs2246618 | T | 0.05 | 0.01 | -0.02 | 0.01 |
| 48 | rs2261181 | T | 0.10 | 0.01 | 0.00 | 0.02 |
| 49 | rs2294120 | G | -0.04 | 0.01 | 0.01 | 0.01 |
| 50 | rs2296173 | G | 0.07 | 0.01 | -0.01 | 0.01 |
| 51 | rs2299383 | T | 0.04 | 0.01 | -0.01 | 0.01 |
| 52 | rs243019 | C | 0.06 | 0.01 | 0.00 | 0.01 |
| 53 | rs2493394 | G | 0.07 | 0.01 | 0.02 | 0.01 |
| 54 | rs2796441 | A | -0.07 | 0.01 | 0.01 | 0.01 |
| 55 | rs2820426 | G | 0.05 | 0.01 | -0.01 | 0.01 |
| 56 | rs2867125 | C | 0.06 | 0.01 | -0.02 | 0.01 |
| 57 | rs2908282 | A | 0.06 | 0.01 | 0.00 | 0.02 |
| 58 | rs2925979 | C | -0.05 | 0.01 | 0.01 | 0.01 |
| 59 | rs2943656 | G | 0.09 | 0.01 | -0.04 | 0.01 |
| 60 | rs3217992 | T | 0.05 | 0.01 | 0.02 | 0.01 |
| 61 | rs340874 | C | 0.06 | 0.01 | -0.01 | 0.01 |
| 62 | rs348330 | A | -0.05 | 0.01 | 0.00 | 0.01 |
| 63 | rs3756784 | G | 0.05 | 0.01 | -0.02 | 0.01 |
| 64 | rs3802177 | A | -0.12 | 0.01 | 0.00 | 0.01 |
| 65 | rs459193 | G | 0.07 | 0.01 | -0.04 | 0.01 |
| 66 | rs4622883 | G | -0.04 | 0.01 | 0.00 | 0.01 |
| 67 | rs4686471 | C | 0.05 | 0.01 | -0.01 | 0.01 |
| 68 | rs4812829 | A | 0.05 | 0.01 | 0.01 | 0.01 |
| 69 | rs4823182 | G | 0.05 | 0.01 | -0.01 | 0.01 |
| 70 | rs4865796 | A | 0.05 | 0.01 | -0.02 | 0.01 |
| 71 | rs516946 | C | 0.08 | 0.01 | 0.00 | 0.01 |
| 72 | rs5215 | T | -0.07 | 0.01 | -0.02 | 0.01 |
| 73 | rs55966194 | G | -0.05 | 0.01 | -0.01 | 0.01 |
| 74 | rs576674 | A | -0.07 | 0.01 | 0.01 | 0.02 |
| 75 | rs6059662 | G | 0.04 | 0.01 | -0.02 | 0.01 |
| 76 | rs61953351 | T | -0.07 | 0.01 | -0.01 | 0.01 |
| 77 | rs622217 | C | -0.05 | 0.01 | 0.02 | 0.01 |
| 78 | rs6494307 | G | -0.04 | 0.01 | 0.01 | 0.01 |
| 79 | rs6515236 | C | -0.05 | 0.01 | 0.01 | 0.01 |
| 80 | rs67232546 | T | 0.06 | 0.01 | 0.03 | 0.01 |
| 81 | rs6767484 | G | 0.12 | 0.01 | -0.02 | 0.01 |
| 82 | rs6785040 | C | -0.06 | 0.01 | 0.00 | 0.01 |
| 83 | rs6795735 | T | -0.06 | 0.01 | 0.01 | 0.01 |
| 84 | rs6878122 | A | -0.06 | 0.01 | 0.00 | 0.01 |
| 85 | rs6960043 | C | 0.06 | 0.01 | 0.00 | 0.01 |
| 86 | rs7144011 | T | 0.05 | 0.01 | -0.02 | 0.01 |
| 87 | rs7177055 | A | 0.06 | 0.01 | -0.01 | 0.01 |
| 88 | rs7240767 | C | 0.05 | 0.01 | 0.00 | 0.01 |
| 89 | rs72802358 | C | -0.12 | 0.01 | 0.05 | 0.02 |
| 90 | rs72892910 | T | 0.06 | 0.01 | 0.00 | 0.01 |
| 91 | rs735949 | C | -0.07 | 0.01 | 0.02 | 0.02 |
| 92 | rs753270 | C | 0.05 | 0.01 | -0.01 | 0.01 |
| 93 | rs7561798 | G | 0.04 | 0.01 | -0.01 | 0.01 |
| 94 | rs7572970 | G | 0.06 | 0.01 | 0.02 | 0.01 |
| 95 | rs7607777 | T | -0.14 | 0.01 | -0.01 | 0.02 |
| 96 | rs7674212 | T | -0.05 | 0.01 | 0.01 | 0.01 |
| 97 | rs7685296 | T | -0.05 | 0.01 | 0.03 | 0.01 |
| 98 | rs7729395 | T | 0.14 | 0.02 | 0.03 | 0.02 |
| 99 | rs7756992 | G | 0.13 | 0.01 | -0.01 | 0.01 |
| 100 | rs7786095 | G | -0.07 | 0.01 | 0.02 | 0.02 |
| 101 | rs780094 | C | 0.07 | 0.01 | 0.00 | 0.01 |
| 102 | rs7845219 | C | -0.04 | 0.01 | -0.01 | 0.01 |
| 103 | rs7903146 | T | 0.31 | 0.01 | -0.03 | 0.01 |
| 104 | rs7929543 | C | 0.08 | 0.01 | -0.02 | 0.02 |
| 105 | rs7955901 | T | -0.04 | 0.01 | 0.00 | 0.01 |
| 106 | rs8068804 | A | 0.06 | 0.01 | -0.01 | 0.01 |
| 107 | rs8108269 | G | 0.06 | 0.01 | 0.01 | 0.01 |
| 108 | rs825476 | T | 0.05 | 0.01 | -0.02 | 0.01 |
| 109 | rs840967 | A | -0.05 | 0.01 | -0.01 | 0.01 |
| 110 | rs849135 | A | -0.10 | 0.01 | 0.01 | 0.01 |
| 111 | rs853974 | C | -0.06 | 0.01 | 0.01 | 0.01 |
| 112 | rs9369425 | A | -0.05 | 0.01 | -0.02 | 0.01 |
| 113 | rs963740 | T | -0.05 | 0.01 | 0.00 | 0.01 |
| 114 | rs9844972 | C | 0.10 | 0.01 | 0.00 | 0.03 |
| 115 | rs9894220 | G | -0.06 | 0.01 | 0.01 | 0.01 |
| 116 | rs9928094 | G | 0.10 | 0.01 | -0.01 | 0.01 |
| 117 | rs993380 | G | -0.05 | 0.01 | 0.00 | 0.01 |
| 118 | rs9940149 | A | -0.06 | 0.01 | 0.03 | 0.02 |

T2DM: Type-2 diabetes; HDL: High-density lipoprotein.

Table 5. Genetic association estimates for the effect of T2DM on LDL (Willer). ea=effect allele, gx=T2DM, gy=LDL, se=standard error

|  | SNP | ea | gx | gx_se | gy | gy_se |
| --- | --- | --- | --- | --- | --- | --- |
| 1 | rs10077431 | A | -0.05 | 0.01 | 0.01 | 0.00 |
| 2 | rs10100265 | C | -0.05 | 0.01 | -0.01 | 0.00 |
| 3 | rs10114341 | C | -0.04 | 0.01 | 0.00 | 0.00 |
| 4 | rs10401969 | C | 0.09 | 0.01 | -0.12 | 0.01 |
| 5 | rs1050226 | G | -0.05 | 0.01 | 0.00 | 0.00 |
| 6 | rs1061813 | A | -0.04 | 0.01 | 0.00 | 0.00 |
| 7 | rs1063355 | G | 0.07 | 0.01 | -0.02 | 0.01 |
| 8 | rs10740322 | A | 0.05 | 0.01 | 0.00 | 0.01 |
| 9 | rs10811661 | C | -0.16 | 0.01 | 0.00 | 0.00 |
| 10 | rs10830963 | G | 0.09 | 0.01 | 0.00 | 0.00 |
| 11 | rs10842994 | T | -0.08 | 0.01 | 0.00 | 0.00 |
| 12 | rs10974438 | C | 0.06 | 0.01 | 0.00 | 0.00 |
| 13 | rs11098676 | C | 0.05 | 0.01 | 0.00 | 0.01 |
| 14 | rs11107116 | T | 0.05 | 0.01 | -0.01 | 0.00 |
| 15 | rs1111875 | T | -0.09 | 0.01 | -0.01 | 0.00 |
| 16 | rs11257655 | T | 0.07 | 0.01 | 0.00 | 0.00 |
| 17 | rs1127655 | T | -0.04 | 0.01 | 0.00 | 0.01 |
| 18 | rs11708067 | G | -0.10 | 0.01 | -0.01 | 0.00 |
| 19 | rs11925227 | A | -0.05 | 0.01 | -0.01 | 0.00 |
| 20 | rs11926707 | C | 0.05 | 0.01 | 0.01 | 0.01 |
| 21 | rs12088739 | G | -0.09 | 0.01 | 0.00 | 0.01 |
| 22 | rs12299509 | G | 0.05 | 0.01 | 0.00 | 0.00 |
| 23 | rs12617659 | T | -0.07 | 0.01 | 0.01 | 0.01 |
| 24 | rs12910825 | G | 0.05 | 0.01 | 0.00 | 0.00 |
| 25 | rs12945601 | C | -0.05 | 0.01 | 0.00 | 0.01 |
| 26 | rs12970134 | A | 0.06 | 0.01 | 0.00 | 0.00 |
| 27 | rs13234269 | A | -0.06 | 0.01 | -0.01 | 0.01 |
| 28 | rs13239186 | T | 0.05 | 0.01 | 0.00 | 0.01 |
| 29 | rs13330951 | G | -0.05 | 0.01 | 0.00 | 0.01 |
| 30 | rs13389219 | T | -0.07 | 0.01 | -0.02 | 0.00 |
| 31 | rs1359790 | A | -0.08 | 0.01 | 0.00 | 0.00 |
| 32 | rs1496653 | G | -0.08 | 0.01 | 0.00 | 0.00 |
| 33 | rs1552224 | C | -0.10 | 0.01 | 0.00 | 0.00 |
| 34 | rs16988333 | G | -0.07 | 0.01 | -0.01 | 0.01 |
| 35 | rs17086692 | T | -0.05 | 0.01 | 0.00 | 0.01 |
| 36 | rs17168486 | T | 0.07 | 0.01 | 0.00 | 0.00 |
| 37 | rs17405722 | A | 0.09 | 0.01 | 0.00 | 0.01 |
| 38 | rs17411031 | G | -0.05 | 0.01 | -0.01 | 0.00 |
| 39 | rs1758632 | G | 0.05 | 0.01 | 0.00 | 0.01 |
| 40 | rs17631783 | T | -0.05 | 0.01 | -0.01 | 0.01 |
| 41 | rs17791513 | G | -0.10 | 0.01 | 0.00 | 0.01 |
| 42 | rs1801214 | T | 0.09 | 0.01 | 0.00 | 0.00 |
| 43 | rs1899951 | T | -0.11 | 0.01 | 0.00 | 0.01 |
| 44 | rs2058913 | T | -0.05 | 0.01 | 0.01 | 0.01 |
| 45 | rs2237892 | T | -0.10 | 0.02 | 0.00 | 0.01 |
| 46 | rs2246618 | T | 0.05 | 0.01 | 0.00 | 0.00 |
| 47 | rs2261181 | T | 0.10 | 0.01 | 0.01 | 0.01 |
| 48 | rs2294120 | G | -0.04 | 0.01 | 0.00 | 0.00 |
| 49 | rs2296173 | G | 0.07 | 0.01 | 0.01 | 0.00 |
| 50 | rs2299383 | T | 0.04 | 0.01 | 0.00 | 0.00 |
| 51 | rs243019 | C | 0.06 | 0.01 | 0.00 | 0.00 |
| 52 | rs2493394 | G | 0.07 | 0.01 | 0.00 | 0.01 |
| 53 | rs2796441 | A | -0.07 | 0.01 | 0.00 | 0.00 |
| 54 | rs2820426 | G | 0.05 | 0.01 | 0.01 | 0.00 |
| 55 | rs2867125 | C | 0.06 | 0.01 | -0.01 | 0.00 |
| 56 | rs2908282 | A | 0.06 | 0.01 | 0.00 | 0.01 |
| 57 | rs2925979 | C | -0.05 | 0.01 | 0.00 | 0.00 |
| 58 | rs2943656 | G | 0.09 | 0.01 | 0.01 | 0.00 |
| 59 | rs3217992 | T | 0.05 | 0.01 | 0.00 | 0.00 |
| 60 | rs340874 | C | 0.06 | 0.01 | 0.01 | 0.00 |
| 61 | rs3756784 | G | 0.05 | 0.01 | -0.01 | 0.00 |
| 62 | rs3802177 | A | -0.12 | 0.01 | -0.01 | 0.00 |
| 63 | rs459193 | G | 0.07 | 0.01 | -0.01 | 0.00 |
| 64 | rs4622883 | G | -0.04 | 0.01 | 0.01 | 0.01 |
| 65 | rs4812829 | A | 0.05 | 0.01 | 0.00 | 0.00 |
| 66 | rs4823182 | G | 0.05 | 0.01 | -0.01 | 0.00 |
| 67 | rs4865796 | A | 0.05 | 0.01 | 0.00 | 0.00 |
| 68 | rs516946 | C | 0.08 | 0.01 | 0.00 | 0.00 |
| 69 | rs5215 | T | -0.07 | 0.01 | -0.01 | 0.00 |
| 70 | rs55966194 | G | -0.05 | 0.01 | -0.01 | 0.01 |
| 71 | rs576674 | A | -0.07 | 0.01 | 0.00 | 0.01 |
| 72 | rs6059662 | G | 0.04 | 0.01 | -0.01 | 0.00 |
| 73 | rs622217 | C | -0.05 | 0.01 | -0.02 | 0.00 |
| 74 | rs6494307 | G | -0.04 | 0.01 | 0.01 | 0.01 |
| 75 | rs6515236 | C | -0.05 | 0.01 | 0.01 | 0.01 |
| 76 | rs6767484 | G | 0.12 | 0.01 | 0.00 | 0.00 |
| 77 | rs6785040 | C | -0.06 | 0.01 | 0.00 | 0.01 |
| 78 | rs6795735 | T | -0.06 | 0.01 | -0.01 | 0.00 |
| 79 | rs6878122 | A | -0.06 | 0.01 | -0.01 | 0.00 |
| 80 | rs6960043 | C | 0.06 | 0.01 | 0.00 | 0.00 |
| 81 | rs7144011 | T | 0.05 | 0.01 | -0.01 | 0.00 |
| 82 | rs7177055 | A | 0.06 | 0.01 | 0.01 | 0.00 |
| 83 | rs7240767 | C | 0.05 | 0.01 | 0.01 | 0.01 |
| 84 | rs72802358 | C | -0.12 | 0.01 | 0.00 | 0.01 |
| 85 | rs72892910 | T | 0.06 | 0.01 | 0.00 | 0.01 |
| 86 | rs735949 | C | -0.07 | 0.01 | 0.01 | 0.01 |
| 87 | rs753270 | C | 0.05 | 0.01 | 0.00 | 0.01 |
| 88 | rs7561798 | G | 0.04 | 0.01 | 0.00 | 0.00 |
| 89 | rs7572970 | G | 0.06 | 0.01 | 0.00 | 0.01 |
| 90 | rs7607777 | T | -0.14 | 0.01 | -0.01 | 0.01 |
| 91 | rs7685296 | T | -0.05 | 0.01 | 0.00 | 0.00 |
| 92 | rs7729395 | T | 0.14 | 0.02 | 0.00 | 0.01 |
| 93 | rs7756992 | G | 0.13 | 0.01 | 0.00 | 0.00 |
| 94 | rs7786095 | G | -0.07 | 0.01 | -0.01 | 0.01 |
| 95 | rs780094 | C | 0.07 | 0.01 | -0.02 | 0.00 |
| 96 | rs7845219 | C | -0.04 | 0.01 | 0.00 | 0.00 |
| 97 | rs7903146 | T | 0.31 | 0.01 | 0.01 | 0.00 |
| 98 | rs7929543 | C | 0.08 | 0.01 | 0.00 | 0.01 |
| 99 | rs7955901 | T | -0.04 | 0.01 | 0.00 | 0.00 |
| 100 | rs8068804 | A | 0.06 | 0.01 | 0.01 | 0.00 |
| 101 | rs8108269 | G | 0.06 | 0.01 | -0.02 | 0.00 |
| 102 | rs825476 | T | 0.05 | 0.01 | 0.00 | 0.00 |
| 103 | rs840967 | A | -0.05 | 0.01 | -0.01 | 0.01 |
| 104 | rs849135 | A | -0.10 | 0.01 | 0.00 | 0.00 |
| 105 | rs9369425 | A | -0.05 | 0.01 | 0.01 | 0.01 |
| 106 | rs963740 | T | -0.05 | 0.01 | 0.00 | 0.01 |
| 107 | rs9844972 | C | 0.10 | 0.01 | 0.01 | 0.01 |
| 108 | rs9894220 | G | -0.06 | 0.01 | 0.00 | 0.01 |
| 109 | rs9928094 | G | 0.10 | 0.01 | 0.00 | 0.00 |
| 110 | rs993380 | G | -0.05 | 0.01 | 0.00 | 0.01 |
| 111 | rs9940149 | A | -0.06 | 0.01 | 0.00 | 0.01 |

T2DM: Type-2 diabetes; LDL: Low-density lipoprotein.

Table 6. Genetic association estimates for the effect of T2DM on LDL (Prins). ea=effect allele, gx=T2DM, gy=LDL, se=standard error

|  | SNP | ea | gx | gx_se | gy | gy_se |
| --- | --- | --- | --- | --- | --- | --- |
| 1 | rs10077431 | A | -0.05 | -0.01 | 0.02 | 0.01 |
| 2 | rs10100265 | C | -0.05 | -0.03 | 0.02 | 0.01 |
| 3 | rs10114341 | C | -0.04 | -0.01 | 0.01 | 0.01 |
| 4 | rs10401969 | C | 0.09 | -0.12 | 0.03 | 0.01 |
| 5 | rs1050226 | G | -0.05 | -0.01 | 0.01 | 0.01 |
| 6 | rs1061813 | A | -0.04 | 0.02 | 0.01 | 0.01 |
| 7 | rs1063355 | G | 0.07 | -0.01 | 0.02 | 0.01 |
| 8 | rs10740322 | A | 0.05 | -0.04 | 0.02 | 0.01 |
| 9 | rs10811661 | C | -0.16 | 0.05 | 0.02 | 0.01 |
| 10 | rs10830963 | G | 0.09 | -0.01 | 0.02 | 0.01 |
| 11 | rs10842994 | T | -0.08 | 0.01 | 0.02 | 0.01 |
| 12 | rs10974438 | C | 0.06 | -0.01 | 0.02 | 0.01 |
| 13 | rs11107116 | T | 0.05 | 0.03 | 0.02 | 0.01 |
| 14 | rs1111875 | T | -0.09 | -0.01 | 0.01 | 0.01 |
| 15 | rs11257655 | T | 0.07 | 0.01 | 0.02 | 0.01 |
| 16 | rs1127655 | T | -0.04 | 0.02 | 0.01 | 0.01 |
| 17 | rs11708067 | G | -0.10 | -0.02 | 0.02 | 0.01 |
| 18 | rs12088739 | G | -0.09 | 0.01 | 0.03 | 0.01 |
| 19 | rs12617659 | T | -0.07 | 0.01 | 0.02 | 0.01 |
| 20 | rs12910825 | G | 0.05 | 0.03 | 0.02 | 0.01 |
| 21 | rs12945601 | C | -0.05 | -0.01 | 0.02 | 0.01 |
| 22 | rs12970134 | A | 0.06 | 0.02 | 0.02 | 0.01 |
| 23 | rs13234269 | A | -0.06 | 0.03 | 0.01 | 0.01 |
| 24 | rs13239186 | T | 0.05 | -0.02 | 0.02 | 0.01 |
| 25 | rs13389219 | T | -0.07 | 0.02 | 0.02 | 0.01 |
| 26 | rs1359790 | A | -0.08 | 0.02 | 0.02 | 0.01 |
| 27 | rs1496653 | G | -0.08 | -0.01 | 0.02 | 0.01 |
| 28 | rs1552224 | C | -0.10 | -0.02 | 0.02 | 0.01 |
| 29 | rs16988333 | G | -0.07 | 0.01 | 0.02 | 0.01 |
| 30 | rs17086692 | T | -0.05 | 0.02 | 0.02 | 0.01 |
| 31 | rs17168486 | T | 0.07 | -0.01 | 0.02 | 0.01 |
| 32 | rs17405722 | A | 0.09 | 0.02 | 0.03 | 0.01 |
| 33 | rs17411031 | G | -0.05 | -0.01 | 0.02 | 0.01 |
| 34 | rs1758632 | G | 0.05 | -0.02 | 0.01 | 0.01 |
| 35 | rs17631783 | T | -0.05 | 0.01 | 0.02 | 0.01 |
| 36 | rs17791513 | G | -0.10 | -0.05 | 0.03 | 0.01 |
| 37 | rs1801214 | T | 0.09 | -0.01 | 0.01 | 0.01 |
| 38 | rs1899951 | T | -0.11 | -0.01 | 0.02 | 0.01 |
| 39 | rs2058913 | T | -0.05 | -0.02 | 0.01 | 0.01 |
| 40 | rs2237892 | T | -0.10 | 0.04 | 0.03 | 0.02 |
| 41 | rs2246618 | T | 0.05 | -0.01 | 0.02 | 0.01 |
| 42 | rs2261181 | T | 0.10 | -0.02 | 0.02 | 0.01 |
| 43 | rs2294120 | G | -0.04 | -0.01 | 0.01 | 0.01 |
| 44 | rs2296173 | G | 0.07 | -0.04 | 0.02 | 0.01 |
| 45 | rs2299383 | T | 0.04 | 0.01 | 0.01 | 0.01 |
| 46 | rs243019 | C | 0.06 | -0.01 | 0.01 | 0.01 |
| 47 | rs2493394 | G | 0.07 | 0.01 | 0.02 | 0.01 |
| 48 | rs2796441 | A | -0.07 | 0.02 | 0.01 | 0.01 |
| 49 | rs2820426 | G | 0.05 | 0.01 | 0.02 | 0.01 |
| 50 | rs2867125 | C | 0.06 | 0.01 | 0.02 | 0.01 |
| 51 | rs2908282 | A | 0.06 | -0.01 | 0.02 | 0.01 |
| 52 | rs2925979 | C | -0.05 | -0.01 | 0.02 | 0.01 |
| 53 | rs2943656 | G | 0.09 | -0.02 | 0.02 | 0.01 |
| 54 | rs3217992 | T | 0.05 | 0.01 | 0.02 | 0.01 |
| 55 | rs340874 | C | 0.06 | -0.01 | 0.01 | 0.01 |
| 56 | rs3756784 | G | 0.05 | -0.01 | 0.02 | 0.01 |
| 57 | rs3802177 | A | -0.12 | 0.01 | 0.02 | 0.01 |
| 58 | rs459193 | G | 0.07 | 0.02 | 0.02 | 0.01 |
| 59 | rs4622883 | G | -0.04 | -0.01 | 0.01 | 0.01 |
| 60 | rs4686471 | C | 0.05 | -0.01 | 0.01 | 0.01 |
| 61 | rs4812829 | A | 0.05 | 0.01 | 0.02 | 0.01 |
| 62 | rs4865796 | A | 0.05 | 0.01 | 0.02 | 0.01 |
| 63 | rs516946 | C | 0.08 | -0.01 | 0.02 | 0.01 |
| 64 | rs5215 | T | -0.07 | -0.01 | 0.02 | 0.01 |
| 65 | rs55966194 | G | -0.05 | 0.01 | 0.02 | 0.01 |
| 66 | rs576674 | A | -0.07 | -0.01 | 0.02 | 0.01 |
| 67 | rs6059662 | G | 0.04 | -0.02 | 0.02 | 0.01 |
| 68 | rs61953351 | T | -0.07 | -0.02 | 0.02 | 0.01 |
| 69 | rs6494307 | G | -0.04 | 0.02 | 0.01 | 0.01 |
| 70 | rs67232546 | T | 0.06 | 0.02 | 0.02 | 0.01 |
| 71 | rs6767484 | G | 0.12 | 0.02 | 0.02 | 0.01 |
| 72 | rs6785040 | C | -0.06 | 0.01 | 0.02 | 0.01 |
| 73 | rs6795735 | T | -0.06 | 0.01 | 0.01 | 0.01 |
| 74 | rs6960043 | C | 0.06 | -0.01 | 0.01 | 0.01 |
| 75 | rs7144011 | T | 0.05 | -0.01 | 0.02 | 0.01 |
| 76 | rs7177055 | A | 0.06 | 0.02 | 0.02 | 0.01 |
| 77 | rs7240767 | C | 0.05 | -0.01 | 0.01 | 0.01 |
| 78 | rs72892910 | T | 0.06 | 0.04 | 0.02 | 0.01 |
| 79 | rs735949 | C | -0.07 | 0.03 | 0.02 | 0.01 |
| 80 | rs7572970 | G | 0.06 | 0.01 | 0.02 | 0.01 |
| 81 | rs7607777 | T | -0.14 | 0.01 | 0.02 | 0.01 |
| 82 | rs7674212 | T | -0.05 | -0.01 | 0.02 | 0.01 |
| 83 | rs7685296 | T | -0.05 | 0.03 | 0.02 | 0.01 |
| 84 | rs7729395 | T | 0.14 | 0.01 | 0.03 | 0.02 |
| 85 | rs7756992 | G | 0.13 | 0.01 | 0.02 | 0.01 |
| 86 | rs7786095 | G | -0.07 | 0.07 | 0.02 | 0.01 |
| 87 | rs780094 | C | 0.07 | -0.01 | 0.01 | 0.01 |
| 88 | rs7845219 | C | -0.04 | -0.01 | 0.01 | 0.01 |
| 89 | rs7903146 | T | 0.31 | -0.03 | 0.02 | 0.01 |
| 90 | rs7929543 | C | 0.08 | -0.05 | 0.03 | 0.01 |
| 91 | rs7955901 | T | -0.04 | 0.01 | 0.01 | 0.01 |
| 92 | rs8068804 | A | 0.06 | 0.01 | 0.02 | 0.01 |
| 93 | rs8108269 | G | 0.06 | 0.02 | 0.02 | 0.01 |
| 94 | rs825476 | T | 0.05 | -0.01 | 0.01 | 0.01 |
| 95 | rs840967 | A | -0.05 | -0.01 | 0.01 | 0.01 |
| 96 | rs849135 | A | -0.10 | -0.01 | 0.01 | 0.01 |
| 97 | rs853974 | C | -0.06 | 0.02 | 0.02 | 0.01 |
| 98 | rs9369425 | A | -0.05 | -0.01 | 0.02 | 0.01 |
| 99 | rs963740 | T | -0.05 | 0.01 | 0.02 | 0.01 |
| 100 | rs9844972 | C | 0.10 | -0.01 | 0.03 | 0.01 |
| 101 | rs9894220 | G | -0.06 | 0.01 | 0.01 | 0.01 |
| 102 | rs9928094 | G | 0.10 | -0.01 | 0.02 | 0.01 |
| 103 | rs9940149 | A | -0.06 | -0.02 | 0.02 | 0.01 |

T2DM: Type-2 diabetes; LDL: Low-density lipoprotein.

Table 7. Genetic association estimates for the effect of T2DM on LDL (Kanai). ea=effect allele, gx=T2DM, gy=LDL, se=standard error

|  | SNP | ea | gx | gx_se | gy | gy_se |
| --- | --- | --- | --- | --- | --- | --- |
| 1 | rs10077431 | A | -0.05 | 0.01 | 0.00 | 0.01 |
| 2 | rs10087241 | A | -0.05 | 0.01 | 0.00 | 0.01 |
| 3 | rs10100265 | C | -0.05 | 0.01 | -0.01 | 0.01 |
| 4 | rs10114341 | C | -0.04 | 0.01 | -0.01 | 0.01 |
| 5 | rs10401969 | C | 0.09 | 0.01 | 0.01 | 0.01 |
| 6 | rs1050226 | G | -0.05 | 0.01 | 0.01 | 0.01 |
| 7 | rs1061813 | A | -0.04 | 0.01 | 0.01 | 0.01 |
| 8 | rs1063355 | G | 0.07 | 0.01 | -0.01 | 0.01 |
| 9 | rs10740322 | A | 0.05 | 0.01 | -0.01 | 0.01 |
| 10 | rs10811661 | C | -0.16 | 0.01 | 0.00 | 0.01 |
| 11 | rs10830963 | G | 0.09 | 0.01 | -0.01 | 0.01 |
| 12 | rs10842994 | T | -0.08 | 0.01 | 0.00 | 0.01 |
| 13 | rs10974438 | C | 0.06 | 0.01 | 0.01 | 0.01 |
| 14 | rs11098676 | C | 0.05 | 0.01 | 0.02 | 0.01 |
| 15 | rs11107116 | T | 0.05 | 0.01 | -0.01 | 0.01 |
| 16 | rs1111875 | T | -0.09 | 0.01 | 0.01 | 0.01 |
| 17 | rs11257655 | T | 0.07 | 0.01 | 0.00 | 0.01 |
| 18 | rs1127655 | T | -0.04 | 0.01 | 0.01 | 0.01 |
| 19 | rs11926707 | C | 0.05 | 0.01 | 0.00 | 0.01 |
| 20 | rs12088739 | G | -0.09 | 0.01 | -0.01 | 0.01 |
| 21 | rs12299509 | G | 0.05 | 0.01 | 0.00 | 0.01 |
| 22 | rs12617659 | T | -0.07 | 0.01 | 0.02 | 0.01 |
| 23 | rs12945601 | C | -0.05 | 0.01 | -0.01 | 0.01 |
| 24 | rs12970134 | A | 0.06 | 0.01 | 0.01 | 0.01 |
| 25 | rs13234269 | A | -0.06 | 0.01 | 0.00 | 0.01 |
| 26 | rs13239186 | T | 0.05 | 0.01 | 0.00 | 0.01 |
| 27 | rs13330951 | G | -0.05 | 0.01 | -0.01 | 0.01 |
| 28 | rs13389219 | T | -0.07 | 0.01 | 0.01 | 0.01 |
| 29 | rs1359790 | A | -0.08 | 0.01 | -0.01 | 0.01 |
| 30 | rs1496653 | G | -0.08 | 0.01 | 0.00 | 0.01 |
| 31 | rs1552224 | C | -0.10 | 0.01 | 0.01 | 0.01 |
| 32 | rs17086692 | T | -0.05 | 0.01 | 0.00 | 0.01 |
| 33 | rs17168486 | T | 0.07 | 0.01 | 0.00 | 0.01 |
| 34 | rs17405722 | A | 0.09 | 0.01 | 0.02 | 0.02 |
| 35 | rs17411031 | G | -0.05 | 0.01 | 0.01 | 0.01 |
| 36 | rs1758632 | G | 0.05 | 0.01 | 0.01 | 0.01 |
| 37 | rs17631783 | T | -0.05 | 0.01 | 0.01 | 0.01 |
| 38 | rs17791513 | G | -0.10 | 0.01 | -0.01 | 0.01 |
| 39 | rs1801214 | T | 0.09 | 0.01 | 0.03 | 0.02 |
| 40 | rs1899951 | T | -0.11 | 0.01 | -0.01 | 0.02 |
| 41 | rs2058913 | T | -0.05 | 0.01 | 0.00 | 0.01 |
| 42 | rs2237892 | T | -0.10 | 0.02 | -0.01 | 0.01 |
| 43 | rs2246618 | T | 0.05 | 0.01 | 0.00 | 0.01 |
| 44 | rs2261181 | T | 0.10 | 0.01 | 0.00 | 0.01 |
| 45 | rs2294120 | G | -0.04 | 0.01 | 0.00 | 0.01 |
| 46 | rs2296173 | G | 0.07 | 0.01 | 0.00 | 0.01 |
| 47 | rs2299383 | T | 0.04 | 0.01 | 0.00 | 0.01 |
| 48 | rs243019 | C | 0.06 | 0.01 | 0.01 | 0.01 |
| 49 | rs2493394 | G | 0.07 | 0.01 | 0.02 | 0.02 |
| 50 | rs2796441 | A | -0.07 | 0.01 | 0.00 | 0.01 |
| 51 | rs2820426 | G | 0.05 | 0.01 | 0.00 | 0.01 |
| 52 | rs2867125 | C | 0.06 | 0.01 | 0.00 | 0.01 |
| 53 | rs2908282 | A | 0.06 | 0.01 | -0.01 | 0.01 |
| 54 | rs2925979 | C | -0.05 | 0.01 | 0.00 | 0.01 |
| 55 | rs2943656 | G | 0.09 | 0.01 | 0.00 | 0.01 |
| 56 | rs3217992 | T | 0.05 | 0.01 | 0.00 | 0.01 |
| 57 | rs340874 | C | 0.06 | 0.01 | 0.00 | 0.01 |
| 58 | rs348330 | A | -0.05 | 0.01 | 0.01 | 0.01 |
| 59 | rs3756784 | G | 0.05 | 0.01 | 0.00 | 0.01 |
| 60 | rs3802177 | A | -0.12 | 0.01 | 0.00 | 0.01 |
| 61 | rs459193 | G | 0.07 | 0.01 | -0.01 | 0.01 |
| 62 | rs4622883 | G | -0.04 | 0.01 | 0.00 | 0.01 |
| 63 | rs4812829 | A | 0.05 | 0.01 | 0.01 | 0.01 |
| 64 | rs4823182 | G | 0.05 | 0.01 | 0.00 | 0.01 |
| 65 | rs4865796 | A | 0.05 | 0.01 | -0.01 | 0.01 |
| 66 | rs516946 | C | 0.08 | 0.01 | 0.01 | 0.01 |
| 67 | rs5215 | T | -0.07 | 0.01 | 0.00 | 0.01 |
| 68 | rs576674 | A | -0.07 | 0.01 | 0.00 | 0.01 |
| 69 | rs6059662 | G | 0.04 | 0.01 | 0.01 | 0.01 |
| 70 | rs622217 | C | -0.05 | 0.01 | 0.00 | 0.01 |
| 71 | rs6494307 | G | -0.04 | 0.01 | -0.01 | 0.01 |
| 72 | rs6515236 | C | -0.05 | 0.01 | 0.01 | 0.01 |
| 73 | rs6767484 | G | 0.12 | 0.01 | -0.01 | 0.01 |
| 74 | rs6785040 | C | -0.06 | 0.01 | -0.01 | 0.01 |
| 75 | rs6795735 | T | -0.06 | 0.01 | 0.01 | 0.01 |
| 76 | rs6878122 | A | -0.06 | 0.01 | 0.02 | 0.02 |
| 77 | rs6960043 | C | 0.06 | 0.01 | 0.00 | 0.01 |
| 78 | rs7177055 | A | 0.06 | 0.01 | 0.00 | 0.01 |
| 79 | rs7240767 | C | 0.05 | 0.01 | 0.01 | 0.01 |
| 80 | rs72802358 | C | -0.12 | 0.01 | -0.02 | 0.02 |
| 81 | rs72892910 | T | 0.06 | 0.01 | 0.00 | 0.01 |
| 82 | rs753270 | C | 0.05 | 0.01 | 0.01 | 0.01 |
| 83 | rs7561798 | G | 0.04 | 0.01 | 0.01 | 0.01 |
| 84 | rs7572970 | G | 0.06 | 0.01 | 0.00 | 0.01 |
| 85 | rs7607777 | T | -0.14 | 0.01 | 0.00 | 0.03 |
| 86 | rs7674212 | T | -0.05 | 0.01 | 0.01 | 0.01 |
| 87 | rs7685296 | T | -0.05 | 0.01 | 0.00 | 0.01 |
| 88 | rs7756992 | G | 0.13 | 0.01 | -0.01 | 0.01 |
| 89 | rs7786095 | G | -0.07 | 0.01 | -0.02 | 0.01 |
| 90 | rs780094 | C | 0.07 | 0.01 | -0.02 | 0.01 |
| 91 | rs7845219 | C | -0.04 | 0.01 | 0.00 | 0.01 |
| 92 | rs7903146 | T | 0.31 | 0.01 | 0.01 | 0.01 |
| 93 | rs7929543 | C | 0.08 | 0.01 | -0.01 | 0.01 |
| 94 | rs7955901 | T | -0.04 | 0.01 | 0.00 | 0.01 |
| 95 | rs8068804 | A | 0.06 | 0.01 | -0.01 | 0.01 |
| 96 | rs8108269 | G | 0.06 | 0.01 | 0.02 | 0.01 |
| 97 | rs825476 | T | 0.05 | 0.01 | 0.00 | 0.01 |
| 98 | rs840967 | A | -0.05 | 0.01 | 0.00 | 0.01 |
| 99 | rs853974 | C | -0.06 | 0.01 | -0.01 | 0.01 |
| 100 | rs9369425 | A | -0.05 | 0.01 | 0.02 | 0.01 |
| 101 | rs963740 | T | -0.05 | 0.01 | 0.00 | 0.01 |
| 102 | rs9894220 | G | -0.06 | 0.01 | -0.01 | 0.01 |
| 103 | rs9928094 | G | 0.10 | 0.01 | 0.01 | 0.01 |
| 104 | rs993380 | G | -0.05 | 0.01 | 0.00 | 0.01 |
| 105 | rs9940149 | A | -0.06 | 0.01 | 0.00 | 0.01 |

T2DM: Type-2 diabetes; LDL: Low-density lipoprotein.

Table 8. Genetic association estimates for the effect of T2DM on LDL (Kettunen). ea=effect allele, gx=T2DM, gy=LDL, se=standard error

|  | SNP | ea | gx | gx_se | gy | gy_se |
| --- | --- | --- | --- | --- | --- | --- |
| 1 | rs10077431 | A | -0.05 | 0.01 | -0.03 | 0.01 |
| 2 | rs10087241 | A | -0.05 | 0.01 | -0.01 | 0.01 |
| 3 | rs10100265 | C | -0.05 | 0.01 | 0.01 | 0.01 |
| 4 | rs10114341 | C | -0.04 | 0.01 | 0.00 | 0.01 |
| 5 | rs10401969 | C | 0.09 | 0.01 | -0.09 | 0.02 |
| 6 | rs1050226 | G | -0.05 | 0.01 | 0.00 | 0.01 |
| 7 | rs1061813 | A | -0.04 | 0.01 | -0.01 | 0.01 |
| 8 | rs1063355 | G | 0.07 | 0.01 | -0.02 | 0.01 |
| 9 | rs10740322 | A | 0.05 | 0.01 | 0.00 | 0.01 |
| 10 | rs10811661 | C | -0.16 | 0.01 | 0.00 | 0.01 |
| 11 | rs10830963 | G | 0.09 | 0.01 | 0.00 | 0.01 |
| 12 | rs10842994 | T | -0.08 | 0.01 | -0.01 | 0.01 |
| 13 | rs10974438 | C | 0.06 | 0.01 | 0.02 | 0.01 |
| 14 | rs11098676 | C | 0.05 | 0.01 | 0.00 | 0.01 |
| 15 | rs11107116 | T | 0.05 | 0.01 | 0.01 | 0.01 |
| 16 | rs1111875 | T | -0.09 | 0.01 | -0.02 | 0.01 |
| 17 | rs11257655 | T | 0.07 | 0.01 | 0.01 | 0.01 |
| 18 | rs1127655 | T | -0.04 | 0.01 | -0.01 | 0.01 |
| 19 | rs11708067 | G | -0.10 | 0.01 | -0.01 | 0.01 |
| 20 | rs11925227 | A | -0.05 | 0.01 | 0.01 | 0.01 |
| 21 | rs11926707 | C | 0.05 | 0.01 | 0.00 | 0.01 |
| 22 | rs12088739 | G | -0.09 | 0.01 | 0.01 | 0.02 |
| 23 | rs12299509 | G | 0.05 | 0.01 | 0.01 | 0.01 |
| 24 | rs12617659 | T | -0.07 | 0.01 | 0.01 | 0.01 |
| 25 | rs12910825 | G | 0.05 | 0.01 | -0.01 | 0.01 |
| 26 | rs12945601 | C | -0.05 | 0.01 | 0.01 | 0.01 |
| 27 | rs12970134 | A | 0.06 | 0.01 | 0.02 | 0.01 |
| 28 | rs13234269 | A | -0.06 | 0.01 | -0.01 | 0.01 |
| 29 | rs13239186 | T | 0.05 | 0.01 | -0.01 | 0.01 |
| 30 | rs13330951 | G | -0.05 | 0.01 | 0.00 | 0.01 |
| 31 | rs13389219 | T | -0.07 | 0.01 | -0.01 | 0.01 |
| 32 | rs1359790 | A | -0.08 | 0.01 | 0.02 | 0.01 |
| 33 | rs1496653 | G | -0.08 | 0.01 | 0.00 | 0.01 |
| 34 | rs1552224 | C | -0.10 | 0.01 | -0.01 | 0.01 |
| 35 | rs16988333 | G | -0.07 | 0.01 | 0.01 | 0.02 |
| 36 | rs17086692 | T | -0.05 | 0.01 | 0.01 | 0.01 |
| 37 | rs17168486 | T | 0.07 | 0.01 | 0.01 | 0.01 |
| 38 | rs17405722 | A | 0.09 | 0.01 | 0.01 | 0.02 |
| 39 | rs17411031 | G | -0.05 | 0.01 | -0.02 | 0.01 |
| 40 | rs1758632 | G | 0.05 | 0.01 | -0.01 | 0.01 |
| 41 | rs17631783 | T | -0.05 | 0.01 | 0.00 | 0.01 |
| 42 | rs17791513 | G | -0.10 | 0.01 | 0.00 | 0.02 |
| 43 | rs1801214 | T | 0.09 | 0.01 | 0.02 | 0.01 |
| 44 | rs1899951 | T | -0.11 | 0.01 | -0.01 | 0.01 |
| 45 | rs2058913 | T | -0.05 | 0.01 | 0.00 | 0.01 |
| 46 | rs2237892 | T | -0.10 | 0.02 | -0.01 | 0.02 |
| 47 | rs2246618 | T | 0.05 | 0.01 | 0.01 | 0.01 |
| 48 | rs2261181 | T | 0.10 | 0.01 | 0.00 | 0.02 |
| 49 | rs2294120 | G | -0.04 | 0.01 | -0.01 | 0.01 |
| 50 | rs2296173 | G | 0.07 | 0.01 | 0.01 | 0.01 |
| 51 | rs2299383 | T | 0.04 | 0.01 | 0.00 | 0.01 |
| 52 | rs243019 | C | 0.06 | 0.01 | 0.00 | 0.01 |
| 53 | rs2493394 | G | 0.07 | 0.01 | 0.03 | 0.01 |
| 54 | rs2796441 | A | -0.07 | 0.01 | 0.01 | 0.01 |
| 55 | rs2820426 | G | 0.05 | 0.01 | 0.02 | 0.01 |
| 56 | rs2867125 | C | 0.06 | 0.01 | 0.01 | 0.01 |
| 57 | rs2908282 | A | 0.06 | 0.01 | -0.02 | 0.01 |
| 58 | rs2925979 | C | -0.05 | 0.01 | 0.00 | 0.01 |
| 59 | rs2943656 | G | 0.09 | 0.01 | 0.00 | 0.01 |
| 60 | rs3217992 | T | 0.05 | 0.01 | 0.00 | 0.01 |
| 61 | rs340874 | C | 0.06 | 0.01 | 0.01 | 0.01 |
| 62 | rs348330 | A | -0.05 | 0.01 | 0.00 | 0.01 |
| 63 | rs3756784 | G | 0.05 | 0.01 | -0.02 | 0.01 |
| 64 | rs3802177 | A | -0.12 | 0.01 | 0.00 | 0.01 |
| 65 | rs459193 | G | 0.07 | 0.01 | -0.01 | 0.01 |
| 66 | rs4622883 | G | -0.04 | 0.01 | 0.01 | 0.01 |
| 67 | rs4686471 | C | 0.05 | 0.01 | -0.01 | 0.01 |
| 68 | rs4812829 | A | 0.05 | 0.01 | -0.01 | 0.01 |
| 69 | rs4823182 | G | 0.05 | 0.01 | 0.00 | 0.01 |
| 70 | rs4865796 | A | 0.05 | 0.01 | 0.01 | 0.01 |
| 71 | rs516946 | C | 0.08 | 0.01 | 0.00 | 0.01 |
| 72 | rs5215 | T | -0.07 | 0.01 | -0.01 | 0.01 |
| 73 | rs55966194 | G | -0.05 | 0.01 | 0.00 | 0.01 |
| 74 | rs576674 | A | -0.07 | 0.01 | 0.01 | 0.02 |
| 75 | rs6059662 | G | 0.04 | 0.01 | 0.00 | 0.01 |
| 76 | rs61953351 | T | -0.07 | 0.01 | -0.02 | 0.01 |
| 77 | rs622217 | C | -0.05 | 0.01 | -0.01 | 0.01 |
| 78 | rs6494307 | G | -0.04 | 0.01 | 0.01 | 0.01 |
| 79 | rs6515236 | C | -0.05 | 0.01 | 0.00 | 0.01 |
| 80 | rs67232546 | T | 0.06 | 0.01 | 0.02 | 0.01 |
| 81 | rs6767484 | G | 0.12 | 0.01 | 0.00 | 0.01 |
| 82 | rs6785040 | C | -0.06 | 0.01 | 0.00 | 0.01 |
| 83 | rs6795735 | T | -0.06 | 0.01 | 0.00 | 0.01 |
| 84 | rs6878122 | A | -0.06 | 0.01 | -0.02 | 0.01 |
| 85 | rs6960043 | C | 0.06 | 0.01 | -0.01 | 0.01 |
| 86 | rs7144011 | T | 0.05 | 0.01 | 0.00 | 0.01 |
| 87 | rs7177055 | A | 0.06 | 0.01 | 0.00 | 0.01 |
| 88 | rs7240767 | C | 0.05 | 0.01 | 0.01 | 0.01 |
| 89 | rs72802358 | C | -0.12 | 0.01 | 0.01 | 0.02 |
| 90 | rs72892910 | T | 0.06 | 0.01 | 0.02 | 0.01 |
| 91 | rs735949 | C | -0.07 | 0.01 | 0.01 | 0.02 |
| 92 | rs753270 | C | 0.05 | 0.01 | 0.02 | 0.01 |
| 93 | rs7561798 | G | 0.04 | 0.01 | 0.00 | 0.01 |
| 94 | rs7572970 | G | 0.06 | 0.01 | 0.00 | 0.01 |
| 95 | rs7607777 | T | -0.14 | 0.01 | -0.05 | 0.02 |
| 96 | rs7674212 | T | -0.05 | 0.01 | -0.01 | 0.01 |
| 97 | rs7685296 | T | -0.05 | 0.01 | 0.00 | 0.01 |
| 98 | rs7729395 | T | 0.14 | 0.02 | -0.02 | 0.02 |
| 99 | rs7756992 | G | 0.13 | 0.01 | -0.01 | 0.01 |
| 100 | rs7786095 | G | -0.07 | 0.01 | -0.03 | 0.02 |
| 101 | rs780094 | C | 0.07 | 0.01 | -0.04 | 0.01 |
| 102 | rs7845219 | C | -0.04 | 0.01 | 0.00 | 0.01 |
| 103 | rs7903146 | T | 0.31 | 0.01 | 0.00 | 0.01 |
| 104 | rs7929543 | C | 0.08 | 0.01 | 0.00 | 0.02 |
| 105 | rs7955901 | T | -0.04 | 0.01 | 0.01 | 0.01 |
| 106 | rs8068804 | A | 0.06 | 0.01 | 0.00 | 0.01 |
| 107 | rs8108269 | G | 0.06 | 0.01 | 0.02 | 0.01 |
| 108 | rs825476 | T | 0.05 | 0.01 | 0.02 | 0.01 |
| 109 | rs840967 | A | -0.05 | 0.01 | 0.00 | 0.01 |
| 110 | rs849135 | A | -0.10 | 0.01 | 0.00 | 0.01 |
| 111 | rs853974 | C | -0.06 | 0.01 | 0.02 | 0.01 |
| 112 | rs9369425 | A | -0.05 | 0.01 | 0.01 | 0.01 |
| 113 | rs963740 | T | -0.05 | 0.01 | -0.01 | 0.01 |
| 114 | rs9844972 | C | 0.10 | 0.01 | -0.01 | 0.02 |
| 115 | rs9894220 | G | -0.06 | 0.01 | 0.00 | 0.01 |
| 116 | rs9928094 | G | 0.10 | 0.01 | 0.02 | 0.01 |
| 117 | rs993380 | G | -0.05 | 0.01 | 0.00 | 0.01 |
| 118 | rs9940149 | A | -0.06 | 0.01 | 0.03 | 0.02 |

T2DM: Type-2 diabetes; LDL: Low-density lipoprotein.

Table 9. Genetic association estimates for the effect of T2DM on TC (Willer). ea=effect allele, gx=T2DM, gy=TC, se=standard error

|  | SNP | ea | gx | gx_se | gy | gy_se |
| --- | --- | --- | --- | --- | --- | --- |
| 1 | rs10077431 | A | -0.05 | 0.01 | 0.01 | 0.00 |
| 2 | rs10100265 | C | -0.05 | 0.01 | 0.00 | 0.00 |
| 3 | rs10114341 | C | -0.04 | 0.01 | 0.00 | 0.00 |
| 4 | rs10401969 | C | 0.09 | 0.01 | -0.14 | 0.01 |
| 5 | rs1050226 | G | -0.05 | 0.01 | 0.00 | 0.00 |
| 6 | rs1061813 | A | -0.04 | 0.01 | 0.00 | 0.00 |
| 7 | rs1063355 | G | 0.07 | 0.01 | -0.02 | 0.01 |
| 8 | rs10740322 | A | 0.05 | 0.01 | 0.00 | 0.01 |
| 9 | rs10811661 | C | -0.16 | 0.01 | 0.00 | 0.00 |
| 10 | rs10830963 | G | 0.09 | 0.01 | 0.01 | 0.00 |
| 11 | rs10842994 | T | -0.08 | 0.01 | 0.00 | 0.00 |
| 12 | rs10974438 | C | 0.06 | 0.01 | 0.01 | 0.00 |
| 13 | rs11098676 | C | 0.05 | 0.01 | 0.00 | 0.01 |
| 14 | rs11107116 | T | 0.05 | 0.01 | -0.01 | 0.00 |
| 15 | rs1111875 | T | -0.09 | 0.01 | -0.01 | 0.00 |
| 16 | rs11257655 | T | 0.07 | 0.01 | 0.00 | 0.00 |
| 17 | rs1127655 | T | -0.04 | 0.01 | 0.00 | 0.01 |
| 18 | rs11708067 | G | -0.10 | 0.01 | -0.02 | 0.00 |
| 19 | rs11925227 | A | -0.05 | 0.01 | -0.01 | 0.00 |
| 20 | rs11926707 | C | 0.05 | 0.01 | 0.01 | 0.01 |
| 21 | rs12088739 | G | -0.09 | 0.01 | -0.01 | 0.01 |
| 22 | rs12299509 | G | 0.05 | 0.01 | 0.00 | 0.00 |
| 23 | rs12617659 | T | -0.07 | 0.01 | 0.01 | 0.01 |
| 24 | rs12910825 | G | 0.05 | 0.01 | 0.00 | 0.00 |
| 25 | rs12945601 | C | -0.05 | 0.01 | 0.00 | 0.01 |
| 26 | rs12970134 | A | 0.06 | 0.01 | -0.01 | 0.00 |
| 27 | rs13234269 | A | -0.06 | 0.01 | -0.01 | 0.01 |
| 28 | rs13239186 | T | 0.05 | 0.01 | 0.01 | 0.01 |
| 29 | rs13330951 | G | -0.05 | 0.01 | 0.00 | 0.01 |
| 30 | rs13389219 | T | -0.07 | 0.01 | -0.02 | 0.00 |
| 31 | rs1359790 | A | -0.08 | 0.01 | 0.00 | 0.00 |
| 32 | rs1496653 | G | -0.08 | 0.01 | 0.00 | 0.00 |
| 33 | rs1552224 | C | -0.10 | 0.01 | 0.00 | 0.00 |
| 34 | rs16988333 | G | -0.07 | 0.01 | -0.01 | 0.01 |
| 35 | rs17086692 | T | -0.05 | 0.01 | 0.00 | 0.01 |
| 36 | rs17168486 | T | 0.07 | 0.01 | 0.00 | 0.00 |
| 37 | rs17405722 | A | 0.09 | 0.01 | 0.00 | 0.01 |
| 38 | rs17411031 | G | -0.05 | 0.01 | 0.00 | 0.00 |
| 39 | rs1758632 | G | 0.05 | 0.01 | 0.00 | 0.01 |
| 40 | rs17631783 | T | -0.05 | 0.01 | -0.01 | 0.01 |
| 41 | rs17791513 | G | -0.10 | 0.01 | 0.00 | 0.01 |
| 42 | rs1801214 | T | 0.09 | 0.01 | 0.00 | 0.00 |
| 43 | rs1899951 | T | -0.11 | 0.01 | 0.00 | 0.01 |
| 44 | rs2058913 | T | -0.05 | 0.01 | 0.00 | 0.01 |
| 45 | rs2237892 | T | -0.10 | 0.02 | 0.00 | 0.01 |
| 46 | rs2246618 | T | 0.05 | 0.01 | -0.01 | 0.00 |
| 47 | rs2261181 | T | 0.10 | 0.01 | 0.01 | 0.01 |
| 48 | rs2294120 | G | -0.04 | 0.01 | 0.00 | 0.00 |
| 49 | rs2296173 | G | 0.07 | 0.01 | 0.00 | 0.00 |
| 50 | rs2299383 | T | 0.04 | 0.01 | 0.00 | 0.00 |
| 51 | rs243019 | C | 0.06 | 0.01 | 0.00 | 0.00 |
| 52 | rs2493394 | G | 0.07 | 0.01 | 0.01 | 0.01 |
| 53 | rs2796441 | A | -0.07 | 0.01 | 0.00 | 0.00 |
| 54 | rs2820426 | G | 0.05 | 0.01 | 0.01 | 0.00 |
| 55 | rs2867125 | C | 0.06 | 0.01 | -0.01 | 0.00 |
| 56 | rs2908282 | A | 0.06 | 0.01 | 0.00 | 0.00 |
| 57 | rs2925979 | C | -0.05 | 0.01 | 0.01 | 0.00 |
| 58 | rs2943656 | G | 0.09 | 0.01 | 0.00 | 0.00 |
| 59 | rs3217992 | T | 0.05 | 0.01 | 0.00 | 0.00 |
| 60 | rs340874 | C | 0.06 | 0.01 | 0.01 | 0.00 |
| 61 | rs3756784 | G | 0.05 | 0.01 | -0.01 | 0.00 |
| 62 | rs3802177 | A | -0.12 | 0.01 | -0.01 | 0.00 |
| 63 | rs459193 | G | 0.07 | 0.01 | -0.01 | 0.00 |
| 64 | rs4622883 | G | -0.04 | 0.01 | 0.01 | 0.01 |
| 65 | rs4812829 | A | 0.05 | 0.01 | 0.00 | 0.00 |
| 66 | rs4823182 | G | 0.05 | 0.01 | -0.02 | 0.00 |
| 67 | rs4865796 | A | 0.05 | 0.01 | 0.00 | 0.00 |
| 68 | rs516946 | C | 0.08 | 0.01 | 0.00 | 0.00 |
| 69 | rs5215 | T | -0.07 | 0.01 | -0.01 | 0.00 |
| 70 | rs55966194 | G | -0.05 | 0.01 | -0.02 | 0.01 |
| 71 | rs576674 | A | -0.07 | 0.01 | 0.00 | 0.01 |
| 72 | rs6059662 | G | 0.04 | 0.01 | -0.01 | 0.00 |
| 73 | rs622217 | C | -0.05 | 0.01 | -0.02 | 0.00 |
| 74 | rs6494307 | G | -0.04 | 0.01 | 0.00 | 0.01 |
| 75 | rs6515236 | C | -0.05 | 0.01 | 0.01 | 0.01 |
| 76 | rs6767484 | G | 0.12 | 0.01 | -0.01 | 0.00 |
| 77 | rs6785040 | C | -0.06 | 0.01 | 0.00 | 0.01 |
| 78 | rs6795735 | T | -0.06 | 0.01 | -0.01 | 0.00 |
| 79 | rs6878122 | A | -0.06 | 0.01 | 0.00 | 0.00 |
| 80 | rs6960043 | C | 0.06 | 0.01 | 0.00 | 0.00 |
| 81 | rs7144011 | T | 0.05 | 0.01 | 0.00 | 0.00 |
| 82 | rs7177055 | A | 0.06 | 0.01 | 0.00 | 0.00 |
| 83 | rs7240767 | C | 0.05 | 0.01 | 0.00 | 0.01 |
| 84 | rs72802358 | C | -0.12 | 0.01 | 0.00 | 0.01 |
| 85 | rs72892910 | T | 0.06 | 0.01 | 0.00 | 0.01 |
| 86 | rs735949 | C | -0.07 | 0.01 | 0.01 | 0.01 |
| 87 | rs753270 | C | 0.05 | 0.01 | 0.01 | 0.01 |
| 88 | rs7561798 | G | 0.04 | 0.01 | 0.00 | 0.00 |
| 89 | rs7572970 | G | 0.06 | 0.01 | 0.00 | 0.01 |
| 90 | rs7607777 | T | -0.14 | 0.01 | -0.01 | 0.01 |
| 91 | rs7685296 | T | -0.05 | 0.01 | 0.00 | 0.00 |
| 92 | rs7729395 | T | 0.14 | 0.02 | 0.01 | 0.01 |
| 93 | rs7756992 | G | 0.13 | 0.01 | 0.00 | 0.00 |
| 94 | rs7786095 | G | -0.07 | 0.01 | -0.01 | 0.01 |
| 95 | rs780094 | C | 0.07 | 0.01 | -0.05 | 0.00 |
| 96 | rs7845219 | C | -0.04 | 0.01 | 0.00 | 0.00 |
| 97 | rs7903146 | T | 0.31 | 0.01 | 0.01 | 0.00 |
| 98 | rs7929543 | C | 0.08 | 0.01 | 0.00 | 0.01 |
| 99 | rs7955901 | T | -0.04 | 0.01 | 0.00 | 0.00 |
| 100 | rs8068804 | A | 0.06 | 0.01 | 0.01 | 0.00 |
| 101 | rs8108269 | G | 0.06 | 0.01 | -0.02 | 0.00 |
| 102 | rs825476 | T | 0.05 | 0.01 | -0.01 | 0.00 |
| 103 | rs840967 | A | -0.05 | 0.01 | -0.01 | 0.01 |
| 104 | rs849135 | A | -0.10 | 0.01 | 0.00 | 0.00 |
| 105 | rs9369425 | A | -0.05 | 0.01 | 0.01 | 0.01 |
| 106 | rs963740 | T | -0.05 | 0.01 | 0.01 | 0.01 |
| 107 | rs9844972 | C | 0.10 | 0.01 | 0.01 | 0.01 |
| 108 | rs9894220 | G | -0.06 | 0.01 | 0.00 | 0.01 |
| 109 | rs9928094 | G | 0.10 | 0.01 | -0.01 | 0.00 |
| 110 | rs993380 | G | -0.05 | 0.01 | 0.00 | 0.01 |
| 111 | rs9940149 | A | -0.06 | 0.01 | 0.00 | 0.00 |

T2DM: Type-2 diabetes; TC: Total cholesterol.

Table 10. Genetic association estimates for the effect of T2DM on TC (Kettunen). ea=effect allele, gx=T2DM, gy=TC, se=standard error

|  | SNP | ea | gx | gx_se | gy | gy_se |
| --- | --- | --- | --- | --- | --- | --- |
| 1 | rs10077431 | A | -0.05 | -0.02 | 0.01 | 0.01 |
| 2 | rs10087241 | A | -0.05 | -0.02 | 0.01 | 0.01 |
| 3 | rs10100265 | C | -0.05 | 0.01 | 0.01 | 0.01 |
| 4 | rs10114341 | C | -0.04 | 0.00 | 0.01 | 0.01 |
| 5 | rs10401969 | C | 0.09 | -0.12 | 0.02 | 0.01 |
| 6 | rs1050226 | G | -0.05 | 0.00 | 0.01 | 0.01 |
| 7 | rs1061813 | A | -0.04 | 0.00 | 0.01 | 0.01 |
| 8 | rs1063355 | G | 0.07 | -0.02 | 0.01 | 0.01 |
| 9 | rs10740322 | A | 0.05 | 0.01 | 0.01 | 0.01 |
| 10 | rs10811661 | C | -0.16 | 0.00 | 0.01 | 0.01 |
| 11 | rs10830963 | G | 0.09 | 0.00 | 0.01 | 0.01 |
| 12 | rs10842994 | T | -0.08 | 0.00 | 0.01 | 0.01 |
| 13 | rs10974438 | C | 0.06 | 0.02 | 0.01 | 0.01 |
| 14 | rs11098676 | C | 0.05 | 0.01 | 0.01 | 0.01 |
| 15 | rs11107116 | T | 0.05 | 0.01 | 0.01 | 0.01 |
| 16 | rs1111875 | T | -0.09 | -0.01 | 0.01 | 0.01 |
| 17 | rs11257655 | T | 0.07 | 0.01 | 0.01 | 0.01 |
| 18 | rs1127655 | T | -0.04 | -0.01 | 0.01 | 0.01 |
| 19 | rs11708067 | G | -0.10 | -0.01 | 0.01 | 0.01 |
| 20 | rs11925227 | A | -0.05 | 0.00 | 0.01 | 0.01 |
| 21 | rs11926707 | C | 0.05 | 0.01 | 0.01 | 0.01 |
| 22 | rs12088739 | G | -0.09 | 0.02 | 0.02 | 0.01 |
| 23 | rs12299509 | G | 0.05 | 0.00 | 0.01 | 0.01 |
| 24 | rs12617659 | T | -0.07 | 0.00 | 0.01 | 0.01 |
| 25 | rs12910825 | G | 0.05 | -0.01 | 0.01 | 0.01 |
| 26 | rs12945601 | C | -0.05 | 0.01 | 0.01 | 0.01 |
| 27 | rs12970134 | A | 0.06 | 0.02 | 0.01 | 0.01 |
| 28 | rs13234269 | A | -0.06 | 0.00 | 0.01 | 0.01 |
| 29 | rs13239186 | T | 0.05 | -0.01 | 0.01 | 0.01 |
| 30 | rs13330951 | G | -0.05 | 0.00 | 0.01 | 0.01 |
| 31 | rs13389219 | T | -0.07 | -0.01 | 0.01 | 0.01 |
| 32 | rs1359790 | A | -0.08 | 0.03 | 0.01 | 0.01 |
| 33 | rs1496653 | G | -0.08 | 0.01 | 0.01 | 0.01 |
| 34 | rs1552224 | C | -0.10 | 0.00 | 0.01 | 0.01 |
| 35 | rs16988333 | G | -0.07 | 0.00 | 0.02 | 0.01 |
| 36 | rs17086692 | T | -0.05 | 0.00 | 0.01 | 0.01 |
| 37 | rs17168486 | T | 0.07 | 0.01 | 0.01 | 0.01 |
| 38 | rs17405722 | A | 0.09 | 0.00 | 0.02 | 0.01 |
| 39 | rs17411031 | G | -0.05 | -0.02 | 0.01 | 0.01 |
| 40 | rs1758632 | G | 0.05 | -0.01 | 0.01 | 0.01 |
| 41 | rs17631783 | T | -0.05 | -0.01 | 0.01 | 0.01 |
| 42 | rs17791513 | G | -0.10 | 0.00 | 0.02 | 0.01 |
| 43 | rs1801214 | T | 0.09 | 0.02 | 0.01 | 0.01 |
| 44 | rs1899951 | T | -0.11 | -0.01 | 0.01 | 0.01 |
| 45 | rs2058913 | T | -0.05 | 0.00 | 0.01 | 0.01 |
| 46 | rs2237892 | T | -0.10 | -0.01 | 0.02 | 0.02 |
| 47 | rs2246618 | T | 0.05 | 0.01 | 0.01 | 0.01 |
| 48 | rs2261181 | T | 0.10 | 0.01 | 0.02 | 0.01 |
| 49 | rs2294120 | G | -0.04 | 0.01 | 0.01 | 0.01 |
| 50 | rs2296173 | G | 0.07 | 0.00 | 0.01 | 0.01 |
| 51 | rs2299383 | T | 0.04 | 0.00 | 0.01 | 0.01 |
| 52 | rs243019 | C | 0.06 | -0.02 | 0.01 | 0.01 |
| 53 | rs2493394 | G | 0.07 | 0.02 | 0.01 | 0.01 |
| 54 | rs2796441 | A | -0.07 | 0.01 | 0.01 | 0.01 |
| 55 | rs2820426 | G | 0.05 | 0.01 | 0.01 | 0.01 |
| 56 | rs2867125 | C | 0.06 | 0.00 | 0.01 | 0.01 |
| 57 | rs2908282 | A | 0.06 | -0.01 | 0.02 | 0.01 |
| 58 | rs2925979 | C | -0.05 | 0.00 | 0.01 | 0.01 |
| 59 | rs2943656 | G | 0.09 | 0.00 | 0.01 | 0.01 |
| 60 | rs3217992 | T | 0.05 | -0.01 | 0.01 | 0.01 |
| 61 | rs340874 | C | 0.06 | 0.01 | 0.01 | 0.01 |
| 62 | rs348330 | A | -0.05 | 0.00 | 0.01 | 0.01 |
| 63 | rs3756784 | G | 0.05 | -0.02 | 0.01 | 0.01 |
| 64 | rs3802177 | A | -0.12 | -0.01 | 0.01 | 0.01 |
| 65 | rs459193 | G | 0.07 | -0.02 | 0.01 | 0.01 |
| 66 | rs4622883 | G | -0.04 | 0.00 | 0.01 | 0.01 |
| 67 | rs4686471 | C | 0.05 | -0.01 | 0.01 | 0.01 |
| 68 | rs4812829 | A | 0.05 | 0.00 | 0.01 | 0.01 |
| 69 | rs4823182 | G | 0.05 | 0.00 | 0.01 | 0.01 |
| 70 | rs4865796 | A | 0.05 | 0.01 | 0.01 | 0.01 |
| 71 | rs516946 | C | 0.08 | -0.01 | 0.01 | 0.01 |
| 72 | rs5215 | T | -0.07 | -0.01 | 0.01 | 0.01 |
| 73 | rs55966194 | G | -0.05 | -0.01 | 0.01 | 0.01 |
| 74 | rs576674 | A | -0.07 | 0.00 | 0.02 | 0.01 |
| 75 | rs6059662 | G | 0.04 | -0.01 | 0.01 | 0.01 |
| 76 | rs61953351 | T | -0.07 | -0.03 | 0.01 | 0.01 |
| 77 | rs622217 | C | -0.05 | -0.01 | 0.01 | 0.01 |
| 78 | rs6494307 | G | -0.04 | 0.01 | 0.01 | 0.01 |
| 79 | rs6515236 | C | -0.05 | 0.00 | 0.01 | 0.01 |
| 80 | rs67232546 | T | 0.06 | 0.03 | 0.01 | 0.01 |
| 81 | rs6767484 | G | 0.12 | 0.00 | 0.01 | 0.01 |
| 82 | rs6785040 | C | -0.06 | 0.00 | 0.01 | 0.01 |
| 83 | rs6795735 | T | -0.06 | 0.01 | 0.01 | 0.01 |
| 84 | rs6878122 | A | -0.06 | -0.02 | 0.01 | 0.01 |
| 85 | rs6960043 | C | 0.06 | 0.00 | 0.01 | 0.01 |
| 86 | rs7144011 | T | 0.05 | -0.01 | 0.01 | 0.01 |
| 87 | rs7177055 | A | 0.06 | 0.00 | 0.01 | 0.01 |
| 88 | rs7240767 | C | 0.05 | 0.01 | 0.01 | 0.01 |
| 89 | rs72802358 | C | -0.12 | 0.02 | 0.02 | 0.01 |
| 90 | rs72892910 | T | 0.06 | 0.02 | 0.01 | 0.01 |
| 91 | rs735949 | C | -0.07 | 0.01 | 0.02 | 0.01 |
| 92 | rs753270 | C | 0.05 | 0.01 | 0.01 | 0.01 |
| 93 | rs7561798 | G | 0.04 | 0.00 | 0.01 | 0.01 |
| 94 | rs7572970 | G | 0.06 | 0.02 | 0.01 | 0.01 |
| 95 | rs7607777 | T | -0.14 | -0.05 | 0.02 | 0.01 |
| 96 | rs7674212 | T | -0.05 | -0.01 | 0.01 | 0.01 |
| 97 | rs7685296 | T | -0.05 | 0.01 | 0.01 | 0.01 |
| 98 | rs7729395 | T | 0.14 | -0.01 | 0.03 | 0.02 |
| 99 | rs7756992 | G | 0.13 | -0.01 | 0.01 | 0.01 |
| 100 | rs7786095 | G | -0.07 | 0.00 | 0.02 | 0.01 |
| 101 | rs780094 | C | 0.07 | -0.06 | 0.01 | 0.01 |
| 102 | rs7845219 | C | -0.04 | 0.00 | 0.01 | 0.01 |
| 103 | rs7903146 | T | 0.31 | 0.00 | 0.01 | 0.01 |
| 104 | rs7929543 | C | 0.08 | -0.01 | 0.02 | 0.01 |
| 105 | rs7955901 | T | -0.04 | 0.02 | 0.01 | 0.01 |
| 106 | rs8068804 | A | 0.06 | 0.00 | 0.01 | 0.01 |
| 107 | rs8108269 | G | 0.06 | 0.02 | 0.01 | 0.01 |
| 108 | rs825476 | T | 0.05 | 0.01 | 0.01 | 0.01 |
| 109 | rs840967 | A | -0.05 | 0.00 | 0.01 | 0.01 |
| 110 | rs849135 | A | -0.10 | 0.00 | 0.01 | 0.01 |
| 111 | rs853974 | C | -0.06 | 0.02 | 0.01 | 0.01 |
| 112 | rs9369425 | A | -0.05 | 0.00 | 0.01 | 0.01 |
| 113 | rs963740 | T | -0.05 | 0.00 | 0.01 | 0.01 |
| 114 | rs9844972 | C | 0.10 | 0.00 | 0.02 | 0.01 |
| 115 | rs9894220 | G | -0.06 | 0.01 | 0.01 | 0.01 |
| 116 | rs9928094 | G | 0.10 | 0.02 | 0.01 | 0.01 |
| 117 | rs993380 | G | -0.05 | 0.00 | 0.01 | 0.01 |
| 118 | rs9940149 | A | -0.06 | 0.03 | 0.02 | 0.01 |

T2DM: Type-2 diabetes; TC: Total cholesterol.

Table 11. Genetic association estimates for the effect of T2DM on TC (Prins). ea=effect allele, gx=T2DM, gy=TC, se=standard error

|  | SNP | ea | gx | gx_se | gy | gy_se |
| --- | --- | --- | --- | --- | --- | --- |
| 1 | rs10100265 | C | -0.05 | 0.01 | -0.02 | 0.02 |
| 2 | rs10114341 | C | -0.04 | 0.01 | -0.01 | 0.01 |
| 3 | rs10401969 | C | 0.09 | 0.01 | -0.14 | 0.03 |
| 4 | rs1050226 | G | -0.05 | 0.01 | -0.01 | 0.01 |
| 5 | rs1061813 | A | -0.04 | 0.01 | 0.01 | 0.01 |
| 6 | rs1063355 | G | 0.07 | 0.01 | -0.01 | 0.02 |
| 7 | rs10740322 | A | 0.05 | 0.01 | -0.03 | 0.02 |
| 8 | rs10811661 | C | -0.16 | 0.01 | 0.06 | 0.02 |
| 9 | rs10830963 | G | 0.09 | 0.01 | -0.01 | 0.02 |
| 10 | rs10842994 | T | -0.08 | 0.01 | 0.01 | 0.02 |
| 11 | rs10974438 | C | 0.06 | 0.01 | -0.01 | 0.02 |
| 12 | rs11098676 | C | 0.05 | 0.01 | -0.01 | 0.02 |
| 13 | rs11107116 | T | 0.05 | 0.01 | 0.02 | 0.02 |
| 14 | rs1127655 | T | -0.04 | 0.01 | 0.02 | 0.01 |
| 15 | rs11708067 | G | -0.10 | 0.01 | -0.02 | 0.02 |
| 16 | rs12299509 | G | 0.05 | 0.01 | 0.01 | 0.02 |
| 17 | rs12617659 | T | -0.07 | 0.01 | 0.01 | 0.02 |
| 18 | rs12910825 | G | 0.05 | 0.01 | 0.05 | 0.02 |
| 19 | rs12945601 | C | -0.05 | 0.01 | 0.01 | 0.02 |
| 20 | rs12970134 | A | 0.06 | 0.01 | 0.01 | 0.02 |
| 21 | rs13234269 | A | -0.06 | 0.01 | 0.02 | 0.01 |
| 22 | rs13239186 | T | 0.05 | 0.01 | -0.01 | 0.02 |
| 23 | rs13330951 | G | -0.05 | 0.01 | 0.01 | 0.01 |
| 24 | rs13389219 | T | -0.07 | 0.01 | 0.02 | 0.02 |
| 25 | rs1359790 | A | -0.08 | 0.01 | 0.02 | 0.02 |
| 26 | rs1496653 | G | -0.08 | 0.01 | -0.01 | 0.02 |
| 27 | rs1552224 | C | -0.10 | 0.01 | -0.02 | 0.02 |
| 28 | rs16988333 | G | -0.07 | 0.01 | 0.02 | 0.02 |
| 29 | rs17086692 | T | -0.05 | 0.01 | 0.03 | 0.02 |
| 30 | rs17168486 | T | 0.07 | 0.01 | 0.01 | 0.02 |
| 31 | rs17405722 | A | 0.09 | 0.01 | 0.01 | 0.03 |
| 32 | rs17411031 | G | -0.05 | 0.01 | -0.01 | 0.02 |
| 33 | rs1758632 | G | 0.05 | 0.01 | -0.02 | 0.01 |
| 34 | rs17631783 | T | -0.05 | 0.01 | 0.01 | 0.02 |
| 35 | rs17791513 | G | -0.10 | 0.01 | -0.06 | 0.03 |
| 36 | rs1801214 | T | 0.09 | 0.01 | 0.01 | 0.01 |
| 37 | rs1899951 | T | -0.11 | 0.01 | 0.01 | 0.02 |
| 38 | rs2058913 | T | -0.05 | 0.01 | -0.01 | 0.01 |
| 39 | rs2237892 | T | -0.10 | 0.02 | 0.04 | 0.03 |
| 40 | rs2246618 | T | 0.05 | 0.01 | -0.02 | 0.02 |
| 41 | rs2261181 | T | 0.10 | 0.01 | -0.01 | 0.02 |
| 42 | rs2294120 | G | -0.04 | 0.01 | -0.01 | 0.01 |
| 43 | rs2296173 | G | 0.07 | 0.01 | -0.04 | 0.02 |
| 44 | rs2299383 | T | 0.04 | 0.01 | 0.01 | 0.01 |
| 45 | rs243019 | C | 0.06 | 0.01 | -0.01 | 0.01 |
| 46 | rs2493394 | G | 0.07 | 0.01 | -0.01 | 0.02 |
| 47 | rs2796441 | A | -0.07 | 0.01 | 0.02 | 0.01 |
| 48 | rs2820426 | G | 0.05 | 0.01 | 0.01 | 0.02 |
| 49 | rs2867125 | C | 0.06 | 0.01 | -0.01 | 0.02 |
| 50 | rs2925979 | C | -0.05 | 0.01 | -0.01 | 0.02 |
| 51 | rs2943656 | G | 0.09 | 0.01 | -0.01 | 0.02 |
| 52 | rs3217992 | T | 0.05 | 0.01 | 0.01 | 0.02 |
| 53 | rs3756784 | G | 0.05 | 0.01 | -0.02 | 0.02 |
| 54 | rs459193 | G | 0.07 | 0.01 | 0.01 | 0.02 |
| 55 | rs4622883 | G | -0.04 | 0.01 | -0.01 | 0.01 |
| 56 | rs4823182 | G | 0.05 | 0.01 | -0.01 | 0.02 |
| 57 | rs516946 | C | 0.08 | 0.01 | -0.01 | 0.02 |
| 58 | rs5215 | T | -0.07 | 0.01 | -0.03 | 0.02 |
| 59 | rs576674 | A | -0.07 | 0.01 | -0.01 | 0.02 |
| 60 | rs6059662 | G | 0.04 | 0.01 | -0.02 | 0.02 |
| 61 | rs61953351 | T | -0.07 | 0.01 | -0.02 | 0.02 |
| 62 | rs6494307 | G | -0.04 | 0.01 | 0.02 | 0.01 |
| 63 | rs67232546 | T | 0.06 | 0.01 | 0.01 | 0.02 |
| 64 | rs6767484 | G | 0.12 | 0.01 | 0.01 | 0.02 |
| 65 | rs6785040 | C | -0.06 | 0.01 | -0.01 | 0.02 |
| 66 | rs6795735 | T | -0.06 | 0.01 | 0.02 | 0.01 |
| 67 | rs6960043 | C | 0.06 | 0.01 | -0.01 | 0.01 |
| 68 | rs7144011 | T | 0.05 | 0.01 | -0.02 | 0.02 |
| 69 | rs7177055 | A | 0.06 | 0.01 | 0.01 | 0.02 |
| 70 | rs7240767 | C | 0.05 | 0.01 | -0.01 | 0.01 |
| 71 | rs72802358 | C | -0.12 | 0.01 | 0.01 | 0.02 |
| 72 | rs72892910 | T | 0.06 | 0.01 | 0.03 | 0.02 |
| 73 | rs735949 | C | -0.07 | 0.01 | 0.03 | 0.02 |
| 74 | rs753270 | C | 0.05 | 0.01 | 0.01 | 0.01 |
| 75 | rs7561798 | G | 0.04 | 0.01 | 0.01 | 0.01 |
| 76 | rs7572970 | G | 0.06 | 0.01 | 0.01 | 0.02 |
| 77 | rs7607777 | T | -0.14 | 0.01 | 0.01 | 0.02 |
| 78 | rs7674212 | T | -0.05 | 0.01 | -0.01 | 0.02 |
| 79 | rs7685296 | T | -0.05 | 0.01 | 0.04 | 0.02 |
| 80 | rs7729395 | T | 0.14 | 0.02 | 0.02 | 0.03 |
| 81 | rs7756992 | G | 0.13 | 0.01 | 0.01 | 0.02 |
| 82 | rs7786095 | G | -0.07 | 0.01 | 0.05 | 0.02 |
| 83 | rs780094 | C | 0.07 | 0.01 | -0.03 | 0.02 |
| 84 | rs7845219 | C | -0.04 | 0.01 | -0.01 | 0.01 |
| 85 | rs7903146 | T | 0.31 | 0.01 | -0.03 | 0.02 |
| 86 | rs7929543 | C | 0.08 | 0.01 | -0.05 | 0.03 |
| 87 | rs7955901 | T | -0.04 | 0.01 | 0.02 | 0.02 |
| 88 | rs8068804 | A | 0.06 | 0.01 | -0.01 | 0.02 |
| 89 | rs8108269 | G | 0.06 | 0.01 | 0.02 | 0.02 |
| 90 | rs825476 | T | 0.05 | 0.01 | -0.01 | 0.01 |
| 91 | rs840967 | A | -0.05 | 0.01 | -0.01 | 0.01 |
| 92 | rs849135 | A | -0.10 | 0.01 | -0.02 | 0.01 |
| 93 | rs853974 | C | -0.06 | 0.01 | 0.02 | 0.02 |
| 94 | rs9369425 | A | -0.05 | 0.01 | -0.01 | 0.02 |
| 95 | rs9894220 | G | -0.06 | 0.01 | 0.01 | 0.01 |
| 96 | rs9928094 | G | 0.10 | 0.01 | -0.01 | 0.02 |
| 97 | rs993380 | G | -0.05 | 0.01 | -0.01 | 0.02 |
| 98 | rs9940149 | A | -0.06 | 0.01 | -0.01 | 0.02 |

T2DM: Type-2 diabetes; TC: Total cholesterol.

Table 12. Genetic association estimates for the effect of T2DM on TC (Kanai). ea=effect allele, gx=T2DM, gy=TC, se=standard error

|  | SNP | ea | gx | gx_se | gy | gy_se |
| --- | --- | --- | --- | --- | --- | --- |
| 1 | rs10077431 | A | -0.05 | 0.01 | 0.00 | 0.01 |
| 2 | rs10087241 | A | -0.05 | 0.01 | 0.00 | 0.01 |
| 3 | rs10100265 | C | -0.05 | 0.01 | -0.01 | 0.00 |
| 4 | rs10114341 | C | -0.04 | 0.01 | 0.00 | 0.01 |
| 5 | rs10401969 | C | 0.09 | 0.01 | 0.00 | 0.01 |
| 6 | rs1050226 | G | -0.05 | 0.01 | 0.00 | 0.00 |
| 7 | rs1061813 | A | -0.04 | 0.01 | 0.00 | 0.01 |
| 8 | rs1063355 | G | 0.07 | 0.01 | -0.01 | 0.00 |
| 9 | rs10740322 | A | 0.05 | 0.01 | -0.01 | 0.00 |
| 10 | rs10811661 | C | -0.16 | 0.01 | -0.01 | 0.00 |
| 11 | rs10830963 | G | 0.09 | 0.01 | 0.00 | 0.00 |
| 12 | rs10842994 | T | -0.08 | 0.01 | 0.00 | 0.00 |
| 13 | rs10974438 | C | 0.06 | 0.01 | 0.00 | 0.00 |
| 14 | rs11098676 | C | 0.05 | 0.01 | 0.01 | 0.01 |
| 15 | rs11107116 | T | 0.05 | 0.01 | -0.01 | 0.00 |
| 16 | rs1111875 | T | -0.09 | 0.01 | 0.00 | 0.00 |
| 17 | rs11257655 | T | 0.07 | 0.01 | 0.00 | 0.00 |
| 18 | rs1127655 | T | -0.04 | 0.01 | 0.00 | 0.00 |
| 19 | rs11926707 | C | 0.05 | 0.01 | -0.01 | 0.00 |
| 20 | rs12088739 | G | -0.09 | 0.01 | -0.01 | 0.01 |
| 21 | rs12299509 | G | 0.05 | 0.01 | 0.00 | 0.00 |
| 22 | rs12617659 | T | -0.07 | 0.01 | 0.01 | 0.01 |
| 23 | rs12945601 | C | -0.05 | 0.01 | -0.01 | 0.01 |
| 24 | rs12970134 | A | 0.06 | 0.01 | 0.00 | 0.01 |
| 25 | rs13234269 | A | -0.06 | 0.01 | 0.00 | 0.00 |
| 26 | rs13239186 | T | 0.05 | 0.01 | 0.00 | 0.01 |
| 27 | rs13330951 | G | -0.05 | 0.01 | -0.01 | 0.01 |
| 28 | rs13389219 | T | -0.07 | 0.01 | 0.00 | 0.01 |
| 29 | rs1359790 | A | -0.08 | 0.01 | -0.01 | 0.00 |
| 30 | rs1496653 | G | -0.08 | 0.01 | 0.00 | 0.01 |
| 31 | rs1552224 | C | -0.10 | 0.01 | -0.01 | 0.01 |
| 32 | rs17086692 | T | -0.05 | 0.01 | 0.01 | 0.00 |
| 33 | rs17168486 | T | 0.07 | 0.01 | 0.00 | 0.00 |
| 34 | rs17405722 | A | 0.09 | 0.01 | 0.03 | 0.01 |
| 35 | rs17411031 | G | -0.05 | 0.01 | 0.01 | 0.00 |
| 36 | rs1758632 | G | 0.05 | 0.01 | 0.00 | 0.00 |
| 37 | rs17631783 | T | -0.05 | 0.01 | 0.01 | 0.01 |
| 38 | rs17791513 | G | -0.10 | 0.01 | -0.01 | 0.01 |
| 39 | rs1801214 | T | 0.09 | 0.01 | -0.02 | 0.02 |
| 40 | rs1899951 | T | -0.11 | 0.01 | 0.00 | 0.01 |
| 41 | rs2058913 | T | -0.05 | 0.01 | 0.00 | 0.01 |
| 42 | rs2237892 | T | -0.10 | 0.02 | -0.01 | 0.00 |
| 43 | rs2246618 | T | 0.05 | 0.01 | 0.00 | 0.01 |
| 44 | rs2261181 | T | 0.10 | 0.01 | 0.00 | 0.01 |
| 45 | rs2294120 | G | -0.04 | 0.01 | 0.00 | 0.00 |
| 46 | rs2296173 | G | 0.07 | 0.01 | 0.00 | 0.01 |
| 47 | rs2299383 | T | 0.04 | 0.01 | 0.00 | 0.00 |
| 48 | rs243019 | C | 0.06 | 0.01 | 0.00 | 0.00 |
| 49 | rs2493394 | G | 0.07 | 0.01 | 0.03 | 0.01 |
| 50 | rs2796441 | A | -0.07 | 0.01 | 0.00 | 0.00 |
| 51 | rs2820426 | G | 0.05 | 0.01 | 0.00 | 0.00 |
| 52 | rs2867125 | C | 0.06 | 0.01 | -0.01 | 0.01 |
| 53 | rs2908282 | A | 0.06 | 0.01 | 0.00 | 0.00 |
| 54 | rs2925979 | C | -0.05 | 0.01 | 0.00 | 0.00 |
| 55 | rs2943656 | G | 0.09 | 0.01 | 0.00 | 0.01 |
| 56 | rs3217992 | T | 0.05 | 0.01 | 0.00 | 0.00 |
| 57 | rs340874 | C | 0.06 | 0.01 | 0.00 | 0.00 |
| 58 | rs348330 | A | -0.05 | 0.01 | 0.00 | 0.00 |
| 59 | rs3756784 | G | 0.05 | 0.01 | 0.00 | 0.00 |
| 60 | rs3802177 | A | -0.12 | 0.01 | 0.00 | 0.00 |
| 61 | rs459193 | G | 0.07 | 0.01 | -0.01 | 0.00 |
| 62 | rs4622883 | G | -0.04 | 0.01 | 0.00 | 0.00 |
| 63 | rs4812829 | A | 0.05 | 0.01 | 0.00 | 0.00 |
| 64 | rs4823182 | G | 0.05 | 0.01 | -0.02 | 0.00 |
| 65 | rs4865796 | A | 0.05 | 0.01 | 0.00 | 0.01 |
| 66 | rs516946 | C | 0.08 | 0.01 | 0.01 | 0.01 |
| 67 | rs5215 | T | -0.07 | 0.01 | -0.01 | 0.00 |
| 68 | rs576674 | A | -0.07 | 0.01 | 0.00 | 0.01 |
| 69 | rs6059662 | G | 0.04 | 0.01 | 0.00 | 0.00 |
| 70 | rs622217 | C | -0.05 | 0.01 | 0.00 | 0.00 |
| 71 | rs6494307 | G | -0.04 | 0.01 | 0.00 | 0.00 |
| 72 | rs6515236 | C | -0.05 | 0.01 | 0.00 | 0.00 |
| 73 | rs6767484 | G | 0.12 | 0.01 | -0.01 | 0.00 |
| 74 | rs6785040 | C | -0.06 | 0.01 | -0.01 | 0.00 |
| 75 | rs6795735 | T | -0.06 | 0.01 | 0.00 | 0.01 |
| 76 | rs6878122 | A | -0.06 | 0.01 | 0.02 | 0.01 |
| 77 | rs6960043 | C | 0.06 | 0.01 | 0.00 | 0.00 |
| 78 | rs7177055 | A | 0.06 | 0.01 | 0.00 | 0.00 |
| 79 | rs7240767 | C | 0.05 | 0.01 | 0.01 | 0.00 |
| 80 | rs72802358 | C | -0.12 | 0.01 | 0.02 | 0.02 |
| 81 | rs72892910 | T | 0.06 | 0.01 | 0.00 | 0.00 |
| 82 | rs753270 | C | 0.05 | 0.01 | 0.00 | 0.00 |
| 83 | rs7561798 | G | 0.04 | 0.01 | 0.01 | 0.00 |
| 84 | rs7572970 | G | 0.06 | 0.01 | 0.00 | 0.01 |
| 85 | rs7607777 | T | -0.14 | 0.01 | 0.01 | 0.02 |
| 86 | rs7674212 | T | -0.05 | 0.01 | 0.00 | 0.00 |
| 87 | rs7685296 | T | -0.05 | 0.01 | 0.00 | 0.00 |
| 88 | rs7756992 | G | 0.13 | 0.01 | 0.00 | 0.00 |
| 89 | rs7786095 | G | -0.07 | 0.01 | -0.01 | 0.01 |
| 90 | rs780094 | C | 0.07 | 0.01 | -0.04 | 0.00 |
| 91 | rs7845219 | C | -0.04 | 0.01 | 0.00 | 0.00 |
| 92 | rs7903146 | T | 0.31 | 0.01 | 0.01 | 0.01 |
| 93 | rs7929543 | C | 0.08 | 0.01 | 0.00 | 0.01 |
| 94 | rs7955901 | T | -0.04 | 0.01 | 0.00 | 0.00 |
| 95 | rs8068804 | A | 0.06 | 0.01 | -0.01 | 0.00 |
| 96 | rs8108269 | G | 0.06 | 0.01 | 0.01 | 0.00 |
| 97 | rs825476 | T | 0.05 | 0.01 | 0.00 | 0.00 |
| 98 | rs840967 | A | -0.05 | 0.01 | 0.00 | 0.00 |
| 99 | rs853974 | C | -0.06 | 0.01 | 0.00 | 0.00 |
| 100 | rs9369425 | A | -0.05 | 0.01 | 0.00 | 0.01 |
| 101 | rs963740 | T | -0.05 | 0.01 | 0.00 | 0.00 |
| 102 | rs9894220 | G | -0.06 | 0.01 | 0.00 | 0.00 |
| 103 | rs9928094 | G | 0.10 | 0.01 | 0.00 | 0.00 |
| 104 | rs993380 | G | -0.05 | 0.01 | 0.00 | 0.00 |
| 105 | rs9940149 | A | -0.06 | 0.01 | 0.00 | 0.00 |

T2DM: Type-2 diabetes; TC: Total cholesterol.

Table 13. Genetic association estimates for the effect of T2DM on TG (Willer). ea=effect allele, gx=T2DM, gy=TG, se=standard error

|  | SNP | ea | gx | gx_se | gy | gy_se |
| --- | --- | --- | --- | --- | --- | --- |
| 1 | rs10077431 | A | -0.05 | 0.01 | -0.01 | 0.00 |
| 2 | rs10100265 | C | -0.05 | 0.01 | 0.02 | 0.00 |
| 3 | rs10114341 | C | -0.04 | 0.01 | -0.01 | 0.00 |
| 4 | rs10401969 | C | 0.09 | 0.01 | -0.12 | 0.01 |
| 5 | rs1050226 | G | -0.05 | 0.01 | 0.00 | 0.00 |
| 6 | rs1061813 | A | -0.04 | 0.01 | 0.00 | 0.00 |
| 7 | rs1063355 | G | 0.07 | 0.01 | -0.02 | 0.01 |
| 8 | rs10740322 | A | 0.05 | 0.01 | 0.00 | 0.01 |
| 9 | rs10811661 | C | -0.16 | 0.01 | 0.00 | 0.00 |
| 10 | rs10830963 | G | 0.09 | 0.01 | 0.00 | 0.00 |
| 11 | rs10842994 | T | -0.08 | 0.01 | 0.00 | 0.00 |
| 12 | rs10974438 | C | 0.06 | 0.01 | 0.00 | 0.00 |
| 13 | rs11098676 | C | 0.05 | 0.01 | 0.01 | 0.01 |
| 14 | rs11107116 | T | 0.05 | 0.01 | 0.01 | 0.00 |
| 15 | rs1111875 | T | -0.09 | 0.01 | -0.01 | 0.00 |
| 16 | rs11257655 | T | 0.07 | 0.01 | 0.00 | 0.00 |
| 17 | rs1127655 | T | -0.04 | 0.01 | -0.01 | 0.00 |
| 18 | rs11708067 | G | -0.10 | 0.01 | 0.00 | 0.00 |
| 19 | rs11925227 | A | -0.05 | 0.01 | 0.00 | 0.00 |
| 20 | rs11926707 | C | 0.05 | 0.01 | 0.01 | 0.01 |
| 21 | rs12088739 | G | -0.09 | 0.01 | -0.02 | 0.01 |
| 22 | rs12299509 | G | 0.05 | 0.01 | 0.00 | 0.00 |
| 23 | rs12617659 | T | -0.07 | 0.01 | -0.02 | 0.00 |
| 24 | rs12910825 | G | 0.05 | 0.01 | 0.00 | 0.00 |
| 25 | rs12945601 | C | -0.05 | 0.01 | -0.01 | 0.00 |
| 26 | rs12970134 | A | 0.06 | 0.01 | 0.01 | 0.00 |
| 27 | rs13234269 | A | -0.06 | 0.01 | -0.02 | 0.00 |
| 28 | rs13239186 | T | 0.05 | 0.01 | 0.01 | 0.00 |
| 29 | rs13330951 | G | -0.05 | 0.01 | -0.01 | 0.00 |
| 30 | rs13389219 | T | -0.07 | 0.01 | -0.03 | 0.00 |
| 31 | rs1359790 | A | -0.08 | 0.01 | 0.00 | 0.00 |
| 32 | rs1496653 | G | -0.08 | 0.01 | 0.00 | 0.00 |
| 33 | rs1552224 | C | -0.10 | 0.01 | 0.01 | 0.00 |
| 34 | rs16988333 | G | -0.07 | 0.01 | 0.00 | 0.01 |
| 35 | rs17086692 | T | -0.05 | 0.01 | -0.01 | 0.01 |
| 36 | rs17168486 | T | 0.07 | 0.01 | 0.00 | 0.00 |
| 37 | rs17405722 | A | 0.09 | 0.01 | 0.00 | 0.01 |
| 38 | rs17411031 | G | -0.05 | 0.01 | -0.11 | 0.00 |
| 39 | rs1758632 | G | 0.05 | 0.01 | 0.01 | 0.00 |
| 40 | rs17631783 | T | -0.05 | 0.01 | 0.00 | 0.01 |
| 41 | rs17791513 | G | -0.10 | 0.01 | 0.00 | 0.01 |
| 42 | rs1801214 | T | 0.09 | 0.01 | 0.00 | 0.00 |
| 43 | rs1899951 | T | -0.11 | 0.01 | -0.02 | 0.00 |
| 44 | rs2058913 | T | -0.05 | 0.01 | -0.02 | 0.00 |
| 45 | rs2237892 | T | -0.10 | 0.02 | -0.01 | 0.01 |
| 46 | rs2246618 | T | 0.05 | 0.01 | 0.00 | 0.00 |
| 47 | rs2261181 | T | 0.10 | 0.01 | 0.01 | 0.01 |
| 48 | rs2294120 | G | -0.04 | 0.01 | 0.00 | 0.00 |
| 49 | rs2296173 | G | 0.07 | 0.01 | 0.02 | 0.00 |
| 50 | rs2299383 | T | 0.04 | 0.01 | 0.01 | 0.00 |
| 51 | rs243019 | C | 0.06 | 0.01 | 0.00 | 0.00 |
| 52 | rs2493394 | G | 0.07 | 0.01 | 0.00 | 0.01 |
| 53 | rs2796441 | A | -0.07 | 0.01 | 0.01 | 0.00 |
| 54 | rs2820426 | G | 0.05 | 0.01 | 0.02 | 0.00 |
| 55 | rs2867125 | C | 0.06 | 0.01 | 0.01 | 0.00 |
| 56 | rs2908282 | A | 0.06 | 0.01 | 0.00 | 0.00 |
| 57 | rs2925979 | C | -0.05 | 0.01 | -0.02 | 0.00 |
| 58 | rs2943656 | G | 0.09 | 0.01 | 0.03 | 0.00 |
| 59 | rs3217992 | T | 0.05 | 0.01 | 0.00 | 0.00 |
| 60 | rs340874 | C | 0.06 | 0.01 | 0.01 | 0.00 |
| 61 | rs3756784 | G | 0.05 | 0.01 | 0.01 | 0.00 |
| 62 | rs3802177 | A | -0.12 | 0.01 | -0.01 | 0.00 |
| 63 | rs459193 | G | 0.07 | 0.01 | 0.02 | 0.00 |
| 64 | rs4622883 | G | -0.04 | 0.01 | 0.00 | 0.00 |
| 65 | rs4812829 | A | 0.05 | 0.01 | 0.00 | 0.00 |
| 66 | rs4823182 | G | 0.05 | 0.01 | 0.00 | 0.00 |
| 67 | rs4865796 | A | 0.05 | 0.01 | 0.01 | 0.00 |
| 68 | rs516946 | C | 0.08 | 0.01 | -0.01 | 0.00 |
| 69 | rs5215 | T | -0.07 | 0.01 | 0.01 | 0.00 |
| 70 | rs55966194 | G | -0.05 | 0.01 | -0.03 | 0.01 |
| 71 | rs576674 | A | -0.07 | 0.01 | 0.00 | 0.00 |
| 72 | rs6059662 | G | 0.04 | 0.01 | 0.01 | 0.00 |
| 73 | rs622217 | C | -0.05 | 0.01 | -0.02 | 0.00 |
| 74 | rs6494307 | G | -0.04 | 0.01 | -0.01 | 0.00 |
| 75 | rs6515236 | C | -0.05 | 0.01 | 0.01 | 0.01 |
| 76 | rs6767484 | G | 0.12 | 0.01 | 0.01 | 0.00 |
| 77 | rs6785040 | C | -0.06 | 0.01 | 0.00 | 0.01 |
| 78 | rs6795735 | T | -0.06 | 0.01 | -0.01 | 0.00 |
| 79 | rs6878122 | A | -0.06 | 0.01 | 0.00 | 0.00 |
| 80 | rs6960043 | C | 0.06 | 0.01 | 0.00 | 0.00 |
| 81 | rs7144011 | T | 0.05 | 0.01 | 0.01 | 0.00 |
| 82 | rs7177055 | A | 0.06 | 0.01 | 0.00 | 0.00 |
| 83 | rs7240767 | C | 0.05 | 0.01 | 0.00 | 0.01 |
| 84 | rs72802358 | C | -0.12 | 0.01 | 0.00 | 0.01 |
| 85 | rs72892910 | T | 0.06 | 0.01 | 0.00 | 0.01 |
| 86 | rs735949 | C | -0.07 | 0.01 | -0.01 | 0.00 |
| 87 | rs753270 | C | 0.05 | 0.01 | 0.01 | 0.00 |
| 88 | rs7561798 | G | 0.04 | 0.01 | 0.00 | 0.00 |
| 89 | rs7572970 | G | 0.06 | 0.01 | 0.01 | 0.01 |
| 90 | rs7607777 | T | -0.14 | 0.01 | -0.01 | 0.01 |
| 91 | rs7685296 | T | -0.05 | 0.01 | -0.01 | 0.00 |
| 92 | rs7729395 | T | 0.14 | 0.02 | 0.01 | 0.01 |
| 93 | rs7756992 | G | 0.13 | 0.01 | 0.01 | 0.00 |
| 94 | rs7786095 | G | -0.07 | 0.01 | 0.00 | 0.01 |
| 95 | rs780094 | C | 0.07 | 0.01 | -0.11 | 0.00 |
| 96 | rs7845219 | C | -0.04 | 0.01 | -0.01 | 0.00 |
| 97 | rs7903146 | T | 0.31 | 0.01 | 0.00 | 0.00 |
| 98 | rs7929543 | C | 0.08 | 0.01 | 0.01 | 0.01 |
| 99 | rs7955901 | T | -0.04 | 0.01 | 0.00 | 0.00 |
| 100 | rs8068804 | A | 0.06 | 0.01 | 0.00 | 0.00 |
| 101 | rs8108269 | G | 0.06 | 0.01 | 0.01 | 0.00 |
| 102 | rs825476 | T | 0.05 | 0.01 | 0.01 | 0.00 |
| 103 | rs840967 | A | -0.05 | 0.01 | -0.01 | 0.00 |
| 104 | rs849135 | A | -0.10 | 0.01 | 0.00 | 0.00 |
| 105 | rs9369425 | A | -0.05 | 0.01 | 0.01 | 0.01 |
| 106 | rs963740 | T | -0.05 | 0.01 | 0.01 | 0.01 |
| 107 | rs9844972 | C | 0.10 | 0.01 | 0.04 | 0.01 |
| 108 | rs9894220 | G | -0.06 | 0.01 | 0.00 | 0.00 |
| 109 | rs9928094 | G | 0.10 | 0.01 | 0.02 | 0.00 |
| 110 | rs993380 | G | -0.05 | 0.01 | 0.00 | 0.00 |
| 111 | rs9940149 | A | -0.06 | 0.01 | 0.00 | 0.00 |

T2DM: Type-2 diabetes; TG: Triglycerides.

Table 14. Genetic association estimates for the effect of T2DM on TG (Prins). ea=effect allele, gx=T2DM, gy=TG, se=standard error

|  | SNP | ea | gx | gx_se | gy | gy_se |
| --- | --- | --- | --- | --- | --- | --- |
| 1 | rs10077431 | A | -0.05 | 0.02 | 0.02 | 0.01 |
| 2 | rs10100265 | C | -0.05 | 0.01 | 0.02 | 0.01 |
| 3 | rs10114341 | C | -0.04 | -0.01 | 0.01 | 0.01 |
| 4 | rs10401969 | C | 0.09 | -0.13 | 0.03 | 0.01 |
| 5 | rs1050226 | G | -0.05 | -0.02 | 0.02 | 0.01 |
| 6 | rs1061813 | A | -0.04 | 0.01 | 0.01 | 0.01 |
| 7 | rs1063355 | G | 0.07 | -0.03 | 0.02 | 0.01 |
| 8 | rs10740322 | A | 0.05 | -0.01 | 0.02 | 0.01 |
| 9 | rs10830963 | G | 0.09 | 0.01 | 0.02 | 0.01 |
| 10 | rs10842994 | T | -0.08 | 0.01 | 0.02 | 0.01 |
| 11 | rs11098676 | C | 0.05 | 0.02 | 0.02 | 0.01 |
| 12 | rs11107116 | T | 0.05 | 0.02 | 0.02 | 0.01 |
| 13 | rs11257655 | T | 0.07 | -0.02 | 0.02 | 0.01 |
| 14 | rs11708067 | G | -0.10 | -0.01 | 0.02 | 0.01 |
| 15 | rs11925227 | A | -0.05 | -0.01 | 0.02 | 0.01 |
| 16 | rs11926707 | C | 0.05 | -0.01 | 0.02 | 0.01 |
| 17 | rs12088739 | G | -0.09 | -0.05 | 0.03 | 0.01 |
| 18 | rs12910825 | G | 0.05 | 0.03 | 0.02 | 0.01 |
| 19 | rs12945601 | C | -0.05 | 0.01 | 0.02 | 0.01 |
| 20 | rs12970134 | A | 0.06 | 0.01 | 0.02 | 0.01 |
| 21 | rs13234269 | A | -0.06 | -0.04 | 0.01 | 0.01 |
| 22 | rs13239186 | T | 0.05 | 0.01 | 0.02 | 0.01 |
| 23 | rs13330951 | G | -0.05 | -0.01 | 0.01 | 0.01 |
| 24 | rs13389219 | T | -0.07 | -0.03 | 0.02 | 0.01 |
| 25 | rs1496653 | G | -0.08 | -0.02 | 0.02 | 0.01 |
| 26 | rs1552224 | C | -0.10 | -0.02 | 0.02 | 0.01 |
| 27 | rs16988333 | G | -0.07 | -0.01 | 0.03 | 0.01 |
| 28 | rs17086692 | T | -0.05 | 0.01 | 0.02 | 0.01 |
| 29 | rs17168486 | T | 0.07 | 0.03 | 0.02 | 0.01 |
| 30 | rs17405722 | A | 0.09 | 0.02 | 0.03 | 0.01 |
| 31 | rs17411031 | G | -0.05 | -0.13 | 0.02 | 0.01 |
| 32 | rs1758632 | G | 0.05 | 0.03 | 0.02 | 0.01 |
| 33 | rs17631783 | T | -0.05 | -0.01 | 0.02 | 0.01 |
| 34 | rs17791513 | G | -0.10 | -0.05 | 0.03 | 0.01 |
| 35 | rs1801214 | T | 0.09 | 0.01 | 0.02 | 0.01 |
| 36 | rs1899951 | T | -0.11 | -0.01 | 0.02 | 0.01 |
| 37 | rs2058913 | T | -0.05 | -0.01 | 0.02 | 0.01 |
| 38 | rs2237892 | T | -0.10 | -0.03 | 0.03 | 0.02 |
| 39 | rs2261181 | T | 0.10 | 0.01 | 0.02 | 0.01 |
| 40 | rs2294120 | G | -0.04 | 0.01 | 0.01 | 0.01 |
| 41 | rs2296173 | G | 0.07 | 0.02 | 0.02 | 0.01 |
| 42 | rs2299383 | T | 0.04 | 0.03 | 0.01 | 0.01 |
| 43 | rs243019 | C | 0.06 | -0.01 | 0.02 | 0.01 |
| 44 | rs2493394 | G | 0.07 | 0.02 | 0.02 | 0.01 |
| 45 | rs2796441 | A | -0.07 | -0.01 | 0.01 | 0.01 |
| 46 | rs2820426 | G | 0.05 | 0.03 | 0.02 | 0.01 |
| 47 | rs2867125 | C | 0.06 | 0.01 | 0.02 | 0.01 |
| 48 | rs2908282 | A | 0.06 | -0.01 | 0.02 | 0.01 |
| 49 | rs2925979 | C | -0.05 | -0.03 | 0.02 | 0.01 |
| 50 | rs2943656 | G | 0.09 | 0.02 | 0.02 | 0.01 |
| 51 | rs3217992 | T | 0.05 | 0.01 | 0.02 | 0.01 |
| 52 | rs348330 | A | -0.05 | -0.01 | 0.02 | 0.01 |
| 53 | rs3756784 | G | 0.05 | 0.02 | 0.02 | 0.01 |
| 54 | rs3802177 | A | -0.12 | -0.02 | 0.02 | 0.01 |
| 55 | rs459193 | G | 0.07 | 0.03 | 0.02 | 0.01 |
| 56 | rs4622883 | G | -0.04 | -0.03 | 0.01 | 0.01 |
| 57 | rs4686471 | C | 0.05 | 0.01 | 0.02 | 0.01 |
| 58 | rs4812829 | A | 0.05 | -0.04 | 0.02 | 0.01 |
| 59 | rs4823182 | G | 0.05 | 0.01 | 0.02 | 0.01 |
| 60 | rs4865796 | A | 0.05 | 0.01 | 0.02 | 0.01 |
| 61 | rs516946 | C | 0.08 | 0.02 | 0.02 | 0.01 |
| 62 | rs5215 | T | -0.07 | -0.02 | 0.02 | 0.01 |
| 63 | rs55966194 | G | -0.05 | -0.01 | 0.02 | 0.01 |
| 64 | rs576674 | A | -0.07 | 0.01 | 0.02 | 0.01 |
| 65 | rs6059662 | G | 0.04 | 0.02 | 0.02 | 0.01 |
| 66 | rs61953351 | T | -0.07 | 0.02 | 0.02 | 0.01 |
| 67 | rs622217 | C | -0.05 | -0.02 | 0.02 | 0.01 |
| 68 | rs6494307 | G | -0.04 | 0.01 | 0.02 | 0.01 |
| 69 | rs6515236 | C | -0.05 | 0.01 | 0.02 | 0.01 |
| 70 | rs67232546 | T | 0.06 | -0.02 | 0.02 | 0.01 |
| 71 | rs6767484 | G | 0.12 | 0.04 | 0.02 | 0.01 |
| 72 | rs6785040 | C | -0.06 | -0.03 | 0.02 | 0.01 |
| 73 | rs6795735 | T | -0.06 | 0.02 | 0.02 | 0.01 |
| 74 | rs6960043 | C | 0.06 | 0.01 | 0.02 | 0.01 |
| 75 | rs7144011 | T | 0.05 | -0.02 | 0.02 | 0.01 |
| 76 | rs7177055 | A | 0.06 | 0.02 | 0.02 | 0.01 |
| 77 | rs7240767 | C | 0.05 | 0.01 | 0.01 | 0.01 |
| 78 | rs72802358 | C | -0.12 | 0.02 | 0.02 | 0.01 |
| 79 | rs72892910 | T | 0.06 | -0.01 | 0.02 | 0.01 |
| 80 | rs735949 | C | -0.07 | 0.03 | 0.02 | 0.01 |
| 81 | rs753270 | C | 0.05 | 0.02 | 0.02 | 0.01 |
| 82 | rs7561798 | G | 0.04 | 0.01 | 0.01 | 0.01 |
| 83 | rs7572970 | G | 0.06 | -0.01 | 0.02 | 0.01 |
| 84 | rs7607777 | T | -0.14 | -0.01 | 0.02 | 0.01 |
| 85 | rs7674212 | T | -0.05 | -0.03 | 0.02 | 0.01 |
| 86 | rs7685296 | T | -0.05 | -0.01 | 0.02 | 0.01 |
| 87 | rs7729395 | T | 0.14 | 0.04 | 0.03 | 0.02 |
| 88 | rs7756992 | G | 0.13 | -0.01 | 0.02 | 0.01 |
| 89 | rs7786095 | G | -0.07 | 0.01 | 0.02 | 0.01 |
| 90 | rs780094 | C | 0.07 | -0.07 | 0.02 | 0.01 |
| 91 | rs7845219 | C | -0.04 | -0.01 | 0.02 | 0.01 |
| 92 | rs7929543 | C | 0.08 | -0.04 | 0.03 | 0.01 |
| 93 | rs7955901 | T | -0.04 | -0.04 | 0.02 | 0.01 |
| 94 | rs8068804 | A | 0.06 | 0.02 | 0.02 | 0.01 |
| 95 | rs8108269 | G | 0.06 | 0.01 | 0.02 | 0.01 |
| 96 | rs825476 | T | 0.05 | 0.01 | 0.01 | 0.01 |
| 97 | rs840967 | A | -0.05 | -0.01 | 0.02 | 0.01 |
| 98 | rs849135 | A | -0.10 | -0.02 | 0.01 | 0.01 |
| 99 | rs9369425 | A | -0.05 | -0.01 | 0.02 | 0.01 |
| 100 | rs963740 | T | -0.05 | -0.02 | 0.02 | 0.01 |
| 101 | rs9844972 | C | 0.10 | 0.03 | 0.03 | 0.01 |
| 102 | rs9894220 | G | -0.06 | -0.02 | 0.02 | 0.01 |
| 103 | rs9928094 | G | 0.10 | 0.03 | 0.02 | 0.01 |
| 104 | rs993380 | G | -0.05 | -0.01 | 0.02 | 0.01 |
| 105 | rs9940149 | A | -0.06 | 0.01 | 0.02 | 0.01 |

T2DM: Type-2 diabetes; TG: Triglycerides.

Table 15. Genetic association estimates for the effect of T2DM on TG (Kettunen). ea=effect allele, gx=T2DM, gy=TG, se=standard error

|  | SNP | ea | gx | gx_se | gy | gy_se |
| --- | --- | --- | --- | --- | --- | --- |
| 1 | rs10077431 | A | -0.05 | 0.01 | -0.02 | 0.01 |
| 2 | rs10087241 | A | -0.05 | 0.01 | 0.00 | 0.01 |
| 3 | rs10100265 | C | -0.05 | 0.01 | 0.02 | 0.01 |
| 4 | rs10114341 | C | -0.04 | 0.01 | -0.01 | 0.01 |
| 5 | rs10401969 | C | 0.09 | 0.01 | -0.16 | 0.02 |
| 6 | rs1050226 | G | -0.05 | 0.01 | -0.01 | 0.01 |
| 7 | rs1061813 | A | -0.04 | 0.01 | -0.01 | 0.01 |
| 8 | rs1063355 | G | 0.07 | 0.01 | 0.00 | 0.01 |
| 9 | rs10740322 | A | 0.05 | 0.01 | 0.00 | 0.01 |
| 10 | rs10811661 | C | -0.16 | 0.01 | 0.00 | 0.01 |
| 11 | rs10830963 | G | 0.09 | 0.01 | -0.01 | 0.01 |
| 12 | rs10842994 | T | -0.08 | 0.01 | -0.01 | 0.01 |
| 13 | rs10974438 | C | 0.06 | 0.01 | 0.00 | 0.01 |
| 14 | rs11098676 | C | 0.05 | 0.01 | 0.00 | 0.01 |
| 15 | rs11107116 | T | 0.05 | 0.01 | 0.03 | 0.01 |
| 16 | rs1111875 | T | -0.09 | 0.01 | -0.02 | 0.01 |
| 17 | rs11257655 | T | 0.07 | 0.01 | 0.00 | 0.01 |
| 18 | rs1127655 | T | -0.04 | 0.01 | -0.01 | 0.01 |
| 19 | rs11708067 | G | -0.10 | 0.01 | -0.01 | 0.01 |
| 20 | rs11925227 | A | -0.05 | 0.01 | 0.02 | 0.01 |
| 21 | rs11926707 | C | 0.05 | 0.01 | 0.02 | 0.01 |
| 22 | rs12088739 | G | -0.09 | 0.01 | 0.01 | 0.02 |
| 23 | rs12299509 | G | 0.05 | 0.01 | -0.01 | 0.01 |
| 24 | rs12617659 | T | -0.07 | 0.01 | -0.02 | 0.01 |
| 25 | rs12910825 | G | 0.05 | 0.01 | -0.01 | 0.01 |
| 26 | rs12945601 | C | -0.05 | 0.01 | 0.00 | 0.01 |
| 27 | rs12970134 | A | 0.06 | 0.01 | 0.03 | 0.01 |
| 28 | rs13234269 | A | -0.06 | 0.01 | -0.02 | 0.01 |
| 29 | rs13239186 | T | 0.05 | 0.01 | 0.00 | 0.01 |
| 30 | rs13330951 | G | -0.05 | 0.01 | -0.02 | 0.01 |
| 31 | rs13389219 | T | -0.07 | 0.01 | -0.03 | 0.01 |
| 32 | rs1359790 | A | -0.08 | 0.01 | 0.03 | 0.01 |
| 33 | rs1496653 | G | -0.08 | 0.01 | 0.01 | 0.01 |
| 34 | rs1552224 | C | -0.10 | 0.01 | 0.01 | 0.01 |
| 35 | rs16988333 | G | -0.07 | 0.01 | -0.01 | 0.02 |
| 36 | rs17086692 | T | -0.05 | 0.01 | 0.00 | 0.01 |
| 37 | rs17168486 | T | 0.07 | 0.01 | 0.01 | 0.01 |
| 38 | rs17405722 | A | 0.09 | 0.01 | -0.01 | 0.02 |
| 39 | rs17411031 | G | -0.05 | 0.01 | -0.09 | 0.01 |
| 40 | rs1758632 | G | 0.05 | 0.01 | 0.00 | 0.01 |
| 41 | rs17631783 | T | -0.05 | 0.01 | 0.00 | 0.01 |
| 42 | rs17791513 | G | -0.10 | 0.01 | 0.00 | 0.02 |
| 43 | rs1801214 | T | 0.09 | 0.01 | 0.01 | 0.01 |
| 44 | rs1899951 | T | -0.11 | 0.01 | -0.01 | 0.01 |
| 45 | rs2058913 | T | -0.05 | 0.01 | -0.01 | 0.01 |
| 46 | rs2237892 | T | -0.10 | 0.02 | -0.02 | 0.02 |
| 47 | rs2246618 | T | 0.05 | 0.01 | 0.02 | 0.01 |
| 48 | rs2261181 | T | 0.10 | 0.01 | 0.00 | 0.02 |
| 49 | rs2294120 | G | -0.04 | 0.01 | 0.00 | 0.01 |
| 50 | rs2296173 | G | 0.07 | 0.01 | 0.02 | 0.01 |
| 51 | rs2299383 | T | 0.04 | 0.01 | 0.00 | 0.01 |
| 52 | rs243019 | C | 0.06 | 0.01 | -0.03 | 0.01 |
| 53 | rs2493394 | G | 0.07 | 0.01 | 0.01 | 0.01 |
| 54 | rs2796441 | A | -0.07 | 0.01 | 0.00 | 0.01 |
| 55 | rs2820426 | G | 0.05 | 0.01 | 0.02 | 0.01 |
| 56 | rs2867125 | C | 0.06 | 0.01 | 0.02 | 0.01 |
| 57 | rs2908282 | A | 0.06 | 0.01 | 0.00 | 0.02 |
| 58 | rs2925979 | C | -0.05 | 0.01 | 0.00 | 0.01 |
| 59 | rs2943656 | G | 0.09 | 0.01 | 0.03 | 0.01 |
| 60 | rs3217992 | T | 0.05 | 0.01 | 0.00 | 0.01 |
| 61 | rs340874 | C | 0.06 | 0.01 | 0.02 | 0.01 |
| 62 | rs348330 | A | -0.05 | 0.01 | 0.00 | 0.01 |
| 63 | rs3756784 | G | 0.05 | 0.01 | -0.01 | 0.01 |
| 64 | rs3802177 | A | -0.12 | 0.01 | 0.00 | 0.01 |
| 65 | rs459193 | G | 0.07 | 0.01 | 0.00 | 0.01 |
| 66 | rs4622883 | G | -0.04 | 0.01 | -0.01 | 0.01 |
| 67 | rs4686471 | C | 0.05 | 0.01 | -0.01 | 0.01 |
| 68 | rs4812829 | A | 0.05 | 0.01 | 0.00 | 0.01 |
| 69 | rs4823182 | G | 0.05 | 0.01 | 0.01 | 0.01 |
| 70 | rs4865796 | A | 0.05 | 0.01 | 0.02 | 0.01 |
| 71 | rs516946 | C | 0.08 | 0.01 | 0.00 | 0.01 |
| 72 | rs5215 | T | -0.07 | 0.01 | 0.00 | 0.01 |
| 73 | rs55966194 | G | -0.05 | 0.01 | -0.02 | 0.01 |
| 74 | rs576674 | A | -0.07 | 0.01 | -0.02 | 0.02 |
| 75 | rs6059662 | G | 0.04 | 0.01 | 0.00 | 0.01 |
| 76 | rs61953351 | T | -0.07 | 0.01 | 0.00 | 0.01 |
| 77 | rs622217 | C | -0.05 | 0.01 | -0.01 | 0.01 |
| 78 | rs6494307 | G | -0.04 | 0.01 | 0.01 | 0.01 |
| 79 | rs6515236 | C | -0.05 | 0.01 | -0.02 | 0.01 |
| 80 | rs67232546 | T | 0.06 | 0.01 | -0.02 | 0.01 |
| 81 | rs6767484 | G | 0.12 | 0.01 | 0.03 | 0.01 |
| 82 | rs6785040 | C | -0.06 | 0.01 | -0.02 | 0.01 |
| 83 | rs6795735 | T | -0.06 | 0.01 | 0.00 | 0.01 |
| 84 | rs6878122 | A | -0.06 | 0.01 | -0.01 | 0.01 |
| 85 | rs6960043 | C | 0.06 | 0.01 | 0.00 | 0.01 |
| 86 | rs7144011 | T | 0.05 | 0.01 | 0.00 | 0.01 |
| 87 | rs7177055 | A | 0.06 | 0.01 | 0.02 | 0.01 |
| 88 | rs7240767 | C | 0.05 | 0.01 | 0.01 | 0.01 |
| 89 | rs72802358 | C | -0.12 | 0.01 | -0.04 | 0.02 |
| 90 | rs72892910 | T | 0.06 | 0.01 | 0.03 | 0.01 |
| 91 | rs735949 | C | -0.07 | 0.01 | -0.01 | 0.02 |
| 92 | rs753270 | C | 0.05 | 0.01 | 0.01 | 0.01 |
| 93 | rs7561798 | G | 0.04 | 0.01 | -0.01 | 0.01 |
| 94 | rs7572970 | G | 0.06 | 0.01 | 0.01 | 0.01 |
| 95 | rs7607777 | T | -0.14 | 0.01 | -0.03 | 0.02 |
| 96 | rs7674212 | T | -0.05 | 0.01 | 0.00 | 0.01 |
| 97 | rs7685296 | T | -0.05 | 0.01 | -0.01 | 0.01 |
| 98 | rs7729395 | T | 0.14 | 0.02 | 0.00 | 0.02 |
| 99 | rs7756992 | G | 0.13 | 0.01 | 0.01 | 0.01 |
| 100 | rs7786095 | G | -0.07 | 0.01 | 0.00 | 0.02 |
| 101 | rs780094 | C | 0.07 | 0.01 | -0.10 | 0.01 |
| 102 | rs7845219 | C | -0.04 | 0.01 | 0.00 | 0.01 |
| 103 | rs7903146 | T | 0.31 | 0.01 | 0.02 | 0.01 |
| 104 | rs7929543 | C | 0.08 | 0.01 | 0.02 | 0.02 |
| 105 | rs7955901 | T | -0.04 | 0.01 | 0.02 | 0.01 |
| 106 | rs8068804 | A | 0.06 | 0.01 | 0.01 | 0.01 |
| 107 | rs8108269 | G | 0.06 | 0.01 | 0.00 | 0.01 |
| 108 | rs825476 | T | 0.05 | 0.01 | 0.03 | 0.01 |
| 109 | rs840967 | A | -0.05 | 0.01 | 0.00 | 0.01 |
| 110 | rs849135 | A | -0.10 | 0.01 | -0.01 | 0.01 |
| 111 | rs853974 | C | -0.06 | 0.01 | 0.00 | 0.01 |
| 112 | rs9369425 | A | -0.05 | 0.01 | 0.02 | 0.01 |
| 113 | rs963740 | T | -0.05 | 0.01 | 0.01 | 0.01 |
| 114 | rs9844972 | C | 0.10 | 0.01 | 0.00 | 0.02 |
| 115 | rs9894220 | G | -0.06 | 0.01 | 0.01 | 0.01 |
| 116 | rs9928094 | G | 0.10 | 0.01 | 0.02 | 0.01 |
| 117 | rs993380 | G | -0.05 | 0.01 | -0.01 | 0.01 |
| 118 | rs9940149 | A | -0.06 | 0.01 | -0.01 | 0.02 |

T2DM: Type-2 diabetes; TG: Triglycerides.

Table 16. Genetic association estimates for the effect of T2DM on TG (Kanai). ea=effect allele, gx=T2DM, gy=TG, se=standard error

|  | SNP | ea | gx | gx_se | gy | gy_se |
| --- | --- | --- | --- | --- | --- | --- |
| 1 | rs10077431 | A | -0.05 | 0.01 | 0.00 | 0.01 |
| 2 | rs10087241 | A | -0.05 | 0.01 | 0.00 | 0.01 |
| 3 | rs10100265 | C | -0.05 | 0.01 | 0.01 | 0.00 |
| 4 | rs10114341 | C | -0.04 | 0.01 | 0.00 | 0.01 |
| 5 | rs10401969 | C | 0.09 | 0.01 | -0.04 | 0.01 |
| 6 | rs1050226 | G | -0.05 | 0.01 | 0.00 | 0.00 |
| 7 | rs1061813 | A | -0.04 | 0.01 | -0.01 | 0.01 |
| 8 | rs1063355 | G | 0.07 | 0.01 | -0.02 | 0.00 |
| 9 | rs10740322 | A | 0.05 | 0.01 | -0.01 | 0.00 |
| 10 | rs10811661 | C | -0.16 | 0.01 | 0.00 | 0.00 |
| 11 | rs10830963 | G | 0.09 | 0.01 | 0.00 | 0.00 |
| 12 | rs10842994 | T | -0.08 | 0.01 | 0.00 | 0.01 |
| 13 | rs10974438 | C | 0.06 | 0.01 | -0.01 | 0.00 |
| 14 | rs11098676 | C | 0.05 | 0.01 | 0.01 | 0.01 |
| 15 | rs11107116 | T | 0.05 | 0.01 | 0.00 | 0.00 |
| 16 | rs1111875 | T | -0.09 | 0.01 | 0.01 | 0.00 |
| 17 | rs11257655 | T | 0.07 | 0.01 | -0.01 | 0.00 |
| 18 | rs1127655 | T | -0.04 | 0.01 | -0.01 | 0.00 |
| 19 | rs11926707 | C | 0.05 | 0.01 | 0.01 | 0.01 |
| 20 | rs12088739 | G | -0.09 | 0.01 | -0.02 | 0.01 |
| 21 | rs12299509 | G | 0.05 | 0.01 | 0.00 | 0.00 |
| 22 | rs12617659 | T | -0.07 | 0.01 | 0.00 | 0.01 |
| 23 | rs12945601 | C | -0.05 | 0.01 | 0.01 | 0.01 |
| 24 | rs12970134 | A | 0.06 | 0.01 | 0.01 | 0.01 |
| 25 | rs13234269 | A | -0.06 | 0.01 | -0.02 | 0.01 |
| 26 | rs13239186 | T | 0.05 | 0.01 | 0.01 | 0.01 |
| 27 | rs13330951 | G | -0.05 | 0.01 | 0.01 | 0.01 |
| 28 | rs13389219 | T | -0.07 | 0.01 | 0.00 | 0.01 |
| 29 | rs1359790 | A | -0.08 | 0.01 | 0.01 | 0.00 |
| 30 | rs1496653 | G | -0.08 | 0.01 | 0.00 | 0.01 |
| 31 | rs1552224 | C | -0.10 | 0.01 | 0.01 | 0.01 |
| 32 | rs17086692 | T | -0.05 | 0.01 | 0.00 | 0.00 |
| 33 | rs17168486 | T | 0.07 | 0.01 | -0.01 | 0.00 |
| 34 | rs17405722 | A | 0.09 | 0.01 | 0.00 | 0.01 |
| 35 | rs17411031 | G | -0.05 | 0.01 | -0.13 | 0.01 |
| 36 | rs1758632 | G | 0.05 | 0.01 | 0.00 | 0.00 |
| 37 | rs17631783 | T | -0.05 | 0.01 | -0.01 | 0.01 |
| 38 | rs17791513 | G | -0.10 | 0.01 | 0.00 | 0.01 |
| 39 | rs1801214 | T | 0.09 | 0.01 | -0.02 | 0.02 |
| 40 | rs1899951 | T | -0.11 | 0.01 | -0.02 | 0.01 |
| 41 | rs2058913 | T | -0.05 | 0.01 | 0.01 | 0.01 |
| 42 | rs2237892 | T | -0.10 | 0.02 | 0.00 | 0.00 |
| 43 | rs2246618 | T | 0.05 | 0.01 | 0.00 | 0.01 |
| 44 | rs2261181 | T | 0.10 | 0.01 | 0.00 | 0.01 |
| 45 | rs2294120 | G | -0.04 | 0.01 | -0.01 | 0.00 |
| 46 | rs2296173 | G | 0.07 | 0.01 | 0.02 | 0.01 |
| 47 | rs2299383 | T | 0.04 | 0.01 | 0.00 | 0.00 |
| 48 | rs243019 | C | 0.06 | 0.01 | 0.00 | 0.00 |
| 49 | rs2493394 | G | 0.07 | 0.01 | -0.03 | 0.02 |
| 50 | rs2796441 | A | -0.07 | 0.01 | 0.01 | 0.00 |
| 51 | rs2820426 | G | 0.05 | 0.01 | 0.01 | 0.00 |
| 52 | rs2867125 | C | 0.06 | 0.01 | 0.01 | 0.01 |
| 53 | rs2908282 | A | 0.06 | 0.01 | 0.01 | 0.01 |
| 54 | rs2925979 | C | -0.05 | 0.01 | -0.02 | 0.00 |
| 55 | rs2943656 | G | 0.09 | 0.01 | 0.03 | 0.01 |
| 56 | rs3217992 | T | 0.05 | 0.01 | 0.00 | 0.00 |
| 57 | rs340874 | C | 0.06 | 0.01 | 0.00 | 0.00 |
| 58 | rs348330 | A | -0.05 | 0.01 | 0.00 | 0.01 |
| 59 | rs3756784 | G | 0.05 | 0.01 | 0.00 | 0.00 |
| 60 | rs3802177 | A | -0.12 | 0.01 | 0.00 | 0.00 |
| 61 | rs459193 | G | 0.07 | 0.01 | 0.02 | 0.00 |
| 62 | rs4622883 | G | -0.04 | 0.01 | 0.00 | 0.01 |
| 63 | rs4812829 | A | 0.05 | 0.01 | 0.01 | 0.00 |
| 64 | rs4823182 | G | 0.05 | 0.01 | -0.01 | 0.00 |
| 65 | rs4865796 | A | 0.05 | 0.01 | 0.01 | 0.01 |
| 66 | rs516946 | C | 0.08 | 0.01 | -0.01 | 0.01 |
| 67 | rs5215 | T | -0.07 | 0.01 | 0.00 | 0.00 |
| 68 | rs576674 | A | -0.07 | 0.01 | 0.01 | 0.01 |
| 69 | rs6059662 | G | 0.04 | 0.01 | 0.01 | 0.01 |
| 70 | rs622217 | C | -0.05 | 0.01 | 0.00 | 0.00 |
| 71 | rs6494307 | G | -0.04 | 0.01 | 0.01 | 0.00 |
| 72 | rs6515236 | C | -0.05 | 0.01 | 0.00 | 0.00 |
| 73 | rs6767484 | G | 0.12 | 0.01 | 0.00 | 0.00 |
| 74 | rs6785040 | C | -0.06 | 0.01 | 0.00 | 0.00 |
| 75 | rs6795735 | T | -0.06 | 0.01 | 0.00 | 0.01 |
| 76 | rs6878122 | A | -0.06 | 0.01 | 0.01 | 0.02 |
| 77 | rs6960043 | C | 0.06 | 0.01 | 0.00 | 0.00 |
| 78 | rs7177055 | A | 0.06 | 0.01 | -0.01 | 0.00 |
| 79 | rs7240767 | C | 0.05 | 0.01 | 0.00 | 0.00 |
| 80 | rs72802358 | C | -0.12 | 0.01 | 0.03 | 0.02 |
| 81 | rs72892910 | T | 0.06 | 0.01 | 0.00 | 0.01 |
| 82 | rs753270 | C | 0.05 | 0.01 | 0.00 | 0.01 |
| 83 | rs7561798 | G | 0.04 | 0.01 | 0.00 | 0.00 |
| 84 | rs7572970 | G | 0.06 | 0.01 | 0.00 | 0.01 |
| 85 | rs7607777 | T | -0.14 | 0.01 | 0.00 | 0.02 |
| 86 | rs7674212 | T | -0.05 | 0.01 | 0.00 | 0.00 |
| 87 | rs7685296 | T | -0.05 | 0.01 | 0.00 | 0.00 |
| 88 | rs7756992 | G | 0.13 | 0.01 | -0.01 | 0.00 |
| 89 | rs7786095 | G | -0.07 | 0.01 | 0.01 | 0.01 |
| 90 | rs780094 | C | 0.07 | 0.01 | -0.08 | 0.00 |
| 91 | rs7845219 | C | -0.04 | 0.01 | 0.00 | 0.00 |
| 92 | rs7903146 | T | 0.31 | 0.01 | -0.03 | 0.01 |
| 93 | rs7929543 | C | 0.08 | 0.01 | 0.00 | 0.01 |
| 94 | rs7955901 | T | -0.04 | 0.01 | 0.00 | 0.00 |
| 95 | rs8068804 | A | 0.06 | 0.01 | 0.00 | 0.00 |
| 96 | rs8108269 | G | 0.06 | 0.01 | 0.00 | 0.00 |
| 97 | rs825476 | T | 0.05 | 0.01 | 0.01 | 0.01 |
| 98 | rs840967 | A | -0.05 | 0.01 | 0.01 | 0.00 |
| 99 | rs853974 | C | -0.06 | 0.01 | -0.01 | 0.00 |
| 100 | rs9369425 | A | -0.05 | 0.01 | 0.02 | 0.01 |
| 101 | rs963740 | T | -0.05 | 0.01 | 0.01 | 0.00 |
| 102 | rs9894220 | G | -0.06 | 0.01 | 0.00 | 0.00 |
| 103 | rs9928094 | G | 0.10 | 0.01 | 0.01 | 0.01 |
| 104 | rs993380 | G | -0.05 | 0.01 | 0.01 | 0.00 |
| 105 | rs9940149 | A | -0.06 | 0.01 | 0.01 | 0.00 |

T2DM: Type-2 diabetes; TG: Triglycerides.

Table 17. Genetic association estimates for the effect of T2DM on small HDL particle. ea=effect allele, gx=T2DM, gy= small HDL particle, se=standard error

|  | SNP | ea | gx | gx_se | gy | gy_se |
| --- | --- | --- | --- | --- | --- | --- |
| 1 | rs10077431 | A | -0.05 | 0.01 | -0.01 | 0.01 |
| 2 | rs10087241 | A | -0.05 | 0.01 | -0.01 | 0.01 |
| 3 | rs10100265 | C | -0.05 | 0.01 | 0.01 | 0.01 |
| 4 | rs10114341 | C | -0.04 | 0.01 | -0.01 | 0.01 |
| 5 | rs10401969 | C | 0.09 | 0.01 | -0.06 | 0.02 |
| 6 | rs1050226 | G | -0.05 | 0.01 | 0.00 | 0.01 |
| 7 | rs1061813 | A | -0.04 | 0.01 | 0.00 | 0.01 |
| 8 | rs1063355 | G | 0.07 | 0.01 | 0.01 | 0.01 |
| 9 | rs10740322 | A | 0.05 | 0.01 | -0.01 | 0.01 |
| 10 | rs10811661 | C | -0.16 | 0.01 | 0.00 | 0.01 |
| 11 | rs10830963 | G | 0.09 | 0.01 | 0.00 | 0.01 |
| 12 | rs10842994 | T | -0.08 | 0.01 | 0.01 | 0.01 |
| 13 | rs10974438 | C | 0.06 | 0.01 | 0.02 | 0.01 |
| 14 | rs11098676 | C | 0.05 | 0.01 | 0.01 | 0.01 |
| 15 | rs11107116 | T | 0.05 | 0.01 | 0.02 | 0.01 |
| 16 | rs1111875 | T | -0.09 | 0.01 | -0.01 | 0.01 |
| 17 | rs11257655 | T | 0.07 | 0.01 | 0.00 | 0.01 |
| 18 | rs1127655 | T | -0.04 | 0.01 | 0.00 | 0.01 |
| 19 | rs11708067 | G | -0.10 | 0.01 | 0.01 | 0.01 |
| 20 | rs11925227 | A | -0.05 | 0.01 | 0.01 | 0.01 |
| 21 | rs11926707 | C | 0.05 | 0.01 | -0.02 | 0.01 |
| 22 | rs12088739 | G | -0.09 | 0.01 | -0.03 | 0.02 |
| 23 | rs12299509 | G | 0.05 | 0.01 | 0.00 | 0.01 |
| 24 | rs12617659 | T | -0.07 | 0.01 | -0.01 | 0.01 |
| 25 | rs12910825 | G | 0.05 | 0.01 | 0.00 | 0.01 |
| 26 | rs12945601 | C | -0.05 | 0.01 | 0.01 | 0.01 |
| 27 | rs12970134 | A | 0.06 | 0.01 | 0.01 | 0.01 |
| 28 | rs13234269 | A | -0.06 | 0.01 | -0.02 | 0.01 |
| 29 | rs13239186 | T | 0.05 | 0.01 | 0.00 | 0.01 |
| 30 | rs13330951 | G | -0.05 | 0.01 | 0.01 | 0.01 |
| 31 | rs13389219 | T | -0.07 | 0.01 | 0.01 | 0.01 |
| 32 | rs1359790 | A | -0.08 | 0.01 | 0.03 | 0.01 |
| 33 | rs1496653 | G | -0.08 | 0.01 | 0.01 | 0.01 |
| 34 | rs1552224 | C | -0.10 | 0.01 | -0.01 | 0.01 |
| 35 | rs16988333 | G | -0.07 | 0.01 | 0.02 | 0.02 |
| 36 | rs17086692 | T | -0.05 | 0.01 | 0.00 | 0.01 |
| 37 | rs17168486 | T | 0.07 | 0.01 | -0.01 | 0.01 |
| 38 | rs17405722 | A | 0.09 | 0.01 | 0.02 | 0.02 |
| 39 | rs17411031 | G | -0.05 | 0.01 | -0.01 | 0.01 |
| 40 | rs1758632 | G | 0.05 | 0.01 | -0.02 | 0.01 |
| 41 | rs17631783 | T | -0.05 | 0.01 | -0.01 | 0.01 |
| 42 | rs17791513 | G | -0.10 | 0.01 | 0.00 | 0.02 |
| 43 | rs1801214 | T | 0.09 | 0.01 | 0.02 | 0.01 |
| 44 | rs1899951 | T | -0.11 | 0.01 | 0.01 | 0.01 |
| 45 | rs2058913 | T | -0.05 | 0.01 | 0.02 | 0.01 |
| 46 | rs2237892 | T | -0.10 | 0.02 | -0.03 | 0.02 |
| 47 | rs2246618 | T | 0.05 | 0.01 | 0.00 | 0.01 |
| 48 | rs2261181 | T | 0.10 | 0.01 | 0.01 | 0.02 |
| 49 | rs2294120 | G | -0.04 | 0.01 | 0.00 | 0.01 |
| 50 | rs2296173 | G | 0.07 | 0.01 | -0.02 | 0.01 |
| 51 | rs2299383 | T | 0.04 | 0.01 | 0.01 | 0.01 |
| 52 | rs243019 | C | 0.06 | 0.01 | 0.00 | 0.01 |
| 53 | rs2493394 | G | 0.07 | 0.01 | 0.03 | 0.02 |
| 54 | rs2796441 | A | -0.07 | 0.01 | 0.00 | 0.01 |
| 55 | rs2820426 | G | 0.05 | 0.01 | 0.01 | 0.01 |
| 56 | rs2867125 | C | 0.06 | 0.01 | 0.01 | 0.01 |
| 57 | rs2908282 | A | 0.06 | 0.01 | 0.01 | 0.02 |
| 58 | rs2925979 | C | -0.05 | 0.01 | 0.00 | 0.01 |
| 59 | rs2943656 | G | 0.09 | 0.01 | -0.01 | 0.01 |
| 60 | rs3217992 | T | 0.05 | 0.01 | 0.01 | 0.01 |
| 61 | rs340874 | C | 0.06 | 0.01 | 0.01 | 0.01 |
| 62 | rs348330 | A | -0.05 | 0.01 | 0.00 | 0.01 |
| 63 | rs3756784 | G | 0.05 | 0.01 | -0.03 | 0.01 |
| 64 | rs3802177 | A | -0.12 | 0.01 | -0.01 | 0.01 |
| 65 | rs459193 | G | 0.07 | 0.01 | 0.01 | 0.01 |
| 66 | rs4622883 | G | -0.04 | 0.01 | -0.01 | 0.01 |
| 67 | rs4686471 | C | 0.05 | 0.01 | -0.01 | 0.01 |
| 68 | rs4812829 | A | 0.05 | 0.01 | 0.01 | 0.01 |
| 69 | rs4823182 | G | 0.05 | 0.01 | 0.01 | 0.01 |
| 70 | rs4865796 | A | 0.05 | 0.01 | 0.02 | 0.01 |
| 71 | rs516946 | C | 0.08 | 0.01 | -0.02 | 0.01 |
| 72 | rs5215 | T | -0.07 | 0.01 | -0.01 | 0.01 |
| 73 | rs55966194 | G | -0.05 | 0.01 | -0.03 | 0.01 |
| 74 | rs576674 | A | -0.07 | 0.01 | 0.01 | 0.02 |
| 75 | rs6059662 | G | 0.04 | 0.01 | -0.01 | 0.01 |
| 76 | rs61953351 | T | -0.07 | 0.01 | -0.01 | 0.01 |
| 77 | rs622217 | C | -0.05 | 0.01 | -0.02 | 0.01 |
| 78 | rs6494307 | G | -0.04 | 0.01 | 0.00 | 0.01 |
| 79 | rs6515236 | C | -0.05 | 0.01 | 0.00 | 0.01 |
| 80 | rs67232546 | T | 0.06 | 0.01 | -0.02 | 0.01 |
| 81 | rs6767484 | G | 0.12 | 0.01 | 0.00 | 0.01 |
| 82 | rs6785040 | C | -0.06 | 0.01 | 0.00 | 0.01 |
| 83 | rs6795735 | T | -0.06 | 0.01 | 0.00 | 0.01 |
| 84 | rs6878122 | A | -0.06 | 0.01 | -0.02 | 0.01 |
| 85 | rs6960043 | C | 0.06 | 0.01 | 0.00 | 0.01 |
| 86 | rs7144011 | T | 0.05 | 0.01 | -0.03 | 0.01 |
| 87 | rs7177055 | A | 0.06 | 0.01 | 0.00 | 0.01 |
| 88 | rs7240767 | C | 0.05 | 0.01 | 0.01 | 0.01 |
| 89 | rs72802358 | C | -0.12 | 0.01 | 0.01 | 0.02 |
| 90 | rs72892910 | T | 0.06 | 0.01 | 0.01 | 0.01 |
| 91 | rs735949 | C | -0.07 | 0.01 | 0.01 | 0.02 |
| 92 | rs753270 | C | 0.05 | 0.01 | 0.00 | 0.01 |
| 93 | rs7561798 | G | 0.04 | 0.01 | 0.01 | 0.01 |
| 94 | rs7572970 | G | 0.06 | 0.01 | 0.01 | 0.01 |
| 95 | rs7607777 | T | -0.14 | 0.01 | -0.05 | 0.02 |
| 96 | rs7674212 | T | -0.05 | 0.01 | -0.01 | 0.01 |
| 97 | rs7685296 | T | -0.05 | 0.01 | 0.02 | 0.01 |
| 98 | rs7729395 | T | 0.14 | 0.02 | 0.01 | 0.02 |
| 99 | rs7756992 | G | 0.13 | 0.01 | 0.00 | 0.01 |
| 100 | rs7786095 | G | -0.07 | 0.01 | -0.02 | 0.02 |
| 101 | rs780094 | C | 0.07 | 0.01 | -0.07 | 0.01 |
| 102 | rs7845219 | C | -0.04 | 0.01 | 0.00 | 0.01 |
| 103 | rs7903146 | T | 0.31 | 0.01 | -0.01 | 0.01 |
| 104 | rs7929543 | C | 0.08 | 0.01 | -0.01 | 0.02 |
| 105 | rs7955901 | T | -0.04 | 0.01 | -0.01 | 0.01 |
| 106 | rs8068804 | A | 0.06 | 0.01 | -0.01 | 0.01 |
| 107 | rs8108269 | G | 0.06 | 0.01 | -0.01 | 0.01 |
| 108 | rs825476 | T | 0.05 | 0.01 | -0.01 | 0.01 |
| 109 | rs840967 | A | -0.05 | 0.01 | -0.01 | 0.01 |
| 110 | rs849135 | A | -0.10 | 0.01 | -0.01 | 0.01 |
| 111 | rs853974 | C | -0.06 | 0.01 | 0.00 | 0.01 |
| 112 | rs9369425 | A | -0.05 | 0.01 | 0.00 | 0.01 |
| 113 | rs963740 | T | -0.05 | 0.01 | 0.02 | 0.01 |
| 114 | rs9844972 | C | 0.10 | 0.01 | 0.03 | 0.02 |
| 115 | rs9894220 | G | -0.06 | 0.01 | 0.01 | 0.01 |
| 116 | rs9928094 | G | 0.10 | 0.01 | 0.01 | 0.01 |
| 117 | rs993380 | G | -0.05 | 0.01 | -0.01 | 0.01 |
| 118 | rs9940149 | A | -0.06 | 0.01 | 0.02 | 0.02 |

T2DM: Type-2 diabetes; HDL: High-density lipoprotein.

Table 18. Genetic association estimates for the effect of T2DM on medium HDL particle. ea=effect allele, gx=T2DM, gy= medium HDL particle, se=standard error

|  | SNP | ea | gx | gx_se | gy | gy_se |
| --- | --- | --- | --- | --- | --- | --- |
| 1 | rs10077431 | A | -0.05 | 0.01 | 0.00 | 0.01 |
| 2 | rs10087241 | A | -0.05 | 0.01 | -0.01 | 0.01 |
| 3 | rs10100265 | C | -0.05 | 0.01 | 0.00 | 0.01 |
| 4 | rs10114341 | C | -0.04 | 0.01 | 0.00 | 0.01 |
| 5 | rs10401969 | C | 0.09 | 0.01 | -0.06 | 0.02 |
| 6 | rs1050226 | G | -0.05 | 0.01 | 0.01 | 0.01 |
| 7 | rs1061813 | A | -0.04 | 0.01 | 0.01 | 0.01 |
| 8 | rs1063355 | G | 0.07 | 0.01 | 0.00 | 0.01 |
| 9 | rs10740322 | A | 0.05 | 0.01 | 0.00 | 0.01 |
| 10 | rs10811661 | C | -0.16 | 0.01 | 0.01 | 0.01 |
| 11 | rs10830963 | G | 0.09 | 0.01 | 0.01 | 0.01 |
| 12 | rs10842994 | T | -0.08 | 0.01 | 0.01 | 0.01 |
| 13 | rs10974438 | C | 0.06 | 0.01 | 0.02 | 0.01 |
| 14 | rs11098676 | C | 0.05 | 0.01 | 0.01 | 0.01 |
| 15 | rs11107116 | T | 0.05 | 0.01 | 0.01 | 0.01 |
| 16 | rs1111875 | T | -0.09 | 0.01 | 0.01 | 0.01 |
| 17 | rs11257655 | T | 0.07 | 0.01 | -0.02 | 0.01 |
| 18 | rs1127655 | T | -0.04 | 0.01 | 0.00 | 0.01 |
| 19 | rs11708067 | G | -0.10 | 0.01 | 0.01 | 0.01 |
| 20 | rs11925227 | A | -0.05 | 0.01 | 0.00 | 0.01 |
| 21 | rs11926707 | C | 0.05 | 0.01 | -0.01 | 0.01 |
| 22 | rs12088739 | G | -0.09 | 0.01 | -0.03 | 0.02 |
| 23 | rs12299509 | G | 0.05 | 0.01 | 0.02 | 0.01 |
| 24 | rs12617659 | T | -0.07 | 0.01 | 0.01 | 0.01 |
| 25 | rs12910825 | G | 0.05 | 0.01 | 0.01 | 0.01 |
| 26 | rs12945601 | C | -0.05 | 0.01 | 0.01 | 0.01 |
| 27 | rs12970134 | A | 0.06 | 0.01 | -0.01 | 0.01 |
| 28 | rs13234269 | A | -0.06 | 0.01 | 0.00 | 0.01 |
| 29 | rs13239186 | T | 0.05 | 0.01 | 0.01 | 0.01 |
| 30 | rs13330951 | G | -0.05 | 0.01 | 0.00 | 0.01 |
| 31 | rs13389219 | T | -0.07 | 0.01 | 0.02 | 0.01 |
| 32 | rs1359790 | A | -0.08 | 0.01 | 0.02 | 0.01 |
| 33 | rs1496653 | G | -0.08 | 0.01 | 0.01 | 0.01 |
| 34 | rs1552224 | C | -0.10 | 0.01 | -0.02 | 0.01 |
| 35 | rs16988333 | G | -0.07 | 0.01 | 0.01 | 0.02 |
| 36 | rs17086692 | T | -0.05 | 0.01 | -0.02 | 0.01 |
| 37 | rs17168486 | T | 0.07 | 0.01 | 0.00 | 0.01 |
| 38 | rs17405722 | A | 0.09 | 0.01 | -0.02 | 0.02 |
| 39 | rs17411031 | G | -0.05 | 0.01 | 0.04 | 0.01 |
| 40 | rs1758632 | G | 0.05 | 0.01 | -0.01 | 0.01 |
| 41 | rs17631783 | T | -0.05 | 0.01 | -0.01 | 0.01 |
| 42 | rs17791513 | G | -0.10 | 0.01 | -0.01 | 0.02 |
| 43 | rs1801214 | T | 0.09 | 0.01 | 0.01 | 0.01 |
| 44 | rs1899951 | T | -0.11 | 0.01 | 0.02 | 0.01 |
| 45 | rs2058913 | T | -0.05 | 0.01 | 0.01 | 0.01 |
| 46 | rs2237892 | T | -0.10 | 0.02 | -0.02 | 0.02 |
| 47 | rs2246618 | T | 0.05 | 0.01 | 0.00 | 0.01 |
| 48 | rs2261181 | T | 0.10 | 0.01 | 0.00 | 0.02 |
| 49 | rs2294120 | G | -0.04 | 0.01 | 0.00 | 0.01 |
| 50 | rs2296173 | G | 0.07 | 0.01 | -0.01 | 0.01 |
| 51 | rs2299383 | T | 0.04 | 0.01 | -0.01 | 0.01 |
| 52 | rs243019 | C | 0.06 | 0.01 | 0.01 | 0.01 |
| 53 | rs2493394 | G | 0.07 | 0.01 | 0.02 | 0.02 |
| 54 | rs2796441 | A | -0.07 | 0.01 | 0.00 | 0.01 |
| 55 | rs2820426 | G | 0.05 | 0.01 | 0.01 | 0.01 |
| 56 | rs2867125 | C | 0.06 | 0.01 | 0.00 | 0.01 |
| 57 | rs2908282 | A | 0.06 | 0.01 | 0.01 | 0.02 |
| 58 | rs2925979 | C | -0.05 | 0.01 | 0.01 | 0.01 |
| 59 | rs2943656 | G | 0.09 | 0.01 | -0.04 | 0.01 |
| 60 | rs3217992 | T | 0.05 | 0.01 | 0.02 | 0.01 |
| 61 | rs340874 | C | 0.06 | 0.01 | -0.01 | 0.01 |
| 62 | rs348330 | A | -0.05 | 0.01 | 0.01 | 0.01 |
| 63 | rs3756784 | G | 0.05 | 0.01 | -0.02 | 0.01 |
| 64 | rs3802177 | A | -0.12 | 0.01 | -0.01 | 0.01 |
| 65 | rs459193 | G | 0.07 | 0.01 | -0.03 | 0.01 |
| 66 | rs4622883 | G | -0.04 | 0.01 | -0.01 | 0.01 |
| 67 | rs4686471 | C | 0.05 | 0.01 | -0.01 | 0.01 |
| 68 | rs4812829 | A | 0.05 | 0.01 | 0.02 | 0.01 |
| 69 | rs4823182 | G | 0.05 | 0.01 | 0.00 | 0.01 |
| 70 | rs4865796 | A | 0.05 | 0.01 | 0.01 | 0.01 |
| 71 | rs516946 | C | 0.08 | 0.01 | -0.02 | 0.01 |
| 72 | rs5215 | T | -0.07 | 0.01 | -0.01 | 0.01 |
| 73 | rs55966194 | G | -0.05 | 0.01 | -0.02 | 0.01 |
| 74 | rs576674 | A | -0.07 | 0.01 | 0.01 | 0.02 |
| 75 | rs6059662 | G | 0.04 | 0.01 | -0.02 | 0.01 |
| 76 | rs61953351 | T | -0.07 | 0.01 | -0.01 | 0.01 |
| 77 | rs622217 | C | -0.05 | 0.01 | 0.00 | 0.01 |
| 78 | rs6494307 | G | -0.04 | 0.01 | -0.01 | 0.01 |
| 79 | rs6515236 | C | -0.05 | 0.01 | 0.01 | 0.01 |
| 80 | rs67232546 | T | 0.06 | 0.01 | 0.00 | 0.01 |
| 81 | rs6767484 | G | 0.12 | 0.01 | -0.01 | 0.01 |
| 82 | rs6785040 | C | -0.06 | 0.01 | 0.00 | 0.01 |
| 83 | rs6795735 | T | -0.06 | 0.01 | 0.01 | 0.01 |
| 84 | rs6878122 | A | -0.06 | 0.01 | 0.01 | 0.01 |
| 85 | rs6960043 | C | 0.06 | 0.01 | 0.00 | 0.01 |
| 86 | rs7144011 | T | 0.05 | 0.01 | -0.02 | 0.01 |
| 87 | rs7177055 | A | 0.06 | 0.01 | -0.01 | 0.01 |
| 88 | rs7240767 | C | 0.05 | 0.01 | 0.00 | 0.01 |
| 89 | rs72802358 | C | -0.12 | 0.01 | 0.04 | 0.02 |
| 90 | rs72892910 | T | 0.06 | 0.01 | 0.01 | 0.01 |
| 91 | rs735949 | C | -0.07 | 0.01 | 0.01 | 0.02 |
| 92 | rs753270 | C | 0.05 | 0.01 | -0.02 | 0.01 |
| 93 | rs7561798 | G | 0.04 | 0.01 | 0.00 | 0.01 |
| 94 | rs7572970 | G | 0.06 | 0.01 | 0.03 | 0.01 |
| 95 | rs7607777 | T | -0.14 | 0.01 | -0.03 | 0.02 |
| 96 | rs7674212 | T | -0.05 | 0.01 | 0.00 | 0.01 |
| 97 | rs7685296 | T | -0.05 | 0.01 | 0.02 | 0.01 |
| 98 | rs7729395 | T | 0.14 | 0.02 | 0.02 | 0.02 |
| 99 | rs7756992 | G | 0.13 | 0.01 | 0.01 | 0.01 |
| 100 | rs7786095 | G | -0.07 | 0.01 | 0.00 | 0.02 |
| 101 | rs780094 | C | 0.07 | 0.01 | -0.04 | 0.01 |
| 102 | rs7845219 | C | -0.04 | 0.01 | -0.02 | 0.01 |
| 103 | rs7903146 | T | 0.31 | 0.01 | -0.02 | 0.01 |
| 104 | rs7929543 | C | 0.08 | 0.01 | -0.03 | 0.02 |
| 105 | rs7955901 | T | -0.04 | 0.01 | -0.01 | 0.01 |
| 106 | rs8068804 | A | 0.06 | 0.01 | -0.01 | 0.01 |
| 107 | rs8108269 | G | 0.06 | 0.01 | 0.00 | 0.01 |
| 108 | rs825476 | T | 0.05 | 0.01 | -0.03 | 0.01 |
| 109 | rs840967 | A | -0.05 | 0.01 | 0.00 | 0.01 |
| 110 | rs849135 | A | -0.10 | 0.01 | 0.01 | 0.01 |
| 111 | rs853974 | C | -0.06 | 0.01 | 0.00 | 0.01 |
| 112 | rs9369425 | A | -0.05 | 0.01 | -0.02 | 0.01 |
| 113 | rs963740 | T | -0.05 | 0.01 | 0.02 | 0.01 |
| 114 | rs9844972 | C | 0.10 | 0.01 | 0.05 | 0.02 |
| 115 | rs9894220 | G | -0.06 | 0.01 | 0.01 | 0.01 |
| 116 | rs9928094 | G | 0.10 | 0.01 | 0.00 | 0.01 |
| 117 | rs993380 | G | -0.05 | 0.01 | 0.01 | 0.01 |
| 118 | rs9940149 | A | -0.06 | 0.01 | 0.03 | 0.02 |

T2DM: Type-2 diabetes; HDL: High-density lipoprotein.

Table 19. Genetic association estimates for the effect of T2DM on large HDL particle. ea=effect allele, gx=T2DM, gy= large HDL particle, se=standard error

|  | SNP | ea | gx | gx_se | gy | gy_se |
| --- | --- | --- | --- | --- | --- | --- |
| 1 | rs10077431 | A | -0.05 | 0.01 | 0.01 | 0.01 |
| 2 | rs10087241 | A | -0.05 | 0.01 | -0.02 | 0.01 |
| 3 | rs10100265 | C | -0.05 | 0.01 | 0.00 | 0.01 |
| 4 | rs10114341 | C | -0.04 | 0.01 | 0.00 | 0.01 |
| 5 | rs10401969 | C | 0.09 | 0.01 | 0.03 | 0.02 |
| 6 | rs1050226 | G | -0.05 | 0.01 | 0.01 | 0.01 |
| 7 | rs1061813 | A | -0.04 | 0.01 | 0.01 | 0.01 |
| 8 | rs1063355 | G | 0.07 | 0.01 | -0.01 | 0.01 |
| 9 | rs10740322 | A | 0.05 | 0.01 | 0.02 | 0.01 |
| 10 | rs10811661 | C | -0.16 | 0.01 | 0.00 | 0.01 |
| 11 | rs10830963 | G | 0.09 | 0.01 | 0.01 | 0.01 |
| 12 | rs10842994 | T | -0.08 | 0.01 | 0.00 | 0.01 |
| 13 | rs10974438 | C | 0.06 | 0.01 | 0.02 | 0.01 |
| 14 | rs11098676 | C | 0.05 | 0.01 | 0.01 | 0.01 |
| 15 | rs11107116 | T | 0.05 | 0.01 | -0.03 | 0.01 |
| 16 | rs1111875 | T | -0.09 | 0.01 | 0.02 | 0.01 |
| 17 | rs11257655 | T | 0.07 | 0.01 | 0.00 | 0.01 |
| 18 | rs1127655 | T | -0.04 | 0.01 | 0.01 | 0.01 |
| 19 | rs11708067 | G | -0.10 | 0.01 | 0.00 | 0.01 |
| 20 | rs11925227 | A | -0.05 | 0.01 | -0.01 | 0.01 |
| 21 | rs11926707 | C | 0.05 | 0.01 | -0.01 | 0.01 |
| 22 | rs12088739 | G | -0.09 | 0.01 | 0.01 | 0.02 |
| 23 | rs12299509 | G | 0.05 | 0.01 | 0.02 | 0.01 |
| 24 | rs12617659 | T | -0.07 | 0.01 | 0.00 | 0.01 |
| 25 | rs12910825 | G | 0.05 | 0.01 | -0.01 | 0.01 |
| 26 | rs12945601 | C | -0.05 | 0.01 | 0.02 | 0.01 |
| 27 | rs12970134 | A | 0.06 | 0.01 | -0.02 | 0.01 |
| 28 | rs13234269 | A | -0.06 | 0.01 | 0.02 | 0.01 |
| 29 | rs13239186 | T | 0.05 | 0.01 | 0.01 | 0.01 |
| 30 | rs13330951 | G | -0.05 | 0.01 | 0.01 | 0.01 |
| 31 | rs13389219 | T | -0.07 | 0.01 | 0.03 | 0.01 |
| 32 | rs1359790 | A | -0.08 | 0.01 | 0.00 | 0.01 |
| 33 | rs1496653 | G | -0.08 | 0.01 | 0.00 | 0.01 |
| 34 | rs1552224 | C | -0.10 | 0.01 | -0.01 | 0.01 |
| 35 | rs16988333 | G | -0.07 | 0.01 | 0.00 | 0.02 |
| 36 | rs17086692 | T | -0.05 | 0.01 | -0.02 | 0.01 |
| 37 | rs17168486 | T | 0.07 | 0.01 | 0.00 | 0.01 |
| 38 | rs17405722 | A | 0.09 | 0.01 | -0.01 | 0.02 |
| 39 | rs17411031 | G | -0.05 | 0.01 | 0.09 | 0.01 |
| 40 | rs1758632 | G | 0.05 | 0.01 | 0.00 | 0.01 |
| 41 | rs17631783 | T | -0.05 | 0.01 | -0.01 | 0.01 |
| 42 | rs17791513 | G | -0.10 | 0.01 | -0.01 | 0.02 |
| 43 | rs1801214 | T | 0.09 | 0.01 | -0.01 | 0.01 |
| 44 | rs1899951 | T | -0.11 | 0.01 | 0.02 | 0.01 |
| 45 | rs2058913 | T | -0.05 | 0.01 | -0.02 | 0.01 |
| 46 | rs2237892 | T | -0.10 | 0.02 | 0.00 | 0.02 |
| 47 | rs2246618 | T | 0.05 | 0.01 | -0.02 | 0.01 |
| 48 | rs2261181 | T | 0.10 | 0.01 | -0.01 | 0.02 |
| 49 | rs2294120 | G | -0.04 | 0.01 | 0.01 | 0.01 |
| 50 | rs2296173 | G | 0.07 | 0.01 | -0.01 | 0.01 |
| 51 | rs2299383 | T | 0.04 | 0.01 | -0.01 | 0.01 |
| 52 | rs243019 | C | 0.06 | 0.01 | 0.00 | 0.01 |
| 53 | rs2493394 | G | 0.07 | 0.01 | 0.01 | 0.02 |
| 54 | rs2796441 | A | -0.07 | 0.01 | 0.01 | 0.01 |
| 55 | rs2820426 | G | 0.05 | 0.01 | -0.01 | 0.01 |
| 56 | rs2867125 | C | 0.06 | 0.01 | -0.02 | 0.01 |
| 57 | rs2908282 | A | 0.06 | 0.01 | 0.00 | 0.02 |
| 58 | rs2925979 | C | -0.05 | 0.01 | 0.00 | 0.01 |
| 59 | rs2943656 | G | 0.09 | 0.01 | -0.04 | 0.01 |
| 60 | rs3217992 | T | 0.05 | 0.01 | 0.02 | 0.01 |
| 61 | rs340874 | C | 0.06 | 0.01 | -0.02 | 0.01 |
| 62 | rs348330 | A | -0.05 | 0.01 | 0.00 | 0.01 |
| 63 | rs3756784 | G | 0.05 | 0.01 | -0.01 | 0.01 |
| 64 | rs3802177 | A | -0.12 | 0.01 | 0.00 | 0.01 |
| 65 | rs459193 | G | 0.07 | 0.01 | -0.04 | 0.01 |
| 66 | rs4622883 | G | -0.04 | 0.01 | 0.01 | 0.01 |
| 67 | rs4686471 | C | 0.05 | 0.01 | -0.01 | 0.01 |
| 68 | rs4812829 | A | 0.05 | 0.01 | 0.00 | 0.01 |
| 69 | rs4823182 | G | 0.05 | 0.01 | -0.01 | 0.01 |
| 70 | rs4865796 | A | 0.05 | 0.01 | -0.02 | 0.01 |
| 71 | rs516946 | C | 0.08 | 0.01 | 0.00 | 0.01 |
| 72 | rs5215 | T | -0.07 | 0.01 | -0.02 | 0.01 |
| 73 | rs55966194 | G | -0.05 | 0.01 | 0.01 | 0.01 |
| 74 | rs576674 | A | -0.07 | 0.01 | 0.01 | 0.02 |
| 75 | rs6059662 | G | 0.04 | 0.01 | -0.02 | 0.01 |
| 76 | rs61953351 | T | -0.07 | 0.01 | -0.01 | 0.01 |
| 77 | rs622217 | C | -0.05 | 0.01 | 0.02 | 0.01 |
| 78 | rs6494307 | G | -0.04 | 0.01 | 0.01 | 0.01 |
| 79 | rs6515236 | C | -0.05 | 0.01 | 0.01 | 0.01 |
| 80 | rs67232546 | T | 0.06 | 0.01 | 0.03 | 0.01 |
| 81 | rs6767484 | G | 0.12 | 0.01 | -0.03 | 0.01 |
| 82 | rs6785040 | C | -0.06 | 0.01 | 0.00 | 0.01 |
| 83 | rs6795735 | T | -0.06 | 0.01 | 0.01 | 0.01 |
| 84 | rs6878122 | A | -0.06 | 0.01 | 0.01 | 0.01 |
| 85 | rs6960043 | C | 0.06 | 0.01 | 0.00 | 0.01 |
| 86 | rs7144011 | T | 0.05 | 0.01 | -0.01 | 0.01 |
| 87 | rs7177055 | A | 0.06 | 0.01 | -0.02 | 0.01 |
| 88 | rs7240767 | C | 0.05 | 0.01 | 0.00 | 0.01 |
| 89 | rs72802358 | C | -0.12 | 0.01 | 0.05 | 0.02 |
| 90 | rs72892910 | T | 0.06 | 0.01 | 0.00 | 0.01 |
| 91 | rs735949 | C | -0.07 | 0.01 | 0.02 | 0.02 |
| 92 | rs753270 | C | 0.05 | 0.01 | -0.01 | 0.01 |
| 93 | rs7561798 | G | 0.04 | 0.01 | -0.02 | 0.01 |
| 94 | rs7572970 | G | 0.06 | 0.01 | 0.03 | 0.01 |
| 95 | rs7607777 | T | -0.14 | 0.01 | 0.01 | 0.02 |
| 96 | rs7674212 | T | -0.05 | 0.01 | 0.02 | 0.01 |
| 97 | rs7685296 | T | -0.05 | 0.01 | 0.02 | 0.01 |
| 98 | rs7729395 | T | 0.14 | 0.02 | 0.03 | 0.02 |
| 99 | rs7756992 | G | 0.13 | 0.01 | -0.01 | 0.01 |
| 100 | rs7786095 | G | -0.07 | 0.01 | 0.02 | 0.02 |
| 101 | rs780094 | C | 0.07 | 0.01 | 0.03 | 0.01 |
| 102 | rs7845219 | C | -0.04 | 0.01 | -0.02 | 0.01 |
| 103 | rs7903146 | T | 0.31 | 0.01 | -0.03 | 0.01 |
| 104 | rs7929543 | C | 0.08 | 0.01 | -0.02 | 0.02 |
| 105 | rs7955901 | T | -0.04 | 0.01 | 0.00 | 0.01 |
| 106 | rs8068804 | A | 0.06 | 0.01 | 0.00 | 0.01 |
| 107 | rs8108269 | G | 0.06 | 0.01 | 0.01 | 0.01 |
| 108 | rs825476 | T | 0.05 | 0.01 | -0.03 | 0.01 |
| 109 | rs840967 | A | -0.05 | 0.01 | 0.00 | 0.01 |
| 110 | rs849135 | A | -0.10 | 0.01 | 0.02 | 0.01 |
| 111 | rs853974 | C | -0.06 | 0.01 | 0.00 | 0.01 |
| 112 | rs9369425 | A | -0.05 | 0.01 | -0.03 | 0.01 |
| 113 | rs963740 | T | -0.05 | 0.01 | 0.00 | 0.01 |
| 114 | rs9844972 | C | 0.10 | 0.01 | 0.00 | 0.02 |
| 115 | rs9894220 | G | -0.06 | 0.01 | 0.01 | 0.01 |
| 116 | rs9928094 | G | 0.10 | 0.01 | -0.01 | 0.01 |
| 117 | rs993380 | G | -0.05 | 0.01 | 0.01 | 0.01 |
| 118 | rs9940149 | A | -0.06 | 0.01 | 0.03 | 0.02 |

T2DM: Type-2 diabetes; HDL: High-density lipoprotein.

Table 20. Genetic association estimates for the effect of T2DM on very large HDL particle. ea=effect allele, gx=T2DM, gy= very large HDL particle, se=standard error

|  | SNP | ea | gx | gx_se | gy | gy_se |
| --- | --- | --- | --- | --- | --- | --- |
| 1 | rs10077431 | A | -0.05 | 0.01 | 0.00 | 0.01 |
| 2 | rs10087241 | A | -0.05 | 0.01 | -0.01 | 0.01 |
| 3 | rs10100265 | C | -0.05 | 0.01 | 0.01 | 0.01 |
| 4 | rs10114341 | C | -0.04 | 0.01 | 0.01 | 0.01 |
| 5 | rs10401969 | C | 0.09 | 0.01 | 0.01 | 0.02 |
| 6 | rs1050226 | G | -0.05 | 0.01 | 0.01 | 0.01 |
| 7 | rs1061813 | A | -0.04 | 0.01 | 0.00 | 0.01 |
| 8 | rs1063355 | G | 0.07 | 0.01 | -0.01 | 0.01 |
| 9 | rs10740322 | A | 0.05 | 0.01 | 0.02 | 0.01 |
| 10 | rs10811661 | C | -0.16 | 0.01 | 0.00 | 0.01 |
| 11 | rs10830963 | G | 0.09 | 0.01 | 0.00 | 0.01 |
| 12 | rs10842994 | T | -0.08 | 0.01 | 0.00 | 0.01 |
| 13 | rs10974438 | C | 0.06 | 0.01 | 0.02 | 0.01 |
| 14 | rs11098676 | C | 0.05 | 0.01 | 0.00 | 0.01 |
| 15 | rs11107116 | T | 0.05 | 0.01 | -0.02 | 0.01 |
| 16 | rs1111875 | T | -0.09 | 0.01 | 0.01 | 0.01 |
| 17 | rs11257655 | T | 0.07 | 0.01 | 0.02 | 0.01 |
| 18 | rs1127655 | T | -0.04 | 0.01 | 0.00 | 0.01 |
| 19 | rs11708067 | G | -0.10 | 0.01 | -0.01 | 0.01 |
| 20 | rs11925227 | A | -0.05 | 0.01 | -0.01 | 0.01 |
| 21 | rs11926707 | C | 0.05 | 0.01 | 0.02 | 0.01 |
| 22 | rs12088739 | G | -0.09 | 0.01 | 0.03 | 0.02 |
| 23 | rs12299509 | G | 0.05 | 0.01 | 0.01 | 0.01 |
| 24 | rs12617659 | T | -0.07 | 0.01 | 0.00 | 0.01 |
| 25 | rs12910825 | G | 0.05 | 0.01 | -0.01 | 0.01 |
| 26 | rs12945601 | C | -0.05 | 0.01 | 0.01 | 0.01 |
| 27 | rs12970134 | A | 0.06 | 0.01 | -0.01 | 0.01 |
| 28 | rs13234269 | A | -0.06 | 0.01 | 0.02 | 0.01 |
| 29 | rs13239186 | T | 0.05 | 0.01 | 0.00 | 0.01 |
| 30 | rs13330951 | G | -0.05 | 0.01 | 0.00 | 0.01 |
| 31 | rs13389219 | T | -0.07 | 0.01 | 0.03 | 0.01 |
| 32 | rs1359790 | A | -0.08 | 0.01 | 0.00 | 0.01 |
| 33 | rs1496653 | G | -0.08 | 0.01 | 0.00 | 0.01 |
| 34 | rs1552224 | C | -0.10 | 0.01 | -0.01 | 0.01 |
| 35 | rs16988333 | G | -0.07 | 0.01 | -0.02 | 0.02 |
| 36 | rs17086692 | T | -0.05 | 0.01 | -0.01 | 0.01 |
| 37 | rs17168486 | T | 0.07 | 0.01 | 0.01 | 0.01 |
| 38 | rs17405722 | A | 0.09 | 0.01 | -0.02 | 0.02 |
| 39 | rs17411031 | G | -0.05 | 0.01 | 0.05 | 0.01 |
| 40 | rs1758632 | G | 0.05 | 0.01 | 0.00 | 0.01 |
| 41 | rs17631783 | T | -0.05 | 0.01 | -0.01 | 0.01 |
| 42 | rs17791513 | G | -0.10 | 0.01 | -0.01 | 0.02 |
| 43 | rs1801214 | T | 0.09 | 0.01 | -0.02 | 0.01 |
| 44 | rs1899951 | T | -0.11 | 0.01 | 0.00 | 0.01 |
| 45 | rs2058913 | T | -0.05 | 0.01 | -0.03 | 0.01 |
| 46 | rs2237892 | T | -0.10 | 0.02 | 0.03 | 0.02 |
| 47 | rs2246618 | T | 0.05 | 0.01 | -0.02 | 0.01 |
| 48 | rs2261181 | T | 0.10 | 0.01 | 0.00 | 0.02 |
| 49 | rs2294120 | G | -0.04 | 0.01 | 0.02 | 0.01 |
| 50 | rs2296173 | G | 0.07 | 0.01 | 0.00 | 0.01 |
| 51 | rs2299383 | T | 0.04 | 0.01 | -0.01 | 0.01 |
| 52 | rs243019 | C | 0.06 | 0.01 | 0.00 | 0.01 |
| 53 | rs2493394 | G | 0.07 | 0.01 | 0.02 | 0.02 |
| 54 | rs2796441 | A | -0.07 | 0.01 | 0.02 | 0.01 |
| 55 | rs2820426 | G | 0.05 | 0.01 | -0.02 | 0.01 |
| 56 | rs2867125 | C | 0.06 | 0.01 | -0.01 | 0.01 |
| 57 | rs2908282 | A | 0.06 | 0.01 | 0.01 | 0.02 |
| 58 | rs2925979 | C | -0.05 | 0.01 | 0.00 | 0.01 |
| 59 | rs2943656 | G | 0.09 | 0.01 | -0.02 | 0.01 |
| 60 | rs3217992 | T | 0.05 | 0.01 | 0.02 | 0.01 |
| 61 | rs340874 | C | 0.06 | 0.01 | -0.01 | 0.01 |
| 62 | rs348330 | A | -0.05 | 0.01 | -0.01 | 0.01 |
| 63 | rs3756784 | G | 0.05 | 0.01 | 0.01 | 0.01 |
| 64 | rs3802177 | A | -0.12 | 0.01 | 0.01 | 0.01 |
| 65 | rs459193 | G | 0.07 | 0.01 | -0.03 | 0.01 |
| 66 | rs4622883 | G | -0.04 | 0.01 | 0.01 | 0.01 |
| 67 | rs4686471 | C | 0.05 | 0.01 | -0.01 | 0.01 |
| 68 | rs4812829 | A | 0.05 | 0.01 | -0.01 | 0.01 |
| 69 | rs4823182 | G | 0.05 | 0.01 | -0.03 | 0.01 |
| 70 | rs4865796 | A | 0.05 | 0.01 | -0.03 | 0.01 |
| 71 | rs516946 | C | 0.08 | 0.01 | 0.02 | 0.01 |
| 72 | rs5215 | T | -0.07 | 0.01 | -0.01 | 0.01 |
| 73 | rs55966194 | G | -0.05 | 0.01 | 0.01 | 0.01 |
| 74 | rs576674 | A | -0.07 | 0.01 | -0.02 | 0.02 |
| 75 | rs6059662 | G | 0.04 | 0.01 | -0.01 | 0.01 |
| 76 | rs61953351 | T | -0.07 | 0.01 | -0.01 | 0.01 |
| 77 | rs622217 | C | -0.05 | 0.01 | 0.02 | 0.01 |
| 78 | rs6494307 | G | -0.04 | 0.01 | 0.01 | 0.01 |
| 79 | rs6515236 | C | -0.05 | 0.01 | -0.01 | 0.01 |
| 80 | rs67232546 | T | 0.06 | 0.01 | 0.04 | 0.01 |
| 81 | rs6767484 | G | 0.12 | 0.01 | -0.02 | 0.01 |
| 82 | rs6785040 | C | -0.06 | 0.01 | 0.01 | 0.01 |
| 83 | rs6795735 | T | -0.06 | 0.01 | 0.00 | 0.01 |
| 84 | rs6878122 | A | -0.06 | 0.01 | 0.00 | 0.01 |
| 85 | rs6960043 | C | 0.06 | 0.01 | 0.00 | 0.01 |
| 86 | rs7144011 | T | 0.05 | 0.01 | 0.00 | 0.01 |
| 87 | rs7177055 | A | 0.06 | 0.01 | -0.01 | 0.01 |
| 88 | rs7240767 | C | 0.05 | 0.01 | 0.00 | 0.01 |
| 89 | rs72802358 | C | -0.12 | 0.01 | 0.02 | 0.02 |
| 90 | rs72892910 | T | 0.06 | 0.01 | 0.01 | 0.01 |
| 91 | rs735949 | C | -0.07 | 0.01 | 0.02 | 0.02 |
| 92 | rs753270 | C | 0.05 | 0.01 | 0.00 | 0.01 |
| 93 | rs7561798 | G | 0.04 | 0.01 | -0.01 | 0.01 |
| 94 | rs7572970 | G | 0.06 | 0.01 | 0.01 | 0.01 |
| 95 | rs7607777 | T | -0.14 | 0.01 | 0.02 | 0.02 |
| 96 | rs7674212 | T | -0.05 | 0.01 | 0.00 | 0.01 |
| 97 | rs7685296 | T | -0.05 | 0.01 | 0.01 | 0.01 |
| 98 | rs7729395 | T | 0.14 | 0.02 | 0.05 | 0.02 |
| 99 | rs7756992 | G | 0.13 | 0.01 | 0.00 | 0.01 |
| 100 | rs7786095 | G | -0.07 | 0.01 | 0.02 | 0.02 |
| 101 | rs780094 | C | 0.07 | 0.01 | 0.01 | 0.01 |
| 102 | rs7845219 | C | -0.04 | 0.01 | -0.01 | 0.01 |
| 103 | rs7903146 | T | 0.31 | 0.01 | -0.02 | 0.01 |
| 104 | rs7929543 | C | 0.08 | 0.01 | 0.01 | 0.02 |
| 105 | rs7955901 | T | -0.04 | 0.01 | 0.01 | 0.01 |
| 106 | rs8068804 | A | 0.06 | 0.01 | 0.01 | 0.01 |
| 107 | rs8108269 | G | 0.06 | 0.01 | 0.02 | 0.01 |
| 108 | rs825476 | T | 0.05 | 0.01 | 0.00 | 0.01 |
| 109 | rs840967 | A | -0.05 | 0.01 | 0.00 | 0.01 |
| 110 | rs849135 | A | -0.10 | 0.01 | 0.00 | 0.01 |
| 111 | rs853974 | C | -0.06 | 0.01 | 0.01 | 0.01 |
| 112 | rs9369425 | A | -0.05 | 0.01 | -0.01 | 0.01 |
| 113 | rs963740 | T | -0.05 | 0.01 | -0.01 | 0.01 |
| 114 | rs9844972 | C | 0.10 | 0.01 | -0.03 | 0.02 |
| 115 | rs9894220 | G | -0.06 | 0.01 | 0.01 | 0.01 |
| 116 | rs9928094 | G | 0.10 | 0.01 | 0.00 | 0.01 |
| 117 | rs993380 | G | -0.05 | 0.01 | 0.00 | 0.01 |
| 118 | rs9940149 | A | -0.06 | 0.01 | 0.03 | 0.02 |

T2DM: Type-2 diabetes; HDL: High-density lipoprotein.

Table 21. Genetic association estimates for the effect of T2DM on small LDL particle. ea=effect allele, gx=T2DM, gy= small LDL particle, se=standard error

|  | SNP | ea | gx | gx_se | gy | gy_se |
| --- | --- | --- | --- | --- | --- | --- |
| 1 | rs10077431 | A | -0.05 | 0.01 | -0.03 | 0.01 |
| 2 | rs10087241 | A | -0.05 | 0.01 | -0.01 | 0.01 |
| 3 | rs10100265 | C | -0.05 | 0.01 | 0.02 | 0.01 |
| 4 | rs10114341 | C | -0.04 | 0.01 | 0.00 | 0.01 |
| 5 | rs10401969 | C | 0.09 | 0.01 | -0.12 | 0.02 |
| 6 | rs1050226 | G | -0.05 | 0.01 | -0.01 | 0.01 |
| 7 | rs1061813 | A | -0.04 | 0.01 | -0.01 | 0.01 |
| 8 | rs1063355 | G | 0.07 | 0.01 | -0.02 | 0.01 |
| 9 | rs10740322 | A | 0.05 | 0.01 | 0.01 | 0.01 |
| 10 | rs10811661 | C | -0.16 | 0.01 | 0.01 | 0.01 |
| 11 | rs10830963 | G | 0.09 | 0.01 | 0.00 | 0.01 |
| 12 | rs10842994 | T | -0.08 | 0.01 | -0.01 | 0.01 |
| 13 | rs10974438 | C | 0.06 | 0.01 | 0.01 | 0.01 |
| 14 | rs11098676 | C | 0.05 | 0.01 | 0.01 | 0.01 |
| 15 | rs11107116 | T | 0.05 | 0.01 | 0.02 | 0.01 |
| 16 | rs1111875 | T | -0.09 | 0.01 | -0.01 | 0.01 |
| 17 | rs11257655 | T | 0.07 | 0.01 | 0.01 | 0.01 |
| 18 | rs1127655 | T | -0.04 | 0.01 | -0.01 | 0.01 |
| 19 | rs11708067 | G | -0.10 | 0.01 | -0.01 | 0.01 |
| 20 | rs11925227 | A | -0.05 | 0.01 | 0.00 | 0.01 |
| 21 | rs11926707 | C | 0.05 | 0.01 | 0.02 | 0.01 |
| 22 | rs12088739 | G | -0.09 | 0.01 | 0.02 | 0.02 |
| 23 | rs12299509 | G | 0.05 | 0.01 | 0.01 | 0.01 |
| 24 | rs12617659 | T | -0.07 | 0.01 | 0.01 | 0.01 |
| 25 | rs12910825 | G | 0.05 | 0.01 | -0.01 | 0.01 |
| 26 | rs12945601 | C | -0.05 | 0.01 | 0.00 | 0.01 |
| 27 | rs12970134 | A | 0.06 | 0.01 | 0.02 | 0.01 |
| 28 | rs13234269 | A | -0.06 | 0.01 | -0.01 | 0.01 |
| 29 | rs13239186 | T | 0.05 | 0.01 | -0.01 | 0.01 |
| 30 | rs13330951 | G | -0.05 | 0.01 | 0.00 | 0.01 |
| 31 | rs13389219 | T | -0.07 | 0.01 | -0.01 | 0.01 |
| 32 | rs1359790 | A | -0.08 | 0.01 | 0.02 | 0.01 |
| 33 | rs1496653 | G | -0.08 | 0.01 | 0.01 | 0.01 |
| 34 | rs1552224 | C | -0.10 | 0.01 | -0.01 | 0.01 |
| 35 | rs16988333 | G | -0.07 | 0.01 | 0.00 | 0.02 |
| 36 | rs17086692 | T | -0.05 | 0.01 | 0.01 | 0.01 |
| 37 | rs17168486 | T | 0.07 | 0.01 | 0.01 | 0.01 |
| 38 | rs17405722 | A | 0.09 | 0.01 | 0.01 | 0.02 |
| 39 | rs17411031 | G | -0.05 | 0.01 | -0.03 | 0.01 |
| 40 | rs1758632 | G | 0.05 | 0.01 | -0.01 | 0.01 |
| 41 | rs17631783 | T | -0.05 | 0.01 | -0.01 | 0.01 |
| 42 | rs17791513 | G | -0.10 | 0.01 | 0.00 | 0.02 |
| 43 | rs1801214 | T | 0.09 | 0.01 | 0.02 | 0.01 |
| 44 | rs1899951 | T | -0.11 | 0.01 | -0.01 | 0.01 |
| 45 | rs2058913 | T | -0.05 | 0.01 | 0.00 | 0.01 |
| 46 | rs2237892 | T | -0.10 | 0.02 | -0.01 | 0.02 |
| 47 | rs2246618 | T | 0.05 | 0.01 | 0.01 | 0.01 |
| 48 | rs2261181 | T | 0.10 | 0.01 | 0.01 | 0.02 |
| 49 | rs2294120 | G | -0.04 | 0.01 | 0.00 | 0.01 |
| 50 | rs2296173 | G | 0.07 | 0.01 | 0.01 | 0.01 |
| 51 | rs2299383 | T | 0.04 | 0.01 | 0.00 | 0.01 |
| 52 | rs243019 | C | 0.06 | 0.01 | -0.01 | 0.01 |
| 53 | rs2493394 | G | 0.07 | 0.01 | 0.03 | 0.02 |
| 54 | rs2796441 | A | -0.07 | 0.01 | 0.01 | 0.01 |
| 55 | rs2820426 | G | 0.05 | 0.01 | 0.02 | 0.01 |
| 56 | rs2867125 | C | 0.06 | 0.01 | 0.01 | 0.01 |
| 57 | rs2908282 | A | 0.06 | 0.01 | -0.02 | 0.02 |
| 58 | rs2925979 | C | -0.05 | 0.01 | 0.01 | 0.01 |
| 59 | rs2943656 | G | 0.09 | 0.01 | 0.01 | 0.01 |
| 60 | rs3217992 | T | 0.05 | 0.01 | 0.00 | 0.01 |
| 61 | rs340874 | C | 0.06 | 0.01 | 0.01 | 0.01 |
| 62 | rs348330 | A | -0.05 | 0.01 | -0.01 | 0.01 |
| 63 | rs3756784 | G | 0.05 | 0.01 | -0.02 | 0.01 |
| 64 | rs3802177 | A | -0.12 | 0.01 | 0.00 | 0.01 |
| 65 | rs459193 | G | 0.07 | 0.01 | 0.00 | 0.01 |
| 66 | rs4622883 | G | -0.04 | 0.01 | 0.00 | 0.01 |
| 67 | rs4686471 | C | 0.05 | 0.01 | -0.01 | 0.01 |
| 68 | rs4812829 | A | 0.05 | 0.01 | -0.01 | 0.01 |
| 69 | rs4823182 | G | 0.05 | 0.01 | 0.00 | 0.01 |
| 70 | rs4865796 | A | 0.05 | 0.01 | 0.01 | 0.01 |
| 71 | rs516946 | C | 0.08 | 0.01 | 0.00 | 0.01 |
| 72 | rs5215 | T | -0.07 | 0.01 | -0.01 | 0.01 |
| 73 | rs55966194 | G | -0.05 | 0.01 | -0.01 | 0.01 |
| 74 | rs576674 | A | -0.07 | 0.01 | -0.01 | 0.02 |
| 75 | rs6059662 | G | 0.04 | 0.01 | 0.00 | 0.01 |
| 76 | rs61953351 | T | -0.07 | 0.01 | -0.02 | 0.01 |
| 77 | rs622217 | C | -0.05 | 0.01 | -0.02 | 0.01 |
| 78 | rs6494307 | G | -0.04 | 0.01 | 0.01 | 0.01 |
| 79 | rs6515236 | C | -0.05 | 0.01 | 0.00 | 0.01 |
| 80 | rs67232546 | T | 0.06 | 0.01 | 0.02 | 0.01 |
| 81 | rs6767484 | G | 0.12 | 0.01 | 0.01 | 0.01 |
| 82 | rs6785040 | C | -0.06 | 0.01 | -0.01 | 0.01 |
| 83 | rs6795735 | T | -0.06 | 0.01 | 0.00 | 0.01 |
| 84 | rs6878122 | A | -0.06 | 0.01 | -0.03 | 0.01 |
| 85 | rs6960043 | C | 0.06 | 0.01 | -0.01 | 0.01 |
| 86 | rs7144011 | T | 0.05 | 0.01 | 0.00 | 0.01 |
| 87 | rs7177055 | A | 0.06 | 0.01 | 0.01 | 0.01 |
| 88 | rs7240767 | C | 0.05 | 0.01 | 0.01 | 0.01 |
| 89 | rs72802358 | C | -0.12 | 0.01 | 0.00 | 0.02 |
| 90 | rs72892910 | T | 0.06 | 0.01 | 0.02 | 0.01 |
| 91 | rs735949 | C | -0.07 | 0.01 | 0.00 | 0.02 |
| 92 | rs753270 | C | 0.05 | 0.01 | 0.02 | 0.01 |
| 93 | rs7561798 | G | 0.04 | 0.01 | 0.00 | 0.01 |
| 94 | rs7572970 | G | 0.06 | 0.01 | -0.01 | 0.01 |
| 95 | rs7607777 | T | -0.14 | 0.01 | -0.05 | 0.02 |
| 96 | rs7674212 | T | -0.05 | 0.01 | -0.02 | 0.01 |
| 97 | rs7685296 | T | -0.05 | 0.01 | 0.00 | 0.01 |
| 98 | rs7729395 | T | 0.14 | 0.02 | -0.01 | 0.02 |
| 99 | rs7756992 | G | 0.13 | 0.01 | 0.00 | 0.01 |
| 100 | rs7786095 | G | -0.07 | 0.01 | -0.02 | 0.02 |
| 101 | rs780094 | C | 0.07 | 0.01 | -0.06 | 0.01 |
| 102 | rs7845219 | C | -0.04 | 0.01 | 0.01 | 0.01 |
| 103 | rs7903146 | T | 0.31 | 0.01 | 0.01 | 0.01 |
| 104 | rs7929543 | C | 0.08 | 0.01 | 0.00 | 0.02 |
| 105 | rs7955901 | T | -0.04 | 0.01 | 0.02 | 0.01 |
| 106 | rs8068804 | A | 0.06 | 0.01 | 0.00 | 0.01 |
| 107 | rs8108269 | G | 0.06 | 0.01 | 0.02 | 0.01 |
| 108 | rs825476 | T | 0.05 | 0.01 | 0.02 | 0.01 |
| 109 | rs840967 | A | -0.05 | 0.01 | -0.01 | 0.01 |
| 110 | rs849135 | A | -0.10 | 0.01 | -0.01 | 0.01 |
| 111 | rs853974 | C | -0.06 | 0.01 | 0.02 | 0.01 |
| 112 | rs9369425 | A | -0.05 | 0.01 | 0.02 | 0.01 |
| 113 | rs963740 | T | -0.05 | 0.01 | -0.01 | 0.01 |
| 114 | rs9844972 | C | 0.10 | 0.01 | 0.00 | 0.02 |
| 115 | rs9894220 | G | -0.06 | 0.01 | 0.00 | 0.01 |
| 116 | rs9928094 | G | 0.10 | 0.01 | 0.02 | 0.01 |
| 117 | rs993380 | G | -0.05 | 0.01 | -0.01 | 0.01 |
| 118 | rs9940149 | A | -0.06 | 0.01 | 0.03 | 0.02 |

T2DM: Type-2 diabetes; LDL: Low-density lipoprotein.

Table 22. Genetic association estimates for the effect of T2DM on medium LDL particle. ea=effect allele, gx=T2DM, gy= medium LDL particle, se=standard error

|  | SNP | ea | gx | gx_se | gy | gy_se |
| --- | --- | --- | --- | --- | --- | --- |
| 1 | rs10077431 | A | -0.05 | 0.01 | -0.03 | 0.01 |
| 2 | rs10087241 | A | -0.05 | 0.01 | -0.01 | 0.01 |
| 3 | rs10100265 | C | -0.05 | 0.01 | 0.02 | 0.01 |
| 4 | rs10114341 | C | -0.04 | 0.01 | -0.01 | 0.01 |
| 5 | rs10401969 | C | 0.09 | 0.01 | -0.10 | 0.02 |
| 6 | rs1050226 | G | -0.05 | 0.01 | -0.01 | 0.01 |
| 7 | rs1061813 | A | -0.04 | 0.01 | -0.01 | 0.01 |
| 8 | rs1063355 | G | 0.07 | 0.01 | -0.02 | 0.01 |
| 9 | rs10740322 | A | 0.05 | 0.01 | 0.01 | 0.01 |
| 10 | rs10811661 | C | -0.16 | 0.01 | 0.01 | 0.01 |
| 11 | rs10830963 | G | 0.09 | 0.01 | 0.00 | 0.01 |
| 12 | rs10842994 | T | -0.08 | 0.01 | -0.01 | 0.01 |
| 13 | rs10974438 | C | 0.06 | 0.01 | 0.01 | 0.01 |
| 14 | rs11098676 | C | 0.05 | 0.01 | 0.01 | 0.01 |
| 15 | rs11107116 | T | 0.05 | 0.01 | 0.01 | 0.01 |
| 16 | rs1111875 | T | -0.09 | 0.01 | -0.02 | 0.01 |
| 17 | rs11257655 | T | 0.07 | 0.01 | 0.01 | 0.01 |
| 18 | rs1127655 | T | -0.04 | 0.01 | -0.01 | 0.01 |
| 19 | rs11708067 | G | -0.10 | 0.01 | -0.01 | 0.01 |
| 20 | rs11925227 | A | -0.05 | 0.01 | 0.01 | 0.01 |
| 21 | rs11926707 | C | 0.05 | 0.01 | 0.01 | 0.01 |
| 22 | rs12088739 | G | -0.09 | 0.01 | 0.02 | 0.02 |
| 23 | rs12299509 | G | 0.05 | 0.01 | 0.01 | 0.01 |
| 24 | rs12617659 | T | -0.07 | 0.01 | 0.01 | 0.01 |
| 25 | rs12910825 | G | 0.05 | 0.01 | -0.01 | 0.01 |
| 26 | rs12945601 | C | -0.05 | 0.01 | 0.01 | 0.01 |
| 27 | rs12970134 | A | 0.06 | 0.01 | 0.02 | 0.01 |
| 28 | rs13234269 | A | -0.06 | 0.01 | -0.01 | 0.01 |
| 29 | rs13239186 | T | 0.05 | 0.01 | -0.01 | 0.01 |
| 30 | rs13330951 | G | -0.05 | 0.01 | 0.00 | 0.01 |
| 31 | rs13389219 | T | -0.07 | 0.01 | -0.01 | 0.01 |
| 32 | rs1359790 | A | -0.08 | 0.01 | 0.02 | 0.01 |
| 33 | rs1496653 | G | -0.08 | 0.01 | 0.01 | 0.01 |
| 34 | rs1552224 | C | -0.10 | 0.01 | -0.01 | 0.01 |
| 35 | rs16988333 | G | -0.07 | 0.01 | 0.00 | 0.02 |
| 36 | rs17086692 | T | -0.05 | 0.01 | 0.01 | 0.01 |
| 37 | rs17168486 | T | 0.07 | 0.01 | 0.01 | 0.01 |
| 38 | rs17405722 | A | 0.09 | 0.01 | 0.02 | 0.02 |
| 39 | rs17411031 | G | -0.05 | 0.01 | -0.03 | 0.01 |
| 40 | rs1758632 | G | 0.05 | 0.01 | -0.01 | 0.01 |
| 41 | rs17631783 | T | -0.05 | 0.01 | -0.01 | 0.01 |
| 42 | rs17791513 | G | -0.10 | 0.01 | 0.00 | 0.02 |
| 43 | rs1801214 | T | 0.09 | 0.01 | 0.02 | 0.01 |
| 44 | rs1899951 | T | -0.11 | 0.01 | -0.01 | 0.01 |
| 45 | rs2058913 | T | -0.05 | 0.01 | 0.00 | 0.01 |
| 46 | rs2237892 | T | -0.10 | 0.02 | -0.01 | 0.02 |
| 47 | rs2246618 | T | 0.05 | 0.01 | 0.01 | 0.01 |
| 48 | rs2261181 | T | 0.10 | 0.01 | 0.01 | 0.02 |
| 49 | rs2294120 | G | -0.04 | 0.01 | 0.00 | 0.01 |
| 50 | rs2296173 | G | 0.07 | 0.01 | 0.01 | 0.01 |
| 51 | rs2299383 | T | 0.04 | 0.01 | 0.01 | 0.01 |
| 52 | rs243019 | C | 0.06 | 0.01 | -0.01 | 0.01 |
| 53 | rs2493394 | G | 0.07 | 0.01 | 0.03 | 0.02 |
| 54 | rs2796441 | A | -0.07 | 0.01 | 0.01 | 0.01 |
| 55 | rs2820426 | G | 0.05 | 0.01 | 0.02 | 0.01 |
| 56 | rs2867125 | C | 0.06 | 0.01 | 0.01 | 0.01 |
| 57 | rs2908282 | A | 0.06 | 0.01 | -0.02 | 0.02 |
| 58 | rs2925979 | C | -0.05 | 0.01 | 0.00 | 0.01 |
| 59 | rs2943656 | G | 0.09 | 0.01 | 0.01 | 0.01 |
| 60 | rs3217992 | T | 0.05 | 0.01 | 0.00 | 0.01 |
| 61 | rs340874 | C | 0.06 | 0.01 | 0.01 | 0.01 |
| 62 | rs348330 | A | -0.05 | 0.01 | -0.01 | 0.01 |
| 63 | rs3756784 | G | 0.05 | 0.01 | -0.02 | 0.01 |
| 64 | rs3802177 | A | -0.12 | 0.01 | -0.01 | 0.01 |
| 65 | rs459193 | G | 0.07 | 0.01 | 0.00 | 0.01 |
| 66 | rs4622883 | G | -0.04 | 0.01 | 0.00 | 0.01 |
| 67 | rs4686471 | C | 0.05 | 0.01 | -0.01 | 0.01 |
| 68 | rs4812829 | A | 0.05 | 0.01 | -0.01 | 0.01 |
| 69 | rs4823182 | G | 0.05 | 0.01 | 0.01 | 0.01 |
| 70 | rs4865796 | A | 0.05 | 0.01 | 0.01 | 0.01 |
| 71 | rs516946 | C | 0.08 | 0.01 | 0.00 | 0.01 |
| 72 | rs5215 | T | -0.07 | 0.01 | -0.01 | 0.01 |
| 73 | rs55966194 | G | -0.05 | 0.01 | -0.01 | 0.01 |
| 74 | rs576674 | A | -0.07 | 0.01 | 0.00 | 0.02 |
| 75 | rs6059662 | G | 0.04 | 0.01 | 0.00 | 0.01 |
| 76 | rs61953351 | T | -0.07 | 0.01 | -0.02 | 0.01 |
| 77 | rs622217 | C | -0.05 | 0.01 | -0.02 | 0.01 |
| 78 | rs6494307 | G | -0.04 | 0.01 | 0.01 | 0.01 |
| 79 | rs6515236 | C | -0.05 | 0.01 | 0.00 | 0.01 |
| 80 | rs67232546 | T | 0.06 | 0.01 | 0.02 | 0.01 |
| 81 | rs6767484 | G | 0.12 | 0.01 | 0.00 | 0.01 |
| 82 | rs6785040 | C | -0.06 | 0.01 | -0.01 | 0.01 |
| 83 | rs6795735 | T | -0.06 | 0.01 | 0.00 | 0.01 |
| 84 | rs6878122 | A | -0.06 | 0.01 | -0.03 | 0.01 |
| 85 | rs6960043 | C | 0.06 | 0.01 | 0.00 | 0.01 |
| 86 | rs7144011 | T | 0.05 | 0.01 | 0.00 | 0.01 |
| 87 | rs7177055 | A | 0.06 | 0.01 | 0.01 | 0.01 |
| 88 | rs7240767 | C | 0.05 | 0.01 | 0.01 | 0.01 |
| 89 | rs72802358 | C | -0.12 | 0.01 | 0.00 | 0.02 |
| 90 | rs72892910 | T | 0.06 | 0.01 | 0.01 | 0.01 |
| 91 | rs735949 | C | -0.07 | 0.01 | 0.00 | 0.02 |
| 92 | rs753270 | C | 0.05 | 0.01 | 0.02 | 0.01 |
| 93 | rs7561798 | G | 0.04 | 0.01 | 0.00 | 0.01 |
| 94 | rs7572970 | G | 0.06 | 0.01 | -0.01 | 0.01 |
| 95 | rs7607777 | T | -0.14 | 0.01 | -0.05 | 0.02 |
| 96 | rs7674212 | T | -0.05 | 0.01 | -0.01 | 0.01 |
| 97 | rs7685296 | T | -0.05 | 0.01 | 0.00 | 0.01 |
| 98 | rs7729395 | T | 0.14 | 0.02 | -0.02 | 0.02 |
| 99 | rs7756992 | G | 0.13 | 0.01 | -0.01 | 0.01 |
| 100 | rs7786095 | G | -0.07 | 0.01 | -0.03 | 0.02 |
| 101 | rs780094 | C | 0.07 | 0.01 | -0.05 | 0.01 |
| 102 | rs7845219 | C | -0.04 | 0.01 | 0.01 | 0.01 |
| 103 | rs7903146 | T | 0.31 | 0.01 | 0.01 | 0.01 |
| 104 | rs7929543 | C | 0.08 | 0.01 | 0.00 | 0.02 |
| 105 | rs7955901 | T | -0.04 | 0.01 | 0.01 | 0.01 |
| 106 | rs8068804 | A | 0.06 | 0.01 | 0.00 | 0.01 |
| 107 | rs8108269 | G | 0.06 | 0.01 | 0.02 | 0.01 |
| 108 | rs825476 | T | 0.05 | 0.01 | 0.02 | 0.01 |
| 109 | rs840967 | A | -0.05 | 0.01 | -0.01 | 0.01 |
| 110 | rs849135 | A | -0.10 | 0.01 | 0.00 | 0.01 |
| 111 | rs853974 | C | -0.06 | 0.01 | 0.02 | 0.01 |
| 112 | rs9369425 | A | -0.05 | 0.01 | 0.01 | 0.01 |
| 113 | rs963740 | T | -0.05 | 0.01 | -0.01 | 0.01 |
| 114 | rs9844972 | C | 0.10 | 0.01 | -0.01 | 0.02 |
| 115 | rs9894220 | G | -0.06 | 0.01 | 0.00 | 0.01 |
| 116 | rs9928094 | G | 0.10 | 0.01 | 0.02 | 0.01 |
| 117 | rs993380 | G | -0.05 | 0.01 | 0.00 | 0.01 |
| 118 | rs9940149 | A | -0.06 | 0.01 | 0.02 | 0.02 |

T2DM: Type-2 diabetes; LDL: Low-density lipoprotein.

Table 23. Genetic association estimates for the effect of T2DM on large LDL particle. ea=effect allele, gx=T2DM, gy= large LDL particle, se=standard error

|  | SNP | ea | gx | gx_se | gy | gy_se |
| --- | --- | --- | --- | --- | --- | --- |
| 1 | rs10077431 | A | -0.05 | 0.01 | -0.03 | 0.01 |
| 2 | rs10087241 | A | -0.05 | 0.01 | -0.01 | 0.01 |
| 3 | rs10100265 | C | -0.05 | 0.01 | 0.01 | 0.01 |
| 4 | rs10114341 | C | -0.04 | 0.01 | -0.01 | 0.01 |
| 5 | rs10401969 | C | 0.09 | 0.01 | -0.10 | 0.02 |
| 6 | rs1050226 | G | -0.05 | 0.01 | 0.00 | 0.01 |
| 7 | rs1061813 | A | -0.04 | 0.01 | -0.01 | 0.01 |
| 8 | rs1063355 | G | 0.07 | 0.01 | -0.02 | 0.01 |
| 9 | rs10740322 | A | 0.05 | 0.01 | 0.01 | 0.01 |
| 10 | rs10811661 | C | -0.16 | 0.01 | 0.01 | 0.01 |
| 11 | rs10830963 | G | 0.09 | 0.01 | 0.00 | 0.01 |
| 12 | rs10842994 | T | -0.08 | 0.01 | -0.01 | 0.01 |
| 13 | rs10974438 | C | 0.06 | 0.01 | 0.01 | 0.01 |
| 14 | rs11098676 | C | 0.05 | 0.01 | 0.01 | 0.01 |
| 15 | rs11107116 | T | 0.05 | 0.01 | 0.01 | 0.01 |
| 16 | rs1111875 | T | -0.09 | 0.01 | -0.02 | 0.01 |
| 17 | rs11257655 | T | 0.07 | 0.01 | 0.00 | 0.01 |
| 18 | rs1127655 | T | -0.04 | 0.01 | -0.01 | 0.01 |
| 19 | rs11708067 | G | -0.10 | 0.01 | -0.01 | 0.01 |
| 20 | rs11925227 | A | -0.05 | 0.01 | 0.01 | 0.01 |
| 21 | rs11926707 | C | 0.05 | 0.01 | 0.01 | 0.01 |
| 22 | rs12088739 | G | -0.09 | 0.01 | 0.02 | 0.02 |
| 23 | rs12299509 | G | 0.05 | 0.01 | 0.01 | 0.01 |
| 24 | rs12617659 | T | -0.07 | 0.01 | 0.01 | 0.01 |
| 25 | rs12910825 | G | 0.05 | 0.01 | -0.01 | 0.01 |
| 26 | rs12945601 | C | -0.05 | 0.01 | 0.01 | 0.01 |
| 27 | rs12970134 | A | 0.06 | 0.01 | 0.02 | 0.01 |
| 28 | rs13234269 | A | -0.06 | 0.01 | -0.01 | 0.01 |
| 29 | rs13239186 | T | 0.05 | 0.01 | -0.01 | 0.01 |
| 30 | rs13330951 | G | -0.05 | 0.01 | 0.00 | 0.01 |
| 31 | rs13389219 | T | -0.07 | 0.01 | -0.01 | 0.01 |
| 32 | rs1359790 | A | -0.08 | 0.01 | 0.02 | 0.01 |
| 33 | rs1496653 | G | -0.08 | 0.01 | 0.01 | 0.01 |
| 34 | rs1552224 | C | -0.10 | 0.01 | -0.01 | 0.01 |
| 35 | rs16988333 | G | -0.07 | 0.01 | 0.01 | 0.02 |
| 36 | rs17086692 | T | -0.05 | 0.01 | 0.01 | 0.01 |
| 37 | rs17168486 | T | 0.07 | 0.01 | 0.01 | 0.01 |
| 38 | rs17405722 | A | 0.09 | 0.01 | 0.01 | 0.02 |
| 39 | rs17411031 | G | -0.05 | 0.01 | -0.02 | 0.01 |
| 40 | rs1758632 | G | 0.05 | 0.01 | -0.01 | 0.01 |
| 41 | rs17631783 | T | -0.05 | 0.01 | -0.01 | 0.01 |
| 42 | rs17791513 | G | -0.10 | 0.01 | 0.00 | 0.02 |
| 43 | rs1801214 | T | 0.09 | 0.01 | 0.02 | 0.01 |
| 44 | rs1899951 | T | -0.11 | 0.01 | 0.00 | 0.01 |
| 45 | rs2058913 | T | -0.05 | 0.01 | 0.00 | 0.01 |
| 46 | rs2237892 | T | -0.10 | 0.02 | -0.01 | 0.02 |
| 47 | rs2246618 | T | 0.05 | 0.01 | 0.01 | 0.01 |
| 48 | rs2261181 | T | 0.10 | 0.01 | 0.00 | 0.02 |
| 49 | rs2294120 | G | -0.04 | 0.01 | 0.00 | 0.01 |
| 50 | rs2296173 | G | 0.07 | 0.01 | 0.00 | 0.01 |
| 51 | rs2299383 | T | 0.04 | 0.01 | 0.01 | 0.01 |
| 52 | rs243019 | C | 0.06 | 0.01 | -0.01 | 0.01 |
| 53 | rs2493394 | G | 0.07 | 0.01 | 0.02 | 0.02 |
| 54 | rs2796441 | A | -0.07 | 0.01 | 0.01 | 0.01 |
| 55 | rs2820426 | G | 0.05 | 0.01 | 0.02 | 0.01 |
| 56 | rs2867125 | C | 0.06 | 0.01 | 0.01 | 0.01 |
| 57 | rs2908282 | A | 0.06 | 0.01 | -0.02 | 0.02 |
| 58 | rs2925979 | C | -0.05 | 0.01 | 0.00 | 0.01 |
| 59 | rs2943656 | G | 0.09 | 0.01 | 0.01 | 0.01 |
| 60 | rs3217992 | T | 0.05 | 0.01 | 0.00 | 0.01 |
| 61 | rs340874 | C | 0.06 | 0.01 | 0.01 | 0.01 |
| 62 | rs348330 | A | -0.05 | 0.01 | 0.00 | 0.01 |
| 63 | rs3756784 | G | 0.05 | 0.01 | -0.01 | 0.01 |
| 64 | rs3802177 | A | -0.12 | 0.01 | -0.01 | 0.01 |
| 65 | rs459193 | G | 0.07 | 0.01 | -0.01 | 0.01 |
| 66 | rs4622883 | G | -0.04 | 0.01 | 0.00 | 0.01 |
| 67 | rs4686471 | C | 0.05 | 0.01 | -0.01 | 0.01 |
| 68 | rs4812829 | A | 0.05 | 0.01 | 0.00 | 0.01 |
| 69 | rs4823182 | G | 0.05 | 0.01 | 0.01 | 0.01 |
| 70 | rs4865796 | A | 0.05 | 0.01 | 0.01 | 0.01 |
| 71 | rs516946 | C | 0.08 | 0.01 | 0.00 | 0.01 |
| 72 | rs5215 | T | -0.07 | 0.01 | -0.01 | 0.01 |
| 73 | rs55966194 | G | -0.05 | 0.01 | -0.01 | 0.01 |
| 74 | rs576674 | A | -0.07 | 0.01 | 0.01 | 0.02 |
| 75 | rs6059662 | G | 0.04 | 0.01 | 0.00 | 0.01 |
| 76 | rs61953351 | T | -0.07 | 0.01 | -0.03 | 0.01 |
| 77 | rs622217 | C | -0.05 | 0.01 | -0.02 | 0.01 |
| 78 | rs6494307 | G | -0.04 | 0.01 | 0.01 | 0.01 |
| 79 | rs6515236 | C | -0.05 | 0.01 | 0.00 | 0.01 |
| 80 | rs67232546 | T | 0.06 | 0.01 | 0.02 | 0.01 |
| 81 | rs6767484 | G | 0.12 | 0.01 | 0.00 | 0.01 |
| 82 | rs6785040 | C | -0.06 | 0.01 | 0.00 | 0.01 |
| 83 | rs6795735 | T | -0.06 | 0.01 | 0.00 | 0.01 |
| 84 | rs6878122 | A | -0.06 | 0.01 | -0.02 | 0.01 |
| 85 | rs6960043 | C | 0.06 | 0.01 | 0.00 | 0.01 |
| 86 | rs7144011 | T | 0.05 | 0.01 | 0.00 | 0.01 |
| 87 | rs7177055 | A | 0.06 | 0.01 | 0.00 | 0.01 |
| 88 | rs7240767 | C | 0.05 | 0.01 | 0.02 | 0.01 |
| 89 | rs72802358 | C | -0.12 | 0.01 | 0.01 | 0.02 |
| 90 | rs72892910 | T | 0.06 | 0.01 | 0.01 | 0.01 |
| 91 | rs735949 | C | -0.07 | 0.01 | 0.00 | 0.02 |
| 92 | rs753270 | C | 0.05 | 0.01 | 0.02 | 0.01 |
| 93 | rs7561798 | G | 0.04 | 0.01 | 0.00 | 0.01 |
| 94 | rs7572970 | G | 0.06 | 0.01 | -0.01 | 0.01 |
| 95 | rs7607777 | T | -0.14 | 0.01 | -0.05 | 0.02 |
| 96 | rs7674212 | T | -0.05 | 0.01 | -0.01 | 0.01 |
| 97 | rs7685296 | T | -0.05 | 0.01 | 0.00 | 0.01 |
| 98 | rs7729395 | T | 0.14 | 0.02 | -0.02 | 0.02 |
| 99 | rs7756992 | G | 0.13 | 0.01 | -0.01 | 0.01 |
| 100 | rs7786095 | G | -0.07 | 0.01 | -0.02 | 0.02 |
| 101 | rs780094 | C | 0.07 | 0.01 | -0.04 | 0.01 |
| 102 | rs7845219 | C | -0.04 | 0.01 | 0.00 | 0.01 |
| 103 | rs7903146 | T | 0.31 | 0.01 | 0.01 | 0.01 |
| 104 | rs7929543 | C | 0.08 | 0.01 | -0.01 | 0.02 |
| 105 | rs7955901 | T | -0.04 | 0.01 | 0.01 | 0.01 |
| 106 | rs8068804 | A | 0.06 | 0.01 | -0.01 | 0.01 |
| 107 | rs8108269 | G | 0.06 | 0.01 | 0.02 | 0.01 |
| 108 | rs825476 | T | 0.05 | 0.01 | 0.02 | 0.01 |
| 109 | rs840967 | A | -0.05 | 0.01 | 0.00 | 0.01 |
| 110 | rs849135 | A | -0.10 | 0.01 | 0.01 | 0.01 |
| 111 | rs853974 | C | -0.06 | 0.01 | 0.02 | 0.01 |
| 112 | rs9369425 | A | -0.05 | 0.01 | 0.01 | 0.01 |
| 113 | rs963740 | T | -0.05 | 0.01 | -0.01 | 0.01 |
| 114 | rs9844972 | C | 0.10 | 0.01 | 0.00 | 0.02 |
| 115 | rs9894220 | G | -0.06 | 0.01 | 0.00 | 0.01 |
| 116 | rs9928094 | G | 0.10 | 0.01 | 0.02 | 0.01 |
| 117 | rs993380 | G | -0.05 | 0.01 | 0.00 | 0.01 |
| 118 | rs9940149 | A | -0.06 | 0.01 | 0.02 | 0.02 |

T2DM: Type-2 diabetes; LDL: Low-density lipoprotein.

Table 24: MR-PRESSO for estimate of T2DM-HDL(Prins).

|  | Causal Estimate | Sd | P-Value |
| --- | --- | --- | --- |
| beta.exposure Raw | -0.14 | 0.03 | <0.001 |
| beta.exposure Outlier-corrected | NA | NA | NA |

No outlier was detected.

T2DM: Type-2 diabetes; HDL: High-density lipoprotein; Sd: Standard deviation.

Table 25: MR-PRESSO for estimate of T2DM-HDL(Willer).

|  | Causal Estimate | Sd | P-Value |
| --- | --- | --- | --- |
| beta.exposure Raw | -0.07 | 0.02 | <0.001 |
| beta.exposure Outlier-corrected | -0.05 | 0.01 | <0.001 |

P-value for distortion test: 0.006.

T2DM: Type-2 diabetes; HDL: High-density lipoprotein; Sd: Standard deviation.

Table 26: MR-PRESSO for estimate of T2DM-HDL(Kettunen).

|  | Causal Estimate | Sd | P-Value |
| --- | --- | --- | --- |
| beta.exposure Raw | -0.08 | 0.02 | <0.001 |
| beta.exposure Outlier-corrected | -0.07 | 0.02 | <0.001 |

P-value for distortion test: 0.680.

T2DM: Type-2 diabetes; HDL: High-density lipoprotein; Sd: Standard deviation.

Table 27: MR-PRESSO for estimate of T2DM-LDL(Willer).

|  | Causal Estimate | Sd | P-Value |
| --- | --- | --- | --- |
| beta.exposure Raw | 0.01 | 0.01 | 0.422 |
| beta.exposure Outlier-corrected | 0.02 | 0.01 | 0.002 |

P-value for distortion test: 0.362.

T2DM: Type-2 diabetes; LDL: Low-density lipoprotein; Sd: Standard deviation.

Table 28: MR-PRESSO for estimate of T2DM-LDL(Prins).

|  | Causal Estimate | Sd | P-Value |
| --- | --- | --- | --- |
| beta.exposure Raw | -0.06 | 0.03 | 0.013 |
| beta.exposure Outlier-corrected | -0.06 | 0.02 | 0.018 |

P-value for distortion test: 0.811.

T2DM: Type-2 diabetes; LDL: Low-density lipoprotein; Sd: Standard deviation.

Table 29: MR-PRESSO for estimate of T2DM-LDL(Kanai).

|  | Causal Estimate | Sd | P-Value |
| --- | --- | --- | --- |
| beta.exposure Raw | 0.00 | 0.01 | 0.978 |
| beta.exposure Outlier-corrected | 0.00 | 0.01 | 0.743 |

P-value for distortion test: 0.052.

T2DM: Type-2 diabetes; LDL: Low-density lipoprotein; Sd: Standard deviation.

Table 30: MR-PRESSO for estimate of T2DM-LDL(Kettunen).

|  | Causal Estimate | Sd | P-Value |
| --- | --- | --- | --- |
| beta.exposure Raw | 0.03 | 0.02 | 0.127 |
| beta.exposure Outlier-corrected | 0.04 | 0.01 | 0.018 |

P-value for distortion test: 0.598.

T2DM: Type-2 diabetes; LDL: Low-density lipoprotein; Sd: Standard deviation.

Table 31: MR-PRESSO for estimate of T2DM-TC(Willer).

|  | Causal Estimate | Sd | P-Value |
| --- | --- | --- | --- |
| beta.exposure Raw | 0.00 | 0.02 | 0.969 |
| beta.exposure Outlier-corrected | 0.01 | 0.01 | 0.066 |

P-value for distortion test: 0.573.

T2DM: Type-2 diabetes; TC: Total cholesterol; Sd: Standard deviation.

Table 32: MR-PRESSO for estimate of T2DM-TC(Kettunen).

|  | Causal Estimate | Sd | P-Value |
| --- | --- | --- | --- |
| beta.exposure Raw | 0.01 | 0.02 | 0.782 |
| beta.exposure Outlier-corrected | 0.02 | 0.01 | 0.162 |

P-value for distortion test: 0.582.

T2DM: Type-2 diabetes; TC: Total cholesterol; Sd: Standard deviation.

Table 33: MR-PRESSO for estimate of T2DM-TC(Prins).

|  | Causal Estimate | Sd | P-Value |
| --- | --- | --- | --- |
| beta.exposure Raw | -0.06 | 0.03 | 0.020 |
| beta.exposure Outlier-corrected | -0.06 | 0.03 | 0.026 |

P-value for distortion test: 0.774.

T2DM: Type-2 diabetes; TC: Total cholesterol; Sd: Standard deviation.

Table 34: MR-PRESSO for estimate of T2DM-TC(Kanai).

|  | Causal Estimate | Sd | P-Value |
| --- | --- | --- | --- |
| beta.exposure Raw | 0.00 | 0.01 | 0.626 |
| beta.exposure Outlier-corrected | 0.01 | 0.01 | 0.500 |

P-value for distortion test: 0.238.

T2DM: Type-2 diabetes; TC: Total cholesterol; Sd: Standard deviation.

Table 35: MR-PRESSO for estimate of T2DM-TG(Willer).

|  | Causal Estimate | Sd | P-Value |
| --- | --- | --- | --- |
| beta.exposure Raw | 0.05 | 0.03 | <0.001 |
| beta.exposure Outlier-corrected | 0.05 | 0.01 | <0.001 |

P-value for distortion test: 0.293.

T2DM: Type-2 diabetes; TG: Triglycerides; Sd: Standard deviation.

Table 36: MR-PRESSO for estimate of T2DM-TG(Prins).

|  | Causal Estimate | Sd | P-Value |
| --- | --- | --- | --- |
| beta.exposure Raw | 0.14 | 0.03 | <0.001 |
| beta.exposure Outlier-corrected | 0.15 | 0.03 | <0.001 |

P-value for distortion test: 0.716.

T2DM: Type-2 diabetes; TG: Triglycerides; Sd: Standard deviation.

Table 37: MR-PRESSO for estimate of T2DM-TG(Kettunen).

|  | Causal Estimate | Sd | P-Value |
| --- | --- | --- | --- |
| beta.exposure Raw | 0.07 | 0.03 | <0.001 |
| beta.exposure Outlier-corrected | 0.09 | 0.02 | <0.001 |

P-value for distortion test: 0.311.

T2DM: Type-2 diabetes; TG: Triglycerides; Sd: Standard deviation.

Table 38: MR-PRESSO for estimate of T2DM-TG(Kanai).

|  | Causal Estimate | Sd | P-Value |
| --- | --- | --- | --- |
| beta.exposure Raw | -0.02 | 0.03 | 0.441 |
| beta.exposure Outlier-corrected | -0.02 | 0.01 | 0.040 |

P-value for distortion test: 0.969.

T2DM: Type-2 diabetes; TG: Triglycerides; Sd: Standard deviation.

Table 39: Single SNP analysis for estimate of T2DM-HDL(Prins).

| SNP | Effect | Se | P-value |
| --- | --- | --- | --- |
| rs10077431 | -0.21 | 0.41 | 0.617 |
| rs10100265 | 0.20 | 0.41 | 0.617 |
| rs10114341 | -0.49 | 0.24 | 0.046 |
| rs10401969 | 0.43 | 0.33 | 0.182 |
| rs1050226 | -0.81 | 0.41 | 0.046 |
| rs1061813 | -0.47 | 0.23 | 0.046 |
| rs1063355 | 0.14 | 0.28 | 0.617 |
| rs10740322 | 0.21 | 0.42 | 0.617 |
| rs10811661 | -0.13 | 0.13 | 0.317 |
| rs10974438 | -0.17 | 0.34 | 0.617 |
| rs11098676 | -0.56 | 0.37 | 0.134 |
| rs11107116 | -0.43 | 0.43 | 0.317 |
| rs11257655 | 0.14 | 0.27 | 0.617 |
| rs1127655 | -0.46 | 0.46 | 0.317 |
| rs11708067 | 0.10 | 0.21 | 0.617 |
| rs11925227 | -0.19 | 0.37 | 0.617 |
| rs12088739 | -0.90 | 0.34 | 0.008 |
| rs12299509 | 0.64 | 0.43 | 0.134 |
| rs12617659 | -0.15 | 0.29 | 0.617 |
| rs12910825 | 0.19 | 0.39 | 0.617 |
| rs12945601 | -0.42 | 0.42 | 0.317 |
| rs12970134 | -0.36 | 0.36 | 0.317 |
| rs13234269 | -0.51 | 0.17 | 0.003 |
| rs13330951 | -0.22 | 0.44 | 0.617 |
| rs13389219 | -0.28 | 0.28 | 0.317 |
| rs1496653 | -0.26 | 0.26 | 0.317 |
| rs1552224 | -0.10 | 0.19 | 0.617 |
| rs16988333 | -0.13 | 0.40 | 0.739 |
| rs17086692 | -2.36 | 1.28 | 0.067 |
| rs17168486 | -0.40 | 0.27 | 0.134 |
| rs17405722 | -0.34 | 0.34 | 0.317 |
| rs17411031 | -2.22 | 0.44 | 0.000 |
| rs1758632 | -0.81 | 0.41 | 0.046 |
| rs17791513 | -0.10 | 0.29 | 0.739 |
| rs1801214 | -0.11 | 0.22 | 0.617 |
| rs1899951 | -0.27 | 0.18 | 0.134 |
| rs2058913 | -0.20 | 0.41 | 0.617 |
| rs2237892 | -0.52 | 0.31 | 0.096 |
| rs2246618 | -0.19 | 0.39 | 0.617 |
| rs2261181 | 0.10 | 0.20 | 0.617 |
| rs2294120 | 0.23 | 0.45 | 0.617 |
| rs2296173 | -0.46 | 0.31 | 0.134 |
| rs2299383 | -0.49 | 0.24 | 0.046 |
| rs243019 | 0.18 | 0.35 | 0.617 |
| rs2493394 | -0.41 | 0.27 | 0.134 |
| rs2820426 | -0.58 | 0.38 | 0.134 |
| rs2867125 | -0.33 | 0.33 | 0.317 |
| rs2908282 | 0.72 | 0.36 | 0.046 |
| rs2925979 | -0.75 | 0.37 | 0.046 |
| rs2943656 | -0.33 | 0.22 | 0.134 |
| rs3217992 | -0.19 | 0.38 | 0.617 |
| rs3756784 | -0.59 | 0.40 | 0.134 |
| rs3802177 | -0.08 | 0.16 | 0.617 |
| rs459193 | -0.28 | 0.28 | 0.317 |
| rs4622883 | -0.46 | 0.23 | 0.046 |
| rs4686471 | -0.19 | 0.37 | 0.617 |
| rs4812829 | 0.19 | 0.38 | 0.617 |
| rs4823182 | -0.62 | 0.41 | 0.134 |
| rs4865796 | -0.19 | 0.38 | 0.617 |
| rs516946 | -0.24 | 0.24 | 0.317 |
| rs5215 | 0.15 | 0.29 | 0.617 |
| rs55966194 | 0.19 | 0.38 | 0.617 |
| rs576674 | 0.31 | 0.31 | 0.317 |
| rs6059662 | 0.22 | 0.45 | 0.617 |
| rs61953351 | 0.14 | 0.29 | 0.617 |
| rs622217 | -0.21 | 0.41 | 0.617 |
| rs6494307 | 0.23 | 0.45 | 0.617 |
| rs6515236 | 0.20 | 0.40 | 0.617 |
| rs67232546 | 0.34 | 0.34 | 0.317 |
| rs6767484 | -0.33 | 0.17 | 0.046 |
| rs6785040 | -0.16 | 0.32 | 0.617 |
| rs6878122 | 0.18 | 0.35 | 0.617 |
| rs6960043 | -0.16 | 0.31 | 0.617 |
| rs7240767 | -0.22 | 0.22 | 0.317 |
| rs72802358 | -0.09 | 0.17 | 0.617 |
| rs72892910 | 0.31 | 0.31 | 0.317 |
| rs735949 | 0.14 | 0.28 | 0.617 |
| rs753270 | -0.19 | 0.38 | 0.617 |
| rs7561798 | 0.50 | 0.25 | 0.046 |
| rs7572970 | -0.17 | 0.34 | 0.617 |
| rs7607777 | 0.22 | 0.15 | 0.134 |
| rs7674212 | -0.22 | 0.43 | 0.617 |
| rs7685296 | -0.59 | 0.39 | 0.134 |
| rs7729395 | -0.44 | 0.22 | 0.046 |
| rs7756992 | -0.08 | 0.15 | 0.617 |
| rs7786095 | -0.13 | 0.27 | 0.617 |
| rs780094 | 0.14 | 0.29 | 0.617 |
| rs7845219 | -0.24 | 0.47 | 0.617 |
| rs7903146 | -0.03 | 0.07 | 0.617 |
| rs7929543 | 0.12 | 0.36 | 0.739 |
| rs7955901 | -0.68 | 0.45 | 0.134 |
| rs8068804 | -0.17 | 0.34 | 0.617 |
| rs8108269 | -0.31 | 0.31 | 0.317 |
| rs825476 | -0.19 | 0.19 | 0.317 |
| rs853974 | -0.17 | 0.33 | 0.617 |
| rs9369425 | -0.37 | 0.37 | 0.317 |
| rs9844972 | -0.31 | 0.31 | 0.317 |
| rs9894220 | -0.17 | 0.34 | 0.617 |
| rs9928094 | -0.19 | 0.19 | 0.317 |
| rs993380 | -0.20 | 0.39 | 0.617 |
| rs9940149 | -0.34 | 0.34 | 0.317 |
| All - Inverse variance weighted | -0.14 | 0.03 | 0.000 |
| All - MR Egger | -0.01 | 0.06 | 0.928 |

T2DM: Type-2 diabetes; HDL: High-density lipoprotein; Se: Standard error.

Table 40: Leave-one-out analysis for estimate of T2DM-HDL(Prins).

| SNP | Effect | Se | P-value |
| --- | --- | --- | --- |
| rs10077431 | -0.14 | 0.03 | 7.61E-07 |
| rs10100265 | -0.14 | 0.03 | 5.28E-07 |
| rs10114341 | -0.14 | 0.03 | 1.29E-06 |
| rs10401969 | -0.15 | 0.03 | 2.76E-07 |
| rs1050226 | -0.14 | 0.03 | 8.93E-07 |
| rs1061813 | -0.14 | 0.03 | 1.35E-06 |
| rs1063355 | -0.15 | 0.03 | 4.52E-07 |
| rs10740322 | -0.14 | 0.03 | 5.32E-07 |
| rs10811661 | -0.14 | 0.03 | 1.04E-06 |
| rs10974438 | -0.14 | 0.03 | 7.68E-07 |
| rs11098676 | -0.14 | 0.03 | 9.29E-07 |
| rs11107116 | -0.14 | 0.03 | 8.35E-07 |
| rs11257655 | -0.15 | 0.03 | 4.42E-07 |
| rs1127655 | -0.14 | 0.03 | 8.23E-07 |
| rs11708067 | -0.15 | 0.03 | 3.63E-07 |
| rs11925227 | -0.14 | 0.03 | 7.65E-07 |
| rs12088739 | -0.14 | 0.03 | 9.75E-07 |
| rs12299509 | -0.15 | 0.03 | 3.01E-07 |
| rs12617659 | -0.14 | 0.03 | 7.70E-07 |
| rs12910825 | -0.15 | 0.03 | 5.19E-07 |
| rs12945601 | -0.14 | 0.03 | 8.41E-07 |
| rs12970134 | -0.14 | 0.03 | 8.71E-07 |
| rs13234269 | -0.13 | 0.03 | 2.55E-06 |
| rs13330951 | -0.14 | 0.03 | 7.58E-07 |
| rs13389219 | -0.14 | 0.03 | 9.31E-07 |
| rs1496653 | -0.14 | 0.03 | 9.46E-07 |
| rs1552224 | -0.14 | 0.03 | 7.47E-07 |
| rs16988333 | -0.14 | 0.03 | 7.27E-07 |
| rs17086692 | -0.14 | 0.03 | 6.01E-07 |
| rs17168486 | -0.14 | 0.03 | 1.08E-06 |
| rs17405722 | -0.14 | 0.03 | 8.80E-07 |
| rs17411031 | -0.14 | 0.03 | 2.08E-07 |
| rs1758632 | -0.14 | 0.03 | 8.93E-07 |
| rs17791513 | -0.14 | 0.03 | 7.18E-07 |
| rs1801214 | -0.14 | 0.03 | 7.61E-07 |
| rs1899951 | -0.14 | 0.03 | 1.37E-06 |
| rs2058913 | -0.14 | 0.03 | 7.61E-07 |
| rs2237892 | -0.14 | 0.03 | 1.03E-06 |
| rs2246618 | -0.14 | 0.03 | 7.63E-07 |
| rs2261181 | -0.15 | 0.03 | 3.56E-07 |
| rs2294120 | -0.14 | 0.03 | 5.44E-07 |
| rs2296173 | -0.14 | 0.03 | 1.01E-06 |
| rs2299383 | -0.14 | 0.03 | 1.30E-06 |
| rs243019 | -0.15 | 0.03 | 5.02E-07 |
| rs2493394 | -0.14 | 0.03 | 1.08E-06 |
| rs2820426 | -0.14 | 0.03 | 9.14E-07 |
| rs2867125 | -0.14 | 0.03 | 8.88E-07 |
| rs2908282 | -0.15 | 0.03 | 1.74E-07 |
| rs2925979 | -0.14 | 0.03 | 9.41E-07 |
| rs2943656 | -0.14 | 0.03 | 1.21E-06 |
| rs3217992 | -0.14 | 0.03 | 7.64E-07 |
| rs3756784 | -0.14 | 0.03 | 9.02E-07 |
| rs3802177 | -0.15 | 0.03 | 7.15E-07 |
| rs459193 | -0.14 | 0.03 | 9.27E-07 |
| rs4622883 | -0.14 | 0.03 | 1.36E-06 |
| rs4686471 | -0.14 | 0.03 | 7.65E-07 |
| rs4812829 | -0.15 | 0.03 | 5.14E-07 |
| rs4823182 | -0.14 | 0.03 | 8.85E-07 |
| rs4865796 | -0.14 | 0.03 | 7.64E-07 |
| rs516946 | -0.14 | 0.03 | 9.62E-07 |
| rs5215 | -0.15 | 0.03 | 4.63E-07 |
| rs55966194 | -0.15 | 0.03 | 5.16E-07 |
| rs576674 | -0.15 | 0.03 | 3.38E-07 |
| rs6059662 | -0.14 | 0.03 | 5.43E-07 |
| rs61953351 | -0.15 | 0.03 | 4.55E-07 |
| rs622217 | -0.14 | 0.03 | 7.61E-07 |
| rs6494307 | -0.14 | 0.03 | 5.44E-07 |
| rs6515236 | -0.14 | 0.03 | 5.23E-07 |
| rs67232546 | -0.15 | 0.03 | 3.61E-07 |
| rs6767484 | -0.14 | 0.03 | 1.86E-06 |
| rs6785040 | -0.14 | 0.03 | 7.69E-07 |
| rs6878122 | -0.15 | 0.03 | 5.03E-07 |
| rs6960043 | -0.14 | 0.03 | 7.69E-07 |
| rs7240767 | -0.14 | 0.03 | 9.84E-07 |
| rs72802358 | -0.14 | 0.03 | 7.25E-07 |
| rs72892910 | -0.15 | 0.03 | 3.40E-07 |
| rs735949 | -0.15 | 0.03 | 4.51E-07 |
| rs753270 | -0.14 | 0.03 | 7.64E-07 |
| rs7561798 | -0.15 | 0.03 | 1.04E-07 |
| rs7572970 | -0.14 | 0.03 | 7.68E-07 |
| rs7607777 | -0.16 | 0.03 | 5.86E-08 |
| rs7674212 | -0.14 | 0.03 | 7.59E-07 |
| rs7685296 | -0.14 | 0.03 | 9.07E-07 |
| rs7729395 | -0.14 | 0.03 | 1.43E-06 |
| rs7756992 | -0.15 | 0.03 | 6.98E-07 |
| rs7786095 | -0.14 | 0.03 | 7.69E-07 |
| rs780094 | -0.15 | 0.03 | 4.58E-07 |
| rs7845219 | -0.14 | 0.03 | 7.55E-07 |
| rs7903146 | -0.16 | 0.03 | 1.27E-07 |
| rs7929543 | -0.14 | 0.03 | 5.51E-07 |
| rs7955901 | -0.14 | 0.03 | 8.56E-07 |
| rs8068804 | -0.14 | 0.03 | 7.67E-07 |
| rs8108269 | -0.14 | 0.03 | 9.04E-07 |
| rs825476 | -0.14 | 0.03 | 1.02E-06 |
| rs853974 | -0.14 | 0.03 | 7.68E-07 |
| rs9369425 | -0.14 | 0.03 | 8.67E-07 |
| rs9844972 | -0.14 | 0.03 | 9.01E-07 |
| rs9894220 | -0.14 | 0.03 | 7.67E-07 |
| rs9928094 | -0.14 | 0.03 | 1.02E-06 |
| rs993380 | -0.14 | 0.03 | 7.63E-07 |
| rs9940149 | -0.14 | 0.03 | 8.80E-07 |
| All | -0.14 | 0.03 | 6.10E-07 |

T2DM: Type-2 diabetes; HDL: High-density lipoprotein; Se: Standard error.

Table 41: Single SNP analysis for estimate of T2DM-HDL(Willer).

| SNP | Effect | Se | P-value |
| --- | --- | --- | --- |
| rs10077431 | -0.22 | 0.09 | 1.370E-02 |
| rs10100265 | 0.03 | 0.07 | 6.272E-01 |
| rs10114341 | -0.22 | 0.08 | 7.440E-03 |
| rs10401969 | 0.14 | 0.07 | 5.979E-02 |
| rs1050226 | 0.00 | 0.07 | 1.000E+00 |
| rs1061813 | 0.17 | 0.08 | 3.700E-02 |
| rs1063355 | 0.16 | 0.08 | 4.935E-02 |
| rs10740322 | 0.01 | 0.11 | 9.398E-01 |
| rs10811661 | -0.02 | 0.03 | 5.634E-01 |
| rs10830963 | 0.04 | 0.04 | 3.975E-01 |
| rs10842994 | -0.08 | 0.06 | 1.700E-01 |
| rs10974438 | 0.08 | 0.06 | 1.649E-01 |
| rs11098676 | -0.10 | 0.11 | 3.430E-01 |
| rs11107116 | -0.25 | 0.09 | 4.322E-03 |
| rs1111875 | -0.06 | 0.04 | 7.279E-02 |
| rs11257655 | 0.01 | 0.06 | 7.885E-01 |
| rs1127655 | -0.05 | 0.11 | 6.171E-01 |
| rs11708067 | 0.16 | 0.04 | 3.241E-04 |
| rs11925227 | -0.04 | 0.09 | 6.637E-01 |
| rs11926707 | -0.23 | 0.11 | 3.781E-02 |
| rs12088739 | 0.04 | 0.07 | 5.375E-01 |
| rs12299509 | 0.10 | 0.08 | 1.735E-01 |
| rs12617659 | -0.23 | 0.07 | 1.242E-03 |
| rs12910825 | -0.07 | 0.07 | 2.904E-01 |
| rs12945601 | -0.26 | 0.10 | 1.013E-02 |
| rs12970134 | -0.37 | 0.07 | 6.504E-07 |
| rs13234269 | -0.70 | 0.08 | 5.893E-17 |
| rs13239186 | -0.02 | 0.09 | 8.572E-01 |
| rs13330951 | -0.21 | 0.11 | 5.019E-02 |
| rs13389219 | -0.31 | 0.05 | 1.067E-10 |
| rs1359790 | 0.00 | 0.05 | 9.580E-01 |
| rs1496653 | 0.06 | 0.05 | 2.301E-01 |
| rs1552224 | 0.02 | 0.04 | 7.056E-01 |
| rs16988333 | -0.10 | 0.08 | 2.128E-01 |
| rs17086692 | -0.08 | 0.11 | 4.851E-01 |
| rs17168486 | 0.09 | 0.06 | 1.198E-01 |
| rs17405722 | -0.12 | 0.10 | 2.577E-01 |
| rs17411031 | -2.33 | 0.08 | 4.115E-167 |
| rs1758632 | -0.11 | 0.10 | 2.531E-01 |
| rs17631783 | 0.05 | 0.11 | 6.567E-01 |
| rs17791513 | 0.06 | 0.06 | 3.173E-01 |
| rs1801214 | -0.03 | 0.04 | 4.073E-01 |
| rs1899951 | -0.11 | 0.05 | 1.863E-02 |
| rs2058913 | -0.17 | 0.10 | 9.423E-02 |
| rs2237892 | 0.08 | 0.08 | 2.865E-01 |
| rs2246618 | -0.29 | 0.08 | 1.953E-04 |
| rs2261181 | -0.07 | 0.06 | 2.491E-01 |
| rs2294120 | 0.12 | 0.10 | 2.492E-01 |
| rs2296173 | -0.57 | 0.06 | 1.920E-18 |
| rs2299383 | -0.10 | 0.08 | 2.414E-01 |
| rs243019 | -0.02 | 0.06 | 7.533E-01 |
| rs2493394 | 0.03 | 0.07 | 6.702E-01 |
| rs2796441 | -0.01 | 0.05 | 9.115E-01 |
| rs2820426 | -0.26 | 0.07 | 1.020E-04 |
| rs2867125 | -0.21 | 0.07 | 5.110E-03 |
| rs2908282 | 0.03 | 0.08 | 7.609E-01 |
| rs2925979 | -0.66 | 0.07 | 2.390E-21 |
| rs2943656 | -0.36 | 0.04 | 4.668E-20 |
| rs3217992 | -0.02 | 0.07 | 7.533E-01 |
| rs340874 | -0.19 | 0.05 | 4.165E-04 |
| rs3756784 | -0.29 | 0.09 | 9.826E-04 |
| rs3802177 | 0.00 | 0.03 | 9.139E-01 |
| rs459193 | -0.33 | 0.05 | 1.684E-09 |
| rs4622883 | -0.20 | 0.11 | 7.924E-02 |
| rs4812829 | 0.08 | 0.08 | 3.506E-01 |
| rs4823182 | -0.18 | 0.07 | 1.516E-02 |
| rs4865796 | -0.25 | 0.07 | 3.993E-04 |
| rs516946 | 0.14 | 0.05 | 4.372E-03 |
| rs5215 | 0.05 | 0.05 | 3.606E-01 |
| rs55966194 | -0.24 | 0.11 | 2.681E-02 |
| rs576674 | 0.08 | 0.07 | 2.886E-01 |
| rs6059662 | -0.33 | 0.08 | 5.649E-05 |
| rs622217 | -0.09 | 0.09 | 3.173E-01 |
| rs6494307 | -0.02 | 0.11 | 8.864E-01 |
| rs6515236 | -0.02 | 0.12 | 8.767E-01 |
| rs6767484 | -0.05 | 0.03 | 7.099E-02 |
| rs6785040 | 0.02 | 0.11 | 8.327E-01 |
| rs6795735 | -0.11 | 0.06 | 6.389E-02 |
| rs6878122 | -0.09 | 0.07 | 1.936E-01 |
| rs6960043 | 0.03 | 0.05 | 5.677E-01 |
| rs7144011 | -0.08 | 0.09 | 3.415E-01 |
| rs7177055 | -0.06 | 0.06 | 3.435E-01 |
| rs7240767 | -0.06 | 0.11 | 6.102E-01 |
| rs72802358 | -0.08 | 0.04 | 7.761E-02 |
| rs72892910 | 0.05 | 0.10 | 6.227E-01 |
| rs735949 | -0.03 | 0.07 | 6.832E-01 |
| rs753270 | -0.17 | 0.09 | 7.508E-02 |
| rs7561798 | -0.24 | 0.09 | 5.697E-03 |
| rs7572970 | -0.09 | 0.09 | 3.359E-01 |
| rs7607777 | -0.03 | 0.04 | 4.533E-01 |
| rs7685296 | -0.14 | 0.07 | 6.170E-02 |
| rs7729395 | -0.06 | 0.07 | 3.506E-01 |
| rs7756992 | 0.00 | 0.03 | 9.371E-01 |
| rs7786095 | 0.00 | 0.10 | 9.795E-01 |
| rs780094 | 0.16 | 0.05 | 1.673E-03 |
| rs7845219 | -0.05 | 0.08 | 5.564E-01 |
| rs7903146 | 0.01 | 0.01 | 2.806E-01 |
| rs7929543 | -0.08 | 0.08 | 3.612E-01 |
| rs7955901 | -0.20 | 0.08 | 1.050E-02 |
| rs8068804 | -0.22 | 0.06 | 6.238E-04 |
| rs8108269 | -0.27 | 0.06 | 7.217E-06 |
| rs825476 | -0.42 | 0.06 | 8.032E-11 |
| rs840967 | 0.04 | 0.10 | 7.134E-01 |
| rs849135 | -0.01 | 0.03 | 8.599E-01 |
| rs9369425 | 0.31 | 0.10 | 1.429E-03 |
| rs963740 | 0.05 | 0.11 | 6.781E-01 |
| rs9844972 | -0.26 | 0.10 | 7.661E-03 |
| rs9894220 | -0.17 | 0.09 | 4.550E-02 |
| rs9928094 | -0.18 | 0.03 | 7.812E-08 |
| rs993380 | -0.02 | 0.10 | 8.065E-01 |
| rs9940149 | 0.07 | 0.08 | 3.728E-01 |
| All - Inverse variance weighted | -0.07 | 0.02 | 6.389E-04 |
| All - MR Egger | 0.06 | 0.04 | 1.444E-01 |

T2DM: Type-2 diabetes; HDL: High-density lipoprotein; Se: Standard error.

Table 42: Leave-one-out analysis for estimate of T2DM-HDL(Willer).

| SNP | Effect | Se | P-value |
| --- | --- | --- | --- |
| rs10077431 | -0.06 | 0.02 | 7.58E-04 |
| rs10100265 | -0.07 | 0.02 | 6.24E-04 |
| rs10114341 | -0.06 | 0.02 | 7.71E-04 |
| rs10401969 | -0.07 | 0.02 | 5.51E-04 |
| rs1050226 | -0.07 | 0.02 | 6.51E-04 |
| rs1061813 | -0.07 | 0.02 | 5.52E-04 |
| rs1063355 | -0.07 | 0.02 | 5.59E-04 |
| rs10740322 | -0.07 | 0.02 | 6.63E-04 |
| rs10811661 | -0.07 | 0.02 | 5.98E-04 |
| rs10830963 | -0.07 | 0.02 | 5.37E-04 |
| rs10842994 | -0.07 | 0.02 | 7.29E-04 |
| rs10974438 | -0.07 | 0.02 | 5.54E-04 |
| rs11098676 | -0.07 | 0.02 | 6.98E-04 |
| rs11107116 | -0.06 | 0.02 | 7.72E-04 |
| rs1111875 | -0.07 | 0.02 | 7.72E-04 |
| rs11257655 | -0.07 | 0.02 | 6.17E-04 |
| rs1127655 | -0.07 | 0.02 | 6.83E-04 |
| rs11708067 | -0.07 | 0.02 | 3.44E-04 |
| rs11925227 | -0.07 | 0.02 | 6.78E-04 |
| rs11926707 | -0.07 | 0.02 | 7.29E-04 |
| rs12088739 | -0.07 | 0.02 | 6.13E-04 |
| rs12299509 | -0.07 | 0.02 | 5.84E-04 |
| rs12617659 | -0.06 | 0.02 | 8.16E-04 |
| rs12910825 | -0.07 | 0.02 | 7.08E-04 |
| rs12945601 | -0.06 | 0.02 | 7.49E-04 |
| rs12970134 | -0.06 | 0.02 | 8.74E-04 |
| rs13234269 | -0.06 | 0.02 | 8.77E-04 |
| rs13239186 | -0.07 | 0.02 | 6.69E-04 |
| rs13330951 | -0.07 | 0.02 | 7.30E-04 |
| rs13389219 | -0.06 | 0.02 | 1.14E-03 |
| rs1359790 | -0.07 | 0.02 | 6.25E-04 |
| rs1496653 | -0.07 | 0.02 | 5.44E-04 |
| rs1552224 | -0.07 | 0.02 | 5.78E-04 |
| rs16988333 | -0.07 | 0.02 | 7.14E-04 |
| rs17086692 | -0.07 | 0.02 | 6.90E-04 |
| rs17168486 | -0.07 | 0.02 | 5.43E-04 |
| rs17405722 | -0.07 | 0.02 | 7.05E-04 |
| rs17411031 | -0.06 | 0.01 | 3.29E-05 |
| rs1758632 | -0.07 | 0.02 | 7.06E-04 |
| rs17631783 | -0.07 | 0.02 | 6.49E-04 |
| rs17791513 | -0.07 | 0.02 | 5.85E-04 |
| rs1801214 | -0.07 | 0.02 | 6.72E-04 |
| rs1899951 | -0.06 | 0.02 | 8.17E-04 |
| rs2058913 | -0.07 | 0.02 | 7.24E-04 |
| rs2237892 | -0.07 | 0.02 | 6.02E-04 |
| rs2246618 | -0.06 | 0.02 | 8.19E-04 |
| rs2261181 | -0.07 | 0.02 | 7.14E-04 |
| rs2294120 | -0.07 | 0.02 | 6.18E-04 |
| rs2296173 | -0.06 | 0.02 | 1.03E-03 |
| rs2299383 | -0.07 | 0.02 | 7.10E-04 |
| rs243019 | -0.07 | 0.02 | 6.62E-04 |
| rs2493394 | -0.07 | 0.02 | 6.30E-04 |
| rs2796441 | -0.07 | 0.02 | 6.34E-04 |
| rs2820426 | -0.06 | 0.02 | 8.55E-04 |
| rs2867125 | -0.06 | 0.02 | 7.88E-04 |
| rs2908282 | -0.07 | 0.02 | 6.43E-04 |
| rs2925979 | -0.06 | 0.02 | 9.91E-04 |
| rs2943656 | -0.06 | 0.02 | 1.66E-03 |
| rs3217992 | -0.07 | 0.02 | 6.65E-04 |
| rs340874 | -0.06 | 0.02 | 8.81E-04 |
| rs3756784 | -0.06 | 0.02 | 7.87E-04 |
| rs3802177 | -0.07 | 0.02 | 5.33E-04 |
| rs459193 | -0.06 | 0.02 | 1.04E-03 |
| rs4622883 | -0.07 | 0.02 | 7.21E-04 |
| rs4812829 | -0.07 | 0.02 | 6.14E-04 |
| rs4823182 | -0.06 | 0.02 | 7.75E-04 |
| rs4865796 | -0.06 | 0.02 | 8.31E-04 |
| rs516946 | -0.07 | 0.02 | 4.18E-04 |
| rs5215 | -0.07 | 0.02 | 5.63E-04 |
| rs55966194 | -0.07 | 0.02 | 7.36E-04 |
| rs576674 | -0.07 | 0.02 | 5.97E-04 |
| rs6059662 | -0.06 | 0.02 | 8.17E-04 |
| rs622217 | -0.07 | 0.02 | 7.02E-04 |
| rs6494307 | -0.07 | 0.02 | 6.71E-04 |
| rs6515236 | -0.07 | 0.02 | 6.72E-04 |
| rs6767484 | -0.07 | 0.02 | 7.68E-04 |
| rs6785040 | -0.07 | 0.02 | 6.58E-04 |
| rs6795735 | -0.07 | 0.02 | 7.57E-04 |
| rs6878122 | -0.07 | 0.02 | 7.20E-04 |
| rs6960043 | -0.07 | 0.02 | 5.94E-04 |
| rs7144011 | -0.07 | 0.02 | 7.01E-04 |
| rs7177055 | -0.07 | 0.02 | 7.01E-04 |
| rs7240767 | -0.07 | 0.02 | 6.83E-04 |
| rs72802358 | -0.07 | 0.02 | 7.66E-04 |
| rs72892910 | -0.07 | 0.02 | 6.42E-04 |
| rs735949 | -0.07 | 0.02 | 6.72E-04 |
| rs753270 | -0.07 | 0.02 | 7.30E-04 |
| rs7561798 | -0.06 | 0.02 | 7.72E-04 |
| rs7572970 | -0.07 | 0.02 | 7.01E-04 |
| rs7607777 | -0.07 | 0.02 | 6.75E-04 |
| rs7685296 | -0.07 | 0.02 | 7.47E-04 |
| rs7729395 | -0.07 | 0.02 | 7.01E-04 |
| rs7756992 | -0.07 | 0.02 | 5.44E-04 |
| rs7786095 | -0.07 | 0.02 | 6.64E-04 |
| rs780094 | -0.07 | 0.02 | 4.09E-04 |
| rs7845219 | -0.07 | 0.02 | 6.84E-04 |
| rs7903146 | -0.08 | 0.02 | 7.17E-05 |
| rs7929543 | -0.07 | 0.02 | 6.99E-04 |
| rs7955901 | -0.06 | 0.02 | 7.75E-04 |
| rs8068804 | -0.06 | 0.02 | 8.39E-04 |
| rs8108269 | -0.06 | 0.02 | 9.11E-04 |
| rs825476 | -0.06 | 0.02 | 9.73E-04 |
| rs840967 | -0.07 | 0.02 | 6.48E-04 |
| rs849135 | -0.07 | 0.02 | 5.88E-04 |
| rs9369425 | -0.07 | 0.02 | 5.19E-04 |
| rs963740 | -0.07 | 0.02 | 6.51E-04 |
| rs9844972 | -0.06 | 0.02 | 7.56E-04 |
| rs9894220 | -0.07 | 0.02 | 7.42E-04 |
| rs9928094 | -0.06 | 0.02 | 1.29E-03 |
| rs993380 | -0.07 | 0.02 | 6.72E-04 |
| rs9940149 | -0.07 | 0.02 | 6.11E-04 |
| All | -0.07 | 0.02 | 6.39E-04 |

T2DM: Type-2 diabetes; HDL: High-density lipoprotein; Se: Standard error.

Table 43: Single SNP analysis for estimate of T2DM-HDL(Kettunen).

| SNP | Effect | Se | P-value |
| --- | --- | --- | --- |
| rs10077431 | 0.06 | 0.27 | 8.34E-01 |
| rs10087241 | 0.28 | 0.22 | 1.95E-01 |
| rs10100265 | -0.06 | 0.20 | 7.71E-01 |
| rs10114341 | -0.07 | 0.24 | 7.72E-01 |
| rs10401969 | 0.07 | 0.22 | 7.63E-01 |
| rs1050226 | -0.15 | 0.21 | 4.68E-01 |
| rs1061813 | -0.16 | 0.24 | 4.96E-01 |
| rs1063355 | -0.12 | 0.15 | 4.07E-01 |
| rs10740322 | 0.32 | 0.23 | 1.73E-01 |
| rs10811661 | -0.01 | 0.09 | 8.87E-01 |
| rs10830963 | 0.03 | 0.12 | 7.84E-01 |
| rs10842994 | -0.07 | 0.17 | 6.94E-01 |
| rs10974438 | 0.38 | 0.17 | 2.98E-02 |
| rs11098676 | 0.09 | 0.23 | 6.88E-01 |
| rs11107116 | -0.51 | 0.24 | 3.37E-02 |
| rs1111875 | -0.15 | 0.11 | 1.44E-01 |
| rs11257655 | 0.10 | 0.16 | 5.43E-01 |
| rs1127655 | -0.19 | 0.22 | 4.02E-01 |
| rs11708067 | 0.02 | 0.13 | 8.90E-01 |
| rs11925227 | 0.35 | 0.29 | 2.30E-01 |
| rs11926707 | -0.29 | 0.23 | 2.11E-01 |
| rs12088739 | 0.01 | 0.24 | 9.76E-01 |
| rs12299509 | 0.44 | 0.23 | 5.04E-02 |
| rs12617659 | -0.05 | 0.21 | 8.01E-01 |
| rs12910825 | -0.13 | 0.20 | 4.96E-01 |
| rs12945601 | -0.18 | 0.21 | 3.94E-01 |
| rs12970134 | -0.27 | 0.22 | 2.09E-01 |
| rs13234269 | -0.29 | 0.17 | 8.49E-02 |
| rs13239186 | 0.07 | 0.20 | 7.11E-01 |
| rs13330951 | -0.18 | 0.22 | 4.09E-01 |
| rs13389219 | -0.49 | 0.14 | 5.82E-04 |
| rs1359790 | -0.11 | 0.14 | 4.18E-01 |
| rs1496653 | -0.02 | 0.14 | 8.69E-01 |
| rs1552224 | 0.17 | 0.11 | 1.24E-01 |
| rs16988333 | -0.02 | 0.23 | 9.32E-01 |
| rs17086692 | 0.32 | 0.22 | 1.55E-01 |
| rs17168486 | 0.15 | 0.16 | 3.75E-01 |
| rs17405722 | -0.02 | 0.20 | 9.29E-01 |
| rs17411031 | -1.93 | 0.25 | 6.14E-15 |
| rs1758632 | -0.29 | 0.20 | 1.51E-01 |
| rs17631783 | 0.29 | 0.24 | 2.20E-01 |
| rs17791513 | 0.18 | 0.16 | 2.63E-01 |
| rs1801214 | -0.05 | 0.11 | 6.74E-01 |
| rs1899951 | -0.14 | 0.12 | 2.49E-01 |
| rs2058913 | 0.22 | 0.20 | 2.70E-01 |
| rs2237892 | -0.04 | 0.23 | 8.77E-01 |
| rs2246618 | -0.44 | 0.21 | 3.86E-02 |
| rs2261181 | 0.05 | 0.19 | 8.05E-01 |
| rs2294120 | -0.24 | 0.22 | 2.87E-01 |
| rs2296173 | -0.20 | 0.19 | 2.93E-01 |
| rs2299383 | -0.20 | 0.24 | 4.00E-01 |
| rs243019 | 0.08 | 0.17 | 6.60E-01 |
| rs2493394 | 0.26 | 0.20 | 2.04E-01 |
| rs2796441 | -0.18 | 0.14 | 2.07E-01 |
| rs2820426 | -0.14 | 0.20 | 4.77E-01 |
| rs2867125 | -0.38 | 0.22 | 8.78E-02 |
| rs2908282 | 0.04 | 0.27 | 8.77E-01 |
| rs2925979 | -0.15 | 0.20 | 4.56E-01 |
| rs2943656 | -0.42 | 0.11 | 1.99E-04 |
| rs3217992 | 0.41 | 0.19 | 3.24E-02 |
| rs340874 | -0.22 | 0.16 | 1.57E-01 |
| rs348330 | -0.03 | 0.22 | 8.86E-01 |
| rs3756784 | -0.31 | 0.24 | 2.03E-01 |
| rs3802177 | 0.04 | 0.08 | 6.61E-01 |
| rs459193 | -0.58 | 0.15 | 1.12E-04 |
| rs4622883 | -0.10 | 0.26 | 7.12E-01 |
| rs4686471 | -0.16 | 0.21 | 4.46E-01 |
| rs4812829 | 0.11 | 0.23 | 6.27E-01 |
| rs4823182 | -0.25 | 0.21 | 2.22E-01 |
| rs4865796 | -0.34 | 0.20 | 8.60E-02 |
| rs516946 | -0.01 | 0.14 | 9.69E-01 |
| rs5215 | 0.23 | 0.15 | 1.21E-01 |
| rs55966194 | 0.14 | 0.23 | 5.41E-01 |
| rs576674 | -0.15 | 0.24 | 5.41E-01 |
| rs6059662 | -0.47 | 0.26 | 7.22E-02 |
| rs61953351 | 0.17 | 0.16 | 2.93E-01 |
| rs622217 | -0.43 | 0.20 | 3.43E-02 |
| rs6494307 | -0.19 | 0.22 | 3.85E-01 |
| rs6515236 | -0.14 | 0.24 | 5.59E-01 |
| rs67232546 | 0.51 | 0.23 | 2.75E-02 |
| rs6767484 | -0.20 | 0.10 | 3.44E-02 |
| rs6785040 | 0.02 | 0.19 | 9.31E-01 |
| rs6795735 | -0.11 | 0.18 | 5.25E-01 |
| rs6878122 | 0.00 | 0.21 | 9.88E-01 |
| rs6960043 | -0.01 | 0.16 | 9.51E-01 |
| rs7144011 | -0.34 | 0.24 | 1.61E-01 |
| rs7177055 | -0.16 | 0.17 | 3.29E-01 |
| rs7240767 | -0.09 | 0.23 | 6.87E-01 |
| rs72802358 | -0.42 | 0.15 | 5.71E-03 |
| rs72892910 | 0.04 | 0.19 | 8.12E-01 |
| rs735949 | -0.21 | 0.22 | 3.42E-01 |
| rs753270 | -0.22 | 0.19 | 2.36E-01 |
| rs7561798 | -0.32 | 0.25 | 1.96E-01 |
| rs7572970 | 0.29 | 0.20 | 1.52E-01 |
| rs7607777 | 0.06 | 0.15 | 7.08E-01 |
| rs7674212 | -0.26 | 0.21 | 2.23E-01 |
| rs7685296 | -0.50 | 0.22 | 2.27E-02 |
| rs7729395 | 0.24 | 0.18 | 1.86E-01 |
| rs7756992 | -0.08 | 0.08 | 2.93E-01 |
| rs7786095 | -0.24 | 0.26 | 3.65E-01 |
| rs780094 | 0.03 | 0.15 | 8.58E-01 |
| rs7845219 | 0.34 | 0.23 | 1.37E-01 |
| rs7903146 | -0.11 | 0.04 | 6.05E-03 |
| rs7929543 | -0.25 | 0.23 | 2.93E-01 |
| rs7955901 | -0.09 | 0.23 | 7.05E-01 |
| rs8068804 | -0.11 | 0.18 | 5.57E-01 |
| rs8108269 | 0.12 | 0.17 | 4.90E-01 |
| rs825476 | -0.41 | 0.19 | 3.08E-02 |
| rs840967 | 0.12 | 0.20 | 5.33E-01 |
| rs849135 | -0.07 | 0.10 | 4.49E-01 |
| rs853974 | -0.17 | 0.19 | 3.53E-01 |
| rs9369425 | 0.39 | 0.20 | 5.76E-02 |
| rs963740 | 0.08 | 0.24 | 7.44E-01 |
| rs9844972 | 0.02 | 0.26 | 9.29E-01 |
| rs9894220 | -0.24 | 0.17 | 1.67E-01 |
| rs9928094 | -0.06 | 0.10 | 5.10E-01 |
| rs993380 | -0.09 | 0.20 | 6.66E-01 |
| rs9940149 | -0.60 | 0.27 | 2.56E-02 |
| All - Inverse variance weighted | -0.08 | 0.02 | 9.03E-05 |
| All - MR Egger | -0.06 | 0.05 | 2.46E-01 |

T2DM: Type-2 diabetes; HDL: High-density lipoprotein; Se: Standard error.

Table 44: Leave-one-out analysis for estimate of T2DM-HDL(Kettunen).

| SNP | Effect | Se | P-value |
| --- | --- | --- | --- |
| rs10077431 | -0.08 | 0.02 | 9.03E-05 |
| rs10087241 | -0.08 | 0.02 | 6.44E-05 |
| rs10100265 | -0.08 | 0.02 | 9.87E-05 |
| rs10114341 | -0.08 | 0.02 | 9.89E-05 |
| rs10401969 | -0.08 | 0.02 | 8.63E-05 |
| rs1050226 | -0.08 | 0.02 | 1.08E-04 |
| rs1061813 | -0.08 | 0.02 | 1.06E-04 |
| rs1063355 | -0.08 | 0.02 | 1.14E-04 |
| rs10740322 | -0.08 | 0.02 | 6.47E-05 |
| rs10811661 | -0.08 | 0.02 | 8.13E-05 |
| rs10830963 | -0.08 | 0.02 | 7.50E-05 |
| rs10842994 | -0.08 | 0.02 | 1.01E-04 |
| rs10974438 | -0.08 | 0.02 | 3.97E-05 |
| rs11098676 | -0.08 | 0.02 | 8.49E-05 |
| rs11107116 | -0.08 | 0.02 | 1.23E-04 |
| rs1111875 | -0.08 | 0.02 | 1.49E-04 |
| rs11257655 | -0.08 | 0.02 | 7.26E-05 |
| rs1127655 | -0.08 | 0.02 | 1.09E-04 |
| rs11708067 | -0.08 | 0.02 | 8.20E-05 |
| rs11925227 | -0.08 | 0.02 | 7.30E-05 |
| rs11926707 | -0.08 | 0.02 | 1.15E-04 |
| rs12088739 | -0.08 | 0.02 | 9.27E-05 |
| rs12299509 | -0.08 | 0.02 | 5.19E-05 |
| rs12617659 | -0.08 | 0.02 | 9.79E-05 |
| rs12910825 | -0.08 | 0.02 | 1.07E-04 |
| rs12945601 | -0.08 | 0.02 | 1.10E-04 |
| rs12970134 | -0.08 | 0.02 | 1.17E-04 |
| rs13234269 | -0.08 | 0.02 | 1.34E-04 |
| rs13239186 | -0.08 | 0.02 | 8.32E-05 |
| rs13330951 | -0.08 | 0.02 | 1.09E-04 |
| rs13389219 | -0.08 | 0.02 | 1.89E-04 |
| rs1359790 | -0.08 | 0.02 | 1.14E-04 |
| rs1496653 | -0.08 | 0.02 | 9.27E-05 |
| rs1552224 | -0.08 | 0.02 | 3.68E-05 |
| rs16988333 | -0.08 | 0.02 | 9.49E-05 |
| rs17086692 | -0.08 | 0.02 | 6.24E-05 |
| rs17168486 | -0.08 | 0.02 | 6.65E-05 |
| rs17405722 | -0.08 | 0.02 | 9.39E-05 |
| rs17411031 | -0.07 | 0.02 | 3.77E-05 |
| rs1758632 | -0.08 | 0.02 | 1.21E-04 |
| rs17631783 | -0.08 | 0.02 | 6.84E-05 |
| rs17791513 | -0.08 | 0.02 | 5.93E-05 |
| rs1801214 | -0.08 | 0.02 | 9.85E-05 |
| rs1899951 | -0.08 | 0.02 | 1.30E-04 |
| rs2058913 | -0.08 | 0.02 | 6.69E-05 |
| rs2237892 | -0.08 | 0.02 | 9.62E-05 |
| rs2246618 | -0.08 | 0.02 | 1.29E-04 |
| rs2261181 | -0.08 | 0.02 | 8.54E-05 |
| rs2294120 | -0.08 | 0.02 | 1.13E-04 |
| rs2296173 | -0.08 | 0.02 | 1.16E-04 |
| rs2299383 | -0.08 | 0.02 | 1.08E-04 |
| rs243019 | -0.08 | 0.02 | 7.92E-05 |
| rs2493394 | -0.08 | 0.02 | 6.33E-05 |
| rs2796441 | -0.08 | 0.02 | 1.28E-04 |
| rs2820426 | -0.08 | 0.02 | 1.08E-04 |
| rs2867125 | -0.08 | 0.02 | 1.22E-04 |
| rs2908282 | -0.08 | 0.02 | 9.15E-05 |
| rs2925979 | -0.08 | 0.02 | 1.08E-04 |
| rs2943656 | -0.07 | 0.02 | 2.60E-04 |
| rs3217992 | -0.08 | 0.02 | 4.33E-05 |
| rs340874 | -0.08 | 0.02 | 1.29E-04 |
| rs348330 | -0.08 | 0.02 | 9.59E-05 |
| rs3756784 | -0.08 | 0.02 | 1.14E-04 |
| rs3802177 | -0.08 | 0.02 | 5.50E-05 |
| rs459193 | -0.08 | 0.02 | 1.86E-04 |
| rs4622883 | -0.08 | 0.02 | 1.00E-04 |
| rs4686471 | -0.08 | 0.02 | 1.08E-04 |
| rs4812829 | -0.08 | 0.02 | 8.30E-05 |
| rs4823182 | -0.08 | 0.02 | 1.17E-04 |
| rs4865796 | -0.08 | 0.02 | 1.27E-04 |
| rs516946 | -0.08 | 0.02 | 8.90E-05 |
| rs5215 | -0.08 | 0.02 | 4.66E-05 |
| rs55966194 | -0.08 | 0.02 | 8.02E-05 |
| rs576674 | -0.08 | 0.02 | 1.05E-04 |
| rs6059662 | -0.08 | 0.02 | 1.18E-04 |
| rs61953351 | -0.08 | 0.02 | 6.17E-05 |
| rs622217 | -0.08 | 0.02 | 1.31E-04 |
| rs6494307 | -0.08 | 0.02 | 1.10E-04 |
| rs6515236 | -0.08 | 0.02 | 1.04E-04 |
| rs67232546 | -0.08 | 0.02 | 4.73E-05 |
| rs6767484 | -0.08 | 0.02 | 1.97E-04 |
| rs6785040 | -0.08 | 0.02 | 8.96E-05 |
| rs6795735 | -0.08 | 0.02 | 1.07E-04 |
| rs6878122 | -0.08 | 0.02 | 9.20E-05 |
| rs6960043 | -0.08 | 0.02 | 9.08E-05 |
| rs7144011 | -0.08 | 0.02 | 1.16E-04 |
| rs7177055 | -0.08 | 0.02 | 1.16E-04 |
| rs7240767 | -0.08 | 0.02 | 1.01E-04 |
| rs72802358 | -0.08 | 0.02 | 1.66E-04 |
| rs72892910 | -0.08 | 0.02 | 8.57E-05 |
| rs735949 | -0.08 | 0.02 | 1.11E-04 |
| rs753270 | -0.08 | 0.02 | 1.19E-04 |
| rs7561798 | -0.08 | 0.02 | 1.14E-04 |
| rs7572970 | -0.08 | 0.02 | 5.98E-05 |
| rs7607777 | -0.08 | 0.02 | 7.83E-05 |
| rs7674212 | -0.08 | 0.02 | 1.16E-04 |
| rs7685296 | -0.08 | 0.02 | 1.29E-04 |
| rs7729395 | -0.08 | 0.02 | 5.90E-05 |
| rs7756992 | -0.08 | 0.02 | 1.31E-04 |
| rs7786095 | -0.08 | 0.02 | 1.08E-04 |
| rs780094 | -0.08 | 0.02 | 8.28E-05 |
| rs7845219 | -0.08 | 0.02 | 6.21E-05 |
| rs7903146 | -0.08 | 0.02 | 6.29E-04 |
| rs7929543 | -0.08 | 0.02 | 1.12E-04 |
| rs7955901 | -0.08 | 0.02 | 1.01E-04 |
| rs8068804 | -0.08 | 0.02 | 1.06E-04 |
| rs8108269 | -0.08 | 0.02 | 7.22E-05 |
| rs825476 | -0.08 | 0.02 | 1.36E-04 |
| rs840967 | -0.08 | 0.02 | 7.73E-05 |
| rs849135 | -0.08 | 0.02 | 1.13E-04 |
| rs853974 | -0.08 | 0.02 | 1.13E-04 |
| rs9369425 | -0.08 | 0.02 | 5.01E-05 |
| rs963740 | -0.08 | 0.02 | 8.70E-05 |
| rs9844972 | -0.08 | 0.02 | 9.24E-05 |
| rs9894220 | -0.08 | 0.02 | 1.26E-04 |
| rs9928094 | -0.08 | 0.02 | 1.08E-04 |
| rs993380 | -0.08 | 0.02 | 1.02E-04 |
| rs9940149 | -0.08 | 0.02 | 1.19E-04 |
| All | -0.08 | 0.02 | 9.03E-05 |

T2DM: Type-2 diabetes; HDL: High-density lipoprotein; Se: Standard error.

Table 45: Single SNP analysis for estimate of T2DM-LDL(Willer).

| SNP | Effect | Se | P-value |
| --- | --- | --- | --- |
| rs10077431 | -0.13 | 0.10 | 1.80E-01 |
| rs10100265 | 0.15 | 0.08 | 5.47E-02 |
| rs10114341 | 0.10 | 0.09 | 2.43E-01 |
| rs10401969 | -1.29 | 0.08 | 9.19E-61 |
| rs1050226 | -0.03 | 0.08 | 6.74E-01 |
| rs1061813 | -0.02 | 0.09 | 8.33E-01 |
| rs1063355 | -0.33 | 0.08 | 1.03E-04 |
| rs10740322 | 0.10 | 0.12 | 4.26E-01 |
| rs10811661 | -0.02 | 0.03 | 6.10E-01 |
| rs10830963 | 0.01 | 0.05 | 7.93E-01 |
| rs10842994 | 0.04 | 0.06 | 5.10E-01 |
| rs10974438 | 0.05 | 0.07 | 4.57E-01 |
| rs11098676 | -0.03 | 0.12 | 8.00E-01 |
| rs11107116 | -0.16 | 0.09 | 8.41E-02 |
| rs1111875 | 0.08 | 0.04 | 4.85E-02 |
| rs11257655 | 0.06 | 0.06 | 2.85E-01 |
| rs1127655 | 0.03 | 0.12 | 8.17E-01 |
| rs11708067 | 0.14 | 0.05 | 2.16E-03 |
| rs11925227 | 0.19 | 0.09 | 4.13E-02 |
| rs11926707 | 0.13 | 0.12 | 2.76E-01 |
| rs12088739 | -0.01 | 0.07 | 8.51E-01 |
| rs12299509 | 0.08 | 0.08 | 3.30E-01 |
| rs12617659 | -0.12 | 0.08 | 1.19E-01 |
| rs12910825 | 0.02 | 0.07 | 8.13E-01 |
| rs12945601 | 0.00 | 0.11 | 9.70E-01 |
| rs12970134 | -0.03 | 0.08 | 7.33E-01 |
| rs13234269 | 0.20 | 0.09 | 3.15E-02 |
| rs13239186 | 0.04 | 0.10 | 7.11E-01 |
| rs13330951 | 0.00 | 0.11 | 9.69E-01 |
| rs13389219 | 0.30 | 0.05 | 2.75E-09 |
| rs1359790 | -0.03 | 0.05 | 5.75E-01 |
| rs1496653 | 0.04 | 0.06 | 4.43E-01 |
| rs1552224 | 0.00 | 0.05 | 9.35E-01 |
| rs16988333 | 0.12 | 0.09 | 1.82E-01 |
| rs17086692 | -0.03 | 0.12 | 7.79E-01 |
| rs17168486 | -0.03 | 0.06 | 6.17E-01 |
| rs17405722 | 0.05 | 0.11 | 6.71E-01 |
| rs17411031 | 0.21 | 0.09 | 2.05E-02 |
| rs1758632 | -0.03 | 0.11 | 8.06E-01 |
| rs17631783 | 0.18 | 0.12 | 1.36E-01 |
| rs17791513 | 0.03 | 0.07 | 7.04E-01 |
| rs1801214 | -0.01 | 0.04 | 7.32E-01 |
| rs1899951 | 0.03 | 0.05 | 4.90E-01 |
| rs2058913 | -0.13 | 0.11 | 2.35E-01 |
| rs2237892 | 0.04 | 0.09 | 6.00E-01 |
| rs2246618 | -0.02 | 0.08 | 8.16E-01 |
| rs2261181 | 0.08 | 0.06 | 2.40E-01 |
| rs2294120 | -0.05 | 0.11 | 6.68E-01 |
| rs2296173 | 0.10 | 0.07 | 1.49E-01 |
| rs2299383 | -0.04 | 0.09 | 6.46E-01 |
| rs243019 | 0.01 | 0.07 | 8.54E-01 |
| rs2493394 | 0.00 | 0.08 | 9.72E-01 |
| rs2796441 | 0.05 | 0.05 | 3.97E-01 |
| rs2820426 | 0.24 | 0.07 | 7.29E-04 |
| rs2867125 | -0.18 | 0.08 | 2.44E-02 |
| rs2908282 | -0.04 | 0.09 | 6.31E-01 |
| rs2925979 | -0.06 | 0.07 | 4.38E-01 |
| rs2943656 | 0.07 | 0.04 | 8.86E-02 |
| rs3217992 | -0.02 | 0.07 | 8.13E-01 |
| rs340874 | 0.11 | 0.06 | 7.02E-02 |
| rs3756784 | -0.16 | 0.09 | 7.74E-02 |
| rs3802177 | 0.08 | 0.03 | 1.43E-02 |
| rs459193 | -0.15 | 0.06 | 9.06E-03 |
| rs4622883 | -0.15 | 0.12 | 2.27E-01 |
| rs4812829 | -0.04 | 0.09 | 6.39E-01 |
| rs4823182 | -0.28 | 0.08 | 3.45E-04 |
| rs4865796 | 0.07 | 0.08 | 3.30E-01 |
| rs516946 | -0.04 | 0.05 | 4.29E-01 |
| rs5215 | 0.11 | 0.06 | 4.55E-02 |
| rs55966194 | 0.17 | 0.12 | 1.54E-01 |
| rs576674 | -0.04 | 0.08 | 6.44E-01 |
| rs6059662 | -0.17 | 0.09 | 5.12E-02 |
| rs622217 | 0.44 | 0.10 | 1.66E-05 |
| rs6494307 | -0.20 | 0.12 | 9.68E-02 |
| rs6515236 | -0.20 | 0.13 | 1.05E-01 |
| rs6767484 | -0.02 | 0.03 | 5.90E-01 |
| rs6785040 | -0.05 | 0.12 | 6.78E-01 |
| rs6795735 | 0.11 | 0.07 | 1.11E-01 |
| rs6878122 | 0.15 | 0.08 | 4.30E-02 |
| rs6960043 | 0.00 | 0.06 | 9.58E-01 |
| rs7144011 | -0.10 | 0.09 | 2.56E-01 |
| rs7177055 | 0.08 | 0.06 | 1.94E-01 |
| rs7240767 | 0.15 | 0.12 | 2.23E-01 |
| rs72802358 | 0.03 | 0.05 | 5.36E-01 |
| rs72892910 | 0.08 | 0.10 | 4.65E-01 |
| rs735949 | -0.11 | 0.07 | 1.26E-01 |
| rs753270 | 0.04 | 0.10 | 6.84E-01 |
| rs7561798 | 0.10 | 0.09 | 2.91E-01 |
| rs7572970 | -0.03 | 0.10 | 7.43E-01 |
| rs7607777 | 0.06 | 0.05 | 1.74E-01 |
| rs7685296 | -0.03 | 0.08 | 7.33E-01 |
| rs7729395 | 0.03 | 0.07 | 6.24E-01 |
| rs7756992 | 0.03 | 0.03 | 4.09E-01 |
| rs7786095 | 0.17 | 0.11 | 1.32E-01 |
| rs780094 | -0.30 | 0.05 | 1.18E-08 |
| rs7845219 | 0.03 | 0.09 | 7.39E-01 |
| rs7903146 | 0.02 | 0.01 | 1.80E-01 |
| rs7929543 | -0.04 | 0.09 | 6.95E-01 |
| rs7955901 | 0.10 | 0.08 | 2.34E-01 |
| rs8068804 | 0.17 | 0.07 | 1.80E-02 |
| rs8108269 | -0.25 | 0.07 | 1.69E-04 |
| rs825476 | 0.02 | 0.07 | 7.66E-01 |
| rs840967 | 0.12 | 0.11 | 2.75E-01 |
| rs849135 | -0.02 | 0.04 | 5.89E-01 |
| rs9369425 | -0.21 | 0.11 | 4.74E-02 |
| rs963740 | -0.06 | 0.12 | 6.17E-01 |
| rs9844972 | 0.10 | 0.10 | 3.32E-01 |
| rs9894220 | 0.00 | 0.10 | 9.72E-01 |
| rs9928094 | 0.00 | 0.04 | 1.00E+00 |
| rs993380 | 0.03 | 0.10 | 7.63E-01 |
| rs9940149 | -0.02 | 0.09 | 8.57E-01 |
| All - Inverse variance weighted | 0.01 | 0.01 | 4.20E-01 |
| All - MR Egger | 0.01 | 0.03 | 6.07E-01 |

T2DM: Type-2 diabetes; LDL: Low-density lipoprotein; Se: Standard error.

Table 46: Leave-one-out analysis for estimate of T2DM-LDL(Willer).

| SNP | Effect | Se | P-value |
| --- | --- | --- | --- |
| rs10077431 | 0.01 | 0.01 | 4.00E-01 |
| rs10100265 | 0.01 | 0.01 | 4.57E-01 |
| rs10114341 | 0.01 | 0.01 | 4.41E-01 |
| rs10401969 | 0.02 | 0.01 | 5.69E-02 |
| rs1050226 | 0.01 | 0.01 | 4.13E-01 |
| rs1061813 | 0.01 | 0.01 | 4.18E-01 |
| rs1063355 | 0.01 | 0.01 | 3.50E-01 |
| rs10740322 | 0.01 | 0.01 | 4.31E-01 |
| rs10811661 | 0.01 | 0.01 | 3.90E-01 |
| rs10830963 | 0.01 | 0.01 | 4.27E-01 |
| rs10842994 | 0.01 | 0.01 | 4.36E-01 |
| rs10974438 | 0.01 | 0.01 | 4.37E-01 |
| rs11098676 | 0.01 | 0.01 | 4.19E-01 |
| rs11107116 | 0.01 | 0.01 | 3.94E-01 |
| rs1111875 | 0.01 | 0.01 | 4.95E-01 |
| rs11257655 | 0.01 | 0.01 | 4.47E-01 |
| rs1127655 | 0.01 | 0.01 | 4.25E-01 |
| rs11708067 | 0.01 | 0.01 | 5.19E-01 |
| rs11925227 | 0.01 | 0.01 | 4.54E-01 |
| rs11926707 | 0.01 | 0.01 | 4.35E-01 |
| rs12088739 | 0.01 | 0.01 | 4.17E-01 |
| rs12299509 | 0.01 | 0.01 | 4.38E-01 |
| rs12617659 | 0.01 | 0.01 | 3.90E-01 |
| rs12910825 | 0.01 | 0.01 | 4.26E-01 |
| rs12945601 | 0.01 | 0.01 | 4.22E-01 |
| rs12970134 | 0.01 | 0.01 | 4.15E-01 |
| rs13234269 | 0.01 | 0.01 | 4.56E-01 |
| rs13239186 | 0.01 | 0.01 | 4.27E-01 |
| rs13330951 | 0.01 | 0.01 | 4.21E-01 |
| rs13389219 | 0.01 | 0.01 | 5.99E-01 |
| rs1359790 | 0.01 | 0.01 | 4.03E-01 |
| rs1496653 | 0.01 | 0.01 | 4.40E-01 |
| rs1552224 | 0.01 | 0.01 | 4.16E-01 |
| rs16988333 | 0.01 | 0.01 | 4.43E-01 |
| rs17086692 | 0.01 | 0.01 | 4.18E-01 |
| rs17168486 | 0.01 | 0.01 | 4.09E-01 |
| rs17405722 | 0.01 | 0.01 | 4.27E-01 |
| rs17411031 | 0.01 | 0.01 | 4.58E-01 |
| rs1758632 | 0.01 | 0.01 | 4.18E-01 |
| rs17631783 | 0.01 | 0.01 | 4.39E-01 |
| rs17791513 | 0.01 | 0.01 | 4.29E-01 |
| rs1801214 | 0.01 | 0.01 | 4.06E-01 |
| rs1899951 | 0.01 | 0.01 | 4.40E-01 |
| rs2058913 | 0.01 | 0.01 | 4.05E-01 |
| rs2237892 | 0.01 | 0.01 | 4.30E-01 |
| rs2246618 | 0.01 | 0.01 | 4.17E-01 |
| rs2261181 | 0.01 | 0.01 | 4.48E-01 |
| rs2294120 | 0.01 | 0.01 | 4.16E-01 |
| rs2296173 | 0.01 | 0.01 | 4.52E-01 |
| rs2299383 | 0.01 | 0.01 | 4.14E-01 |
| rs243019 | 0.01 | 0.01 | 4.25E-01 |
| rs2493394 | 0.01 | 0.01 | 4.22E-01 |
| rs2796441 | 0.01 | 0.01 | 4.43E-01 |
| rs2820426 | 0.01 | 0.01 | 4.90E-01 |
| rs2867125 | 0.01 | 0.01 | 3.78E-01 |
| rs2908282 | 0.01 | 0.01 | 4.14E-01 |
| rs2925979 | 0.01 | 0.01 | 4.06E-01 |
| rs2943656 | 0.01 | 0.01 | 4.81E-01 |
| rs3217992 | 0.01 | 0.01 | 4.16E-01 |
| rs340874 | 0.01 | 0.01 | 4.66E-01 |
| rs3756784 | 0.01 | 0.01 | 3.93E-01 |
| rs3802177 | 0.01 | 0.01 | 5.32E-01 |
| rs459193 | 0.01 | 0.01 | 3.53E-01 |
| rs4622883 | 0.01 | 0.01 | 4.07E-01 |
| rs4812829 | 0.01 | 0.01 | 4.14E-01 |
| rs4823182 | 0.01 | 0.01 | 3.51E-01 |
| rs4865796 | 0.01 | 0.01 | 4.40E-01 |
| rs516946 | 0.01 | 0.01 | 3.97E-01 |
| rs5215 | 0.01 | 0.01 | 4.73E-01 |
| rs55966194 | 0.01 | 0.01 | 4.39E-01 |
| rs576674 | 0.01 | 0.01 | 4.13E-01 |
| rs6059662 | 0.01 | 0.01 | 3.88E-01 |
| rs622217 | 0.01 | 0.01 | 4.80E-01 |
| rs6494307 | 0.01 | 0.01 | 4.00E-01 |
| rs6515236 | 0.01 | 0.01 | 4.02E-01 |
| rs6767484 | 0.01 | 0.01 | 3.90E-01 |
| rs6785040 | 0.01 | 0.01 | 4.17E-01 |
| rs6795735 | 0.01 | 0.01 | 4.56E-01 |
| rs6878122 | 0.01 | 0.01 | 4.60E-01 |
| rs6960043 | 0.01 | 0.01 | 4.22E-01 |
| rs7144011 | 0.01 | 0.01 | 4.03E-01 |
| rs7177055 | 0.01 | 0.01 | 4.52E-01 |
| rs7240767 | 0.01 | 0.01 | 4.36E-01 |
| rs72802358 | 0.01 | 0.01 | 4.39E-01 |
| rs72892910 | 0.01 | 0.01 | 4.32E-01 |
| rs735949 | 0.01 | 0.01 | 3.90E-01 |
| rs753270 | 0.01 | 0.01 | 4.28E-01 |
| rs7561798 | 0.01 | 0.01 | 4.39E-01 |
| rs7572970 | 0.01 | 0.01 | 4.17E-01 |
| rs7607777 | 0.01 | 0.01 | 4.63E-01 |
| rs7685296 | 0.01 | 0.01 | 4.15E-01 |
| rs7729395 | 0.01 | 0.01 | 4.31E-01 |
| rs7756992 | 0.01 | 0.01 | 4.55E-01 |
| rs7786095 | 0.01 | 0.01 | 4.41E-01 |
| rs780094 | 0.01 | 0.01 | 2.64E-01 |
| rs7845219 | 0.01 | 0.01 | 4.27E-01 |
| rs7903146 | 0.01 | 0.01 | 5.45E-01 |
| rs7929543 | 0.01 | 0.01 | 4.15E-01 |
| rs7955901 | 0.01 | 0.01 | 4.42E-01 |
| rs8068804 | 0.01 | 0.01 | 4.71E-01 |
| rs8108269 | 0.01 | 0.01 | 3.34E-01 |
| rs825476 | 0.01 | 0.01 | 4.27E-01 |
| rs840967 | 0.01 | 0.01 | 4.36E-01 |
| rs849135 | 0.01 | 0.01 | 3.95E-01 |
| rs9369425 | 0.01 | 0.01 | 3.93E-01 |
| rs963740 | 0.01 | 0.01 | 4.16E-01 |
| rs9844972 | 0.01 | 0.01 | 4.35E-01 |
| rs9894220 | 0.01 | 0.01 | 4.22E-01 |
| rs9928094 | 0.01 | 0.01 | 4.17E-01 |
| rs993380 | 0.01 | 0.01 | 4.26E-01 |
| rs9940149 | 0.01 | 0.01 | 4.18E-01 |
| All | 0.01 | 0.01 | 4.20E-01 |

T2DM: Type-2 diabetes; LDL: Low-density lipoprotein; Se: Standard error.

Table 47: Single SNP analysis for estimate of T2DM-LDL(Prins).

| SNP | Effect | Se | P-value |
| --- | --- | --- | --- |
| rs10077431 | 0.21 | 0.41 | 6.17E-01 |
| rs10100265 | 0.61 | 0.41 | 1.34E-01 |
| rs10114341 | 0.24 | 0.24 | 3.17E-01 |
| rs10401969 | -1.30 | 0.33 | 6.33E-05 |
| rs1050226 | 0.20 | 0.20 | 3.17E-01 |
| rs1061813 | -0.47 | 0.23 | 4.55E-02 |
| rs1063355 | -0.14 | 0.28 | 6.17E-01 |
| rs10740322 | -0.84 | 0.42 | 4.55E-02 |
| rs10811661 | -0.32 | 0.13 | 1.24E-02 |
| rs10830963 | -0.11 | 0.22 | 6.17E-01 |
| rs10842994 | -0.13 | 0.26 | 6.17E-01 |
| rs10974438 | -0.17 | 0.34 | 6.17E-01 |
| rs11107116 | 0.64 | 0.43 | 1.34E-01 |
| rs1111875 | 0.11 | 0.11 | 3.17E-01 |
| rs11257655 | 0.14 | 0.27 | 6.17E-01 |
| rs1127655 | -0.46 | 0.23 | 4.55E-02 |
| rs11708067 | 0.21 | 0.21 | 3.17E-01 |
| rs12088739 | -0.11 | 0.34 | 7.39E-01 |
| rs12617659 | -0.15 | 0.29 | 6.17E-01 |
| rs12910825 | 0.58 | 0.39 | 1.34E-01 |
| rs12945601 | 0.21 | 0.42 | 6.17E-01 |
| rs12970134 | 0.36 | 0.36 | 3.17E-01 |
| rs13234269 | -0.51 | 0.17 | 2.70E-03 |
| rs13239186 | -0.37 | 0.37 | 3.17E-01 |
| rs13389219 | -0.28 | 0.28 | 3.17E-01 |
| rs1359790 | -0.25 | 0.25 | 3.17E-01 |
| rs1496653 | 0.13 | 0.26 | 6.17E-01 |
| rs1552224 | 0.19 | 0.19 | 3.17E-01 |
| rs16988333 | -0.13 | 0.27 | 6.17E-01 |
| rs17086692 | -0.43 | 0.43 | 3.17E-01 |
| rs17168486 | -0.13 | 0.27 | 6.17E-01 |
| rs17405722 | 0.23 | 0.34 | 5.05E-01 |
| rs17411031 | 0.22 | 0.44 | 6.17E-01 |
| rs1758632 | -0.41 | 0.20 | 4.55E-02 |
| rs17631783 | -0.21 | 0.41 | 6.17E-01 |
| rs17791513 | 0.49 | 0.29 | 9.56E-02 |
| rs1801214 | -0.11 | 0.11 | 3.17E-01 |
| rs1899951 | 0.09 | 0.18 | 6.17E-01 |
| rs2058913 | 0.41 | 0.20 | 4.55E-02 |
| rs2237892 | -0.42 | 0.31 | 1.82E-01 |
| rs2246618 | -0.19 | 0.39 | 6.17E-01 |
| rs2261181 | -0.20 | 0.20 | 3.17E-01 |
| rs2294120 | 0.23 | 0.23 | 3.17E-01 |
| rs2296173 | -0.62 | 0.31 | 4.55E-02 |
| rs2299383 | 0.24 | 0.24 | 3.17E-01 |
| rs243019 | -0.18 | 0.18 | 3.17E-01 |
| rs2493394 | 0.14 | 0.27 | 6.17E-01 |
| rs2796441 | -0.28 | 0.14 | 4.55E-02 |
| rs2820426 | 0.19 | 0.38 | 6.17E-01 |
| rs2867125 | 0.17 | 0.33 | 6.17E-01 |
| rs2908282 | -0.18 | 0.36 | 6.17E-01 |
| rs2925979 | 0.19 | 0.37 | 6.17E-01 |
| rs2943656 | -0.22 | 0.22 | 3.17E-01 |
| rs3217992 | 0.19 | 0.38 | 6.17E-01 |
| rs340874 | -0.16 | 0.16 | 3.17E-01 |
| rs3756784 | -0.20 | 0.40 | 6.17E-01 |
| rs3802177 | -0.08 | 0.16 | 6.17E-01 |
| rs459193 | 0.28 | 0.28 | 3.17E-01 |
| rs4622883 | 0.23 | 0.23 | 3.17E-01 |
| rs4686471 | -0.19 | 0.19 | 3.17E-01 |
| rs4812829 | 0.19 | 0.38 | 6.17E-01 |
| rs4865796 | 0.19 | 0.38 | 6.17E-01 |
| rs516946 | -0.12 | 0.24 | 6.17E-01 |
| rs5215 | 0.15 | 0.29 | 6.17E-01 |
| rs55966194 | -0.19 | 0.38 | 6.17E-01 |
| rs576674 | 0.15 | 0.31 | 6.17E-01 |
| rs6059662 | -0.45 | 0.45 | 3.17E-01 |
| rs61953351 | 0.29 | 0.29 | 3.17E-01 |
| rs6494307 | -0.45 | 0.23 | 4.55E-02 |
| rs67232546 | 0.34 | 0.34 | 3.17E-01 |
| rs6767484 | 0.17 | 0.17 | 3.17E-01 |
| rs6785040 | -0.16 | 0.32 | 6.17E-01 |
| rs6795735 | -0.18 | 0.18 | 3.17E-01 |
| rs6960043 | -0.16 | 0.16 | 3.17E-01 |
| rs7144011 | -0.21 | 0.41 | 6.17E-01 |
| rs7177055 | 0.31 | 0.31 | 3.17E-01 |
| rs7240767 | -0.22 | 0.22 | 3.17E-01 |
| rs72892910 | 0.62 | 0.31 | 4.55E-02 |
| rs735949 | -0.42 | 0.28 | 1.34E-01 |
| rs7572970 | 0.17 | 0.34 | 6.17E-01 |
| rs7607777 | -0.07 | 0.15 | 6.17E-01 |
| rs7674212 | 0.22 | 0.43 | 6.17E-01 |
| rs7685296 | -0.59 | 0.39 | 1.34E-01 |
| rs7729395 | 0.07 | 0.22 | 7.39E-01 |
| rs7756992 | 0.08 | 0.15 | 6.17E-01 |
| rs7786095 | -0.94 | 0.27 | 4.65E-04 |
| rs780094 | -0.14 | 0.14 | 3.17E-01 |
| rs7845219 | 0.24 | 0.24 | 3.17E-01 |
| rs7903146 | -0.10 | 0.07 | 1.34E-01 |
| rs7929543 | -0.60 | 0.36 | 9.56E-02 |
| rs7955901 | -0.23 | 0.23 | 3.17E-01 |
| rs8068804 | 0.17 | 0.34 | 6.17E-01 |
| rs8108269 | 0.31 | 0.31 | 3.17E-01 |
| rs825476 | -0.19 | 0.19 | 3.17E-01 |
| rs840967 | 0.20 | 0.20 | 3.17E-01 |
| rs849135 | 0.10 | 0.10 | 3.17E-01 |
| rs853974 | -0.33 | 0.33 | 3.17E-01 |
| rs9369425 | 0.18 | 0.37 | 6.17E-01 |
| rs963740 | -0.21 | 0.42 | 6.17E-01 |
| rs9844972 | -0.10 | 0.31 | 7.39E-01 |
| rs9894220 | -0.17 | 0.17 | 3.17E-01 |
| rs9928094 | -0.10 | 0.19 | 6.17E-01 |
| rs9940149 | 0.34 | 0.34 | 3.17E-01 |
| All - Inverse variance weighted | -0.06 | 0.03 | 1.19E-02 |
| All - MR Egger | -0.08 | 0.06 | 2.34E-01 |

T2DM: Type-2 diabetes; LDL: Low-density lipoprotein; Se: Standard error.

Table 48: Leave-one-out analysis for estimate of T2DM-LDL(Prins).

| SNP | Effect | Se | P-value |
| --- | --- | --- | --- |
| rs10077431 | -0.06 | 0.03 | 1.14E-02 |
| rs10100265 | -0.07 | 0.03 | 9.36E-03 |
| rs10114341 | -0.07 | 0.03 | 9.28E-03 |
| rs10401969 | -0.06 | 0.02 | 1.59E-02 |
| rs1050226 | -0.07 | 0.03 | 8.72E-03 |
| rs1061813 | -0.06 | 0.03 | 1.73E-02 |
| rs1063355 | -0.06 | 0.03 | 1.32E-02 |
| rs10740322 | -0.06 | 0.03 | 1.44E-02 |
| rs10811661 | -0.06 | 0.03 | 2.73E-02 |
| rs10830963 | -0.06 | 0.03 | 1.34E-02 |
| rs10842994 | -0.06 | 0.03 | 1.33E-02 |
| rs10974438 | -0.06 | 0.03 | 1.31E-02 |
| rs11107116 | -0.07 | 0.03 | 9.47E-03 |
| rs1111875 | -0.07 | 0.03 | 5.78E-03 |
| rs11257655 | -0.06 | 0.03 | 1.08E-02 |
| rs1127655 | -0.06 | 0.03 | 1.74E-02 |
| rs11708067 | -0.07 | 0.03 | 8.78E-03 |
| rs12088739 | -0.06 | 0.03 | 1.28E-02 |
| rs12617659 | -0.06 | 0.03 | 1.32E-02 |
| rs12910825 | -0.07 | 0.03 | 9.25E-03 |
| rs12945601 | -0.06 | 0.03 | 1.14E-02 |
| rs12970134 | -0.07 | 0.03 | 1.02E-02 |
| rs13234269 | -0.06 | 0.02 | 2.41E-02 |
| rs13239186 | -0.06 | 0.03 | 1.38E-02 |
| rs13389219 | -0.06 | 0.03 | 1.44E-02 |
| rs1359790 | -0.06 | 0.03 | 1.46E-02 |
| rs1496653 | -0.06 | 0.03 | 1.07E-02 |
| rs1552224 | -0.07 | 0.03 | 8.55E-03 |
| rs16988333 | -0.06 | 0.03 | 1.33E-02 |
| rs17086692 | -0.06 | 0.03 | 1.36E-02 |
| rs17168486 | -0.06 | 0.03 | 1.33E-02 |
| rs17405722 | -0.06 | 0.03 | 1.08E-02 |
| rs17411031 | -0.06 | 0.03 | 1.14E-02 |
| rs1758632 | -0.06 | 0.03 | 1.83E-02 |
| rs17631783 | -0.06 | 0.03 | 1.30E-02 |
| rs17791513 | -0.07 | 0.03 | 8.11E-03 |
| rs1801214 | -0.06 | 0.03 | 1.71E-02 |
| rs1899951 | -0.07 | 0.03 | 9.92E-03 |
| rs2058913 | -0.07 | 0.03 | 5.95E-03 |
| rs2237892 | -0.06 | 0.03 | 1.47E-02 |
| rs2246618 | -0.06 | 0.03 | 1.30E-02 |
| rs2261181 | -0.06 | 0.03 | 1.51E-02 |
| rs2294120 | -0.07 | 0.03 | 9.05E-03 |
| rs2296173 | -0.06 | 0.03 | 1.57E-02 |
| rs2299383 | -0.07 | 0.03 | 9.26E-03 |
| rs243019 | -0.06 | 0.03 | 1.55E-02 |
| rs2493394 | -0.06 | 0.03 | 1.08E-02 |
| rs2796441 | -0.06 | 0.03 | 2.22E-02 |
| rs2820426 | -0.06 | 0.03 | 1.13E-02 |
| rs2867125 | -0.06 | 0.03 | 1.11E-02 |
| rs2908282 | -0.06 | 0.03 | 1.31E-02 |
| rs2925979 | -0.06 | 0.03 | 1.13E-02 |
| rs2943656 | -0.06 | 0.03 | 1.49E-02 |
| rs3217992 | -0.06 | 0.03 | 1.13E-02 |
| rs340874 | -0.06 | 0.03 | 1.59E-02 |
| rs3756784 | -0.06 | 0.03 | 1.30E-02 |
| rs3802177 | -0.06 | 0.03 | 1.36E-02 |
| rs459193 | -0.07 | 0.03 | 9.64E-03 |
| rs4622883 | -0.07 | 0.03 | 9.10E-03 |
| rs4686471 | -0.06 | 0.03 | 1.54E-02 |
| rs4812829 | -0.06 | 0.03 | 1.13E-02 |
| rs4865796 | -0.06 | 0.03 | 1.13E-02 |
| rs516946 | -0.06 | 0.03 | 1.33E-02 |
| rs5215 | -0.06 | 0.03 | 1.10E-02 |
| rs55966194 | -0.06 | 0.03 | 1.30E-02 |
| rs576674 | -0.06 | 0.03 | 1.10E-02 |
| rs6059662 | -0.06 | 0.03 | 1.35E-02 |
| rs61953351 | -0.07 | 0.03 | 9.68E-03 |
| rs6494307 | -0.06 | 0.03 | 1.75E-02 |
| rs67232546 | -0.07 | 0.03 | 1.00E-02 |
| rs6767484 | -0.07 | 0.03 | 7.97E-03 |
| rs6785040 | -0.06 | 0.03 | 1.32E-02 |
| rs6795735 | -0.06 | 0.03 | 1.55E-02 |
| rs6960043 | -0.06 | 0.03 | 1.59E-02 |
| rs7144011 | -0.06 | 0.03 | 1.30E-02 |
| rs7177055 | -0.07 | 0.03 | 9.86E-03 |
| rs7240767 | -0.06 | 0.03 | 1.49E-02 |
| rs72892910 | -0.07 | 0.03 | 7.50E-03 |
| rs735949 | -0.06 | 0.03 | 1.53E-02 |
| rs7572970 | -0.06 | 0.03 | 1.11E-02 |
| rs7607777 | -0.06 | 0.03 | 1.37E-02 |
| rs7674212 | -0.06 | 0.03 | 1.14E-02 |
| rs7685296 | -0.06 | 0.03 | 1.42E-02 |
| rs7729395 | -0.06 | 0.03 | 1.09E-02 |
| rs7756992 | -0.07 | 0.03 | 9.47E-03 |
| rs7786095 | -0.06 | 0.02 | 1.81E-02 |
| rs780094 | -0.06 | 0.03 | 1.62E-02 |
| rs7845219 | -0.07 | 0.03 | 9.19E-03 |
| rs7903146 | -0.06 | 0.03 | 2.73E-02 |
| rs7929543 | -0.06 | 0.03 | 1.46E-02 |
| rs7955901 | -0.06 | 0.03 | 1.48E-02 |
| rs8068804 | -0.06 | 0.03 | 1.11E-02 |
| rs8108269 | -0.07 | 0.03 | 9.87E-03 |
| rs825476 | -0.06 | 0.03 | 1.53E-02 |
| rs840967 | -0.07 | 0.03 | 8.68E-03 |
| rs849135 | -0.07 | 0.03 | 5.48E-03 |
| rs853974 | -0.06 | 0.03 | 1.40E-02 |
| rs9369425 | -0.06 | 0.03 | 1.12E-02 |
| rs963740 | -0.06 | 0.03 | 1.30E-02 |
| rs9844972 | -0.06 | 0.03 | 1.28E-02 |
| rs9894220 | -0.06 | 0.03 | 1.56E-02 |
| rs9928094 | -0.06 | 0.03 | 1.35E-02 |
| rs9940149 | -0.07 | 0.03 | 1.01E-02 |
| All | -0.06 | 0.03 | 1.19E-02 |

T2DM: Type-2 diabetes; LDL: Low-density lipoprotein; Se: Standard error.

Table 49: Single SNP analysis for estimate of T2DM-LDL(Kanai).

| SNP | Effect | Se | P-value |
| --- | --- | --- | --- |
| rs10077431 | -0.09 | 0.23 | 6.94E-01 |
| rs10087241 | 0.03 | 0.21 | 8.72E-01 |
| rs10100265 | 0.27 | 0.11 | 1.50E-02 |
| rs10114341 | 0.22 | 0.17 | 1.97E-01 |
| rs10401969 | 0.15 | 0.09 | 1.10E-01 |
| rs1050226 | -0.25 | 0.11 | 2.17E-02 |
| rs1061813 | -0.17 | 0.22 | 4.24E-01 |
| rs1063355 | -0.18 | 0.07 | 1.23E-02 |
| rs10740322 | -0.14 | 0.12 | 2.61E-01 |
| rs10811661 | 0.02 | 0.04 | 5.90E-01 |
| rs10830963 | -0.06 | 0.06 | 2.64E-01 |
| rs10842994 | -0.04 | 0.09 | 6.21E-01 |
| rs10974438 | 0.16 | 0.09 | 8.21E-02 |
| rs11098676 | 0.40 | 0.21 | 6.31E-02 |
| rs11107116 | -0.14 | 0.12 | 2.37E-01 |
| rs1111875 | -0.05 | 0.06 | 3.75E-01 |
| rs11257655 | 0.06 | 0.07 | 3.72E-01 |
| rs1127655 | -0.17 | 0.12 | 1.56E-01 |
| rs11926707 | 0.06 | 0.14 | 6.47E-01 |
| rs12088739 | 0.08 | 0.11 | 4.68E-01 |
| rs12299509 | -0.10 | 0.11 | 3.52E-01 |
| rs12617659 | -0.27 | 0.11 | 1.46E-02 |
| rs12945601 | 0.22 | 0.16 | 1.76E-01 |
| rs12970134 | 0.14 | 0.13 | 2.66E-01 |
| rs13234269 | -0.01 | 0.11 | 9.34E-01 |
| rs13239186 | -0.05 | 0.14 | 7.16E-01 |
| rs13330951 | 0.28 | 0.17 | 9.29E-02 |
| rs13389219 | -0.10 | 0.13 | 4.64E-01 |
| rs1359790 | 0.10 | 0.08 | 1.94E-01 |
| rs1496653 | -0.04 | 0.09 | 6.64E-01 |
| rs1552224 | -0.05 | 0.13 | 6.98E-01 |
| rs17086692 | 0.00 | 0.13 | 9.93E-01 |
| rs17168486 | -0.06 | 0.07 | 4.19E-01 |
| rs17405722 | 0.28 | 0.19 | 1.30E-01 |
| rs17411031 | -0.22 | 0.15 | 1.44E-01 |
| rs1758632 | 0.12 | 0.12 | 3.27E-01 |
| rs17631783 | -0.16 | 0.23 | 4.91E-01 |
| rs17791513 | 0.09 | 0.11 | 4.12E-01 |
| rs1801214 | 0.32 | 0.23 | 1.67E-01 |
| rs1899951 | 0.07 | 0.13 | 6.18E-01 |
| rs2058913 | -0.01 | 0.21 | 9.53E-01 |
| rs2237892 | 0.08 | 0.06 | 1.44E-01 |
| rs2246618 | -0.02 | 0.14 | 9.04E-01 |
| rs2261181 | 0.01 | 0.08 | 9.20E-01 |
| rs2294120 | -0.11 | 0.14 | 4.13E-01 |
| rs2296173 | 0.03 | 0.11 | 7.71E-01 |
| rs2299383 | 0.07 | 0.13 | 5.70E-01 |
| rs243019 | 0.12 | 0.10 | 2.37E-01 |
| rs2493394 | 0.31 | 0.27 | 2.60E-01 |
| rs2796441 | -0.04 | 0.07 | 5.89E-01 |
| rs2820426 | 0.00 | 0.10 | 9.81E-01 |
| rs2867125 | 0.01 | 0.15 | 9.40E-01 |
| rs2908282 | -0.14 | 0.12 | 2.37E-01 |
| rs2925979 | -0.04 | 0.10 | 6.95E-01 |
| rs2943656 | -0.04 | 0.10 | 6.97E-01 |
| rs3217992 | -0.03 | 0.10 | 7.78E-01 |
| rs340874 | 0.03 | 0.09 | 7.14E-01 |
| rs348330 | -0.14 | 0.14 | 2.94E-01 |
| rs3756784 | -0.05 | 0.11 | 6.47E-01 |
| rs3802177 | 0.01 | 0.04 | 7.33E-01 |
| rs459193 | -0.13 | 0.07 | 8.72E-02 |
| rs4622883 | -0.05 | 0.14 | 7.34E-01 |
| rs4812829 | 0.15 | 0.10 | 1.18E-01 |
| rs4823182 | 0.00 | 0.11 | 9.82E-01 |
| rs4865796 | -0.13 | 0.13 | 3.31E-01 |
| rs516946 | 0.10 | 0.09 | 2.84E-01 |
| rs5215 | 0.01 | 0.08 | 8.53E-01 |
| rs576674 | 0.01 | 0.12 | 9.50E-01 |
| rs6059662 | 0.20 | 0.15 | 1.79E-01 |
| rs622217 | -0.09 | 0.12 | 4.74E-01 |
| rs6494307 | 0.15 | 0.12 | 1.96E-01 |
| rs6515236 | -0.12 | 0.11 | 2.73E-01 |
| rs6767484 | -0.09 | 0.05 | 5.25E-02 |
| rs6785040 | 0.16 | 0.09 | 6.83E-02 |
| rs6795735 | -0.12 | 0.13 | 3.50E-01 |
| rs6878122 | -0.43 | 0.35 | 2.16E-01 |
| rs6960043 | -0.03 | 0.08 | 7.12E-01 |
| rs7177055 | -0.05 | 0.08 | 5.25E-01 |
| rs7240767 | 0.11 | 0.13 | 3.79E-01 |
| rs72802358 | 0.16 | 0.18 | 3.58E-01 |
| rs72892910 | 0.02 | 0.10 | 8.24E-01 |
| rs753270 | 0.24 | 0.13 | 5.66E-02 |
| rs7561798 | 0.20 | 0.14 | 1.62E-01 |
| rs7572970 | -0.04 | 0.12 | 7.35E-01 |
| rs7607777 | -0.04 | 0.20 | 8.56E-01 |
| rs7674212 | -0.18 | 0.12 | 1.54E-01 |
| rs7685296 | 0.07 | 0.10 | 5.11E-01 |
| rs7756992 | -0.05 | 0.04 | 1.84E-01 |
| rs7786095 | 0.22 | 0.14 | 1.12E-01 |
| rs780094 | -0.23 | 0.08 | 1.97E-03 |
| rs7845219 | 0.05 | 0.14 | 6.93E-01 |
| rs7903146 | 0.03 | 0.04 | 4.38E-01 |
| rs7929543 | -0.09 | 0.09 | 3.17E-01 |
| rs7955901 | 0.02 | 0.12 | 8.84E-01 |
| rs8068804 | -0.19 | 0.10 | 4.88E-02 |
| rs8108269 | 0.31 | 0.08 | 1.77E-04 |
| rs825476 | 0.04 | 0.12 | 7.23E-01 |
| rs840967 | 0.09 | 0.11 | 4.09E-01 |
| rs853974 | 0.12 | 0.09 | 1.81E-01 |
| rs9369425 | -0.36 | 0.15 | 1.95E-02 |
| rs963740 | 0.07 | 0.11 | 4.98E-01 |
| rs9894220 | 0.09 | 0.10 | 3.71E-01 |
| rs9928094 | 0.06 | 0.06 | 3.50E-01 |
| rs993380 | 0.05 | 0.11 | 6.25E-01 |
| rs9940149 | -0.05 | 0.09 | 6.17E-01 |
| All - Inverse variance weighted | 0.00 | 0.01 | 9.78E-01 |
| All - MR Egger | 0.00 | 0.03 | 9.18E-01 |

T2DM: Type-2 diabetes; LDL: Low-density lipoprotein; Se: Standard error.

Table 50: Leave-one-out analysis for estimate of T2DM-LDL(Kanai).

| SNP | Effect | Se | P-value |
| --- | --- | --- | --- |
| rs10077431 | 0.00 | 0.01 | 9.67E-01 |
| rs10087241 | 0.00 | 0.01 | 9.83E-01 |
| rs10100265 | 0.00 | 0.01 | 8.90E-01 |
| rs10114341 | 0.00 | 0.01 | 9.77E-01 |
| rs10401969 | 0.00 | 0.01 | 9.19E-01 |
| rs1050226 | 0.00 | 0.01 | 8.46E-01 |
| rs1061813 | 0.00 | 0.01 | 9.55E-01 |
| rs1063355 | 0.00 | 0.01 | 7.69E-01 |
| rs10740322 | 0.00 | 0.01 | 9.23E-01 |
| rs10811661 | 0.00 | 0.01 | 9.32E-01 |
| rs10830963 | 0.00 | 0.01 | 8.60E-01 |
| rs10842994 | 0.00 | 0.01 | 9.44E-01 |
| rs10974438 | 0.00 | 0.01 | 9.06E-01 |
| rs11098676 | 0.00 | 0.01 | 9.69E-01 |
| rs11107116 | 0.00 | 0.01 | 9.19E-01 |
| rs1111875 | 0.00 | 0.01 | 8.88E-01 |
| rs11257655 | 0.00 | 0.01 | 9.45E-01 |
| rs1127655 | 0.00 | 0.01 | 9.08E-01 |
| rs11926707 | 0.00 | 0.01 | 9.98E-01 |
| rs12088739 | 0.00 | 0.01 | 9.81E-01 |
| rs12299509 | 0.00 | 0.01 | 9.28E-01 |
| rs12617659 | 0.00 | 0.01 | 8.43E-01 |
| rs12945601 | 0.00 | 0.01 | 9.72E-01 |
| rs12970134 | 0.00 | 0.01 | 9.69E-01 |
| rs13234269 | 0.00 | 0.01 | 9.73E-01 |
| rs13239186 | 0.00 | 0.01 | 9.62E-01 |
| rs13330951 | 0.00 | 0.01 | 9.61E-01 |
| rs13389219 | 0.00 | 0.01 | 9.45E-01 |
| rs1359790 | 0.00 | 0.01 | 9.17E-01 |
| rs1496653 | 0.00 | 0.01 | 9.50E-01 |
| rs1552224 | 0.00 | 0.01 | 9.60E-01 |
| rs17086692 | 0.00 | 0.01 | 9.78E-01 |
| rs17168486 | 0.00 | 0.01 | 9.09E-01 |
| rs17405722 | 0.00 | 0.01 | 9.73E-01 |
| rs17411031 | 0.00 | 0.01 | 9.18E-01 |
| rs1758632 | 0.00 | 0.01 | 9.73E-01 |
| rs17631783 | 0.00 | 0.01 | 9.60E-01 |
| rs17791513 | 0.00 | 0.01 | 9.78E-01 |
| rs1801214 | 0.00 | 0.01 | 9.86E-01 |
| rs1899951 | 0.00 | 0.01 | 1.00E+00 |
| rs2058913 | 0.00 | 0.01 | 9.76E-01 |
| rs2237892 | 0.00 | 0.01 | 8.62E-01 |
| rs2246618 | 0.00 | 0.01 | 9.73E-01 |
| rs2261181 | 0.00 | 0.01 | 9.86E-01 |
| rs2294120 | 0.00 | 0.01 | 9.42E-01 |
| rs2296173 | 0.00 | 0.01 | 9.95E-01 |
| rs2299383 | 0.00 | 0.01 | 9.95E-01 |
| rs243019 | 0.00 | 0.01 | 9.50E-01 |
| rs2493394 | 0.00 | 0.01 | 9.97E-01 |
| rs2796441 | 0.00 | 0.01 | 9.34E-01 |
| rs2820426 | 0.00 | 0.01 | 9.76E-01 |
| rs2867125 | 0.00 | 0.01 | 9.81E-01 |
| rs2908282 | 0.00 | 0.01 | 9.16E-01 |
| rs2925979 | 0.00 | 0.01 | 9.55E-01 |
| rs2943656 | 0.00 | 0.01 | 9.54E-01 |
| rs3217992 | 0.00 | 0.01 | 9.61E-01 |
| rs340874 | 0.00 | 0.01 | 9.96E-01 |
| rs348330 | 0.00 | 0.01 | 9.31E-01 |
| rs3756784 | 0.00 | 0.01 | 9.52E-01 |
| rs3802177 | 0.00 | 0.01 | 9.74E-01 |
| rs459193 | 0.00 | 0.01 | 8.36E-01 |
| rs4622883 | 0.00 | 0.01 | 9.63E-01 |
| rs4812829 | 0.00 | 0.01 | 9.25E-01 |
| rs4823182 | 0.00 | 0.01 | 9.77E-01 |
| rs4865796 | 0.00 | 0.01 | 9.33E-01 |
| rs516946 | 0.00 | 0.01 | 9.51E-01 |
| rs5215 | 0.00 | 0.01 | 9.92E-01 |
| rs576674 | 0.00 | 0.01 | 9.81E-01 |
| rs6059662 | 0.00 | 0.01 | 9.67E-01 |
| rs622217 | 0.00 | 0.01 | 9.42E-01 |
| rs6494307 | 0.00 | 0.01 | 9.56E-01 |
| rs6515236 | 0.00 | 0.01 | 9.17E-01 |
| rs6767484 | 0.00 | 0.01 | 7.21E-01 |
| rs6785040 | 0.00 | 0.01 | 8.97E-01 |
| rs6795735 | 0.00 | 0.01 | 9.33E-01 |
| rs6878122 | 0.00 | 0.01 | 9.56E-01 |
| rs6960043 | 0.00 | 0.01 | 9.50E-01 |
| rs7177055 | 0.00 | 0.01 | 9.31E-01 |
| rs7240767 | 0.00 | 0.01 | 9.80E-01 |
| rs72802358 | 0.00 | 0.01 | 9.91E-01 |
| rs72892910 | 0.00 | 0.01 | 9.92E-01 |
| rs753270 | 0.00 | 0.01 | 9.29E-01 |
| rs7561798 | 0.00 | 0.01 | 9.63E-01 |
| rs7572970 | 0.00 | 0.01 | 9.61E-01 |
| rs7607777 | 0.00 | 0.01 | 9.73E-01 |
| rs7674212 | 0.00 | 0.01 | 9.08E-01 |
| rs7685296 | 0.00 | 0.01 | 9.83E-01 |
| rs7756992 | 0.00 | 0.01 | 7.72E-01 |
| rs7786095 | 0.00 | 0.01 | 9.52E-01 |
| rs780094 | 0.00 | 0.01 | 7.25E-01 |
| rs7845219 | 0.00 | 0.01 | 9.96E-01 |
| rs7903146 | 0.00 | 0.01 | 9.04E-01 |
| rs7929543 | 0.00 | 0.01 | 9.07E-01 |
| rs7955901 | 0.00 | 0.01 | 9.85E-01 |
| rs8068804 | 0.00 | 0.01 | 8.56E-01 |
| rs8108269 | 0.00 | 0.01 | 7.42E-01 |
| rs825476 | 0.00 | 0.01 | 9.96E-01 |
| rs840967 | 0.00 | 0.01 | 9.75E-01 |
| rs853974 | 0.00 | 0.01 | 9.35E-01 |
| rs9369425 | 0.00 | 0.01 | 8.83E-01 |
| rs963740 | 0.00 | 0.01 | 9.85E-01 |
| rs9894220 | 0.00 | 0.01 | 9.69E-01 |
| rs9928094 | 0.00 | 0.01 | 9.25E-01 |
| rs993380 | 0.00 | 0.01 | 9.94E-01 |
| rs9940149 | 0.00 | 0.01 | 9.45E-01 |
| All | 0.00 | 0.01 | 9.78E-01 |

T2DM: Type-2 diabetes; LDL: Low-density lipoprotein; Se: Standard error.

Table 51: Single SNP analysis for estimate of T2DM-LDL(Kettunen).

| SNP | Effect | Se | P-value |
| --- | --- | --- | --- |
| rs10077431 | 0.55 | 0.27 | 4.00E-02 |
| rs10087241 | 0.23 | 0.22 | 2.78E-01 |
| rs10100265 | -0.22 | 0.20 | 2.79E-01 |
| rs10114341 | 0.07 | 0.24 | 7.68E-01 |
| rs10401969 | -0.92 | 0.22 | 2.22E-05 |
| rs1050226 | 0.06 | 0.20 | 7.61E-01 |
| rs1061813 | 0.19 | 0.24 | 4.35E-01 |
| rs1063355 | -0.28 | 0.15 | 5.76E-02 |
| rs10740322 | 0.05 | 0.23 | 8.14E-01 |
| rs10811661 | -0.03 | 0.09 | 7.67E-01 |
| rs10830963 | 0.01 | 0.12 | 9.62E-01 |
| rs10842994 | 0.09 | 0.17 | 6.00E-01 |
| rs10974438 | 0.26 | 0.17 | 1.29E-01 |
| rs11098676 | -0.05 | 0.23 | 8.36E-01 |
| rs11107116 | 0.25 | 0.24 | 3.10E-01 |
| rs1111875 | 0.17 | 0.11 | 9.92E-02 |
| rs11257655 | 0.12 | 0.16 | 4.44E-01 |
| rs1127655 | 0.25 | 0.22 | 2.56E-01 |
| rs11708067 | 0.07 | 0.13 | 5.76E-01 |
| rs11925227 | -0.15 | 0.25 | 5.57E-01 |
| rs11926707 | -0.06 | 0.23 | 7.97E-01 |
| rs12088739 | -0.07 | 0.23 | 7.56E-01 |
| rs12299509 | 0.16 | 0.23 | 4.80E-01 |
| rs12617659 | -0.17 | 0.20 | 4.05E-01 |
| rs12910825 | -0.28 | 0.20 | 1.55E-01 |
| rs12945601 | -0.16 | 0.21 | 4.45E-01 |
| rs12970134 | 0.40 | 0.22 | 6.31E-02 |
| rs13234269 | 0.15 | 0.17 | 3.67E-01 |
| rs13239186 | -0.23 | 0.20 | 2.41E-01 |
| rs13330951 | -0.07 | 0.22 | 7.47E-01 |
| rs13389219 | 0.18 | 0.14 | 2.12E-01 |
| rs1359790 | -0.24 | 0.14 | 8.61E-02 |
| rs1496653 | -0.04 | 0.14 | 7.63E-01 |
| rs1552224 | 0.12 | 0.11 | 2.90E-01 |
| rs16988333 | -0.10 | 0.23 | 6.55E-01 |
| rs17086692 | -0.13 | 0.22 | 5.63E-01 |
| rs17168486 | 0.12 | 0.16 | 4.49E-01 |
| rs17405722 | 0.14 | 0.20 | 4.67E-01 |
| rs17411031 | 0.36 | 0.25 | 1.42E-01 |
| rs1758632 | -0.23 | 0.20 | 2.64E-01 |
| rs17631783 | 0.10 | 0.24 | 6.91E-01 |
| rs17791513 | 0.04 | 0.16 | 8.01E-01 |
| rs1801214 | 0.17 | 0.11 | 1.20E-01 |
| rs1899951 | 0.10 | 0.12 | 3.81E-01 |
| rs2058913 | 0.00 | 0.20 | 9.98E-01 |
| rs2237892 | 0.08 | 0.23 | 7.36E-01 |
| rs2246618 | 0.26 | 0.21 | 2.17E-01 |
| rs2261181 | -0.04 | 0.19 | 8.41E-01 |
| rs2294120 | 0.18 | 0.22 | 4.15E-01 |
| rs2296173 | 0.20 | 0.19 | 2.81E-01 |
| rs2299383 | 0.09 | 0.24 | 7.18E-01 |
| rs243019 | -0.07 | 0.17 | 6.94E-01 |
| rs2493394 | 0.37 | 0.20 | 6.63E-02 |
| rs2796441 | -0.17 | 0.14 | 2.31E-01 |
| rs2820426 | 0.32 | 0.20 | 1.01E-01 |
| rs2867125 | 0.09 | 0.22 | 6.95E-01 |
| rs2908282 | -0.38 | 0.27 | 1.61E-01 |
| rs2925979 | -0.08 | 0.20 | 6.84E-01 |
| rs2943656 | 0.03 | 0.11 | 7.99E-01 |
| rs3217992 | 0.00 | 0.19 | 1.00E+00 |
| rs340874 | 0.13 | 0.16 | 3.96E-01 |
| rs348330 | 0.07 | 0.22 | 7.38E-01 |
| rs3756784 | -0.33 | 0.24 | 1.79E-01 |
| rs3802177 | 0.03 | 0.08 | 7.26E-01 |
| rs459193 | -0.14 | 0.15 | 3.39E-01 |
| rs4622883 | -0.12 | 0.23 | 5.95E-01 |
| rs4686471 | -0.24 | 0.19 | 1.94E-01 |
| rs4812829 | -0.10 | 0.23 | 6.58E-01 |
| rs4823182 | 0.05 | 0.21 | 8.26E-01 |
| rs4865796 | 0.12 | 0.20 | 5.34E-01 |
| rs516946 | 0.05 | 0.14 | 7.15E-01 |
| rs5215 | 0.14 | 0.15 | 3.30E-01 |
| rs55966194 | 0.09 | 0.23 | 6.79E-01 |
| rs576674 | -0.17 | 0.24 | 4.77E-01 |
| rs6059662 | 0.02 | 0.26 | 9.28E-01 |
| rs61953351 | 0.34 | 0.16 | 3.33E-02 |
| rs622217 | 0.27 | 0.20 | 1.87E-01 |
| rs6494307 | -0.16 | 0.22 | 4.68E-01 |
| rs6515236 | -0.04 | 0.24 | 8.77E-01 |
| rs67232546 | 0.39 | 0.23 | 8.75E-02 |
| rs6767484 | -0.03 | 0.09 | 6.96E-01 |
| rs6785040 | 0.07 | 0.19 | 7.06E-01 |
| rs6795735 | -0.01 | 0.18 | 9.51E-01 |
| rs6878122 | 0.38 | 0.21 | 6.65E-02 |
| rs6960043 | -0.09 | 0.15 | 5.56E-01 |
| rs7144011 | 0.04 | 0.24 | 8.60E-01 |
| rs7177055 | 0.06 | 0.17 | 7.38E-01 |
| rs7240767 | 0.28 | 0.23 | 2.09E-01 |
| rs72802358 | -0.07 | 0.15 | 6.48E-01 |
| rs72892910 | 0.24 | 0.19 | 2.04E-01 |
| rs735949 | -0.15 | 0.22 | 5.03E-01 |
| rs753270 | 0.32 | 0.19 | 9.11E-02 |
| rs7561798 | 0.01 | 0.25 | 9.83E-01 |
| rs7572970 | -0.03 | 0.20 | 8.77E-01 |
| rs7607777 | 0.38 | 0.15 | 1.35E-02 |
| rs7674212 | 0.24 | 0.21 | 2.65E-01 |
| rs7685296 | 0.06 | 0.22 | 7.72E-01 |
| rs7729395 | -0.17 | 0.18 | 3.62E-01 |
| rs7756992 | -0.11 | 0.08 | 1.57E-01 |
| rs7786095 | 0.35 | 0.26 | 1.83E-01 |
| rs780094 | -0.52 | 0.15 | 4.09E-04 |
| rs7845219 | -0.12 | 0.23 | 6.13E-01 |
| rs7903146 | 0.00 | 0.04 | 9.15E-01 |
| rs7929543 | -0.02 | 0.23 | 9.18E-01 |
| rs7955901 | -0.21 | 0.23 | 3.58E-01 |
| rs8068804 | -0.05 | 0.18 | 7.94E-01 |
| rs8108269 | 0.26 | 0.17 | 1.20E-01 |
| rs825476 | 0.39 | 0.19 | 3.97E-02 |
| rs840967 | 0.09 | 0.20 | 6.60E-01 |
| rs849135 | -0.03 | 0.10 | 7.35E-01 |
| rs853974 | -0.26 | 0.19 | 1.62E-01 |
| rs9369425 | -0.20 | 0.20 | 3.38E-01 |
| rs963740 | 0.27 | 0.24 | 2.61E-01 |
| rs9844972 | -0.05 | 0.23 | 8.15E-01 |
| rs9894220 | 0.06 | 0.17 | 7.20E-01 |
| rs9928094 | 0.20 | 0.10 | 3.36E-02 |
| rs993380 | 0.08 | 0.20 | 6.68E-01 |
| rs9940149 | -0.43 | 0.27 | 1.04E-01 |
| All - Inverse variance weighted | 0.03 | 0.02 | 1.24E-01 |
| All - MR Egger | -0.01 | 0.04 | 8.58E-01 |

T2DM: Type-2 diabetes; LDL: Low-density lipoprotein; Se: Standard error.

Table 52: Leave-one-out analysis for estimate of T2DM-LDL(Kettunen).

| SNP | Effect | Se | P-value |
| --- | --- | --- | --- |
| rs10077431 | 0.02 | 0.02 | 1.47E-01 |
| rs10087241 | 0.02 | 0.02 | 1.41E-01 |
| rs10100265 | 0.03 | 0.02 | 1.07E-01 |
| rs10114341 | 0.02 | 0.02 | 1.29E-01 |
| rs10401969 | 0.03 | 0.02 | 5.50E-02 |
| rs1050226 | 0.02 | 0.02 | 1.30E-01 |
| rs1061813 | 0.02 | 0.02 | 1.36E-01 |
| rs1063355 | 0.03 | 0.02 | 8.28E-02 |
| rs10740322 | 0.03 | 0.02 | 1.29E-01 |
| rs10811661 | 0.03 | 0.02 | 1.10E-01 |
| rs10830963 | 0.03 | 0.02 | 1.24E-01 |
| rs10842994 | 0.02 | 0.02 | 1.34E-01 |
| rs10974438 | 0.02 | 0.02 | 1.53E-01 |
| rs11098676 | 0.03 | 0.02 | 1.22E-01 |
| rs11107116 | 0.02 | 0.02 | 1.38E-01 |
| rs1111875 | 0.02 | 0.02 | 1.78E-01 |
| rs11257655 | 0.02 | 0.02 | 1.40E-01 |
| rs1127655 | 0.02 | 0.02 | 1.41E-01 |
| rs11708067 | 0.02 | 0.02 | 1.38E-01 |
| rs11925227 | 0.03 | 0.02 | 1.17E-01 |
| rs11926707 | 0.03 | 0.02 | 1.22E-01 |
| rs12088739 | 0.03 | 0.02 | 1.21E-01 |
| rs12299509 | 0.02 | 0.02 | 1.35E-01 |
| rs12617659 | 0.03 | 0.02 | 1.11E-01 |
| rs12910825 | 0.03 | 0.02 | 1.00E-01 |
| rs12945601 | 0.03 | 0.02 | 1.13E-01 |
| rs12970134 | 0.02 | 0.02 | 1.51E-01 |
| rs13234269 | 0.02 | 0.02 | 1.42E-01 |
| rs13239186 | 0.03 | 0.02 | 1.05E-01 |
| rs13330951 | 0.03 | 0.02 | 1.20E-01 |
| rs13389219 | 0.02 | 0.02 | 1.53E-01 |
| rs1359790 | 0.03 | 0.02 | 8.48E-02 |
| rs1496653 | 0.03 | 0.02 | 1.17E-01 |
| rs1552224 | 0.02 | 0.02 | 1.55E-01 |
| rs16988333 | 0.03 | 0.02 | 1.19E-01 |
| rs17086692 | 0.03 | 0.02 | 1.16E-01 |
| rs17168486 | 0.02 | 0.02 | 1.40E-01 |
| rs17405722 | 0.02 | 0.02 | 1.37E-01 |
| rs17411031 | 0.02 | 0.02 | 1.43E-01 |
| rs1758632 | 0.03 | 0.02 | 1.06E-01 |
| rs17631783 | 0.02 | 0.02 | 1.31E-01 |
| rs17791513 | 0.03 | 0.02 | 1.30E-01 |
| rs1801214 | 0.02 | 0.02 | 1.72E-01 |
| rs1899951 | 0.02 | 0.02 | 1.48E-01 |
| rs2058913 | 0.03 | 0.02 | 1.25E-01 |
| rs2237892 | 0.02 | 0.02 | 1.30E-01 |
| rs2246618 | 0.02 | 0.02 | 1.43E-01 |
| rs2261181 | 0.03 | 0.02 | 1.21E-01 |
| rs2294120 | 0.02 | 0.02 | 1.37E-01 |
| rs2296173 | 0.02 | 0.02 | 1.43E-01 |
| rs2299383 | 0.02 | 0.02 | 1.30E-01 |
| rs243019 | 0.03 | 0.02 | 1.17E-01 |
| rs2493394 | 0.02 | 0.02 | 1.53E-01 |
| rs2796441 | 0.03 | 0.02 | 9.70E-02 |
| rs2820426 | 0.02 | 0.02 | 1.51E-01 |
| rs2867125 | 0.02 | 0.02 | 1.31E-01 |
| rs2908282 | 0.03 | 0.02 | 1.07E-01 |
| rs2925979 | 0.03 | 0.02 | 1.18E-01 |
| rs2943656 | 0.03 | 0.02 | 1.30E-01 |
| rs3217992 | 0.03 | 0.02 | 1.25E-01 |
| rs340874 | 0.02 | 0.02 | 1.42E-01 |
| rs348330 | 0.02 | 0.02 | 1.30E-01 |
| rs3756784 | 0.03 | 0.02 | 1.06E-01 |
| rs3802177 | 0.03 | 0.02 | 1.34E-01 |
| rs459193 | 0.03 | 0.02 | 1.03E-01 |
| rs4622883 | 0.03 | 0.02 | 1.17E-01 |
| rs4686471 | 0.03 | 0.02 | 1.02E-01 |
| rs4812829 | 0.03 | 0.02 | 1.19E-01 |
| rs4823182 | 0.03 | 0.02 | 1.29E-01 |
| rs4865796 | 0.02 | 0.02 | 1.35E-01 |
| rs516946 | 0.02 | 0.02 | 1.32E-01 |
| rs5215 | 0.02 | 0.02 | 1.46E-01 |
| rs55966194 | 0.02 | 0.02 | 1.31E-01 |
| rs576674 | 0.03 | 0.02 | 1.15E-01 |
| rs6059662 | 0.03 | 0.02 | 1.27E-01 |
| rs61953351 | 0.02 | 0.02 | 1.67E-01 |
| rs622217 | 0.02 | 0.02 | 1.45E-01 |
| rs6494307 | 0.03 | 0.02 | 1.14E-01 |
| rs6515236 | 0.03 | 0.02 | 1.23E-01 |
| rs67232546 | 0.02 | 0.02 | 1.48E-01 |
| rs6767484 | 0.03 | 0.02 | 1.07E-01 |
| rs6785040 | 0.02 | 0.02 | 1.31E-01 |
| rs6795735 | 0.03 | 0.02 | 1.24E-01 |
| rs6878122 | 0.02 | 0.02 | 1.52E-01 |
| rs6960043 | 0.03 | 0.02 | 1.12E-01 |
| rs7144011 | 0.03 | 0.02 | 1.28E-01 |
| rs7177055 | 0.02 | 0.02 | 1.31E-01 |
| rs7240767 | 0.02 | 0.02 | 1.42E-01 |
| rs72802358 | 0.03 | 0.02 | 1.14E-01 |
| rs72892910 | 0.02 | 0.02 | 1.46E-01 |
| rs735949 | 0.03 | 0.02 | 1.15E-01 |
| rs753270 | 0.02 | 0.02 | 1.53E-01 |
| rs7561798 | 0.03 | 0.02 | 1.26E-01 |
| rs7572970 | 0.03 | 0.02 | 1.22E-01 |
| rs7607777 | 0.02 | 0.02 | 1.77E-01 |
| rs7674212 | 0.02 | 0.02 | 1.42E-01 |
| rs7685296 | 0.02 | 0.02 | 1.29E-01 |
| rs7729395 | 0.03 | 0.02 | 1.08E-01 |
| rs7756992 | 0.03 | 0.02 | 6.96E-02 |
| rs7786095 | 0.02 | 0.02 | 1.41E-01 |
| rs780094 | 0.03 | 0.02 | 5.11E-02 |
| rs7845219 | 0.03 | 0.02 | 1.18E-01 |
| rs7903146 | 0.03 | 0.02 | 1.08E-01 |
| rs7929543 | 0.03 | 0.02 | 1.24E-01 |
| rs7955901 | 0.03 | 0.02 | 1.11E-01 |
| rs8068804 | 0.03 | 0.02 | 1.20E-01 |
| rs8108269 | 0.02 | 0.02 | 1.54E-01 |
| rs825476 | 0.02 | 0.02 | 1.59E-01 |
| rs840967 | 0.02 | 0.02 | 1.32E-01 |
| rs849135 | 0.03 | 0.02 | 1.11E-01 |
| rs853974 | 0.03 | 0.02 | 9.97E-02 |
| rs9369425 | 0.03 | 0.02 | 1.09E-01 |
| rs963740 | 0.02 | 0.02 | 1.40E-01 |
| rs9844972 | 0.03 | 0.02 | 1.22E-01 |
| rs9894220 | 0.02 | 0.02 | 1.31E-01 |
| rs9928094 | 0.02 | 0.02 | 2.05E-01 |
| rs993380 | 0.02 | 0.02 | 1.32E-01 |
| rs9940149 | 0.03 | 0.02 | 1.03E-01 |
| All | 0.03 | 0.02 | 1.24E-01 |

T2DM: Type-2 diabetes; LDL: Low-density lipoprotein; Se: Standard error.

Table 53: Single SNP analysis for estimate of T2DM-TC(Willer).

| SNP | Effect | Se | P-value |
| --- | --- | --- | --- |
| rs10077431 | -0.11 | 0.09 | 2.30E-01 |
| rs10100265 | 0.05 | 0.07 | 4.53E-01 |
| rs10114341 | 0.07 | 0.09 | 4.24E-01 |
| rs10401969 | -1.49 | 0.08 | 3.59E-85 |
| rs1050226 | -0.06 | 0.07 | 4.20E-01 |
| rs1061813 | 0.07 | 0.09 | 3.87E-01 |
| rs1063355 | -0.25 | 0.08 | 1.69E-03 |
| rs10740322 | 0.07 | 0.12 | 5.63E-01 |
| rs10811661 | 0.01 | 0.03 | 7.98E-01 |
| rs10830963 | 0.09 | 0.05 | 4.82E-02 |
| rs10842994 | 0.04 | 0.06 | 5.34E-01 |
| rs10974438 | 0.09 | 0.06 | 1.52E-01 |
| rs11098676 | -0.02 | 0.11 | 8.70E-01 |
| rs11107116 | -0.27 | 0.09 | 2.50E-03 |
| rs1111875 | 0.09 | 0.04 | 1.40E-02 |
| rs11257655 | 0.06 | 0.06 | 2.85E-01 |
| rs1127655 | 0.07 | 0.12 | 5.30E-01 |
| rs11708067 | 0.21 | 0.05 | 6.80E-06 |
| rs11925227 | 0.19 | 0.09 | 3.52E-02 |
| rs11926707 | 0.17 | 0.12 | 1.56E-01 |
| rs12088739 | 0.15 | 0.07 | 3.33E-02 |
| rs12299509 | 0.08 | 0.08 | 3.05E-01 |
| rs12617659 | -0.08 | 0.07 | 2.80E-01 |
| rs12910825 | -0.06 | 0.07 | 4.17E-01 |
| rs12945601 | 0.07 | 0.11 | 5.21E-01 |
| rs12970134 | -0.09 | 0.08 | 2.25E-01 |
| rs13234269 | 0.10 | 0.09 | 2.41E-01 |
| rs13239186 | 0.12 | 0.10 | 2.06E-01 |
| rs13330951 | -0.02 | 0.11 | 8.60E-01 |
| rs13389219 | 0.28 | 0.05 | 3.24E-08 |
| rs1359790 | -0.02 | 0.05 | 6.89E-01 |
| rs1496653 | 0.02 | 0.05 | 7.21E-01 |
| rs1552224 | -0.04 | 0.05 | 3.95E-01 |
| rs16988333 | 0.09 | 0.09 | 2.95E-01 |
| rs17086692 | -0.04 | 0.12 | 7.34E-01 |
| rs17168486 | -0.01 | 0.06 | 8.45E-01 |
| rs17405722 | -0.02 | 0.11 | 8.68E-01 |
| rs17411031 | 0.04 | 0.09 | 6.17E-01 |
| rs1758632 | 0.04 | 0.11 | 7.01E-01 |
| rs17631783 | 0.11 | 0.12 | 3.61E-01 |
| rs17791513 | 0.04 | 0.07 | 5.76E-01 |
| rs1801214 | -0.01 | 0.04 | 7.18E-01 |
| rs1899951 | 0.02 | 0.05 | 6.31E-01 |
| rs2058913 | -0.03 | 0.11 | 7.58E-01 |
| rs2237892 | 0.05 | 0.08 | 5.52E-01 |
| rs2246618 | -0.14 | 0.08 | 8.78E-02 |
| rs2261181 | 0.07 | 0.06 | 2.31E-01 |
| rs2294120 | 0.03 | 0.11 | 7.82E-01 |
| rs2296173 | -0.02 | 0.07 | 7.98E-01 |
| rs2299383 | -0.06 | 0.09 | 5.05E-01 |
| rs243019 | 0.01 | 0.06 | 8.24E-01 |
| rs2493394 | 0.10 | 0.08 | 1.86E-01 |
| rs2796441 | 0.01 | 0.05 | 8.93E-01 |
| rs2820426 | 0.17 | 0.07 | 1.15E-02 |
| rs2867125 | -0.13 | 0.08 | 8.59E-02 |
| rs2908282 | -0.01 | 0.09 | 9.17E-01 |
| rs2925979 | -0.12 | 0.07 | 1.01E-01 |
| rs2943656 | -0.01 | 0.04 | 8.90E-01 |
| rs3217992 | -0.03 | 0.07 | 6.27E-01 |
| rs340874 | 0.16 | 0.06 | 4.61E-03 |
| rs3756784 | -0.23 | 0.09 | 1.03E-02 |
| rs3802177 | 0.08 | 0.03 | 1.71E-02 |
| rs459193 | -0.16 | 0.06 | 4.73E-03 |
| rs4622883 | -0.22 | 0.12 | 6.21E-02 |
| rs4812829 | 0.00 | 0.09 | 9.83E-01 |
| rs4823182 | -0.34 | 0.08 | 1.20E-05 |
| rs4865796 | 0.05 | 0.07 | 5.11E-01 |
| rs516946 | 0.01 | 0.05 | 8.07E-01 |
| rs5215 | 0.15 | 0.05 | 4.22E-03 |
| rs55966194 | 0.29 | 0.11 | 1.24E-02 |
| rs576674 | 0.03 | 0.08 | 6.89E-01 |
| rs6059662 | -0.22 | 0.09 | 8.50E-03 |
| rs622217 | 0.42 | 0.10 | 1.29E-05 |
| rs6494307 | -0.10 | 0.12 | 4.08E-01 |
| rs6515236 | -0.25 | 0.12 | 4.21E-02 |
| rs6767484 | -0.04 | 0.03 | 1.63E-01 |
| rs6785040 | -0.05 | 0.12 | 6.79E-01 |
| rs6795735 | 0.09 | 0.06 | 1.49E-01 |
| rs6878122 | 0.05 | 0.07 | 5.10E-01 |
| rs6960043 | 0.02 | 0.06 | 6.77E-01 |
| rs7144011 | -0.08 | 0.09 | 3.77E-01 |
| rs7177055 | 0.04 | 0.06 | 4.57E-01 |
| rs7240767 | 0.07 | 0.12 | 5.53E-01 |
| rs72802358 | 0.01 | 0.05 | 8.50E-01 |
| rs72892910 | 0.01 | 0.10 | 9.01E-01 |
| rs735949 | -0.09 | 0.07 | 2.02E-01 |
| rs753270 | 0.11 | 0.10 | 2.91E-01 |
| rs7561798 | -0.02 | 0.09 | 8.19E-01 |
| rs7572970 | 0.00 | 0.10 | 9.86E-01 |
| rs7607777 | 0.08 | 0.05 | 8.44E-02 |
| rs7685296 | -0.03 | 0.08 | 7.45E-01 |
| rs7729395 | 0.05 | 0.07 | 4.98E-01 |
| rs7756992 | 0.00 | 0.03 | 8.98E-01 |
| rs7786095 | 0.13 | 0.11 | 2.38E-01 |
| rs780094 | -0.73 | 0.05 | 1.56E-44 |
| rs7845219 | -0.02 | 0.08 | 8.41E-01 |
| rs7903146 | 0.03 | 0.01 | 2.40E-02 |
| rs7929543 | 0.01 | 0.09 | 8.77E-01 |
| rs7955901 | 0.00 | 0.08 | 9.77E-01 |
| rs8068804 | 0.11 | 0.07 | 1.01E-01 |
| rs8108269 | -0.30 | 0.06 | 2.83E-06 |
| rs825476 | -0.15 | 0.07 | 2.78E-02 |
| rs840967 | 0.19 | 0.10 | 7.37E-02 |
| rs849135 | -0.01 | 0.04 | 7.75E-01 |
| rs9369425 | -0.20 | 0.10 | 5.60E-02 |
| rs963740 | -0.11 | 0.12 | 3.71E-01 |
| rs9844972 | 0.12 | 0.10 | 2.45E-01 |
| rs9894220 | -0.06 | 0.09 | 5.29E-01 |
| rs9928094 | -0.05 | 0.03 | 1.41E-01 |
| rs993380 | 0.00 | 0.10 | 9.85E-01 |
| rs9940149 | -0.01 | 0.08 | 9.50E-01 |
| All - Inverse variance weighted | 0.00 | 0.02 | 9.69E-01 |
| All - MR Egger | 0.03 | 0.03 | 3.67E-01 |

T2DM: Type-2 diabetes; TC: Total cholesterol; Se: Standard error.

Table 54: Leave-one-out analysis for estimate of T2DM-TC(Willer).

| SNP | Effect | Se | P-value |
| --- | --- | --- | --- |
| rs10077431 | 0.00 | 0.02 | 9.49E-01 |
| rs10100265 | 0.00 | 0.02 | 9.85E-01 |
| rs10114341 | 0.00 | 0.02 | 9.84E-01 |
| rs10401969 | 0.01 | 0.01 | 4.66E-01 |
| rs1050226 | 0.00 | 0.02 | 9.52E-01 |
| rs1061813 | 0.00 | 0.02 | 9.85E-01 |
| rs1063355 | 0.00 | 0.02 | 9.08E-01 |
| rs10740322 | 0.00 | 0.02 | 9.76E-01 |
| rs10811661 | 0.00 | 0.02 | 9.82E-01 |
| rs10830963 | 0.00 | 0.02 | 9.62E-01 |
| rs10842994 | 0.00 | 0.02 | 9.85E-01 |
| rs10974438 | 0.00 | 0.02 | 9.95E-01 |
| rs11098676 | 0.00 | 0.02 | 9.67E-01 |
| rs11107116 | 0.00 | 0.02 | 9.16E-01 |
| rs1111875 | 0.00 | 0.02 | 9.26E-01 |
| rs11257655 | 0.00 | 0.02 | 9.98E-01 |
| rs1127655 | 0.00 | 0.02 | 9.77E-01 |
| rs11708067 | 0.00 | 0.02 | 8.75E-01 |
| rs11925227 | 0.00 | 0.02 | 9.94E-01 |
| rs11926707 | 0.00 | 0.02 | 9.88E-01 |
| rs12088739 | 0.00 | 0.02 | 9.84E-01 |
| rs12299509 | 0.00 | 0.02 | 9.89E-01 |
| rs12617659 | 0.00 | 0.02 | 9.46E-01 |
| rs12910825 | 0.00 | 0.02 | 9.51E-01 |
| rs12945601 | 0.00 | 0.02 | 9.78E-01 |
| rs12970134 | 0.00 | 0.02 | 9.44E-01 |
| rs13234269 | 0.00 | 0.02 | 9.89E-01 |
| rs13239186 | 0.00 | 0.02 | 9.89E-01 |
| rs13330951 | 0.00 | 0.02 | 9.66E-01 |
| rs13389219 | 0.00 | 0.02 | 8.56E-01 |
| rs1359790 | 0.00 | 0.02 | 9.56E-01 |
| rs1496653 | 0.00 | 0.02 | 9.79E-01 |
| rs1552224 | 0.00 | 0.02 | 9.39E-01 |
| rs16988333 | 0.00 | 0.02 | 9.88E-01 |
| rs17086692 | 0.00 | 0.02 | 9.65E-01 |
| rs17168486 | 0.00 | 0.02 | 9.64E-01 |
| rs17405722 | 0.00 | 0.02 | 9.67E-01 |
| rs17411031 | 0.00 | 0.02 | 9.78E-01 |
| rs1758632 | 0.00 | 0.02 | 9.75E-01 |
| rs17631783 | 0.00 | 0.02 | 9.81E-01 |
| rs17791513 | 0.00 | 0.02 | 9.82E-01 |
| rs1801214 | 0.00 | 0.02 | 9.54E-01 |
| rs1899951 | 0.00 | 0.02 | 9.85E-01 |
| rs2058913 | 0.00 | 0.02 | 9.64E-01 |
| rs2237892 | 0.00 | 0.02 | 9.80E-01 |
| rs2246618 | 0.00 | 0.02 | 9.35E-01 |
| rs2261181 | 0.00 | 0.02 | 9.99E-01 |
| rs2294120 | 0.00 | 0.02 | 9.73E-01 |
| rs2296173 | 0.00 | 0.02 | 9.63E-01 |
| rs2299383 | 0.00 | 0.02 | 9.57E-01 |
| rs243019 | 0.00 | 0.02 | 9.74E-01 |
| rs2493394 | 0.00 | 0.02 | 9.96E-01 |
| rs2796441 | 0.00 | 0.02 | 9.73E-01 |
| rs2820426 | 0.00 | 0.02 | 9.74E-01 |
| rs2867125 | 0.00 | 0.02 | 9.34E-01 |
| rs2908282 | 0.00 | 0.02 | 9.67E-01 |
| rs2925979 | 0.00 | 0.02 | 9.34E-01 |
| rs2943656 | 0.00 | 0.02 | 9.63E-01 |
| rs3217992 | 0.00 | 0.02 | 9.58E-01 |
| rs340874 | 0.00 | 0.02 | 9.54E-01 |
| rs3756784 | 0.00 | 0.02 | 9.25E-01 |
| rs3802177 | 0.00 | 0.02 | 9.14E-01 |
| rs459193 | 0.00 | 0.02 | 8.90E-01 |
| rs4622883 | 0.00 | 0.02 | 9.45E-01 |
| rs4812829 | 0.00 | 0.02 | 9.69E-01 |
| rs4823182 | 0.00 | 0.02 | 8.79E-01 |
| rs4865796 | 0.00 | 0.02 | 9.83E-01 |
| rs516946 | 0.00 | 0.02 | 9.76E-01 |
| rs5215 | 0.00 | 0.02 | 9.47E-01 |
| rs55966194 | 0.00 | 0.02 | 9.97E-01 |
| rs576674 | 0.00 | 0.02 | 9.77E-01 |
| rs6059662 | 0.00 | 0.02 | 9.20E-01 |
| rs622217 | 0.00 | 0.02 | 9.60E-01 |
| rs6494307 | 0.00 | 0.02 | 9.58E-01 |
| rs6515236 | 0.00 | 0.02 | 9.43E-01 |
| rs6767484 | 0.00 | 0.02 | 8.98E-01 |
| rs6785040 | 0.00 | 0.02 | 9.63E-01 |
| rs6795735 | 0.00 | 0.02 | 9.96E-01 |
| rs6878122 | 0.00 | 0.02 | 9.83E-01 |
| rs6960043 | 0.00 | 0.02 | 9.80E-01 |
| rs7144011 | 0.00 | 0.02 | 9.53E-01 |
| rs7177055 | 0.00 | 0.02 | 9.88E-01 |
| rs7240767 | 0.00 | 0.02 | 9.77E-01 |
| rs72802358 | 0.00 | 0.02 | 9.75E-01 |
| rs72892910 | 0.00 | 0.02 | 9.71E-01 |
| rs735949 | 0.00 | 0.02 | 9.41E-01 |
| rs753270 | 0.00 | 0.02 | 9.85E-01 |
| rs7561798 | 0.00 | 0.02 | 9.65E-01 |
| rs7572970 | 0.00 | 0.02 | 9.69E-01 |
| rs7607777 | 0.00 | 0.02 | 9.71E-01 |
| rs7685296 | 0.00 | 0.02 | 9.62E-01 |
| rs7729395 | 0.00 | 0.02 | 9.85E-01 |
| rs7756992 | 0.00 | 0.02 | 9.75E-01 |
| rs7786095 | 0.00 | 0.02 | 9.85E-01 |
| rs780094 | 0.01 | 0.01 | 5.18E-01 |
| rs7845219 | 0.00 | 0.02 | 9.65E-01 |
| rs7903146 | -0.01 | 0.02 | 7.33E-01 |
| rs7929543 | 0.00 | 0.02 | 9.72E-01 |
| rs7955901 | 0.00 | 0.02 | 9.69E-01 |
| rs8068804 | 0.00 | 0.02 | 9.92E-01 |
| rs8108269 | 0.00 | 0.02 | 8.53E-01 |
| rs825476 | 0.00 | 0.02 | 9.17E-01 |
| rs840967 | 0.00 | 0.02 | 9.96E-01 |
| rs849135 | 0.00 | 0.02 | 9.56E-01 |
| rs9369425 | 0.00 | 0.02 | 9.40E-01 |
| rs963740 | 0.00 | 0.02 | 9.57E-01 |
| rs9844972 | 0.00 | 0.02 | 9.87E-01 |
| rs9894220 | 0.00 | 0.02 | 9.58E-01 |
| rs9928094 | 0.00 | 0.02 | 9.01E-01 |
| rs993380 | 0.00 | 0.02 | 9.69E-01 |
| rs9940149 | 0.00 | 0.02 | 9.68E-01 |
| All | 0.00 | 0.02 | 9.69E-01 |

T2DM: Type-2 diabetes; TC: Total cholesterol; Se: Standard error.

Table 55: Single SNP analysis for estimate of T2DM- TC(Kettunen).

| SNP | Effect | Se | P-value |
| --- | --- | --- | --- |
| rs10077431 | 0.40 | 0.27 | 1.38E-01 |
| rs10087241 | 0.34 | 0.22 | 1.20E-01 |
| rs10100265 | -0.29 | 0.20 | 1.56E-01 |
| rs10114341 | 0.11 | 0.24 | 6.40E-01 |
| rs10401969 | -1.29 | 0.22 | 3.35E-09 |
| rs1050226 | -0.02 | 0.21 | 9.26E-01 |
| rs1061813 | -0.03 | 0.24 | 9.10E-01 |
| rs1063355 | -0.26 | 0.15 | 7.29E-02 |
| rs10740322 | 0.27 | 0.23 | 2.51E-01 |
| rs10811661 | -0.03 | 0.09 | 7.37E-01 |
| rs10830963 | 0.02 | 0.12 | 8.91E-01 |
| rs10842994 | 0.04 | 0.17 | 8.26E-01 |
| rs10974438 | 0.29 | 0.17 | 9.16E-02 |
| rs11098676 | 0.14 | 0.23 | 5.53E-01 |
| rs11107116 | 0.18 | 0.24 | 4.55E-01 |
| rs1111875 | 0.14 | 0.11 | 1.88E-01 |
| rs11257655 | 0.09 | 0.16 | 5.77E-01 |
| rs1127655 | 0.22 | 0.22 | 3.15E-01 |
| rs11708067 | 0.13 | 0.15 | 3.65E-01 |
| rs11925227 | 0.03 | 0.28 | 9.23E-01 |
| rs11926707 | 0.25 | 0.25 | 3.29E-01 |
| rs12088739 | -0.19 | 0.24 | 4.29E-01 |
| rs12299509 | -0.03 | 0.23 | 8.81E-01 |
| rs12617659 | 0.01 | 0.21 | 9.50E-01 |
| rs12910825 | -0.26 | 0.20 | 1.87E-01 |
| rs12945601 | -0.20 | 0.21 | 3.24E-01 |
| rs12970134 | 0.31 | 0.22 | 1.51E-01 |
| rs13234269 | -0.02 | 0.17 | 9.01E-01 |
| rs13239186 | -0.16 | 0.20 | 4.22E-01 |
| rs13330951 | -0.02 | 0.22 | 9.09E-01 |
| rs13389219 | 0.09 | 0.14 | 5.18E-01 |
| rs1359790 | -0.34 | 0.14 | 1.33E-02 |
| rs1496653 | -0.16 | 0.16 | 3.00E-01 |
| rs1552224 | 0.04 | 0.11 | 7.42E-01 |
| rs16988333 | 0.01 | 0.23 | 9.76E-01 |
| rs17086692 | -0.05 | 0.22 | 8.11E-01 |
| rs17168486 | 0.12 | 0.16 | 4.58E-01 |
| rs17405722 | -0.03 | 0.20 | 8.90E-01 |
| rs17411031 | 0.35 | 0.25 | 1.63E-01 |
| rs1758632 | -0.28 | 0.20 | 1.70E-01 |
| rs17631783 | 0.14 | 0.24 | 5.58E-01 |
| rs17791513 | 0.05 | 0.16 | 7.68E-01 |
| rs1801214 | 0.19 | 0.11 | 8.70E-02 |
| rs1899951 | 0.05 | 0.13 | 6.97E-01 |
| rs2058913 | 0.08 | 0.20 | 6.92E-01 |
| rs2237892 | 0.13 | 0.23 | 5.62E-01 |
| rs2246618 | 0.29 | 0.21 | 1.67E-01 |
| rs2261181 | 0.07 | 0.19 | 7.26E-01 |
| rs2294120 | -0.13 | 0.22 | 5.66E-01 |
| rs2296173 | 0.07 | 0.19 | 7.22E-01 |
| rs2299383 | -0.04 | 0.24 | 8.77E-01 |
| rs243019 | -0.31 | 0.17 | 7.60E-02 |
| rs2493394 | 0.34 | 0.20 | 9.71E-02 |
| rs2796441 | -0.13 | 0.14 | 3.62E-01 |
| rs2820426 | 0.26 | 0.20 | 1.77E-01 |
| rs2867125 | 0.07 | 0.22 | 7.68E-01 |
| rs2908282 | -0.13 | 0.27 | 6.42E-01 |
| rs2925979 | -0.04 | 0.20 | 8.55E-01 |
| rs2943656 | -0.03 | 0.11 | 8.23E-01 |
| rs3217992 | -0.12 | 0.19 | 5.31E-01 |
| rs340874 | 0.16 | 0.16 | 3.09E-01 |
| rs348330 | 0.05 | 0.22 | 8.25E-01 |
| rs3756784 | -0.44 | 0.24 | 7.10E-02 |
| rs3802177 | 0.07 | 0.08 | 4.31E-01 |
| rs459193 | -0.27 | 0.15 | 7.61E-02 |
| rs4622883 | -0.03 | 0.25 | 8.98E-01 |
| rs4686471 | -0.17 | 0.21 | 4.20E-01 |
| rs4812829 | -0.01 | 0.23 | 9.60E-01 |
| rs4823182 | 0.05 | 0.21 | 8.24E-01 |
| rs4865796 | 0.12 | 0.20 | 5.39E-01 |
| rs516946 | -0.06 | 0.14 | 6.60E-01 |
| rs5215 | 0.13 | 0.15 | 3.81E-01 |
| rs55966194 | 0.24 | 0.23 | 2.86E-01 |
| rs576674 | 0.00 | 0.24 | 9.91E-01 |
| rs6059662 | -0.30 | 0.26 | 2.44E-01 |
| rs61953351 | 0.37 | 0.16 | 2.14E-02 |
| rs622217 | 0.15 | 0.20 | 4.61E-01 |
| rs6494307 | -0.22 | 0.22 | 3.17E-01 |
| rs6515236 | -0.01 | 0.24 | 9.71E-01 |
| rs67232546 | 0.45 | 0.23 | 4.98E-02 |
| rs6767484 | 0.01 | 0.10 | 9.42E-01 |
| rs6785040 | 0.04 | 0.21 | 8.60E-01 |
| rs6795735 | -0.23 | 0.20 | 2.44E-01 |
| rs6878122 | 0.33 | 0.21 | 1.09E-01 |
| rs6960043 | -0.04 | 0.16 | 8.05E-01 |
| rs7144011 | -0.20 | 0.24 | 4.16E-01 |
| rs7177055 | 0.00 | 0.17 | 9.86E-01 |
| rs7240767 | 0.26 | 0.23 | 2.44E-01 |
| rs72802358 | -0.17 | 0.15 | 2.72E-01 |
| rs72892910 | 0.34 | 0.19 | 7.52E-02 |
| rs735949 | -0.15 | 0.22 | 5.02E-01 |
| rs753270 | 0.25 | 0.19 | 1.84E-01 |
| rs7561798 | -0.07 | 0.25 | 7.79E-01 |
| rs7572970 | 0.29 | 0.20 | 1.54E-01 |
| rs7607777 | 0.36 | 0.15 | 1.83E-02 |
| rs7674212 | 0.12 | 0.21 | 5.87E-01 |
| rs7685296 | -0.17 | 0.22 | 4.36E-01 |
| rs7729395 | -0.08 | 0.18 | 6.61E-01 |
| rs7756992 | -0.05 | 0.08 | 5.36E-01 |
| rs7786095 | 0.04 | 0.26 | 8.68E-01 |
| rs780094 | -0.93 | 0.15 | 2.87E-10 |
| rs7845219 | -0.05 | 0.23 | 8.39E-01 |
| rs7903146 | 0.01 | 0.04 | 8.27E-01 |
| rs7929543 | -0.07 | 0.23 | 7.56E-01 |
| rs7955901 | -0.37 | 0.23 | 1.12E-01 |
| rs8068804 | -0.06 | 0.18 | 7.56E-01 |
| rs8108269 | 0.26 | 0.17 | 1.25E-01 |
| rs825476 | 0.25 | 0.19 | 1.89E-01 |
| rs840967 | 0.09 | 0.20 | 6.57E-01 |
| rs849135 | -0.05 | 0.10 | 6.16E-01 |
| rs853974 | -0.28 | 0.19 | 1.43E-01 |
| rs9369425 | -0.05 | 0.20 | 8.25E-01 |
| rs963740 | -0.10 | 0.24 | 6.88E-01 |
| rs9844972 | -0.04 | 0.25 | 8.82E-01 |
| rs9894220 | -0.09 | 0.17 | 5.89E-01 |
| rs9928094 | 0.19 | 0.10 | 4.92E-02 |
| rs993380 | 0.08 | 0.20 | 6.81E-01 |
| rs9940149 | -0.45 | 0.27 | 9.62E-02 |
| All - Inverse variance weighted | 0.01 | 0.02 | 7.82E-01 |
| All - MR Egger | 0.01 | 0.05 | 8.67E-01 |

T2DM: Type-2 diabetes; TC: Total cholesterol; Se: Standard error.

Table 56: Leave-one-out analysis for estimate of T2DM- TC(Kettunen).

| SNP | Effect | Se | P-value |
| --- | --- | --- | --- |
| rs10077431 | 0.00 | 0.02 | 8.31E-01 |
| rs10087241 | 0.00 | 0.02 | 8.46E-01 |
| rs10100265 | 0.01 | 0.02 | 7.19E-01 |
| rs10114341 | 0.00 | 0.02 | 8.00E-01 |
| rs10401969 | 0.01 | 0.02 | 5.13E-01 |
| rs1050226 | 0.01 | 0.02 | 7.78E-01 |
| rs1061813 | 0.01 | 0.02 | 7.78E-01 |
| rs1063355 | 0.01 | 0.02 | 6.72E-01 |
| rs10740322 | 0.00 | 0.02 | 8.26E-01 |
| rs10811661 | 0.01 | 0.02 | 7.46E-01 |
| rs10830963 | 0.01 | 0.02 | 7.91E-01 |
| rs10842994 | 0.01 | 0.02 | 7.93E-01 |
| rs10974438 | 0.00 | 0.02 | 8.68E-01 |
| rs11098676 | 0.00 | 0.02 | 8.05E-01 |
| rs11107116 | 0.00 | 0.02 | 8.10E-01 |
| rs1111875 | 0.00 | 0.02 | 8.94E-01 |
| rs11257655 | 0.00 | 0.02 | 8.13E-01 |
| rs1127655 | 0.00 | 0.02 | 8.22E-01 |
| rs11708067 | 0.00 | 0.02 | 8.37E-01 |
| rs11925227 | 0.01 | 0.02 | 7.86E-01 |
| rs11926707 | 0.00 | 0.02 | 8.16E-01 |
| rs12088739 | 0.01 | 0.02 | 7.52E-01 |
| rs12299509 | 0.01 | 0.02 | 7.76E-01 |
| rs12617659 | 0.01 | 0.02 | 7.85E-01 |
| rs12910825 | 0.01 | 0.02 | 7.22E-01 |
| rs12945601 | 0.01 | 0.02 | 7.40E-01 |
| rs12970134 | 0.00 | 0.02 | 8.41E-01 |
| rs13234269 | 0.01 | 0.02 | 7.75E-01 |
| rs13239186 | 0.01 | 0.02 | 7.46E-01 |
| rs13330951 | 0.01 | 0.02 | 7.78E-01 |
| rs13389219 | 0.00 | 0.02 | 8.22E-01 |
| rs1359790 | 0.01 | 0.02 | 6.22E-01 |
| rs1496653 | 0.01 | 0.02 | 7.23E-01 |
| rs1552224 | 0.00 | 0.02 | 8.07E-01 |
| rs16988333 | 0.01 | 0.02 | 7.83E-01 |
| rs17086692 | 0.01 | 0.02 | 7.73E-01 |
| rs17168486 | 0.00 | 0.02 | 8.22E-01 |
| rs17405722 | 0.01 | 0.02 | 7.76E-01 |
| rs17411031 | 0.00 | 0.02 | 8.32E-01 |
| rs1758632 | 0.01 | 0.02 | 7.21E-01 |
| rs17631783 | 0.00 | 0.02 | 8.04E-01 |
| rs17791513 | 0.00 | 0.02 | 7.99E-01 |
| rs1801214 | 0.00 | 0.02 | 9.22E-01 |
| rs1899951 | 0.00 | 0.02 | 8.08E-01 |
| rs2058913 | 0.00 | 0.02 | 8.00E-01 |
| rs2237892 | 0.00 | 0.02 | 8.05E-01 |
| rs2246618 | 0.00 | 0.02 | 8.40E-01 |
| rs2261181 | 0.00 | 0.02 | 7.99E-01 |
| rs2294120 | 0.01 | 0.02 | 7.59E-01 |
| rs2296173 | 0.00 | 0.02 | 7.99E-01 |
| rs2299383 | 0.01 | 0.02 | 7.77E-01 |
| rs243019 | 0.01 | 0.02 | 6.90E-01 |
| rs2493394 | 0.00 | 0.02 | 8.55E-01 |
| rs2796441 | 0.01 | 0.02 | 7.25E-01 |
| rs2820426 | 0.00 | 0.02 | 8.44E-01 |
| rs2867125 | 0.00 | 0.02 | 7.94E-01 |
| rs2908282 | 0.01 | 0.02 | 7.67E-01 |
| rs2925979 | 0.01 | 0.02 | 7.74E-01 |
| rs2943656 | 0.01 | 0.02 | 7.63E-01 |
| rs3217992 | 0.01 | 0.02 | 7.53E-01 |
| rs340874 | 0.00 | 0.02 | 8.39E-01 |
| rs348330 | 0.01 | 0.02 | 7.91E-01 |
| rs3756784 | 0.01 | 0.02 | 7.15E-01 |
| rs3802177 | 0.00 | 0.02 | 8.64E-01 |
| rs459193 | 0.01 | 0.02 | 6.76E-01 |
| rs4622883 | 0.01 | 0.02 | 7.78E-01 |
| rs4686471 | 0.01 | 0.02 | 7.48E-01 |
| rs4812829 | 0.01 | 0.02 | 7.80E-01 |
| rs4823182 | 0.01 | 0.02 | 7.92E-01 |
| rs4865796 | 0.00 | 0.02 | 8.10E-01 |
| rs516946 | 0.01 | 0.02 | 7.54E-01 |
| rs5215 | 0.00 | 0.02 | 8.35E-01 |
| rs55966194 | 0.00 | 0.02 | 8.24E-01 |
| rs576674 | 0.01 | 0.02 | 7.83E-01 |
| rs6059662 | 0.01 | 0.02 | 7.42E-01 |
| rs61953351 | 0.00 | 0.02 | 9.10E-01 |
| rs622217 | 0.00 | 0.02 | 8.14E-01 |
| rs6494307 | 0.01 | 0.02 | 7.42E-01 |
| rs6515236 | 0.01 | 0.02 | 7.81E-01 |
| rs67232546 | 0.00 | 0.02 | 8.58E-01 |
| rs6767484 | 0.01 | 0.02 | 7.87E-01 |
| rs6785040 | 0.01 | 0.02 | 7.90E-01 |
| rs6795735 | 0.01 | 0.02 | 7.30E-01 |
| rs6878122 | 0.00 | 0.02 | 8.51E-01 |
| rs6960043 | 0.01 | 0.02 | 7.68E-01 |
| rs7144011 | 0.01 | 0.02 | 7.52E-01 |
| rs7177055 | 0.01 | 0.02 | 7.83E-01 |
| rs7240767 | 0.00 | 0.02 | 8.28E-01 |
| rs72802358 | 0.01 | 0.02 | 7.17E-01 |
| rs72892910 | 0.00 | 0.02 | 8.66E-01 |
| rs735949 | 0.01 | 0.02 | 7.55E-01 |
| rs753270 | 0.00 | 0.02 | 8.44E-01 |
| rs7561798 | 0.01 | 0.02 | 7.72E-01 |
| rs7572970 | 0.00 | 0.02 | 8.44E-01 |
| rs7607777 | 0.00 | 0.02 | 9.20E-01 |
| rs7674212 | 0.00 | 0.02 | 8.05E-01 |
| rs7685296 | 0.01 | 0.02 | 7.50E-01 |
| rs7729395 | 0.01 | 0.02 | 7.61E-01 |
| rs7756992 | 0.01 | 0.02 | 7.09E-01 |
| rs7786095 | 0.01 | 0.02 | 7.88E-01 |
| rs780094 | 0.01 | 0.02 | 3.81E-01 |
| rs7845219 | 0.01 | 0.02 | 7.75E-01 |
| rs7903146 | 0.00 | 0.02 | 8.19E-01 |
| rs7929543 | 0.01 | 0.02 | 7.70E-01 |
| rs7955901 | 0.01 | 0.02 | 7.20E-01 |
| rs8068804 | 0.01 | 0.02 | 7.67E-01 |
| rs8108269 | 0.00 | 0.02 | 8.63E-01 |
| rs825476 | 0.00 | 0.02 | 8.44E-01 |
| rs840967 | 0.00 | 0.02 | 8.02E-01 |
| rs849135 | 0.01 | 0.02 | 7.35E-01 |
| rs853974 | 0.01 | 0.02 | 7.12E-01 |
| rs9369425 | 0.01 | 0.02 | 7.73E-01 |
| rs963740 | 0.01 | 0.02 | 7.67E-01 |
| rs9844972 | 0.01 | 0.02 | 7.77E-01 |
| rs9894220 | 0.01 | 0.02 | 7.54E-01 |
| rs9928094 | 0.00 | 0.02 | 9.70E-01 |
| rs993380 | 0.00 | 0.02 | 8.01E-01 |
| rs9940149 | 0.01 | 0.02 | 7.26E-01 |
| All | 0.01 | 0.02 | 7.82E-01 |

T2DM: Type-2 diabetes; TC: Total cholesterol; Se: Standard error.

Table 57: Single SNP analysis for estimate of T2DM- TC(Prins).

| SNP | Effect | Se | P-value |
| --- | --- | --- | --- |
| rs10100265 | 0.41 | 0.41 | 3.17E-01 |
| rs10114341 | 0.24 | 0.24 | 3.17E-01 |
| rs10401969 | -1.52 | 0.33 | 3.06E-06 |
| rs1050226 | 0.20 | 0.20 | 3.17E-01 |
| rs1061813 | -0.23 | 0.23 | 3.17E-01 |
| rs1063355 | -0.14 | 0.28 | 6.17E-01 |
| rs10740322 | -0.63 | 0.42 | 1.34E-01 |
| rs10811661 | -0.38 | 0.13 | 2.70E-03 |
| rs10830963 | -0.11 | 0.22 | 6.17E-01 |
| rs10842994 | -0.13 | 0.26 | 6.17E-01 |
| rs10974438 | -0.17 | 0.34 | 6.17E-01 |
| rs11098676 | -0.19 | 0.37 | 6.17E-01 |
| rs11107116 | 0.43 | 0.43 | 3.17E-01 |
| rs1127655 | -0.46 | 0.23 | 4.55E-02 |
| rs11708067 | 0.21 | 0.21 | 3.17E-01 |
| rs12299509 | 0.21 | 0.43 | 6.17E-01 |
| rs12617659 | -0.15 | 0.29 | 6.17E-01 |
| rs12910825 | 0.97 | 0.39 | 1.24E-02 |
| rs12945601 | -0.21 | 0.42 | 6.17E-01 |
| rs12970134 | 0.18 | 0.36 | 6.17E-01 |
| rs13234269 | -0.34 | 0.17 | 4.55E-02 |
| rs13239186 | -0.19 | 0.37 | 6.17E-01 |
| rs13330951 | -0.22 | 0.22 | 3.17E-01 |
| rs13389219 | -0.28 | 0.28 | 3.17E-01 |
| rs1359790 | -0.25 | 0.25 | 3.17E-01 |
| rs1496653 | 0.13 | 0.26 | 6.17E-01 |
| rs1552224 | 0.19 | 0.19 | 3.17E-01 |
| rs16988333 | -0.27 | 0.27 | 3.17E-01 |
| rs17086692 | -0.64 | 0.43 | 1.34E-01 |
| rs17168486 | 0.13 | 0.27 | 6.17E-01 |
| rs17405722 | 0.11 | 0.34 | 7.39E-01 |
| rs17411031 | 0.22 | 0.44 | 6.17E-01 |
| rs1758632 | -0.41 | 0.20 | 4.55E-02 |
| rs17631783 | -0.21 | 0.41 | 6.17E-01 |
| rs17791513 | 0.58 | 0.29 | 4.55E-02 |
| rs1801214 | 0.11 | 0.11 | 3.17E-01 |
| rs1899951 | -0.09 | 0.18 | 6.17E-01 |
| rs2058913 | 0.20 | 0.20 | 3.17E-01 |
| rs2237892 | -0.42 | 0.31 | 1.82E-01 |
| rs2246618 | -0.39 | 0.39 | 3.17E-01 |
| rs2261181 | -0.10 | 0.20 | 6.17E-01 |
| rs2294120 | 0.23 | 0.23 | 3.17E-01 |
| rs2296173 | -0.62 | 0.31 | 4.55E-02 |
| rs2299383 | 0.24 | 0.24 | 3.17E-01 |
| rs243019 | -0.18 | 0.18 | 3.17E-01 |
| rs2493394 | -0.14 | 0.27 | 6.17E-01 |
| rs2796441 | -0.28 | 0.14 | 4.55E-02 |
| rs2820426 | 0.19 | 0.38 | 6.17E-01 |
| rs2867125 | -0.17 | 0.33 | 6.17E-01 |
| rs2925979 | 0.19 | 0.37 | 6.17E-01 |
| rs2943656 | -0.11 | 0.22 | 6.17E-01 |
| rs3217992 | 0.19 | 0.38 | 6.17E-01 |
| rs3756784 | -0.40 | 0.40 | 3.17E-01 |
| rs459193 | 0.14 | 0.28 | 6.17E-01 |
| rs4622883 | 0.23 | 0.23 | 3.17E-01 |
| rs4823182 | -0.21 | 0.41 | 6.17E-01 |
| rs516946 | -0.12 | 0.24 | 6.17E-01 |
| rs5215 | 0.44 | 0.29 | 1.34E-01 |
| rs576674 | 0.15 | 0.31 | 6.17E-01 |
| rs6059662 | -0.45 | 0.45 | 3.17E-01 |
| rs61953351 | 0.29 | 0.29 | 3.17E-01 |
| rs6494307 | -0.45 | 0.23 | 4.55E-02 |
| rs67232546 | 0.17 | 0.34 | 6.17E-01 |
| rs6767484 | 0.08 | 0.17 | 6.17E-01 |
| rs6785040 | 0.16 | 0.32 | 6.17E-01 |
| rs6795735 | -0.36 | 0.18 | 4.55E-02 |
| rs6960043 | -0.16 | 0.16 | 3.17E-01 |
| rs7144011 | -0.41 | 0.41 | 3.17E-01 |
| rs7177055 | 0.15 | 0.31 | 6.17E-01 |
| rs7240767 | -0.22 | 0.22 | 3.17E-01 |
| rs72802358 | -0.09 | 0.17 | 6.17E-01 |
| rs72892910 | 0.46 | 0.31 | 1.34E-01 |
| rs735949 | -0.42 | 0.28 | 1.34E-01 |
| rs753270 | 0.19 | 0.19 | 3.17E-01 |
| rs7561798 | 0.25 | 0.25 | 3.17E-01 |
| rs7572970 | 0.17 | 0.34 | 6.17E-01 |
| rs7607777 | -0.07 | 0.15 | 6.17E-01 |
| rs7674212 | 0.22 | 0.43 | 6.17E-01 |
| rs7685296 | -0.78 | 0.39 | 4.55E-02 |
| rs7729395 | 0.15 | 0.22 | 5.05E-01 |
| rs7756992 | -0.08 | 0.15 | 6.17E-01 |
| rs7786095 | -0.67 | 0.27 | 1.24E-02 |
| rs780094 | -0.43 | 0.29 | 1.34E-01 |
| rs7845219 | 0.24 | 0.24 | 3.17E-01 |
| rs7903146 | -0.10 | 0.07 | 1.34E-01 |
| rs7929543 | -0.60 | 0.36 | 9.56E-02 |
| rs7955901 | -0.45 | 0.45 | 3.17E-01 |
| rs8068804 | -0.17 | 0.34 | 6.17E-01 |
| rs8108269 | 0.31 | 0.31 | 3.17E-01 |
| rs825476 | -0.19 | 0.19 | 3.17E-01 |
| rs840967 | 0.20 | 0.20 | 3.17E-01 |
| rs849135 | 0.20 | 0.10 | 4.55E-02 |
| rs853974 | -0.33 | 0.33 | 3.17E-01 |
| rs9369425 | 0.18 | 0.37 | 6.17E-01 |
| rs9894220 | -0.17 | 0.17 | 3.17E-01 |
| rs9928094 | -0.10 | 0.19 | 6.17E-01 |
| rs993380 | 0.20 | 0.39 | 6.17E-01 |
| rs9940149 | 0.17 | 0.34 | 6.17E-01 |
| All - Inverse variance weighted | -0.06 | 0.03 | 1.80E-02 |
| All - MR Egger | -0.09 | 0.07 | 1.67E-01 |

T2DM: Type-2 diabetes; TC: Total cholesterol; Se: Standard error.

Table 58: Leave-one-out analysis for estimate of T2DM- TC(Prins).

| SNP | Effect | Se | P-value |
| --- | --- | --- | --- |
| rs10100265 | -0.07 | 0.03 | 1.57E-02 |
| rs10114341 | -0.07 | 0.03 | 1.41E-02 |
| rs10401969 | -0.06 | 0.03 | 2.39E-02 |
| rs1050226 | -0.07 | 0.03 | 1.33E-02 |
| rs1061813 | -0.06 | 0.03 | 2.21E-02 |
| rs1063355 | -0.06 | 0.03 | 1.99E-02 |
| rs10740322 | -0.06 | 0.03 | 2.12E-02 |
| rs10811661 | -0.05 | 0.03 | 4.68E-02 |
| rs10830963 | -0.06 | 0.03 | 2.01E-02 |
| rs10842994 | -0.06 | 0.03 | 2.00E-02 |
| rs10974438 | -0.06 | 0.03 | 1.97E-02 |
| rs11098676 | -0.06 | 0.03 | 1.96E-02 |
| rs11107116 | -0.07 | 0.03 | 1.59E-02 |
| rs1127655 | -0.06 | 0.03 | 2.62E-02 |
| rs11708067 | -0.07 | 0.03 | 1.33E-02 |
| rs12299509 | -0.06 | 0.03 | 1.72E-02 |
| rs12617659 | -0.06 | 0.03 | 1.99E-02 |
| rs12910825 | -0.07 | 0.03 | 1.09E-02 |
| rs12945601 | -0.06 | 0.03 | 1.95E-02 |
| rs12970134 | -0.07 | 0.03 | 1.69E-02 |
| rs13234269 | -0.06 | 0.03 | 2.97E-02 |
| rs13239186 | -0.06 | 0.03 | 1.96E-02 |
| rs13330951 | -0.06 | 0.03 | 2.24E-02 |
| rs13389219 | -0.06 | 0.03 | 2.15E-02 |
| rs1359790 | -0.06 | 0.03 | 2.19E-02 |
| rs1496653 | -0.07 | 0.03 | 1.62E-02 |
| rs1552224 | -0.07 | 0.03 | 1.30E-02 |
| rs16988333 | -0.06 | 0.03 | 2.16E-02 |
| rs17086692 | -0.06 | 0.03 | 2.11E-02 |
| rs17168486 | -0.07 | 0.03 | 1.63E-02 |
| rs17405722 | -0.06 | 0.03 | 1.74E-02 |
| rs17411031 | -0.06 | 0.03 | 1.72E-02 |
| rs1758632 | -0.06 | 0.03 | 2.74E-02 |
| rs17631783 | -0.06 | 0.03 | 1.95E-02 |
| rs17791513 | -0.07 | 0.03 | 1.12E-02 |
| rs1801214 | -0.07 | 0.03 | 9.26E-03 |
| rs1899951 | -0.06 | 0.03 | 2.03E-02 |
| rs2058913 | -0.07 | 0.03 | 1.33E-02 |
| rs2237892 | -0.06 | 0.03 | 2.20E-02 |
| rs2246618 | -0.06 | 0.03 | 2.06E-02 |
| rs2261181 | -0.06 | 0.03 | 2.02E-02 |
| rs2294120 | -0.07 | 0.03 | 1.37E-02 |
| rs2296173 | -0.06 | 0.03 | 2.36E-02 |
| rs2299383 | -0.07 | 0.03 | 1.41E-02 |
| rs243019 | -0.06 | 0.03 | 2.33E-02 |
| rs2493394 | -0.06 | 0.03 | 1.99E-02 |
| rs2796441 | -0.06 | 0.03 | 3.31E-02 |
| rs2820426 | -0.07 | 0.03 | 1.70E-02 |
| rs2867125 | -0.06 | 0.03 | 1.97E-02 |
| rs2925979 | -0.07 | 0.03 | 1.70E-02 |
| rs2943656 | -0.06 | 0.03 | 2.01E-02 |
| rs3217992 | -0.07 | 0.03 | 1.70E-02 |
| rs3756784 | -0.06 | 0.03 | 2.06E-02 |
| rs459193 | -0.07 | 0.03 | 1.64E-02 |
| rs4622883 | -0.07 | 0.03 | 1.38E-02 |
| rs4823182 | -0.06 | 0.03 | 1.95E-02 |
| rs516946 | -0.06 | 0.03 | 2.00E-02 |
| rs5215 | -0.07 | 0.03 | 1.30E-02 |
| rs576674 | -0.07 | 0.03 | 1.66E-02 |
| rs6059662 | -0.06 | 0.03 | 2.03E-02 |
| rs61953351 | -0.07 | 0.03 | 1.47E-02 |
| rs6494307 | -0.06 | 0.03 | 2.63E-02 |
| rs67232546 | -0.07 | 0.03 | 1.68E-02 |
| rs6767484 | -0.07 | 0.03 | 1.47E-02 |
| rs6785040 | -0.07 | 0.03 | 1.67E-02 |
| rs6795735 | -0.06 | 0.03 | 2.91E-02 |
| rs6960043 | -0.06 | 0.03 | 2.38E-02 |
| rs7144011 | -0.06 | 0.03 | 2.05E-02 |
| rs7177055 | -0.07 | 0.03 | 1.66E-02 |
| rs7240767 | -0.06 | 0.03 | 2.23E-02 |
| rs72802358 | -0.06 | 0.03 | 2.04E-02 |
| rs72892910 | -0.07 | 0.03 | 1.32E-02 |
| rs735949 | -0.06 | 0.03 | 2.30E-02 |
| rs753270 | -0.07 | 0.03 | 1.29E-02 |
| rs7561798 | -0.07 | 0.03 | 1.42E-02 |
| rs7572970 | -0.07 | 0.03 | 1.68E-02 |
| rs7607777 | -0.06 | 0.03 | 2.05E-02 |
| rs7674212 | -0.06 | 0.03 | 1.72E-02 |
| rs7685296 | -0.06 | 0.03 | 2.21E-02 |
| rs7729395 | -0.07 | 0.03 | 1.50E-02 |
| rs7756992 | -0.06 | 0.03 | 2.05E-02 |
| rs7786095 | -0.06 | 0.03 | 2.59E-02 |
| rs780094 | -0.06 | 0.03 | 2.28E-02 |
| rs7845219 | -0.07 | 0.03 | 1.40E-02 |
| rs7903146 | -0.06 | 0.03 | 4.02E-02 |
| rs7929543 | -0.06 | 0.03 | 2.20E-02 |
| rs7955901 | -0.06 | 0.03 | 2.03E-02 |
| rs8068804 | -0.06 | 0.03 | 1.97E-02 |
| rs8108269 | -0.07 | 0.03 | 1.50E-02 |
| rs825476 | -0.06 | 0.03 | 2.29E-02 |
| rs840967 | -0.07 | 0.03 | 1.32E-02 |
| rs849135 | -0.08 | 0.03 | 3.99E-03 |
| rs853974 | -0.06 | 0.03 | 2.10E-02 |
| rs9369425 | -0.07 | 0.03 | 1.70E-02 |
| rs9894220 | -0.06 | 0.03 | 2.34E-02 |
| rs9928094 | -0.06 | 0.03 | 2.03E-02 |
| rs993380 | -0.06 | 0.03 | 1.71E-02 |
| rs9940149 | -0.07 | 0.03 | 1.68E-02 |
| All | -0.06 | 0.03 | 1.80E-02 |

T2DM: Type-2 diabetes; TC: Total cholesterol; Se: Standard error.

Table 59: Single SNP analysis for estimate of T2DM-TC(Kanai).

| SNP | Effect | Se | P-value |
| --- | --- | --- | --- |
| rs10077431 | -0.04 | 0.17 | 8.03E-01 |
| rs10087241 | -0.03 | 0.16 | 8.46E-01 |
| rs10100265 | 0.21 | 0.08 | 1.45E-02 |
| rs10114341 | 0.06 | 0.13 | 6.44E-01 |
| rs10401969 | -0.03 | 0.07 | 7.12E-01 |
| rs1050226 | -0.02 | 0.08 | 7.72E-01 |
| rs1061813 | -0.09 | 0.16 | 5.86E-01 |
| rs1063355 | -0.17 | 0.05 | 1.52E-03 |
| rs10740322 | -0.19 | 0.09 | 4.04E-02 |
| rs10811661 | 0.04 | 0.03 | 1.70E-01 |
| rs10830963 | -0.04 | 0.04 | 3.58E-01 |
| rs10842994 | 0.07 | 0.07 | 3.20E-01 |
| rs10974438 | 0.07 | 0.07 | 3.21E-01 |
| rs11098676 | 0.16 | 0.16 | 3.10E-01 |
| rs11107116 | -0.14 | 0.09 | 1.33E-01 |
| rs1111875 | 0.02 | 0.05 | 7.22E-01 |
| rs11257655 | 0.07 | 0.05 | 2.07E-01 |
| rs1127655 | -0.04 | 0.09 | 6.83E-01 |
| rs11926707 | -0.11 | 0.10 | 2.91E-01 |
| rs12088739 | 0.15 | 0.08 | 5.85E-02 |
| rs12299509 | 0.05 | 0.08 | 5.84E-01 |
| rs12617659 | -0.10 | 0.08 | 2.20E-01 |
| rs12945601 | 0.12 | 0.12 | 3.44E-01 |
| rs12970134 | 0.03 | 0.09 | 7.34E-01 |
| rs13234269 | -0.04 | 0.08 | 6.15E-01 |
| rs13239186 | -0.04 | 0.11 | 6.77E-01 |
| rs13330951 | 0.31 | 0.12 | 1.19E-02 |
| rs13389219 | 0.04 | 0.10 | 7.03E-01 |
| rs1359790 | 0.10 | 0.06 | 6.50E-02 |
| rs1496653 | 0.06 | 0.07 | 4.17E-01 |
| rs1552224 | 0.06 | 0.10 | 5.67E-01 |
| rs17086692 | -0.24 | 0.10 | 1.33E-02 |
| rs17168486 | -0.02 | 0.05 | 6.89E-01 |
| rs17405722 | 0.32 | 0.14 | 2.03E-02 |
| rs17411031 | -0.18 | 0.11 | 9.75E-02 |
| rs1758632 | -0.03 | 0.09 | 7.50E-01 |
| rs17631783 | -0.20 | 0.17 | 2.46E-01 |
| rs17791513 | 0.08 | 0.08 | 3.49E-01 |
| rs1801214 | -0.17 | 0.17 | 3.28E-01 |
| rs1899951 | 0.04 | 0.10 | 6.80E-01 |
| rs2058913 | 0.10 | 0.16 | 5.38E-01 |
| rs2237892 | 0.07 | 0.04 | 7.72E-02 |
| rs2246618 | 0.09 | 0.10 | 3.56E-01 |
| rs2261181 | -0.01 | 0.06 | 9.06E-01 |
| rs2294120 | -0.10 | 0.10 | 3.49E-01 |
| rs2296173 | -0.06 | 0.08 | 4.43E-01 |
| rs2299383 | 0.08 | 0.10 | 4.21E-01 |
| rs243019 | 0.03 | 0.07 | 6.51E-01 |
| rs2493394 | 0.48 | 0.20 | 1.97E-02 |
| rs2796441 | -0.06 | 0.06 | 2.64E-01 |
| rs2820426 | 0.07 | 0.07 | 3.55E-01 |
| rs2867125 | -0.10 | 0.11 | 3.57E-01 |
| rs2908282 | 0.03 | 0.09 | 7.56E-01 |
| rs2925979 | -0.02 | 0.08 | 7.72E-01 |
| rs2943656 | 0.01 | 0.07 | 8.59E-01 |
| rs3217992 | 0.00 | 0.07 | 9.69E-01 |
| rs340874 | -0.07 | 0.06 | 2.70E-01 |
| rs348330 | 0.08 | 0.10 | 4.10E-01 |
| rs3756784 | -0.09 | 0.08 | 2.80E-01 |
| rs3802177 | -0.01 | 0.03 | 8.67E-01 |
| rs459193 | -0.07 | 0.06 | 1.96E-01 |
| rs4622883 | 0.02 | 0.11 | 8.74E-01 |
| rs4812829 | 0.08 | 0.07 | 2.76E-01 |
| rs4823182 | -0.36 | 0.08 | 8.11E-06 |
| rs4865796 | -0.03 | 0.10 | 7.44E-01 |
| rs516946 | 0.07 | 0.07 | 3.36E-01 |
| rs5215 | 0.07 | 0.06 | 2.09E-01 |
| rs576674 | 0.00 | 0.09 | 9.88E-01 |
| rs6059662 | -0.10 | 0.11 | 3.61E-01 |
| rs622217 | 0.00 | 0.09 | 9.87E-01 |
| rs6494307 | 0.01 | 0.09 | 9.55E-01 |
| rs6515236 | 0.03 | 0.08 | 7.34E-01 |
| rs6767484 | -0.05 | 0.03 | 1.66E-01 |
| rs6785040 | 0.08 | 0.07 | 2.06E-01 |
| rs6795735 | 0.00 | 0.09 | 9.67E-01 |
| rs6878122 | -0.42 | 0.26 | 1.06E-01 |
| rs6960043 | -0.03 | 0.06 | 6.71E-01 |
| rs7177055 | 0.02 | 0.06 | 7.57E-01 |
| rs7240767 | 0.12 | 0.09 | 2.21E-01 |
| rs72802358 | -0.20 | 0.13 | 1.30E-01 |
| rs72892910 | 0.06 | 0.07 | 3.89E-01 |
| rs753270 | 0.04 | 0.09 | 6.51E-01 |
| rs7561798 | 0.16 | 0.11 | 1.37E-01 |
| rs7572970 | 0.01 | 0.09 | 9.00E-01 |
| rs7607777 | -0.08 | 0.15 | 5.95E-01 |
| rs7674212 | -0.03 | 0.09 | 7.20E-01 |
| rs7685296 | -0.09 | 0.08 | 2.33E-01 |
| rs7756992 | 0.00 | 0.03 | 9.20E-01 |
| rs7786095 | 0.13 | 0.10 | 2.19E-01 |
| rs780094 | -0.51 | 0.06 | 4.22E-19 |
| rs7845219 | -0.06 | 0.10 | 5.49E-01 |
| rs7903146 | 0.02 | 0.03 | 5.03E-01 |
| rs7929543 | -0.05 | 0.06 | 4.25E-01 |
| rs7955901 | 0.02 | 0.09 | 8.64E-01 |
| rs8068804 | -0.15 | 0.07 | 4.73E-02 |
| rs8108269 | 0.15 | 0.06 | 1.68E-02 |
| rs825476 | 0.05 | 0.09 | 5.94E-01 |
| rs840967 | 0.04 | 0.08 | 5.83E-01 |
| rs853974 | -0.01 | 0.07 | 8.88E-01 |
| rs9369425 | -0.06 | 0.11 | 5.92E-01 |
| rs963740 | -0.09 | 0.08 | 2.90E-01 |
| rs9894220 | -0.05 | 0.08 | 4.74E-01 |
| rs9928094 | 0.04 | 0.04 | 3.13E-01 |
| rs993380 | -0.02 | 0.08 | 8.00E-01 |
| rs9940149 | -0.05 | 0.07 | 4.58E-01 |
| All - Inverse variance weighted | 0.00 | 0.01 | 6.25E-01 |
| All - MR Egger | 0.03 | 0.03 | 3.27E-01 |

T2DM: Type-2 diabetes; TC: Total cholesterol; Se: Standard error.

Table 60: Leave-one-out analysis for estimate of T2DM-TC(Kanai).

| SNP | Effect | Se | P-value |
| --- | --- | --- | --- |
| rs10077431 | 0.00 | 0.01 | 6.31E-01 |
| rs10087241 | 0.00 | 0.01 | 6.31E-01 |
| rs10100265 | -0.01 | 0.01 | 5.28E-01 |
| rs10114341 | -0.01 | 0.01 | 6.14E-01 |
| rs10401969 | 0.00 | 0.01 | 6.42E-01 |
| rs1050226 | 0.00 | 0.01 | 6.38E-01 |
| rs1061813 | 0.00 | 0.01 | 6.37E-01 |
| rs1063355 | 0.00 | 0.01 | 8.22E-01 |
| rs10740322 | 0.00 | 0.01 | 6.98E-01 |
| rs10811661 | -0.01 | 0.01 | 4.58E-01 |
| rs10830963 | 0.00 | 0.01 | 6.94E-01 |
| rs10842994 | -0.01 | 0.01 | 5.75E-01 |
| rs10974438 | -0.01 | 0.01 | 5.77E-01 |
| rs11098676 | -0.01 | 0.01 | 6.05E-01 |
| rs11107116 | 0.00 | 0.01 | 6.80E-01 |
| rs1111875 | -0.01 | 0.01 | 5.97E-01 |
| rs11257655 | -0.01 | 0.01 | 5.46E-01 |
| rs1127655 | 0.00 | 0.01 | 6.40E-01 |
| rs11926707 | 0.00 | 0.01 | 6.59E-01 |
| rs12088739 | -0.01 | 0.01 | 5.47E-01 |
| rs12299509 | -0.01 | 0.01 | 6.04E-01 |
| rs12617659 | 0.00 | 0.01 | 6.74E-01 |
| rs12945601 | -0.01 | 0.01 | 6.00E-01 |
| rs12970134 | -0.01 | 0.01 | 6.14E-01 |
| rs13234269 | 0.00 | 0.01 | 6.46E-01 |
| rs13239186 | 0.00 | 0.01 | 6.39E-01 |
| rs13330951 | -0.01 | 0.01 | 5.56E-01 |
| rs13389219 | -0.01 | 0.01 | 6.13E-01 |
| rs1359790 | -0.01 | 0.01 | 5.17E-01 |
| rs1496653 | -0.01 | 0.01 | 5.87E-01 |
| rs1552224 | -0.01 | 0.01 | 6.07E-01 |
| rs17086692 | 0.00 | 0.01 | 7.08E-01 |
| rs17168486 | 0.00 | 0.01 | 6.49E-01 |
| rs17405722 | -0.01 | 0.01 | 5.68E-01 |
| rs17411031 | 0.00 | 0.01 | 6.75E-01 |
| rs1758632 | 0.00 | 0.01 | 6.37E-01 |
| rs17631783 | 0.00 | 0.01 | 6.48E-01 |
| rs17791513 | -0.01 | 0.01 | 5.89E-01 |
| rs1801214 | 0.00 | 0.01 | 6.44E-01 |
| rs1899951 | -0.01 | 0.01 | 6.12E-01 |
| rs2058913 | -0.01 | 0.01 | 6.13E-01 |
| rs2237892 | -0.01 | 0.01 | 4.85E-01 |
| rs2246618 | -0.01 | 0.01 | 5.96E-01 |
| rs2261181 | 0.00 | 0.01 | 6.31E-01 |
| rs2294120 | 0.00 | 0.01 | 6.56E-01 |
| rs2296173 | 0.00 | 0.01 | 6.57E-01 |
| rs2299383 | -0.01 | 0.01 | 5.98E-01 |
| rs243019 | -0.01 | 0.01 | 6.05E-01 |
| rs2493394 | -0.01 | 0.01 | 5.85E-01 |
| rs2796441 | 0.00 | 0.01 | 6.91E-01 |
| rs2820426 | -0.01 | 0.01 | 5.84E-01 |
| rs2867125 | 0.00 | 0.01 | 6.54E-01 |
| rs2908282 | -0.01 | 0.01 | 6.14E-01 |
| rs2925979 | 0.00 | 0.01 | 6.38E-01 |
| rs2943656 | -0.01 | 0.01 | 6.18E-01 |
| rs3217992 | 0.00 | 0.01 | 6.24E-01 |
| rs340874 | 0.00 | 0.01 | 6.81E-01 |
| rs348330 | -0.01 | 0.01 | 5.99E-01 |
| rs3756784 | 0.00 | 0.01 | 6.70E-01 |
| rs3802177 | 0.00 | 0.01 | 6.36E-01 |
| rs459193 | 0.00 | 0.01 | 7.03E-01 |
| rs4622883 | 0.00 | 0.01 | 6.21E-01 |
| rs4812829 | -0.01 | 0.01 | 5.76E-01 |
| rs4823182 | 0.00 | 0.01 | 8.09E-01 |
| rs4865796 | 0.00 | 0.01 | 6.37E-01 |
| rs516946 | -0.01 | 0.01 | 5.78E-01 |
| rs5215 | -0.01 | 0.01 | 5.55E-01 |
| rs576674 | 0.00 | 0.01 | 6.26E-01 |
| rs6059662 | 0.00 | 0.01 | 6.53E-01 |
| rs622217 | 0.00 | 0.01 | 6.25E-01 |
| rs6494307 | 0.00 | 0.01 | 6.24E-01 |
| rs6515236 | -0.01 | 0.01 | 6.12E-01 |
| rs6767484 | 0.00 | 0.01 | 7.58E-01 |
| rs6785040 | -0.01 | 0.01 | 5.62E-01 |
| rs6795735 | 0.00 | 0.01 | 6.27E-01 |
| rs6878122 | 0.00 | 0.01 | 6.46E-01 |
| rs6960043 | 0.00 | 0.01 | 6.48E-01 |
| rs7177055 | -0.01 | 0.01 | 6.08E-01 |
| rs7240767 | -0.01 | 0.01 | 5.83E-01 |
| rs72802358 | 0.00 | 0.01 | 6.62E-01 |
| rs72892910 | -0.01 | 0.01 | 5.87E-01 |
| rs753270 | -0.01 | 0.01 | 6.10E-01 |
| rs7561798 | -0.01 | 0.01 | 5.80E-01 |
| rs7572970 | 0.00 | 0.01 | 6.21E-01 |
| rs7607777 | 0.00 | 0.01 | 6.38E-01 |
| rs7674212 | 0.00 | 0.01 | 6.39E-01 |
| rs7685296 | 0.00 | 0.01 | 6.77E-01 |
| rs7756992 | -0.01 | 0.01 | 6.06E-01 |
| rs7786095 | -0.01 | 0.01 | 5.86E-01 |
| rs780094 | 0.00 | 0.01 | 7.59E-01 |
| rs7845219 | 0.00 | 0.01 | 6.45E-01 |
| rs7903146 | -0.01 | 0.01 | 5.46E-01 |
| rs7929543 | 0.00 | 0.01 | 6.66E-01 |
| rs7955901 | -0.01 | 0.01 | 6.20E-01 |
| rs8068804 | 0.00 | 0.01 | 7.14E-01 |
| rs8108269 | -0.01 | 0.01 | 4.99E-01 |
| rs825476 | -0.01 | 0.01 | 6.06E-01 |
| rs840967 | -0.01 | 0.01 | 6.03E-01 |
| rs853974 | 0.00 | 0.01 | 6.32E-01 |
| rs9369425 | 0.00 | 0.01 | 6.42E-01 |
| rs963740 | 0.00 | 0.01 | 6.68E-01 |
| rs9894220 | 0.00 | 0.01 | 6.56E-01 |
| rs9928094 | -0.01 | 0.01 | 5.48E-01 |
| rs993380 | 0.00 | 0.01 | 6.36E-01 |
| rs9940149 | 0.00 | 0.01 | 6.60E-01 |
| All | 0.00 | 0.01 | 6.25E-01 |

T2DM: Type-2 diabetes; TC: Total cholesterol; Se: Standard error.

Table 61: Single SNP analysis for estimate of T2DM-TG(Willer).

| SNP | Effect | Se | P-value |
| --- | --- | --- | --- |
| rs10077431 | 0.16 | 0.09 | 6.33E-02 |
| rs10100265 | -0.31 | 0.07 | 6.80E-06 |
| rs10114341 | 0.21 | 0.08 | 8.38E-03 |
| rs10401969 | -1.31 | 0.07 | 2.41E-77 |
| rs1050226 | 0.05 | 0.07 | 4.99E-01 |
| rs1061813 | -0.08 | 0.08 | 3.32E-01 |
| rs1063355 | -0.31 | 0.08 | 5.41E-05 |
| rs10740322 | -0.07 | 0.11 | 5.01E-01 |
| rs10811661 | 0.02 | 0.03 | 4.67E-01 |
| rs10830963 | 0.05 | 0.04 | 2.82E-01 |
| rs10842994 | -0.01 | 0.06 | 7.93E-01 |
| rs10974438 | 0.02 | 0.06 | 7.97E-01 |
| rs11098676 | 0.26 | 0.10 | 1.12E-02 |
| rs11107116 | 0.11 | 0.09 | 2.02E-01 |
| rs1111875 | 0.11 | 0.03 | 1.62E-03 |
| rs11257655 | 0.03 | 0.05 | 6.17E-01 |
| rs1127655 | 0.19 | 0.11 | 6.78E-02 |
| rs11708067 | -0.04 | 0.04 | 3.06E-01 |
| rs11925227 | 0.00 | 0.08 | 9.64E-01 |
| rs11926707 | 0.26 | 0.11 | 1.64E-02 |
| rs12088739 | 0.21 | 0.07 | 1.70E-03 |
| rs12299509 | 0.01 | 0.07 | 8.64E-01 |
| rs12617659 | 0.27 | 0.07 | 1.08E-04 |
| rs12910825 | 0.05 | 0.07 | 4.75E-01 |
| rs12945601 | 0.31 | 0.10 | 2.19E-03 |
| rs12970134 | 0.23 | 0.07 | 1.13E-03 |
| rs13234269 | 0.40 | 0.08 | 5.73E-07 |
| rs13239186 | 0.13 | 0.09 | 1.53E-01 |
| rs13330951 | 0.19 | 0.10 | 6.73E-02 |
| rs13389219 | 0.38 | 0.05 | 1.58E-15 |
| rs1359790 | -0.02 | 0.05 | 7.25E-01 |
| rs1496653 | -0.06 | 0.05 | 2.70E-01 |
| rs1552224 | -0.06 | 0.04 | 1.28E-01 |
| rs16988333 | 0.04 | 0.08 | 5.94E-01 |
| rs17086692 | 0.12 | 0.11 | 2.90E-01 |
| rs17168486 | -0.03 | 0.06 | 6.09E-01 |
| rs17405722 | -0.02 | 0.10 | 8.56E-01 |
| rs17411031 | 2.39 | 0.08 | 1.37E-185 |
| rs1758632 | 0.22 | 0.10 | 2.44E-02 |
| rs17631783 | 0.03 | 0.11 | 7.81E-01 |
| rs17791513 | 0.01 | 0.06 | 8.15E-01 |
| rs1801214 | 0.01 | 0.04 | 8.14E-01 |
| rs1899951 | 0.14 | 0.04 | 1.02E-03 |
| rs2058913 | 0.34 | 0.10 | 5.03E-04 |
| rs2237892 | 0.06 | 0.08 | 4.37E-01 |
| rs2246618 | 0.08 | 0.08 | 3.05E-01 |
| rs2261181 | 0.11 | 0.06 | 5.56E-02 |
| rs2294120 | -0.05 | 0.10 | 6.42E-01 |
| rs2296173 | 0.32 | 0.06 | 5.73E-07 |
| rs2299383 | 0.15 | 0.08 | 6.82E-02 |
| rs243019 | -0.04 | 0.06 | 5.37E-01 |
| rs2493394 | 0.05 | 0.07 | 4.97E-01 |
| rs2796441 | -0.08 | 0.05 | 1.16E-01 |
| rs2820426 | 0.32 | 0.07 | 1.05E-06 |
| rs2867125 | 0.12 | 0.07 | 8.96E-02 |
| rs2908282 | 0.06 | 0.08 | 4.73E-01 |
| rs2925979 | 0.38 | 0.07 | 1.24E-08 |
| rs2943656 | 0.29 | 0.04 | 1.03E-14 |
| rs3217992 | -0.04 | 0.06 | 5.37E-01 |
| rs340874 | 0.18 | 0.05 | 7.69E-04 |
| rs3756784 | 0.19 | 0.09 | 2.56E-02 |
| rs3802177 | 0.05 | 0.03 | 1.01E-01 |
| rs459193 | 0.25 | 0.05 | 2.81E-06 |
| rs4622883 | 0.11 | 0.11 | 3.27E-01 |
| rs4812829 | -0.03 | 0.08 | 6.99E-01 |
| rs4823182 | 0.00 | 0.07 | 9.54E-01 |
| rs4865796 | 0.18 | 0.07 | 8.32E-03 |
| rs516946 | -0.07 | 0.05 | 1.51E-01 |
| rs5215 | -0.08 | 0.05 | 1.06E-01 |
| rs55966194 | 0.57 | 0.10 | 3.42E-08 |
| rs576674 | 0.07 | 0.07 | 3.17E-01 |
| rs6059662 | 0.21 | 0.08 | 9.79E-03 |
| rs622217 | 0.35 | 0.09 | 1.89E-04 |
| rs6494307 | 0.12 | 0.11 | 2.61E-01 |
| rs6515236 | -0.12 | 0.11 | 2.84E-01 |
| rs6767484 | 0.05 | 0.03 | 6.75E-02 |
| rs6785040 | 0.02 | 0.11 | 8.73E-01 |
| rs6795735 | 0.13 | 0.06 | 3.95E-02 |
| rs6878122 | 0.04 | 0.07 | 5.22E-01 |
| rs6960043 | 0.02 | 0.05 | 6.81E-01 |
| rs7144011 | 0.11 | 0.08 | 1.69E-01 |
| rs7177055 | -0.02 | 0.06 | 6.85E-01 |
| rs7240767 | 0.03 | 0.11 | 7.95E-01 |
| rs72802358 | 0.01 | 0.04 | 9.04E-01 |
| rs72892910 | -0.03 | 0.10 | 7.84E-01 |
| rs735949 | 0.09 | 0.07 | 1.82E-01 |
| rs753270 | 0.18 | 0.09 | 5.01E-02 |
| rs7561798 | 0.07 | 0.08 | 4.31E-01 |
| rs7572970 | 0.17 | 0.09 | 6.18E-02 |
| rs7607777 | 0.04 | 0.04 | 3.09E-01 |
| rs7685296 | 0.15 | 0.07 | 3.76E-02 |
| rs7729395 | 0.09 | 0.06 | 1.57E-01 |
| rs7756992 | 0.08 | 0.03 | 3.22E-03 |
| rs7786095 | 0.02 | 0.10 | 8.23E-01 |
| rs780094 | -1.59 | 0.05 | 1.87E-230 |
| rs7845219 | 0.17 | 0.08 | 2.91E-02 |
| rs7903146 | 0.00 | 0.01 | 8.71E-01 |
| rs7929543 | 0.14 | 0.08 | 8.69E-02 |
| rs7955901 | 0.04 | 0.07 | 6.28E-01 |
| rs8068804 | -0.01 | 0.06 | 8.50E-01 |
| rs8108269 | 0.10 | 0.06 | 8.72E-02 |
| rs825476 | 0.18 | 0.06 | 4.39E-03 |
| rs840967 | 0.12 | 0.10 | 2.19E-01 |
| rs849135 | 0.02 | 0.03 | 6.28E-01 |
| rs9369425 | -0.17 | 0.10 | 7.07E-02 |
| rs963740 | -0.27 | 0.11 | 1.38E-02 |
| rs9844972 | 0.37 | 0.10 | 1.20E-04 |
| rs9894220 | 0.07 | 0.08 | 4.03E-01 |
| rs9928094 | 0.18 | 0.03 | 3.80E-08 |
| rs993380 | 0.00 | 0.09 | 9.83E-01 |
| rs9940149 | -0.06 | 0.08 | 4.37E-01 |
| All - Inverse variance weighted | 0.05 | 0.03 | 8.40E-02 |
| All - MR Egger | -0.03 | 0.06 | 5.90E-01 |

T2DM: Type-2 diabetes; TG: Triglycerides; Se: Standard error.

Table 62: Leave-one-out analysis for estimate of T2DM-TG(Willer).

| SNP | Effect | Se | P-value |
| --- | --- | --- | --- |
| rs10077431 | 0.04 | 0.03 | 8.88E-02 |
| rs10100265 | 0.05 | 0.03 | 7.18E-02 |
| rs10114341 | 0.04 | 0.03 | 9.08E-02 |
| rs10401969 | 0.05 | 0.02 | 3.21E-02 |
| rs1050226 | 0.05 | 0.03 | 8.64E-02 |
| rs1061813 | 0.05 | 0.03 | 8.24E-02 |
| rs1063355 | 0.05 | 0.03 | 7.42E-02 |
| rs10740322 | 0.05 | 0.03 | 8.39E-02 |
| rs10811661 | 0.05 | 0.03 | 8.48E-02 |
| rs10830963 | 0.05 | 0.03 | 8.78E-02 |
| rs10842994 | 0.05 | 0.03 | 8.31E-02 |
| rs10974438 | 0.05 | 0.03 | 8.50E-02 |
| rs11098676 | 0.04 | 0.03 | 8.94E-02 |
| rs11107116 | 0.04 | 0.03 | 8.76E-02 |
| rs1111875 | 0.04 | 0.03 | 9.96E-02 |
| rs11257655 | 0.05 | 0.03 | 8.57E-02 |
| rs1127655 | 0.04 | 0.03 | 8.82E-02 |
| rs11708067 | 0.05 | 0.03 | 7.86E-02 |
| rs11925227 | 0.05 | 0.03 | 8.47E-02 |
| rs11926707 | 0.04 | 0.03 | 8.90E-02 |
| rs12088739 | 0.04 | 0.03 | 9.33E-02 |
| rs12299509 | 0.05 | 0.03 | 8.51E-02 |
| rs12617659 | 0.04 | 0.03 | 9.49E-02 |
| rs12910825 | 0.05 | 0.03 | 8.65E-02 |
| rs12945601 | 0.04 | 0.03 | 9.04E-02 |
| rs12970134 | 0.04 | 0.03 | 9.31E-02 |
| rs13234269 | 0.04 | 0.03 | 9.57E-02 |
| rs13239186 | 0.04 | 0.03 | 8.78E-02 |
| rs13330951 | 0.04 | 0.03 | 8.83E-02 |
| rs13389219 | 0.04 | 0.03 | 1.16E-01 |
| rs1359790 | 0.05 | 0.03 | 8.20E-02 |
| rs1496653 | 0.05 | 0.03 | 7.97E-02 |
| rs1552224 | 0.05 | 0.03 | 7.64E-02 |
| rs16988333 | 0.05 | 0.03 | 8.60E-02 |
| rs17086692 | 0.04 | 0.03 | 8.68E-02 |
| rs17168486 | 0.05 | 0.03 | 8.25E-02 |
| rs17405722 | 0.05 | 0.03 | 8.47E-02 |
| rs17411031 | 0.04 | 0.02 | 1.06E-01 |
| rs1758632 | 0.04 | 0.03 | 8.92E-02 |
| rs17631783 | 0.05 | 0.03 | 8.55E-02 |
| rs17791513 | 0.05 | 0.03 | 8.50E-02 |
| rs1801214 | 0.05 | 0.03 | 8.36E-02 |
| rs1899951 | 0.04 | 0.03 | 9.76E-02 |
| rs2058913 | 0.04 | 0.03 | 9.13E-02 |
| rs2237892 | 0.05 | 0.03 | 8.66E-02 |
| rs2246618 | 0.04 | 0.03 | 8.72E-02 |
| rs2261181 | 0.04 | 0.03 | 9.03E-02 |
| rs2294120 | 0.05 | 0.03 | 8.40E-02 |
| rs2296173 | 0.04 | 0.03 | 9.89E-02 |
| rs2299383 | 0.04 | 0.03 | 8.89E-02 |
| rs243019 | 0.05 | 0.03 | 8.23E-02 |
| rs2493394 | 0.05 | 0.03 | 8.64E-02 |
| rs2796441 | 0.05 | 0.03 | 7.76E-02 |
| rs2820426 | 0.04 | 0.03 | 9.81E-02 |
| rs2867125 | 0.04 | 0.03 | 8.90E-02 |
| rs2908282 | 0.05 | 0.03 | 8.64E-02 |
| rs2925979 | 0.04 | 0.03 | 9.98E-02 |
| rs2943656 | 0.04 | 0.03 | 1.24E-01 |
| rs3217992 | 0.05 | 0.03 | 8.26E-02 |
| rs340874 | 0.04 | 0.03 | 9.60E-02 |
| rs3756784 | 0.04 | 0.03 | 8.96E-02 |
| rs3802177 | 0.04 | 0.03 | 9.11E-02 |
| rs459193 | 0.04 | 0.03 | 1.00E-01 |
| rs4622883 | 0.04 | 0.03 | 8.67E-02 |
| rs4812829 | 0.05 | 0.03 | 8.39E-02 |
| rs4823182 | 0.05 | 0.03 | 8.48E-02 |
| rs4865796 | 0.04 | 0.03 | 9.17E-02 |
| rs516946 | 0.05 | 0.03 | 7.78E-02 |
| rs5215 | 0.05 | 0.03 | 7.77E-02 |
| rs55966194 | 0.04 | 0.03 | 9.39E-02 |
| rs576674 | 0.04 | 0.03 | 8.72E-02 |
| rs6059662 | 0.04 | 0.03 | 9.06E-02 |
| rs622217 | 0.04 | 0.03 | 9.20E-02 |
| rs6494307 | 0.04 | 0.03 | 8.70E-02 |
| rs6515236 | 0.05 | 0.03 | 8.32E-02 |
| rs6767484 | 0.04 | 0.03 | 9.24E-02 |
| rs6785040 | 0.05 | 0.03 | 8.53E-02 |
| rs6795735 | 0.04 | 0.03 | 9.06E-02 |
| rs6878122 | 0.05 | 0.03 | 8.63E-02 |
| rs6960043 | 0.05 | 0.03 | 8.54E-02 |
| rs7144011 | 0.04 | 0.03 | 8.79E-02 |
| rs7177055 | 0.05 | 0.03 | 8.28E-02 |
| rs7240767 | 0.05 | 0.03 | 8.55E-02 |
| rs72802358 | 0.05 | 0.03 | 8.36E-02 |
| rs72892910 | 0.05 | 0.03 | 8.44E-02 |
| rs735949 | 0.04 | 0.03 | 8.82E-02 |
| rs753270 | 0.04 | 0.03 | 8.88E-02 |
| rs7561798 | 0.04 | 0.03 | 8.66E-02 |
| rs7572970 | 0.04 | 0.03 | 8.87E-02 |
| rs7607777 | 0.05 | 0.03 | 8.76E-02 |
| rs7685296 | 0.04 | 0.03 | 8.98E-02 |
| rs7729395 | 0.04 | 0.03 | 8.86E-02 |
| rs7756992 | 0.04 | 0.03 | 1.01E-01 |
| rs7786095 | 0.05 | 0.03 | 8.54E-02 |
| rs780094 | 0.06 | 0.02 | 1.85E-03 |
| rs7845219 | 0.04 | 0.03 | 8.99E-02 |
| rs7903146 | 0.05 | 0.03 | 5.83E-02 |
| rs7929543 | 0.04 | 0.03 | 8.87E-02 |
| rs7955901 | 0.05 | 0.03 | 8.59E-02 |
| rs8068804 | 0.05 | 0.03 | 8.38E-02 |
| rs8108269 | 0.04 | 0.03 | 8.97E-02 |
| rs825476 | 0.04 | 0.03 | 9.28E-02 |
| rs840967 | 0.04 | 0.03 | 8.73E-02 |
| rs849135 | 0.05 | 0.03 | 8.42E-02 |
| rs9369425 | 0.05 | 0.03 | 8.12E-02 |
| rs963740 | 0.05 | 0.03 | 8.05E-02 |
| rs9844972 | 0.04 | 0.03 | 9.20E-02 |
| rs9894220 | 0.04 | 0.03 | 8.67E-02 |
| rs9928094 | 0.04 | 0.03 | 1.16E-01 |
| rs993380 | 0.05 | 0.03 | 8.49E-02 |
| rs9940149 | 0.05 | 0.03 | 8.28E-02 |
| All | 0.05 | 0.03 | 8.40E-02 |

T2DM: Type-2 diabetes; TG: Triglycerides; Se: Standard error.

Table 63: Single SNP analysis for estimate of T2DM-TG(Prins).

| SNP | Effect | Se | P-value |
| --- | --- | --- | --- |
| rs10077431 | -0.41 | 0.41 | 3.17E-01 |
| rs10100265 | -0.20 | 0.41 | 6.17E-01 |
| rs10114341 | 0.24 | 0.24 | 3.17E-01 |
| rs10401969 | -1.41 | 0.33 | 1.47E-05 |
| rs1050226 | 0.41 | 0.41 | 3.17E-01 |
| rs1061813 | -0.23 | 0.23 | 3.17E-01 |
| rs1063355 | -0.42 | 0.28 | 1.34E-01 |
| rs10740322 | -0.21 | 0.42 | 6.17E-01 |
| rs10830963 | 0.11 | 0.22 | 6.17E-01 |
| rs10842994 | -0.13 | 0.26 | 6.17E-01 |
| rs11098676 | 0.37 | 0.37 | 3.17E-01 |
| rs11107116 | 0.43 | 0.43 | 3.17E-01 |
| rs11257655 | -0.27 | 0.27 | 3.17E-01 |
| rs11708067 | 0.10 | 0.21 | 6.17E-01 |
| rs11925227 | 0.19 | 0.37 | 6.17E-01 |
| rs11926707 | -0.22 | 0.43 | 6.17E-01 |
| rs12088739 | 0.57 | 0.34 | 9.56E-02 |
| rs12910825 | 0.58 | 0.39 | 1.34E-01 |
| rs12945601 | -0.21 | 0.42 | 6.17E-01 |
| rs12970134 | 0.18 | 0.36 | 6.17E-01 |
| rs13234269 | 0.69 | 0.17 | 6.33E-05 |
| rs13239186 | 0.19 | 0.37 | 6.17E-01 |
| rs13330951 | 0.22 | 0.22 | 3.17E-01 |
| rs13389219 | 0.42 | 0.28 | 1.34E-01 |
| rs1496653 | 0.26 | 0.26 | 3.17E-01 |
| rs1552224 | 0.19 | 0.19 | 3.17E-01 |
| rs16988333 | 0.13 | 0.40 | 7.39E-01 |
| rs17086692 | -0.21 | 0.43 | 6.17E-01 |
| rs17168486 | 0.40 | 0.27 | 1.34E-01 |
| rs17405722 | 0.23 | 0.34 | 5.05E-01 |
| rs17411031 | 2.89 | 0.44 | 8.03E-11 |
| rs1758632 | 0.61 | 0.41 | 1.34E-01 |
| rs17631783 | 0.21 | 0.41 | 6.17E-01 |
| rs17791513 | 0.49 | 0.29 | 9.56E-02 |
| rs1801214 | 0.11 | 0.22 | 6.17E-01 |
| rs1899951 | 0.09 | 0.18 | 6.17E-01 |
| rs2058913 | 0.20 | 0.41 | 6.17E-01 |
| rs2237892 | 0.31 | 0.31 | 3.17E-01 |
| rs2261181 | 0.10 | 0.20 | 6.17E-01 |
| rs2294120 | -0.23 | 0.23 | 3.17E-01 |
| rs2296173 | 0.31 | 0.31 | 3.17E-01 |
| rs2299383 | 0.73 | 0.24 | 2.70E-03 |
| rs243019 | -0.18 | 0.35 | 6.17E-01 |
| rs2493394 | 0.27 | 0.27 | 3.17E-01 |
| rs2796441 | 0.14 | 0.14 | 3.17E-01 |
| rs2820426 | 0.58 | 0.38 | 1.34E-01 |
| rs2867125 | 0.17 | 0.33 | 6.17E-01 |
| rs2908282 | -0.18 | 0.36 | 6.17E-01 |
| rs2925979 | 0.56 | 0.37 | 1.34E-01 |
| rs2943656 | 0.22 | 0.22 | 3.17E-01 |
| rs3217992 | 0.19 | 0.38 | 6.17E-01 |
| rs348330 | 0.21 | 0.41 | 6.17E-01 |
| rs3756784 | 0.40 | 0.40 | 3.17E-01 |
| rs3802177 | 0.16 | 0.16 | 3.17E-01 |
| rs459193 | 0.42 | 0.28 | 1.34E-01 |
| rs4622883 | 0.69 | 0.23 | 2.70E-03 |
| rs4686471 | 0.19 | 0.37 | 6.17E-01 |
| rs4812829 | -0.75 | 0.38 | 4.55E-02 |
| rs4823182 | 0.21 | 0.41 | 6.17E-01 |
| rs4865796 | 0.19 | 0.38 | 6.17E-01 |
| rs516946 | 0.24 | 0.24 | 3.17E-01 |
| rs5215 | 0.29 | 0.29 | 3.17E-01 |
| rs55966194 | 0.19 | 0.38 | 6.17E-01 |
| rs576674 | -0.15 | 0.31 | 6.17E-01 |
| rs6059662 | 0.45 | 0.45 | 3.17E-01 |
| rs61953351 | -0.29 | 0.29 | 3.17E-01 |
| rs622217 | 0.41 | 0.41 | 3.17E-01 |
| rs6494307 | -0.23 | 0.45 | 6.17E-01 |
| rs6515236 | -0.20 | 0.40 | 6.17E-01 |
| rs67232546 | -0.34 | 0.34 | 3.17E-01 |
| rs6767484 | 0.33 | 0.17 | 4.55E-02 |
| rs6785040 | 0.47 | 0.32 | 1.34E-01 |
| rs6795735 | -0.36 | 0.36 | 3.17E-01 |
| rs6960043 | 0.16 | 0.31 | 6.17E-01 |
| rs7144011 | -0.41 | 0.41 | 3.17E-01 |
| rs7177055 | 0.31 | 0.31 | 3.17E-01 |
| rs7240767 | 0.22 | 0.22 | 3.17E-01 |
| rs72802358 | -0.17 | 0.17 | 3.17E-01 |
| rs72892910 | -0.15 | 0.31 | 6.17E-01 |
| rs735949 | -0.42 | 0.28 | 1.34E-01 |
| rs753270 | 0.38 | 0.38 | 3.17E-01 |
| rs7561798 | 0.25 | 0.25 | 3.17E-01 |
| rs7572970 | -0.17 | 0.34 | 6.17E-01 |
| rs7607777 | 0.07 | 0.15 | 6.17E-01 |
| rs7674212 | 0.65 | 0.43 | 1.34E-01 |
| rs7685296 | 0.20 | 0.39 | 6.17E-01 |
| rs7729395 | 0.29 | 0.22 | 1.82E-01 |
| rs7756992 | -0.08 | 0.15 | 6.17E-01 |
| rs7786095 | -0.13 | 0.27 | 6.17E-01 |
| rs780094 | -1.01 | 0.29 | 4.65E-04 |
| rs7845219 | 0.24 | 0.47 | 6.17E-01 |
| rs7929543 | -0.48 | 0.36 | 1.82E-01 |
| rs7955901 | 0.90 | 0.45 | 4.55E-02 |
| rs8068804 | 0.34 | 0.34 | 3.17E-01 |
| rs8108269 | 0.16 | 0.31 | 6.17E-01 |
| rs825476 | 0.19 | 0.19 | 3.17E-01 |
| rs840967 | 0.20 | 0.40 | 6.17E-01 |
| rs849135 | 0.20 | 0.10 | 4.55E-02 |
| rs9369425 | 0.18 | 0.37 | 6.17E-01 |
| rs963740 | 0.42 | 0.42 | 3.17E-01 |
| rs9844972 | 0.31 | 0.31 | 3.17E-01 |
| rs9894220 | 0.34 | 0.34 | 3.17E-01 |
| rs9928094 | 0.29 | 0.19 | 1.34E-01 |
| rs993380 | 0.20 | 0.39 | 6.17E-01 |
| rs9940149 | -0.17 | 0.34 | 6.17E-01 |
| All - Inverse variance weighted | 0.14 | 0.03 | 3.29E-05 |
| All - MR Egger | 0.00 | 0.10 | 9.79E-01 |

T2DM: Type-2 diabetes; TG: Triglycerides; Se: Standard error.

Table 64: Leave-one-out analysis for estimate of T2DM-TG(Prins).

| SNP | Effect | Se | P-value |
| --- | --- | --- | --- |
| rs10077431 | 0.15 | 0.03 | 2.54E-05 |
| rs10100265 | 0.14 | 0.03 | 2.99E-05 |
| rs10114341 | 0.14 | 0.03 | 4.59E-05 |
| rs10401969 | 0.15 | 0.03 | 2.22E-06 |
| rs1050226 | 0.14 | 0.03 | 4.20E-05 |
| rs1061813 | 0.15 | 0.03 | 1.89E-05 |
| rs1063355 | 0.15 | 0.03 | 1.65E-05 |
| rs10740322 | 0.14 | 0.03 | 3.01E-05 |
| rs10830963 | 0.14 | 0.03 | 3.85E-05 |
| rs10842994 | 0.15 | 0.03 | 2.62E-05 |
| rs11098676 | 0.14 | 0.03 | 4.27E-05 |
| rs11107116 | 0.14 | 0.03 | 4.17E-05 |
| rs11257655 | 0.15 | 0.03 | 2.09E-05 |
| rs11708067 | 0.14 | 0.03 | 3.83E-05 |
| rs11925227 | 0.14 | 0.03 | 3.86E-05 |
| rs11926707 | 0.14 | 0.03 | 3.03E-05 |
| rs12088739 | 0.14 | 0.03 | 4.84E-05 |
| rs12910825 | 0.14 | 0.03 | 4.54E-05 |
| rs12945601 | 0.14 | 0.03 | 3.00E-05 |
| rs12970134 | 0.14 | 0.03 | 3.87E-05 |
| rs13234269 | 0.13 | 0.03 | 1.34E-04 |
| rs13239186 | 0.14 | 0.03 | 3.86E-05 |
| rs13330951 | 0.14 | 0.03 | 4.68E-05 |
| rs13389219 | 0.14 | 0.03 | 5.06E-05 |
| rs1496653 | 0.14 | 0.03 | 4.54E-05 |
| rs1552224 | 0.14 | 0.03 | 4.77E-05 |
| rs16988333 | 0.14 | 0.03 | 3.72E-05 |
| rs17086692 | 0.14 | 0.03 | 3.02E-05 |
| rs17168486 | 0.14 | 0.03 | 5.12E-05 |
| rs17405722 | 0.14 | 0.03 | 4.03E-05 |
| rs17411031 | 0.13 | 0.03 | 1.40E-05 |
| rs1758632 | 0.14 | 0.03 | 4.47E-05 |
| rs17631783 | 0.14 | 0.03 | 3.85E-05 |
| rs17791513 | 0.14 | 0.03 | 5.13E-05 |
| rs1801214 | 0.14 | 0.03 | 3.85E-05 |
| rs1899951 | 0.14 | 0.03 | 3.76E-05 |
| rs2058913 | 0.14 | 0.03 | 3.85E-05 |
| rs2237892 | 0.14 | 0.03 | 4.40E-05 |
| rs2261181 | 0.14 | 0.03 | 3.82E-05 |
| rs2294120 | 0.15 | 0.03 | 1.84E-05 |
| rs2296173 | 0.14 | 0.03 | 4.41E-05 |
| rs2299383 | 0.14 | 0.03 | 7.10E-05 |
| rs243019 | 0.14 | 0.03 | 2.89E-05 |
| rs2493394 | 0.14 | 0.03 | 4.50E-05 |
| rs2796441 | 0.14 | 0.04 | 4.88E-05 |
| rs2820426 | 0.14 | 0.03 | 4.55E-05 |
| rs2867125 | 0.14 | 0.03 | 3.87E-05 |
| rs2908282 | 0.14 | 0.03 | 2.91E-05 |
| rs2925979 | 0.14 | 0.03 | 4.58E-05 |
| rs2943656 | 0.14 | 0.03 | 4.67E-05 |
| rs3217992 | 0.14 | 0.03 | 3.86E-05 |
| rs348330 | 0.14 | 0.03 | 3.85E-05 |
| rs3756784 | 0.14 | 0.03 | 4.22E-05 |
| rs3802177 | 0.14 | 0.04 | 4.86E-05 |
| rs459193 | 0.14 | 0.03 | 5.04E-05 |
| rs4622883 | 0.14 | 0.03 | 7.51E-05 |
| rs4686471 | 0.14 | 0.03 | 3.86E-05 |
| rs4812829 | 0.15 | 0.03 | 1.56E-05 |
| rs4823182 | 0.14 | 0.03 | 3.85E-05 |
| rs4865796 | 0.14 | 0.03 | 3.86E-05 |
| rs516946 | 0.14 | 0.03 | 4.60E-05 |
| rs5215 | 0.14 | 0.03 | 4.44E-05 |
| rs55966194 | 0.14 | 0.03 | 3.86E-05 |
| rs576674 | 0.15 | 0.03 | 2.77E-05 |
| rs6059662 | 0.14 | 0.03 | 4.14E-05 |
| rs61953351 | 0.15 | 0.03 | 2.16E-05 |
| rs622217 | 0.14 | 0.03 | 4.19E-05 |
| rs6494307 | 0.14 | 0.03 | 3.05E-05 |
| rs6515236 | 0.14 | 0.03 | 2.97E-05 |
| rs67232546 | 0.15 | 0.03 | 2.34E-05 |
| rs6767484 | 0.14 | 0.03 | 7.69E-05 |
| rs6785040 | 0.14 | 0.03 | 4.84E-05 |
| rs6795735 | 0.15 | 0.03 | 2.41E-05 |
| rs6960043 | 0.14 | 0.03 | 3.88E-05 |
| rs7144011 | 0.15 | 0.03 | 2.54E-05 |
| rs7177055 | 0.14 | 0.03 | 4.40E-05 |
| rs7240767 | 0.14 | 0.03 | 4.67E-05 |
| rs72802358 | 0.15 | 0.03 | 1.40E-05 |
| rs72892910 | 0.15 | 0.03 | 2.77E-05 |
| rs735949 | 0.15 | 0.03 | 1.65E-05 |
| rs753270 | 0.14 | 0.03 | 4.25E-05 |
| rs7561798 | 0.14 | 0.03 | 4.57E-05 |
| rs7572970 | 0.14 | 0.03 | 2.86E-05 |
| rs7607777 | 0.15 | 0.04 | 3.56E-05 |
| rs7674212 | 0.14 | 0.03 | 4.40E-05 |
| rs7685296 | 0.14 | 0.03 | 3.86E-05 |
| rs7729395 | 0.14 | 0.03 | 5.25E-05 |
| rs7756992 | 0.15 | 0.03 | 1.82E-05 |
| rs7786095 | 0.15 | 0.03 | 2.64E-05 |
| rs780094 | 0.15 | 0.03 | 4.04E-06 |
| rs7845219 | 0.14 | 0.03 | 3.83E-05 |
| rs7929543 | 0.15 | 0.03 | 2.10E-05 |
| rs7955901 | 0.14 | 0.03 | 4.45E-05 |
| rs8068804 | 0.14 | 0.03 | 4.33E-05 |
| rs8108269 | 0.14 | 0.03 | 3.88E-05 |
| rs825476 | 0.14 | 0.03 | 4.78E-05 |
| rs840967 | 0.14 | 0.03 | 3.85E-05 |
| rs849135 | 0.14 | 0.04 | 1.09E-04 |
| rs9369425 | 0.14 | 0.03 | 3.86E-05 |
| rs963740 | 0.14 | 0.03 | 4.18E-05 |
| rs9844972 | 0.14 | 0.03 | 4.39E-05 |
| rs9894220 | 0.14 | 0.03 | 4.33E-05 |
| rs9928094 | 0.14 | 0.03 | 5.84E-05 |
| rs993380 | 0.14 | 0.03 | 3.85E-05 |
| rs9940149 | 0.14 | 0.03 | 2.87E-05 |
| All | 0.14 | 0.03 | 3.29E-05 |

T2DM: Type-2 diabetes; TG: Triglycerides; Se: Standard error.

Table 65: Single SNP analysis for estimate of T2DM-TG(Kettunen).

| SNP | Effect | Se | P-value |
| --- | --- | --- | --- |
| rs10077431 | 0.46 | 0.27 | 8.35E-02 |
| rs10087241 | 0.10 | 0.22 | 6.37E-01 |
| rs10100265 | -0.31 | 0.20 | 1.22E-01 |
| rs10114341 | 0.32 | 0.24 | 1.92E-01 |
| rs10401969 | -1.70 | 0.22 | 5.78E-15 |
| rs1050226 | 0.15 | 0.21 | 4.70E-01 |
| rs1061813 | 0.22 | 0.24 | 3.70E-01 |
| rs1063355 | -0.06 | 0.15 | 6.68E-01 |
| rs10740322 | -0.08 | 0.23 | 7.17E-01 |
| rs10811661 | -0.03 | 0.09 | 7.44E-01 |
| rs10830963 | -0.13 | 0.12 | 2.84E-01 |
| rs10842994 | 0.08 | 0.17 | 6.61E-01 |
| rs10974438 | 0.07 | 0.17 | 6.97E-01 |
| rs11098676 | -0.08 | 0.23 | 7.44E-01 |
| rs11107116 | 0.62 | 0.24 | 9.93E-03 |
| rs1111875 | 0.26 | 0.11 | 1.48E-02 |
| rs11257655 | -0.03 | 0.16 | 8.71E-01 |
| rs1127655 | 0.15 | 0.22 | 4.99E-01 |
| rs11708067 | 0.13 | 0.13 | 3.27E-01 |
| rs11925227 | -0.32 | 0.25 | 2.14E-01 |
| rs11926707 | 0.35 | 0.23 | 1.31E-01 |
| rs12088739 | -0.08 | 0.24 | 7.29E-01 |
| rs12299509 | -0.15 | 0.23 | 4.96E-01 |
| rs12617659 | 0.25 | 0.20 | 2.27E-01 |
| rs12910825 | -0.28 | 0.20 | 1.50E-01 |
| rs12945601 | -0.07 | 0.21 | 7.21E-01 |
| rs12970134 | 0.49 | 0.22 | 2.56E-02 |
| rs13234269 | 0.29 | 0.17 | 8.56E-02 |
| rs13239186 | -0.08 | 0.20 | 6.73E-01 |
| rs13330951 | 0.42 | 0.22 | 5.51E-02 |
| rs13389219 | 0.44 | 0.14 | 1.87E-03 |
| rs1359790 | -0.40 | 0.14 | 4.47E-03 |
| rs1496653 | -0.08 | 0.14 | 5.86E-01 |
| rs1552224 | -0.09 | 0.11 | 4.01E-01 |
| rs16988333 | 0.17 | 0.23 | 4.64E-01 |
| rs17086692 | 0.02 | 0.22 | 9.38E-01 |
| rs17168486 | 0.10 | 0.16 | 5.35E-01 |
| rs17405722 | -0.10 | 0.20 | 6.02E-01 |
| rs17411031 | 1.92 | 0.25 | 1.05E-14 |
| rs1758632 | 0.00 | 0.20 | 9.97E-01 |
| rs17631783 | 0.08 | 0.24 | 7.24E-01 |
| rs17791513 | 0.01 | 0.16 | 9.46E-01 |
| rs1801214 | 0.09 | 0.11 | 4.05E-01 |
| rs1899951 | 0.12 | 0.12 | 3.18E-01 |
| rs2058913 | 0.23 | 0.20 | 2.66E-01 |
| rs2237892 | 0.20 | 0.23 | 3.81E-01 |
| rs2246618 | 0.45 | 0.21 | 3.38E-02 |
| rs2261181 | 0.04 | 0.19 | 8.46E-01 |
| rs2294120 | -0.06 | 0.22 | 7.71E-01 |
| rs2296173 | 0.25 | 0.19 | 1.82E-01 |
| rs2299383 | 0.09 | 0.24 | 7.18E-01 |
| rs243019 | -0.56 | 0.17 | 1.40E-03 |
| rs2493394 | 0.11 | 0.20 | 5.96E-01 |
| rs2796441 | 0.07 | 0.14 | 6.48E-01 |
| rs2820426 | 0.32 | 0.20 | 1.02E-01 |
| rs2867125 | 0.32 | 0.22 | 1.59E-01 |
| rs2908282 | -0.07 | 0.27 | 8.07E-01 |
| rs2925979 | -0.06 | 0.20 | 7.85E-01 |
| rs2943656 | 0.32 | 0.11 | 3.72E-03 |
| rs3217992 | -0.08 | 0.19 | 6.65E-01 |
| rs340874 | 0.29 | 0.16 | 7.00E-02 |
| rs348330 | 0.00 | 0.22 | 9.99E-01 |
| rs3756784 | -0.23 | 0.24 | 3.50E-01 |
| rs3802177 | 0.04 | 0.08 | 6.52E-01 |
| rs459193 | 0.01 | 0.15 | 9.52E-01 |
| rs4622883 | 0.30 | 0.23 | 1.88E-01 |
| rs4686471 | -0.14 | 0.19 | 4.68E-01 |
| rs4812829 | -0.08 | 0.23 | 7.15E-01 |
| rs4823182 | 0.28 | 0.21 | 1.79E-01 |
| rs4865796 | 0.38 | 0.20 | 5.67E-02 |
| rs516946 | -0.02 | 0.14 | 8.90E-01 |
| rs5215 | 0.06 | 0.15 | 6.64E-01 |
| rs55966194 | 0.33 | 0.23 | 1.43E-01 |
| rs576674 | 0.29 | 0.24 | 2.33E-01 |
| rs6059662 | -0.02 | 0.26 | 9.52E-01 |
| rs61953351 | 0.06 | 0.16 | 6.90E-01 |
| rs622217 | 0.16 | 0.20 | 4.38E-01 |
| rs6494307 | -0.21 | 0.22 | 3.56E-01 |
| rs6515236 | 0.35 | 0.24 | 1.44E-01 |
| rs67232546 | -0.26 | 0.23 | 2.57E-01 |
| rs6767484 | 0.25 | 0.09 | 4.79E-03 |
| rs6785040 | 0.28 | 0.19 | 1.52E-01 |
| rs6795735 | -0.01 | 0.18 | 9.35E-01 |
| rs6878122 | 0.15 | 0.21 | 4.58E-01 |
| rs6960043 | -0.06 | 0.16 | 6.84E-01 |
| rs7144011 | 0.02 | 0.24 | 9.34E-01 |
| rs7177055 | 0.28 | 0.17 | 9.35E-02 |
| rs7240767 | 0.16 | 0.23 | 4.87E-01 |
| rs72802358 | 0.36 | 0.15 | 1.96E-02 |
| rs72892910 | 0.46 | 0.19 | 1.54E-02 |
| rs735949 | 0.10 | 0.22 | 6.52E-01 |
| rs753270 | 0.27 | 0.19 | 1.60E-01 |
| rs7561798 | -0.22 | 0.25 | 3.82E-01 |
| rs7572970 | 0.17 | 0.20 | 4.13E-01 |
| rs7607777 | 0.21 | 0.15 | 1.81E-01 |
| rs7674212 | 0.11 | 0.21 | 6.24E-01 |
| rs7685296 | 0.16 | 0.22 | 4.62E-01 |
| rs7729395 | 0.01 | 0.18 | 9.73E-01 |
| rs7756992 | 0.09 | 0.08 | 2.45E-01 |
| rs7786095 | 0.02 | 0.26 | 9.45E-01 |
| rs780094 | -1.46 | 0.15 | 3.24E-23 |
| rs7845219 | -0.10 | 0.23 | 6.79E-01 |
| rs7903146 | 0.07 | 0.04 | 9.70E-02 |
| rs7929543 | 0.20 | 0.23 | 3.86E-01 |
| rs7955901 | -0.43 | 0.23 | 6.09E-02 |
| rs8068804 | 0.19 | 0.18 | 2.87E-01 |
| rs8108269 | 0.03 | 0.17 | 8.62E-01 |
| rs825476 | 0.60 | 0.19 | 1.62E-03 |
| rs840967 | 0.08 | 0.20 | 6.98E-01 |
| rs849135 | 0.12 | 0.10 | 2.28E-01 |
| rs853974 | -0.02 | 0.19 | 9.25E-01 |
| rs9369425 | -0.35 | 0.20 | 8.52E-02 |
| rs963740 | -0.24 | 0.24 | 3.24E-01 |
| rs9844972 | 0.03 | 0.23 | 8.80E-01 |
| rs9894220 | -0.22 | 0.17 | 2.01E-01 |
| rs9928094 | 0.16 | 0.10 | 8.36E-02 |
| rs993380 | 0.16 | 0.20 | 4.35E-01 |
| rs9940149 | 0.21 | 0.27 | 4.32E-01 |
| All - Inverse variance weighted | 0.07 | 0.03 | 1.43E-02 |
| All - MR Egger | 0.05 | 0.06 | 4.32E-01 |

T2DM: Type-2 diabetes; TG: Triglycerides; Se: Standard error.

Table 66: Leave-one-out analysis for estimate of T2DM-TG(Kettunen).

| SNP | Effect | Se | P-value |
| --- | --- | --- | --- |
| rs10077431 | 0.06 | 0.03 | 1.66E-02 |
| rs10087241 | 0.06 | 0.03 | 1.53E-02 |
| rs10100265 | 0.07 | 0.03 | 1.18E-02 |
| rs10114341 | 0.06 | 0.03 | 1.63E-02 |
| rs10401969 | 0.07 | 0.02 | 2.65E-03 |
| rs1050226 | 0.06 | 0.03 | 1.57E-02 |
| rs1061813 | 0.06 | 0.03 | 1.58E-02 |
| rs1063355 | 0.07 | 0.03 | 1.33E-02 |
| rs10740322 | 0.07 | 0.03 | 1.40E-02 |
| rs10811661 | 0.07 | 0.03 | 1.22E-02 |
| rs10830963 | 0.07 | 0.03 | 1.12E-02 |
| rs10842994 | 0.07 | 0.03 | 1.53E-02 |
| rs10974438 | 0.07 | 0.03 | 1.52E-02 |
| rs11098676 | 0.07 | 0.03 | 1.41E-02 |
| rs11107116 | 0.06 | 0.03 | 1.77E-02 |
| rs1111875 | 0.06 | 0.03 | 2.24E-02 |
| rs11257655 | 0.07 | 0.03 | 1.40E-02 |
| rs1127655 | 0.06 | 0.03 | 1.56E-02 |
| rs11708067 | 0.06 | 0.03 | 1.67E-02 |
| rs11925227 | 0.07 | 0.03 | 1.28E-02 |
| rs11926707 | 0.06 | 0.03 | 1.66E-02 |
| rs12088739 | 0.07 | 0.03 | 1.41E-02 |
| rs12299509 | 0.07 | 0.03 | 1.35E-02 |
| rs12617659 | 0.06 | 0.03 | 1.64E-02 |
| rs12910825 | 0.07 | 0.03 | 1.19E-02 |
| rs12945601 | 0.07 | 0.03 | 1.39E-02 |
| rs12970134 | 0.06 | 0.03 | 1.77E-02 |
| rs13234269 | 0.06 | 0.03 | 1.78E-02 |
| rs13239186 | 0.07 | 0.03 | 1.37E-02 |
| rs13330951 | 0.06 | 0.03 | 1.73E-02 |
| rs13389219 | 0.06 | 0.03 | 2.17E-02 |
| rs1359790 | 0.07 | 0.03 | 7.88E-03 |
| rs1496653 | 0.07 | 0.03 | 1.29E-02 |
| rs1552224 | 0.07 | 0.03 | 1.15E-02 |
| rs16988333 | 0.06 | 0.03 | 1.56E-02 |
| rs17086692 | 0.07 | 0.03 | 1.47E-02 |
| rs17168486 | 0.06 | 0.03 | 1.56E-02 |
| rs17405722 | 0.07 | 0.03 | 1.36E-02 |
| rs17411031 | 0.06 | 0.02 | 1.78E-02 |
| rs1758632 | 0.07 | 0.03 | 1.45E-02 |
| rs17631783 | 0.07 | 0.03 | 1.51E-02 |
| rs17791513 | 0.07 | 0.03 | 1.45E-02 |
| rs1801214 | 0.06 | 0.03 | 1.65E-02 |
| rs1899951 | 0.06 | 0.03 | 1.69E-02 |
| rs2058913 | 0.06 | 0.03 | 1.63E-02 |
| rs2237892 | 0.06 | 0.03 | 1.58E-02 |
| rs2246618 | 0.06 | 0.03 | 1.77E-02 |
| rs2261181 | 0.07 | 0.03 | 1.48E-02 |
| rs2294120 | 0.07 | 0.03 | 1.41E-02 |
| rs2296173 | 0.06 | 0.03 | 1.68E-02 |
| rs2299383 | 0.07 | 0.03 | 1.51E-02 |
| rs243019 | 0.07 | 0.03 | 8.28E-03 |
| rs2493394 | 0.06 | 0.03 | 1.54E-02 |
| rs2796441 | 0.07 | 0.03 | 1.53E-02 |
| rs2820426 | 0.06 | 0.03 | 1.72E-02 |
| rs2867125 | 0.06 | 0.03 | 1.66E-02 |
| rs2908282 | 0.07 | 0.03 | 1.43E-02 |
| rs2925979 | 0.07 | 0.03 | 1.40E-02 |
| rs2943656 | 0.06 | 0.03 | 2.36E-02 |
| rs3217992 | 0.07 | 0.03 | 1.37E-02 |
| rs340874 | 0.06 | 0.03 | 1.82E-02 |
| rs348330 | 0.07 | 0.03 | 1.45E-02 |
| rs3756784 | 0.07 | 0.03 | 1.32E-02 |
| rs3802177 | 0.07 | 0.03 | 1.50E-02 |
| rs459193 | 0.07 | 0.03 | 1.44E-02 |
| rs4622883 | 0.06 | 0.03 | 1.64E-02 |
| rs4686471 | 0.07 | 0.03 | 1.31E-02 |
| rs4812829 | 0.07 | 0.03 | 1.40E-02 |
| rs4823182 | 0.06 | 0.03 | 1.66E-02 |
| rs4865796 | 0.06 | 0.03 | 1.76E-02 |
| rs516946 | 0.07 | 0.03 | 1.39E-02 |
| rs5215 | 0.07 | 0.03 | 1.53E-02 |
| rs55966194 | 0.06 | 0.03 | 1.66E-02 |
| rs576674 | 0.06 | 0.03 | 1.62E-02 |
| rs6059662 | 0.07 | 0.03 | 1.45E-02 |
| rs61953351 | 0.07 | 0.03 | 1.52E-02 |
| rs622217 | 0.06 | 0.03 | 1.58E-02 |
| rs6494307 | 0.07 | 0.03 | 1.31E-02 |
| rs6515236 | 0.06 | 0.03 | 1.65E-02 |
| rs67232546 | 0.07 | 0.03 | 1.28E-02 |
| rs6767484 | 0.06 | 0.03 | 2.63E-02 |
| rs6785040 | 0.06 | 0.03 | 1.69E-02 |
| rs6795735 | 0.07 | 0.03 | 1.43E-02 |
| rs6878122 | 0.06 | 0.03 | 1.57E-02 |
| rs6960043 | 0.07 | 0.03 | 1.34E-02 |
| rs7144011 | 0.07 | 0.03 | 1.47E-02 |
| rs7177055 | 0.06 | 0.03 | 1.78E-02 |
| rs7240767 | 0.06 | 0.03 | 1.56E-02 |
| rs72802358 | 0.06 | 0.03 | 1.95E-02 |
| rs72892910 | 0.06 | 0.03 | 1.86E-02 |
| rs735949 | 0.06 | 0.03 | 1.52E-02 |
| rs753270 | 0.06 | 0.03 | 1.69E-02 |
| rs7561798 | 0.07 | 0.03 | 1.33E-02 |
| rs7572970 | 0.06 | 0.03 | 1.58E-02 |
| rs7607777 | 0.06 | 0.03 | 1.73E-02 |
| rs7674212 | 0.06 | 0.03 | 1.53E-02 |
| rs7685296 | 0.06 | 0.03 | 1.57E-02 |
| rs7729395 | 0.07 | 0.03 | 1.45E-02 |
| rs7756992 | 0.06 | 0.03 | 1.83E-02 |
| rs7786095 | 0.07 | 0.03 | 1.47E-02 |
| rs780094 | 0.08 | 0.02 | 3.89E-04 |
| rs7845219 | 0.07 | 0.03 | 1.39E-02 |
| rs7903146 | 0.06 | 0.03 | 2.38E-02 |
| rs7929543 | 0.06 | 0.03 | 1.58E-02 |
| rs7955901 | 0.07 | 0.03 | 1.16E-02 |
| rs8068804 | 0.06 | 0.03 | 1.64E-02 |
| rs8108269 | 0.07 | 0.03 | 1.47E-02 |
| rs825476 | 0.06 | 0.03 | 1.96E-02 |
| rs840967 | 0.07 | 0.03 | 1.52E-02 |
| rs849135 | 0.06 | 0.03 | 1.80E-02 |
| rs853974 | 0.07 | 0.03 | 1.43E-02 |
| rs9369425 | 0.07 | 0.03 | 1.15E-02 |
| rs963740 | 0.07 | 0.03 | 1.31E-02 |
| rs9844972 | 0.07 | 0.03 | 1.48E-02 |
| rs9894220 | 0.07 | 0.03 | 1.19E-02 |
| rs9928094 | 0.06 | 0.03 | 2.02E-02 |
| rs993380 | 0.06 | 0.03 | 1.58E-02 |
| rs9940149 | 0.06 | 0.03 | 1.56E-02 |
| All | 0.07 | 0.03 | 1.43E-02 |

T2DM: Type-2 diabetes; TG: Triglycerides; Se: Standard error.

Table 67: Single SNP analysis for estimate of T2DM-TG(Kanai).

| SNP | Effect | Se | P-value |
| --- | --- | --- | --- |
| rs10077431 | 0.06 | 0.19 | 7.55E-01 |
| rs10087241 | 0.05 | 0.17 | 7.92E-01 |
| rs10100265 | -0.16 | 0.09 | 8.55E-02 |
| rs10114341 | -0.03 | 0.14 | 8.41E-01 |
| rs10401969 | -0.47 | 0.08 | 1.49E-09 |
| rs1050226 | 0.00 | 0.09 | 9.74E-01 |
| rs1061813 | 0.15 | 0.18 | 3.83E-01 |
| rs1063355 | -0.23 | 0.06 | 1.12E-04 |
| rs10740322 | -0.18 | 0.10 | 8.40E-02 |
| rs10811661 | -0.02 | 0.03 | 5.26E-01 |
| rs10830963 | 0.00 | 0.05 | 9.70E-01 |
| rs10842994 | -0.03 | 0.07 | 6.29E-01 |
| rs10974438 | -0.15 | 0.07 | 4.82E-02 |
| rs11098676 | 0.12 | 0.18 | 4.98E-01 |
| rs11107116 | -0.02 | 0.10 | 8.21E-01 |
| rs1111875 | -0.06 | 0.05 | 2.59E-01 |
| rs11257655 | -0.12 | 0.06 | 3.09E-02 |
| rs1127655 | 0.21 | 0.10 | 3.72E-02 |
| rs11926707 | 0.14 | 0.11 | 2.32E-01 |
| rs12088739 | 0.19 | 0.09 | 3.41E-02 |
| rs12299509 | 0.04 | 0.09 | 6.97E-01 |
| rs12617659 | 0.02 | 0.09 | 8.00E-01 |
| rs12945601 | -0.14 | 0.13 | 2.88E-01 |
| rs12970134 | 0.24 | 0.10 | 2.23E-02 |
| rs13234269 | 0.32 | 0.09 | 2.35E-04 |
| rs13239186 | 0.20 | 0.12 | 7.80E-02 |
| rs13330951 | -0.13 | 0.14 | 3.58E-01 |
| rs13389219 | 0.03 | 0.11 | 8.18E-01 |
| rs1359790 | -0.10 | 0.06 | 9.78E-02 |
| rs1496653 | -0.01 | 0.08 | 8.53E-01 |
| rs1552224 | -0.13 | 0.11 | 2.37E-01 |
| rs17086692 | 0.02 | 0.11 | 8.25E-01 |
| rs17168486 | -0.07 | 0.06 | 2.32E-01 |
| rs17405722 | -0.03 | 0.15 | 8.53E-01 |
| rs17411031 | 2.84 | 0.12 | 4.73E-121 |
| rs1758632 | 0.04 | 0.10 | 6.84E-01 |
| rs17631783 | 0.19 | 0.19 | 3.25E-01 |
| rs17791513 | -0.02 | 0.09 | 8.04E-01 |
| rs1801214 | -0.24 | 0.19 | 2.20E-01 |
| rs1899951 | 0.20 | 0.11 | 7.55E-02 |
| rs2058913 | -0.19 | 0.17 | 2.74E-01 |
| rs2237892 | -0.04 | 0.05 | 4.29E-01 |
| rs2246618 | -0.02 | 0.11 | 8.84E-01 |
| rs2261181 | 0.01 | 0.06 | 8.48E-01 |
| rs2294120 | 0.14 | 0.11 | 2.21E-01 |
| rs2296173 | 0.26 | 0.09 | 3.04E-03 |
| rs2299383 | 0.02 | 0.11 | 8.58E-01 |
| rs243019 | -0.06 | 0.08 | 4.56E-01 |
| rs2493394 | -0.43 | 0.22 | 5.43E-02 |
| rs2796441 | -0.12 | 0.06 | 4.76E-02 |
| rs2820426 | 0.19 | 0.08 | 2.06E-02 |
| rs2867125 | 0.19 | 0.12 | 1.14E-01 |
| rs2908282 | 0.14 | 0.10 | 1.43E-01 |
| rs2925979 | 0.40 | 0.08 | 1.80E-06 |
| rs2943656 | 0.37 | 0.08 | 8.46E-06 |
| rs3217992 | -0.04 | 0.08 | 6.16E-01 |
| rs340874 | -0.08 | 0.07 | 2.67E-01 |
| rs348330 | -0.02 | 0.11 | 8.30E-01 |
| rs3756784 | 0.00 | 0.09 | 9.70E-01 |
| rs3802177 | 0.01 | 0.04 | 7.46E-01 |
| rs459193 | 0.31 | 0.06 | 2.39E-07 |
| rs4622883 | -0.01 | 0.12 | 9.12E-01 |
| rs4812829 | 0.10 | 0.08 | 2.04E-01 |
| rs4823182 | -0.20 | 0.09 | 2.27E-02 |
| rs4865796 | 0.21 | 0.11 | 5.50E-02 |
| rs516946 | -0.08 | 0.07 | 2.80E-01 |
| rs5215 | -0.01 | 0.07 | 9.33E-01 |
| rs576674 | -0.17 | 0.10 | 8.84E-02 |
| rs6059662 | 0.18 | 0.12 | 1.43E-01 |
| rs622217 | -0.08 | 0.10 | 4.45E-01 |
| rs6494307 | -0.24 | 0.10 | 1.41E-02 |
| rs6515236 | -0.06 | 0.09 | 4.68E-01 |
| rs6767484 | 0.00 | 0.04 | 9.76E-01 |
| rs6785040 | -0.08 | 0.07 | 2.85E-01 |
| rs6795735 | -0.03 | 0.10 | 7.95E-01 |
| rs6878122 | -0.21 | 0.28 | 4.66E-01 |
| rs6960043 | -0.06 | 0.07 | 3.67E-01 |
| rs7177055 | -0.11 | 0.07 | 1.02E-01 |
| rs7240767 | -0.11 | 0.10 | 2.89E-01 |
| rs72802358 | -0.24 | 0.15 | 1.04E-01 |
| rs72892910 | 0.04 | 0.08 | 6.63E-01 |
| rs753270 | -0.01 | 0.10 | 9.00E-01 |
| rs7561798 | 0.07 | 0.12 | 5.79E-01 |
| rs7572970 | -0.01 | 0.10 | 9.42E-01 |
| rs7607777 | -0.03 | 0.16 | 8.56E-01 |
| rs7674212 | 0.09 | 0.10 | 4.01E-01 |
| rs7685296 | 0.10 | 0.08 | 2.54E-01 |
| rs7756992 | -0.05 | 0.03 | 9.92E-02 |
| rs7786095 | -0.07 | 0.11 | 5.32E-01 |
| rs780094 | -1.20 | 0.06 | 1.46E-83 |
| rs7845219 | -0.07 | 0.11 | 5.20E-01 |
| rs7903146 | -0.08 | 0.03 | 1.34E-02 |
| rs7929543 | 0.04 | 0.07 | 6.17E-01 |
| rs7955901 | 0.06 | 0.10 | 5.31E-01 |
| rs8068804 | -0.02 | 0.08 | 8.28E-01 |
| rs8108269 | 0.01 | 0.07 | 9.09E-01 |
| rs825476 | 0.12 | 0.10 | 2.34E-01 |
| rs840967 | -0.13 | 0.09 | 1.27E-01 |
| rs853974 | 0.14 | 0.08 | 6.55E-02 |
| rs9369425 | -0.32 | 0.13 | 9.94E-03 |
| rs963740 | -0.18 | 0.09 | 4.26E-02 |
| rs9894220 | 0.02 | 0.08 | 7.84E-01 |
| rs9928094 | 0.08 | 0.05 | 1.04E-01 |
| rs993380 | -0.11 | 0.09 | 2.10E-01 |
| rs9940149 | -0.10 | 0.08 | 1.75E-01 |
| All - Inverse variance weighted | -0.02 | 0.03 | 4.39E-01 |
| All - MR Egger | -0.09 | 0.07 | 1.72E-01 |

T2DM: Type-2 diabetes; TG: Triglycerides; Se: Standard error.

Table 68: Leave-one-out analysis for estimate of T2DM-TG(Kanai).

| SNP | Effect | Se | P-value |
| --- | --- | --- | --- |
| rs10077431 | -0.02 | 0.03 | 4.39E-01 |
| rs10087241 | -0.02 | 0.03 | 4.39E-01 |
| rs10100265 | -0.02 | 0.03 | 4.64E-01 |
| rs10114341 | -0.02 | 0.03 | 4.43E-01 |
| rs10401969 | -0.02 | 0.03 | 5.42E-01 |
| rs1050226 | -0.02 | 0.03 | 4.39E-01 |
| rs1061813 | -0.02 | 0.03 | 4.34E-01 |
| rs1063355 | -0.02 | 0.03 | 5.24E-01 |
| rs10740322 | -0.02 | 0.03 | 4.62E-01 |
| rs10811661 | -0.02 | 0.03 | 4.55E-01 |
| rs10830963 | -0.02 | 0.03 | 4.37E-01 |
| rs10842994 | -0.02 | 0.03 | 4.48E-01 |
| rs10974438 | -0.02 | 0.03 | 4.74E-01 |
| rs11098676 | -0.02 | 0.03 | 4.36E-01 |
| rs11107116 | -0.02 | 0.03 | 4.43E-01 |
| rs1111875 | -0.02 | 0.03 | 4.66E-01 |
| rs11257655 | -0.02 | 0.03 | 4.87E-01 |
| rs1127655 | -0.02 | 0.03 | 4.13E-01 |
| rs11926707 | -0.02 | 0.03 | 4.27E-01 |
| rs12088739 | -0.02 | 0.03 | 4.08E-01 |
| rs12299509 | -0.02 | 0.03 | 4.34E-01 |
| rs12617659 | -0.02 | 0.03 | 4.36E-01 |
| rs12945601 | -0.02 | 0.03 | 4.51E-01 |
| rs12970134 | -0.02 | 0.03 | 4.11E-01 |
| rs13234269 | -0.02 | 0.03 | 3.84E-01 |
| rs13239186 | -0.02 | 0.03 | 4.20E-01 |
| rs13330951 | -0.02 | 0.03 | 4.49E-01 |
| rs13389219 | -0.02 | 0.03 | 4.38E-01 |
| rs1359790 | -0.02 | 0.03 | 4.73E-01 |
| rs1496653 | -0.02 | 0.03 | 4.42E-01 |
| rs1552224 | -0.02 | 0.03 | 4.55E-01 |
| rs17086692 | -0.02 | 0.03 | 4.38E-01 |
| rs17168486 | -0.02 | 0.03 | 4.64E-01 |
| rs17405722 | -0.02 | 0.03 | 4.42E-01 |
| rs17411031 | -0.03 | 0.02 | 1.03E-01 |
| rs1758632 | -0.02 | 0.03 | 4.35E-01 |
| rs17631783 | -0.02 | 0.03 | 4.34E-01 |
| rs17791513 | -0.02 | 0.03 | 4.43E-01 |
| rs1801214 | -0.02 | 0.03 | 4.49E-01 |
| rs1899951 | -0.02 | 0.03 | 4.19E-01 |
| rs2058913 | -0.02 | 0.03 | 4.49E-01 |
| rs2237892 | -0.02 | 0.03 | 4.58E-01 |
| rs2246618 | -0.02 | 0.03 | 4.42E-01 |
| rs2261181 | -0.02 | 0.03 | 4.34E-01 |
| rs2294120 | -0.02 | 0.03 | 4.26E-01 |
| rs2296173 | -0.02 | 0.03 | 3.95E-01 |
| rs2299383 | -0.02 | 0.03 | 4.38E-01 |
| rs243019 | -0.02 | 0.03 | 4.51E-01 |
| rs2493394 | -0.02 | 0.03 | 4.52E-01 |
| rs2796441 | -0.02 | 0.03 | 4.81E-01 |
| rs2820426 | -0.02 | 0.03 | 4.02E-01 |
| rs2867125 | -0.02 | 0.03 | 4.23E-01 |
| rs2908282 | -0.02 | 0.03 | 4.20E-01 |
| rs2925979 | -0.02 | 0.03 | 3.65E-01 |
| rs2943656 | -0.02 | 0.03 | 3.68E-01 |
| rs3217992 | -0.02 | 0.03 | 4.48E-01 |
| rs340874 | -0.02 | 0.03 | 4.59E-01 |
| rs348330 | -0.02 | 0.03 | 4.43E-01 |
| rs3756784 | -0.02 | 0.03 | 4.40E-01 |
| rs3802177 | -0.02 | 0.03 | 4.19E-01 |
| rs459193 | -0.02 | 0.03 | 3.29E-01 |
| rs4622883 | -0.02 | 0.03 | 4.42E-01 |
| rs4812829 | -0.02 | 0.03 | 4.19E-01 |
| rs4823182 | -0.02 | 0.03 | 4.73E-01 |
| rs4865796 | -0.02 | 0.03 | 4.17E-01 |
| rs516946 | -0.02 | 0.03 | 4.58E-01 |
| rs5215 | -0.02 | 0.03 | 4.40E-01 |
| rs576674 | -0.02 | 0.03 | 4.62E-01 |
| rs6059662 | -0.02 | 0.03 | 4.25E-01 |
| rs622217 | -0.02 | 0.03 | 4.50E-01 |
| rs6494307 | -0.02 | 0.03 | 4.73E-01 |
| rs6515236 | -0.02 | 0.03 | 4.50E-01 |
| rs6767484 | -0.02 | 0.03 | 4.31E-01 |
| rs6785040 | -0.02 | 0.03 | 4.58E-01 |
| rs6795735 | -0.02 | 0.03 | 4.43E-01 |
| rs6878122 | -0.02 | 0.03 | 4.45E-01 |
| rs6960043 | -0.02 | 0.03 | 4.56E-01 |
| rs7177055 | -0.02 | 0.03 | 4.70E-01 |
| rs7240767 | -0.02 | 0.03 | 4.53E-01 |
| rs72802358 | -0.02 | 0.03 | 4.55E-01 |
| rs72892910 | -0.02 | 0.03 | 4.32E-01 |
| rs753270 | -0.02 | 0.03 | 4.42E-01 |
| rs7561798 | -0.02 | 0.03 | 4.34E-01 |
| rs7572970 | -0.02 | 0.03 | 4.41E-01 |
| rs7607777 | -0.02 | 0.03 | 4.42E-01 |
| rs7674212 | -0.02 | 0.03 | 4.29E-01 |
| rs7685296 | -0.02 | 0.03 | 4.22E-01 |
| rs7756992 | -0.02 | 0.03 | 4.98E-01 |
| rs7786095 | -0.02 | 0.03 | 4.48E-01 |
| rs780094 | 0.00 | 0.02 | 9.17E-01 |
| rs7845219 | -0.02 | 0.03 | 4.48E-01 |
| rs7903146 | -0.02 | 0.03 | 5.32E-01 |
| rs7929543 | -0.02 | 0.03 | 4.29E-01 |
| rs7955901 | -0.02 | 0.03 | 4.32E-01 |
| rs8068804 | -0.02 | 0.03 | 4.43E-01 |
| rs8108269 | -0.02 | 0.03 | 4.36E-01 |
| rs825476 | -0.02 | 0.03 | 4.24E-01 |
| rs840967 | -0.02 | 0.03 | 4.63E-01 |
| rs853974 | -0.02 | 0.03 | 4.08E-01 |
| rs9369425 | -0.02 | 0.03 | 4.67E-01 |
| rs963740 | -0.02 | 0.03 | 4.69E-01 |
| rs9894220 | -0.02 | 0.03 | 4.35E-01 |
| rs9928094 | -0.02 | 0.03 | 3.93E-01 |
| rs993380 | -0.02 | 0.03 | 4.58E-01 |
| rs9940149 | -0.02 | 0.03 | 4.62E-01 |
| All | -0.02 | 0.03 | 4.39E-01 |

T2DM: Type-2 diabetes; TG: Triglycerides; Se: Standard error.

## Table 69. Weighted Median, MR-Egger regression results for estimate of causal effect of T2DM on blood lipid profiles.

|  | Author | Methods | β | se | p-value |
| --- | --- | --- | --- | --- | --- |
| HDL | Kettunen | Weighted Median | -0.09 | 0.03 | 0.001 |
|  |  | MR-Egger regression | -0.06 | 0.05 | 0.246 |
|  |  | MR-Egger intercept | -1.80E-03 | 3.48E-03 | 0.606 |
|  |  | IVW | -0.08 | 0.02 | <0.001 |
|  | Prins | Weighted Median | -0.12 | 0.04 | 0.003 |
|  |  | MR-Egger regression | -0.01 | 0.06 | 0.928 |
|  |  | MR-Egger intercept | -0.01 | 4.41E-03 | 0.015 |
|  |  | IVW | -0.14 | 0.03 | <0.001 |
|  | Willer | Weighted Median | -3.82E-03 | 0.01 | 0.695 |
|  |  | MR-Egger regression | 0.06 | 0.04 | 0.144 |
|  |  | MR-Egger intercept | -0.01 | 3.08E-03 | 0.001 |
|  |  | IVW | -0.07 | 0.02 | 0.001 |
| LDL | Kanai | Weighted Median | 0.01 | 0.01 | 0.390 |
|  |  | MR-Egger regression | -3.06E-03 | 0.03 | 0.918 |
|  |  | MR-Egger intercept | 2.48E-04 | 2.02E-03 | 0.903 |
|  |  | IVW | 3.02E-04 | 0.01 | 0.978 |
|  | Kettunen | Weighted Median | 0.01 | 0.03 | 0.855 |
|  |  | MR-Egger regression | -0.01 | 0.04 | 0.858 |
|  |  | MR-Egger intercept | 2.51E-03 | 2.77E-03 | 0.367 |
|  |  | IVW | 0.03 | 0.02 | 0.124 |
|  | Prins | Weighted Median | -0.10 | 0.04 | 0.009 |
|  |  | MR-Egger regression | -0.08 | 0.06 | 0.234 |
|  |  | MR-Egger intercept | 9.13E-04 | 4.37E-03 | 0.835 |
|  |  | IVW | -0.06 | 0.03 | 0.012 |
|  | Willer | Weighted Median | 0.02 | 0.01 | 0.114 |
|  |  | MR-Egger regression | 0.01 | 0.03 | 0.607 |
|  |  | MR-Egger intercept | -3.74E-04 | 2.17E-03 | 0.863 |
|  |  | IVW | 0.01 | 0.01 | 0.420 |
| TC | Kanai | Weighted Median | 4.25E-03 | 0.01 | 0.704 |
|  |  | MR-Egger regression | 0.03 | 0.03 | 0.327 |
|  |  | MR-Egger intercept | -2.32E-03 | 1.85E-03 | 0.213 |
|  |  | IVW | -4.89E-03 | 0.01 | 0.625 |
|  | Kettunen | Weighted Median | 0.01 | 0.03 | 0.756 |
|  |  | MR-Egger regression | 0.01 | 0.05 | 0.867 |
|  |  | MR-Egger intercept | -1.84E-04 | 3.21E-03 | 0.954 |
|  |  | IVW | 0.01 | 0.02 | 0.782 |
|  | Prins | Weighted Median | -0.10 | 0.04 | 0.012 |
|  |  | MR-Egger regression | -0.09 | 0.07 | 0.167 |
|  |  | MR-Egger intercept | 2.01E-03 | 4.44E-03 | 0.652 |
|  |  | IVW | -0.06 | 0.03 | 0.018 |
|  | Willer | Weighted Median | 0.02 | 0.01 | 0.020 |
|  |  | MR-Egger regression | 0.03 | 0.03 | 0.367 |
|  |  | MR-Egger intercept | -2.60E-03 | 2.61E-03 | 0.321 |
|  |  | IVW | 6.04E-04 | 0.02 | 0.969 |
| TG | Kanai | Weighted Median | -0.02 | 0.01 | 0.133 |
|  |  | MR-Egger regression | -0.09 | 0.07 | 0.172 |
|  |  | MR-Egger intercept | 0.01 | 0.00 | 0.245 |
|  |  | IVW | -0.02 | 0.03 | 0.439 |
|  | Kettunen | Weighted Median | 0.07 | 0.03 | 0.017 |
|  |  | MR-Egger regression | 0.05 | 0.06 | 0.432 |
|  |  | MR-Egger intercept | 1.16E-03 | 4.52E-03 | 0.797 |
|  |  | IVW | 0.07 | 0.03 | 0.014 |
|  | Prins | Weighted Median | 0.19 | 0.04 | <0.001 |
|  |  | MR-Egger regression | 2.71E-03 | 0.10 | 0.979 |
|  |  | MR-Egger intercept | 0.01 | 0.01 | 0.151 |
|  |  | IVW | 0.14 | 0.03 | <0.001 |
|  | Willer | Weighted Median | 0.02 | 0.01 | 0.026 |
|  |  | MR-Egger regression | -0.03 | 0.06 | 0.590 |
|  |  | MR-Egger intercept | 0.01 | 4.38E-03 | 0.140 |
|  |  | IVW | 0.05 | 0.03 | 0.084 |

HDL: High-density lipoprotein, LDL: Low-density lipoprotein, TC: Total cholesterol, TG: Triglycerides, se: standard error
